# Dehomologative C–C Borylation of Aldehydes and Alcohols via a Rh-Catalyzed Dehydroformylation–Borylation Relay

**DOI:** 10.1021/jacs.5c02181

**Published:** 2025-05-12

**Authors:** Kuhali Das, Nikodem Kuźnik, Paweł Dydio

**Affiliations:** † Yusuf Hamied Department of Chemistry, 2152 University of Cambridge, Cambridge CB2 1EW, United Kingdom; ‡ University of Strasbourg, CNRS, ISIS UMR 7006, 67000 Strasbourg, France; § Silesian University of Technology, Krzywoustego 4, 44-100 Gliwice, Poland

## Abstract

The dehomologative conversion of linear or α-methyl aldehydes to vinyl boronates is achieved via a one-pot sequence of rhodium-catalyzed transfer dehydroformylation and transfer borylation of the resulting alkenes. Similarly, allylic or aliphatic alcohols are converted to vinyl boronates through a sequence involving, respectively, rhodium-catalyzed isomerization or transfer dehydrogenation to aldehyde intermediates, followed by dehydroformylation–borylation. The vinyl boronates can be further hydrogenated to alkyl boronates using the same rhodium precatalyst, enabling all five catalytic steps with a single catalyst system.

## Graphical abstract

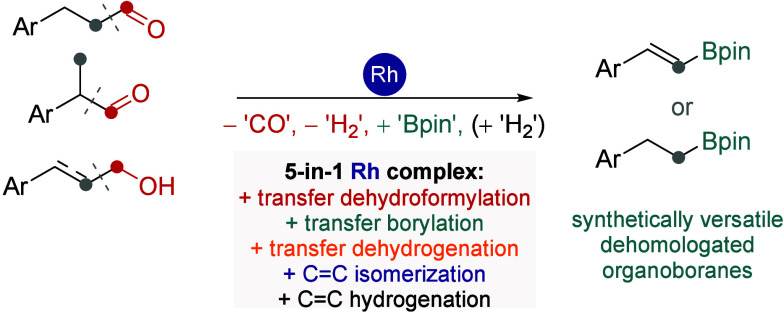

Aldehydes and alcohols, prevalent functional groups in commercial building blocks,^1^ synthetic intermediates,^2^ and natural products,^3^ are essential in the production of bulk and fine chemicals. Developing efficient methods for their synthesis and functionalization continues to be a critical and active area of research.^1,4^ Beyond conventional functional group interconversions and more recent C–H chain functionalization strategies, transformations involving C–C bond cleavage offer a powerful means to access molecules with modified carbon frameworks.^5^ Different activation pathways, such as transition metal-catalyzed C–C bond activation,^6^ oxidative cleavage of C–C or C=C bonds,^7^ and radical-mediated C–C bond scission,^8^ have enabled a range of dehomologative transformations, each having its advantages and limitations (Figure 1a).^9–11^ These methods are valuable in natural product synthesis and the late-stage functionalization of biologically relevant molecules.^12^ We considered that the development of methods for C–C borylation would present a particularly appealing synthetic methodology, given the versatile reactivity and broad utility of organoboron compounds.^13^ Herein, we describe the design and implementation of a convenient one-pot strategy that achieves such transformations. Our approach exploits the unique catalytic capabilities of a single rhodium complex, orchestrating up to five distinct mechanistic processes in a seamless manner (Figure 1b-c).

**Figure 1:**
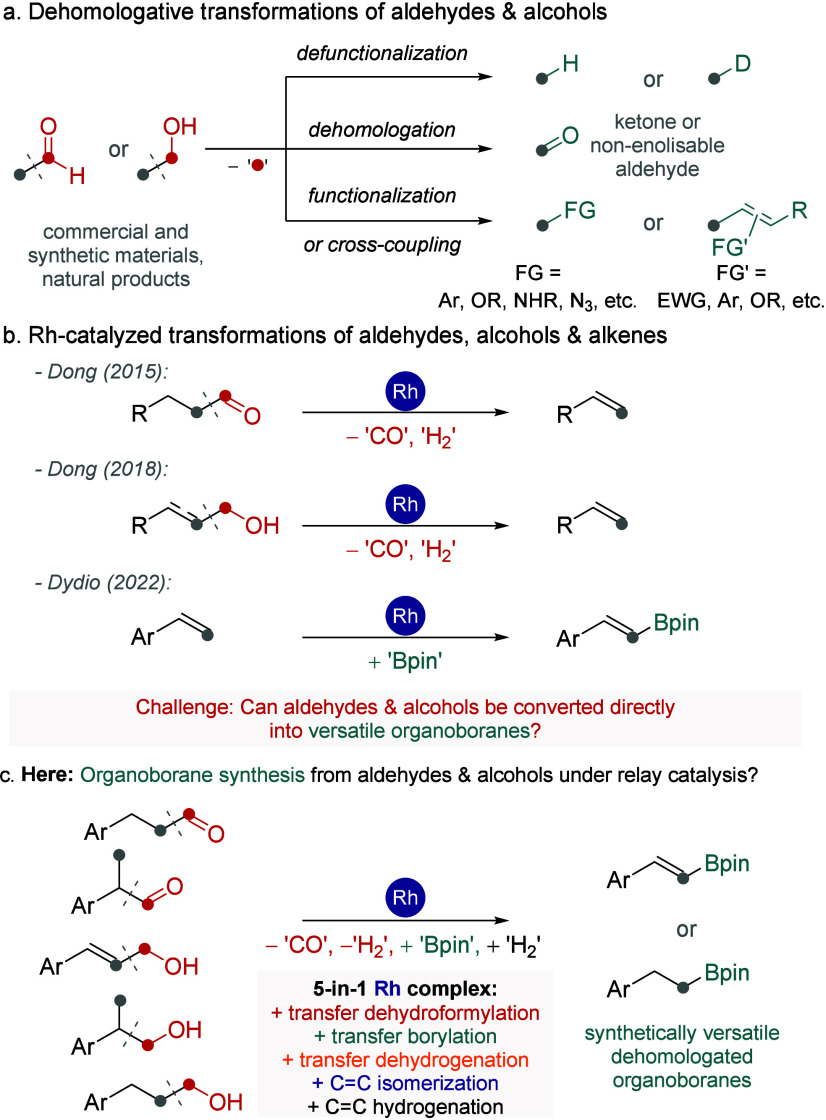
Context of the current work.

At the outset of these studies, we hypothesized that combining rhodium-catalyzed transfer dehydroformylation of aldehydes with the subsequent transfer C–H borylation of the resulting alkenes could enable a direct dehomologative borylation of aldehydes (Scheme 1). Notably, both reactions have been reported to proceed in the presence of Rh­(I)/xantphos catalysts,^14,15^ suggesting that the entire transformation could be carried out using a single rhodium complex. The requirement of a single catalyst minimizes compatibility challenges often encountered in catalytic relay processes involving different catalysts.^16^ We anticipated that achieving the selective formation of the target products over potential side products would hinge on the choice of complementary formyl group/hydride acceptor for the transfer dehydroformylation step and the boryl group donor for the transfer borylation step.

**Scheme 1:**
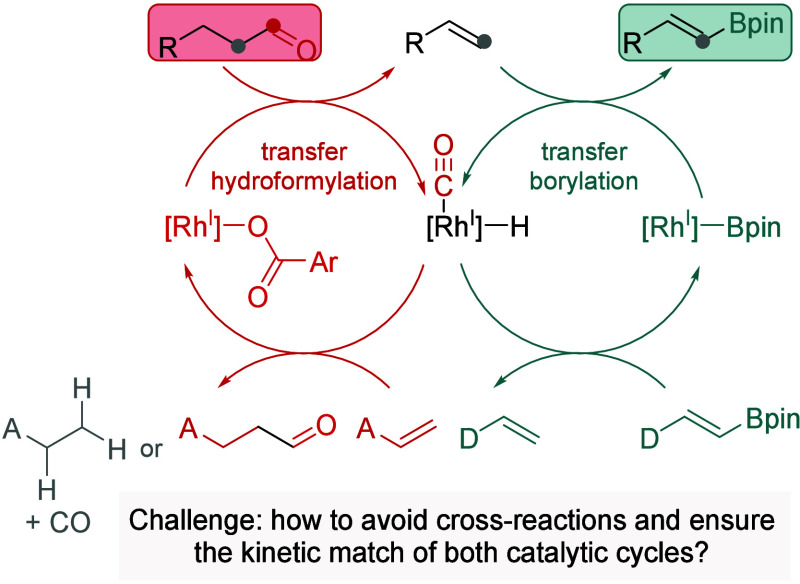
Envisioned C–C bond Borylation of Aldehydes under Rh-Catalyzed Relay

Upon exploration of conditions for hydrocinnamaldehyde (**1**) as a model aldehyde, we found that vinyl boronate **2** was formed in 80% NMR yield (75% isolated yield) when **1** (1 equiv) was reacted with norbornene (**nbe**, 1 equiv) as the acceptor, vinylboronic acid pinacol ester (**vBpin**, 1.25 equiv) as the donor, in the presence of the Rh­(xantphos)­Cl complex (4 mol %), sodium *tert*-butoxide (4 mol %), and 3-methoxybenzoic acid (**mbza**, 4 mol %), in thf at 120 °C (Figure 2a, top). The reaction mixture contained trace amounts of side products, such as ethylbenzene **sp-1** (3%) from competitive hydrogenation, and 14% of alkene intermediate **int-1**. Notably, the control experiments with the sequential reactions of dehydroformylation and borylation executed in a stepwise one-pot fashion—often referred to as a ‘telescoped synthesis’—resulted in inferior yields of **2** due to pronounced side processes. Specifically, when **vBpin** was added after aldehyde was converted into alkene **int-1**, the reactions furnished **2** in 45–65% yield (without or with a fresh rhodium catalyst addition), along with ketone **sp-2** in 18–22% yield, **sp-1** in 11–13% yield, and unreacted alkene **int-1** 4–12% yield (Figure 2a, bottom; Section S9 of the Supporting Information). The formation of **sp-1** and **sp-2** most likely originates, respectively, from the competitive hydrogenation of accumulating **int-1** instead of **nbe**—the hydride acceptor, and Rh-catalyzed hydroacylation of alkene **int-1** with aldehyde **1**.^17^ These side processes are suppressed in the relay catalysis most likely because alkene **int-1** does not accumulate but directly converts into product **2**. This example illustrates an advantage of the all-reagents-in-one-pot (relay) reactions over sequential (telescoped) reactions. Further experiments confirmed the importance of each element of the reaction conditions, as summarized in Table S1 in the Supporting Information. Particularly important proved to be the use of **nbe** as the acceptor. When **nbe** was replaced with, for instance, norbornadiene or dimethylacrylamide, the excellent acceptors in functional group transfer catalysis,^15,18^ the target transformation was not observed (<2% of **2**), due to their inhibiting effect on the transfer borylation step.

**Figure 2:**
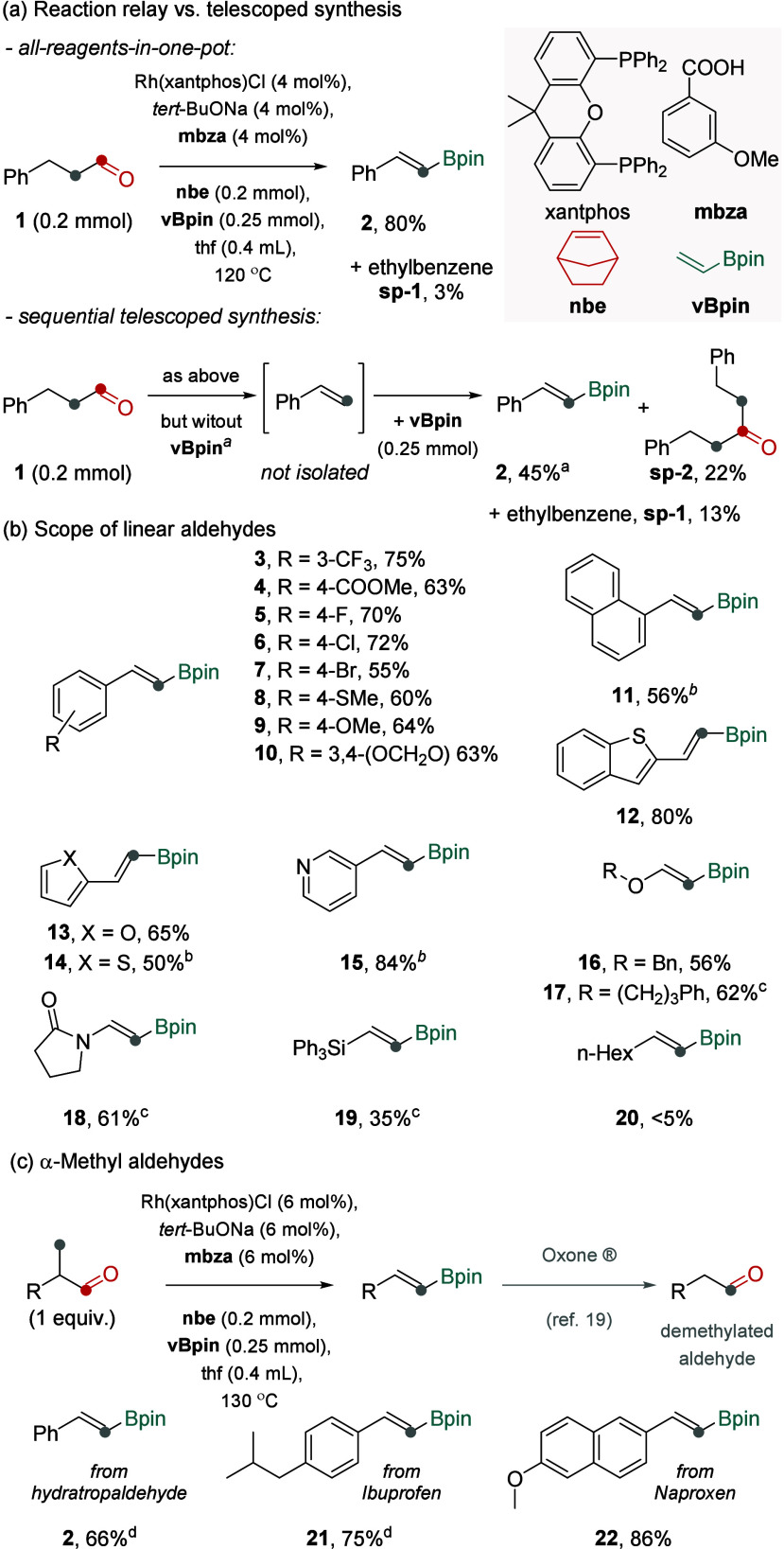
Transforming aldehydes into vinyl boronates via dehydroformylation-borylation sequence carried out under relay catalysis versus sequentially in one pot (a) and the scope of liner and α-methyl aldehydes under relay catalysis (b and c). Because vinylboronic esters tend to overadsorb and partially decompose during chromatography on silica gel,^26^ especially when isolated on small scale, the NMR or GC yields are reported here to indicate the actual reaction performance. For isolated yields, NMR yields of intermediates, and the presence of potential side products, as well as the reactions under varied conditions, see Figure S1. *^a^* Full conversion of the aldehyde was observed after 2 h; **vBpin** was added, and the reaction continued for 8 h (Section S9). *^b^* 6 mol % Rh, at 130 °C. *^c^* After 6 h, the reaction mixture was filtered through a plug of silica, followed by adding 4 mol % Rh and **vBpin** (0.25 mmol), and the reaction continued for 6 h. *^d^* After 2 h, 4 mol % Rh and **vBpin** were added, and the reaction continued for 6 h.

We found that a range of linear aldehydes bearing an β-aryl, heteroaryl, O-ether, N-lactam, or Si group were effective starting materials for the devised transformation (Figure 2b). Specifically, a series of vinyl boronates **2–15** with (hetero)­aryl groups bearing a range of electron-withdrawing or electron-donating functional groups were formed in 50–84% yields under the standard relay conditions. The reaction mixtures typically contained small amounts of alkene intermediates and trace-to-low amounts of hydrogenation side-products (typically <10%). β-O-Benzyl ether **16** was formed in 56% yield along with 22% of the alkene intermediate. In turn, other β-heteroatom-containing vinyl boronates were formed in modest (35–42% for **17–18**) or trace yields (<5% for **19**) under the standard conditions (Figure S1). In these cases, we observed substantial amounts of unreacted alkene intermediates remaining. By addition of an extra portion of **vBpin** and fresh rhodium catalyst, vinyl boronates **17–19** were formed in higher yields (35–62%). Noteworthy, higher yields were achieved when **vBpin** and rhodium catalyst were added in two portions, as opposed to adding them in larger quantities at the start of the reaction. Additionally, we observed that the fresh rhodium complex was prone to rapid deactivation, likely due to the accumulation of inactive rhodium species in the reaction mixture. However, passing the mixture through a short silica plug before introducing the fresh rhodium catalyst significantly mitigated this deactivation. Although this adjustment is less operationally convenient, it enabled more efficient conversion of the remaining alkene intermediates into the target boronate products, resulting in higher yields. The reactions for aldehydes forming isomerizable alkenes upon dehydroformylation, such as *n*-nonanal furnishing 1-octene, resulted in the formation of trace amounts of the target vinyl boronate **20**, illustrating the current limitation.

Because α-methyl aldehydes might form α-alkenes upon transfer dehydroformylation, we reasoned that the dehydroformylation–borylation sequence could also open the path for their formal demethylation (Figure 2c).^19^ Noteworthy, the reported strategies for aldehyde dehomologation convert α-methyl aldehydes into methyl ketones rather than demethylated aldehydes.^10a–10c^

We found that α-methyl aldehydes, including the aldehyde derivatives of Ibuprofen and Naproxen, nonsteroidal anti-inflammatory drugs, reacted to form corresponding organoboronates **2**, **21–22** in 66–86% yields (Figure 2c), demonstrating the capacity of the strategy to modify the carbon framework of bioactive molecules.^20,21^ In some cases, adding extra **vBpin** and a fresh rhodium catalyst enabled the formation of the target products in higher yields.

We next investigated the possibility of extending the strategy toward allylic and aliphatic alcohols by integrating either Rh-catalyzed isomerization of allylic alcohols into aldehydes (Scheme 2a) or Rh-catalyzed hydrogen transfer from aliphatic alcohols to an acceptor to form aldehydes (Scheme 2b), followed by the dehydroformylation–borylation sequence. Encouragingly, Dong previously reported the conversion of allylic and aliphatic alcohols into alkenes under Rh-catalysis with dimethylacrylamide as an acceptor.^18c^ The critical questions were whether these additional processes would also be compatible with the transfer borylation of alkenes and whether the kinetics of three different catalytic cycles could be harmonized toward the productive formation of vinyl boronates.

**Scheme 2:**
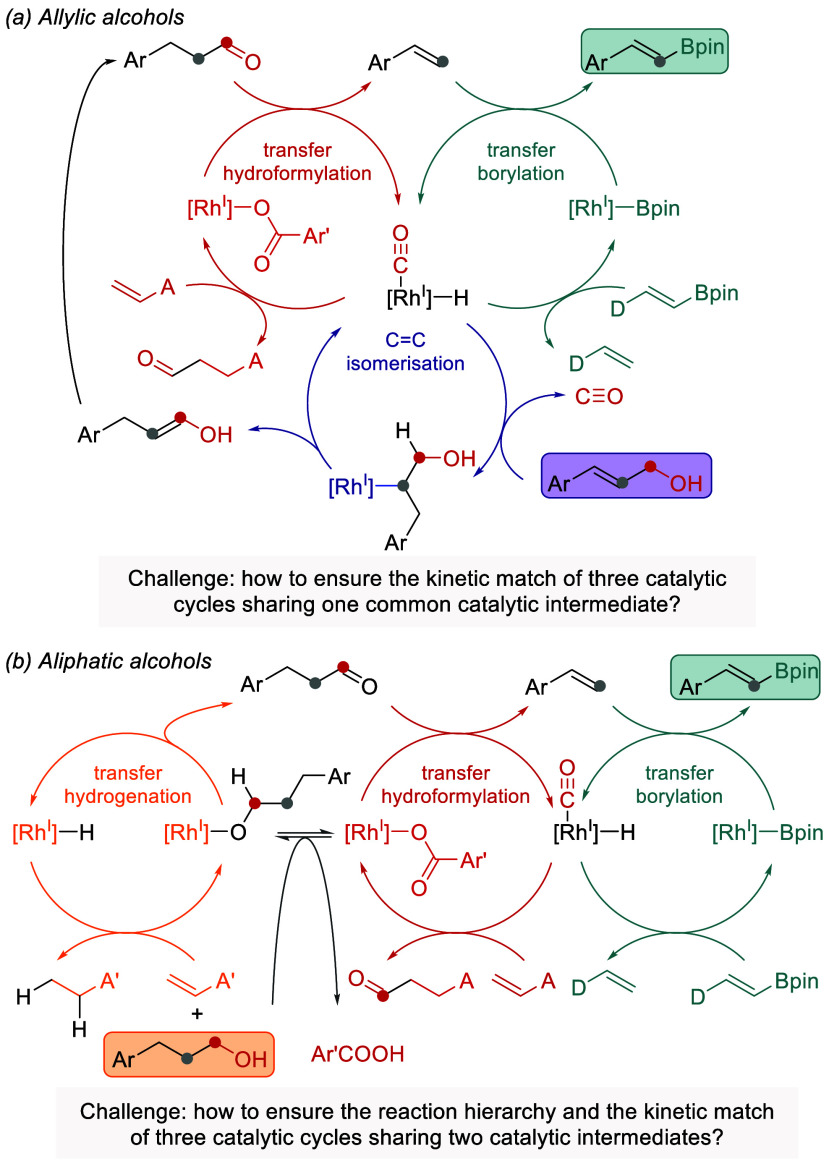
Envisioned C–C Bond Borylation of Allylic and Aliphatic Alcohols under Rh-Catalyzed Relay

We found that adjustments to the reaction conditions enabled the integration of the isomerization activity, furnishing a protocol converting directly allylic alcohols into dehomologated vinyl boronates using a single rhodium catalyst (Figure 3). We observed that a range of allylic alcohols reacted to form target products **2**–**3**, **23**–**31** in 63–87% yields under the developed conditions. In all cases, the reactions using the all-reagents-in-one-pot fashion yielded vinyl boronates as the major products. However, in some cases, an extra portion of the rhodium catalyst and delayed addition of **vBpin** resulted in improved yields of the target products (Figure S2). Again, reactions with allylic alcohols producing isomerisable alkene intermediates, such as hex-2-en-1-ol forming 1-pentene as an intermediate, delivered nearly no product, illustrating the current limitations.

**Figure 3:**
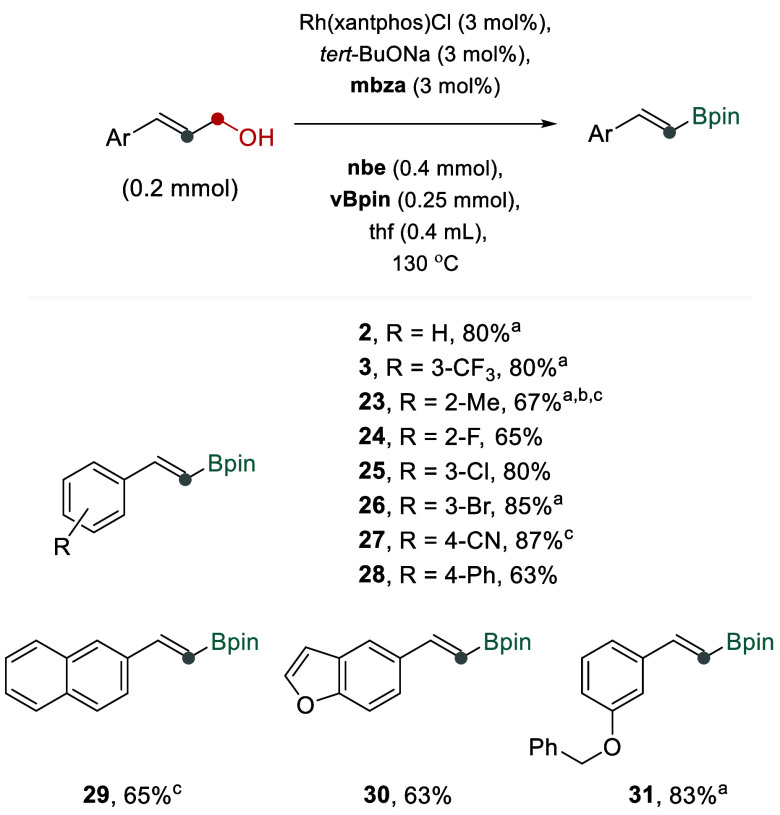
Transforming allylic alcohols into dehomologated vinyl boronates via the isomerization–dehydroformylation–borylation sequence. NMR yields reported; for all data, see Figure S2. ^*a*^The reaction was performed at 120 °C for 2 h, followed by addition of 4 mol % Rh catalyst and **vBpin** (0.25 mmol), and continued for 8 h. ^*b*^The reaction mixture contained a substantial amount of alkene intermediate; the mixture filtered through a plug of silica, followed by adding 4 mol % Rh catalyst and **vBpin** (0.25 mmol), and the reaction continued for 8 h. ^*c*^120 °C.

For reactions involving aliphatic alcohols, we found that **nbe** serves as an effective acceptor for both transfer dehydrogenation and subsequent transfer dehydroformylation of the resulting aldehydes. By optimizing the stoichiometry of the reagents and fine-tuning the reaction conditions, aliphatic alcohols were directly converted into dehomologated or demethylated vinyl boronates **2**–**15**, **22** in 48–88% yields (Figure 4). Noteworthy, one-pot experiments confirmed the successful operation of the designed triple relay. However, the target vinyl boronates were obtained in moderate overall yields (typically 30–40%, Figure S3), with significant amounts of unreacted alkene intermediates and starting alcohols remaining. Efforts to address catalyst deactivation have been largely unsuccessful. Nonetheless, a modified protocol provided vinyl boronates in synthetically useful yields. In this protocol, near-complete conversion of starting materials was achieved by adding **vBpin** and fresh rhodium catalyst in three portions. The final portion was added following filtration of the reaction mixture through a boric-acid-impregnated silica plug.

**Figure 4:**
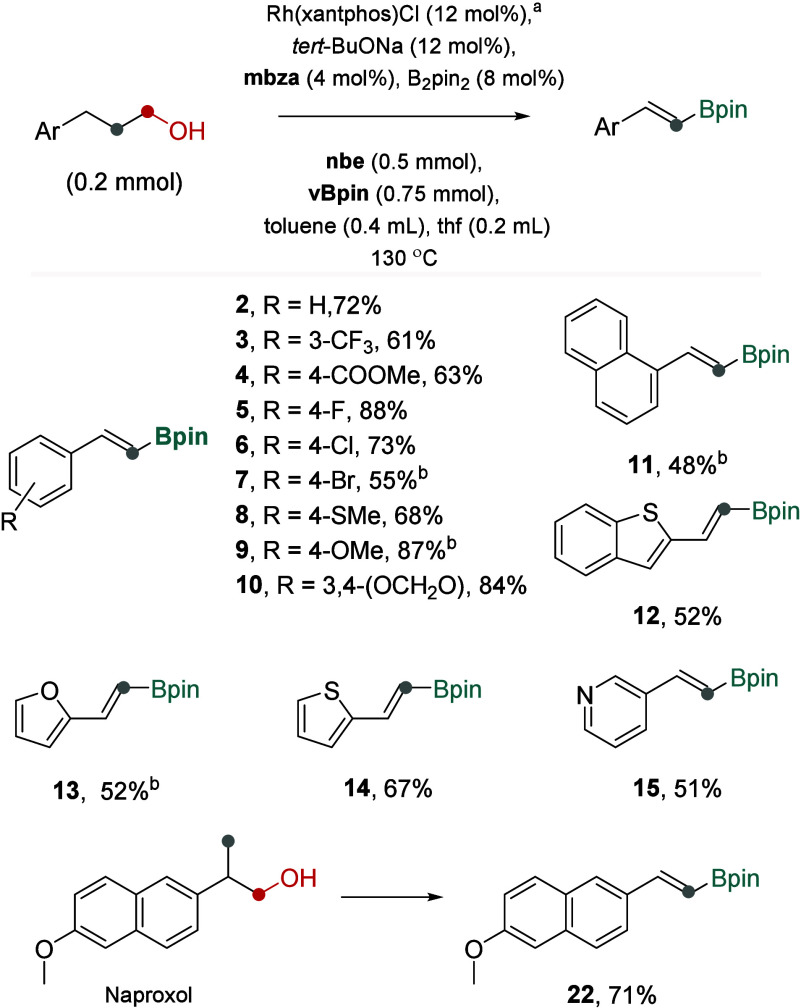
Transforming linear and β-methyl aliphatic alcohols into dehomologated or demethylated vinyl boronates via dehydrogenation-dehydroformylation-borylation. NMR yields reported; for all details, see Figure S3. ^*a*^Reagents added in three portions; 2nd and 3rd portions added after 2 and 9 h, respectively; 3rd portion added after the mixture filtered through a plug of silica, and the reactions continued for 7 h. ^*b*^ **vBpin** added only after 2 and 9 h.

Notably, these protocols are readily scalable (Figure 5). Amounts >1 g of vinyl boronates **2**, **9**, and **31** were prepared from aldehyde, aliphatic alcohol and allylic alcohol respectively, corresponding to 72–80% isolated yields. Interestingly, the gram-scale reaction for the aliphatic alcohol formed the target product **9** in high yield without the need for filtration of the reaction mixture and an extra portion of Rh, thereby simplifying the reaction setup and improving the efficiency.

**Figure 5:**
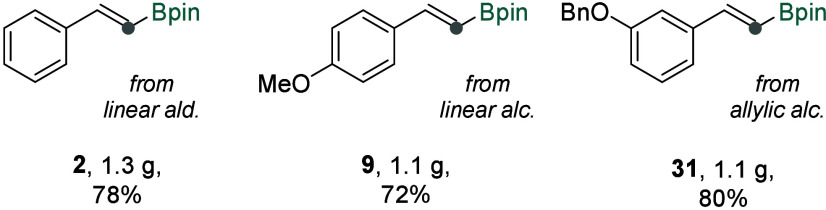
Gram scale experiments. Isolated amounts and yields were reported.

Lastly, considering the reactivity of rhodium complexes in the hydrogenation of vinyl boronates,^22^ we investigated the potential to convert vinyl derivatives into alkyl boronate products. By exchanging the inert atmosphere for dihydrogen (1–5 bar) and stirring the reaction mixture at 60 °C for 16 h after the initial conversion of starting aldehydes or alcohols into vinyl boronates, we achieved the formation of alkyl boronates (Figure 6). For example, hydrocinnamaldehyde was successfully transformed into alkyl boronate **32** in 50% yield. Similarly, α-methyl aldehyde, allylic alcohol, linear alcohol, and β-methyl alcohol were converted to alkyl boronates **33–36** in 57–71% yields, demonstrating the general compatibility of the dehomologative borylation process with the subsequent hydrogenation step. Notably, the transformations of alcohols into alkyl boronates **34**–**36** involved four mechanistically distinct consecutive catalytic processes, all executed by the same rhodium complex.

**Figure 6:**
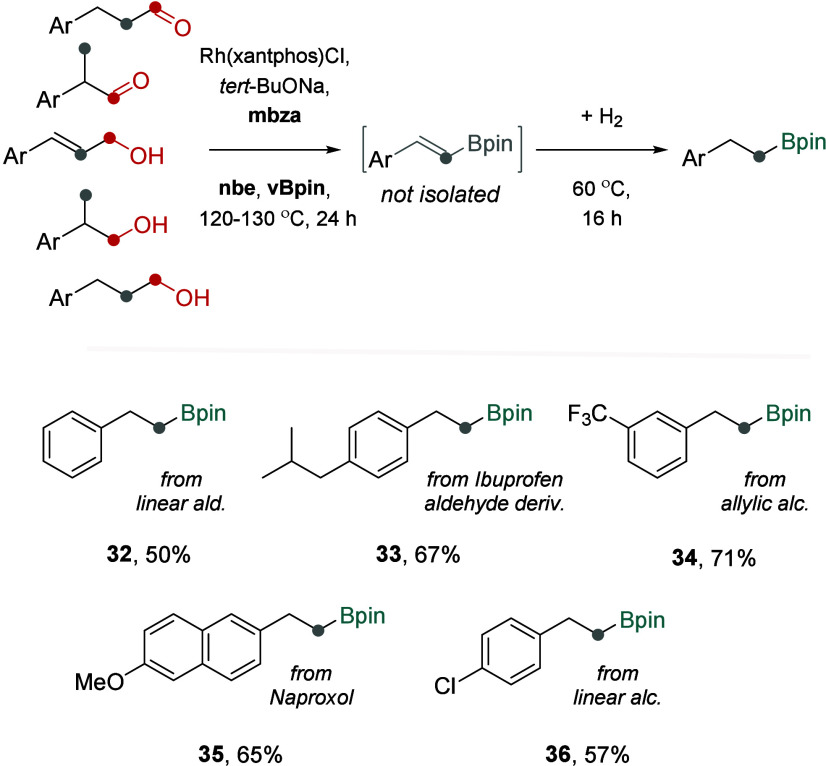
Transforming aldehydes and alcohols into alkyl boronates incorporating telescoped Rh-catalyzed hydrogenation of vinyl boronate intermediates. Isolated yields reported (no analytical yields available). (i) Aldehyde/alcohol (0.2 mmol) converted into a vinyl boronate under conditions shown in Figures 2–4, (ii) + thf (0.4 mL), H_2_ (1–5 bar), 60 °C, 16 h.

Overall, this work introduces a novel class of C–C bond functionalization methods targeting common functional groups—aldehydes and alcohols—to generate organoboronates as versatile linchpins for one-carbon-removing transformations. The approach leverages the unique reactivity of rhodium complexes, enabling multiple catalytic processes to occur in a sequence. These protocols for dehomologative borylations complement existing methods for homologative^23^ and carbon framework-preserving^24^ borylation, providing straightforward access to homologous product series from the same substrates.^25^ More broadly, this study highlights the potential of multicatalysis to integrate multiple catalytic steps into efficient, complex transformations, streamlining the synthesis of fine chemicals.

## Supplementary Material



## Notes

Details on experimental procedures, including the detailed catalytic protocols, the optimization and control experiments, analytical data, and copies of the NMR spectra (PDF)
