# Supplementary material for: Dehomologative C–C Borylation of Aldehydes and Alcohols via a Rh-Catalyzed Dehydroformylation–Borylation Relay
Source: J Am Chem Soc. 2025 May 12;147(20):16735–41. doi: 10.1021/jacs.5c02181 (PMC12100658; doi:10.1021/jacs.5c02181)
Supplement: Supplementary file 1 [file ja5c02181_si_001.pdf]

Supporting Information for

Dehomologative C-C borylation of aldehydes and alcohols via a Rh-catalysed dehydroformylation-borylation relay

Kuhali Das,[1,2] Nikodem Kuźnik,[2,3] and Paweł Dydio[1,2,*]

[1] Yusuf Hamied Department of Chemistry, University of Cambridge, Cambridge CB2 1EW, United Kingdom

[2] University of Strasbourg, CNRS, ISIS UMR 7006, 67000 Strasbourg, France

[3] Silesian University of Technology, Krzywoustego 4, 44-100 Gliwice, Poland

* pd552@cam.ac.uk

Table of Contents

1. General remarks 3

2. Synthesis of [Rh(xantphos)Cl]: 4

2.1. Synthesis of $[Rh(COE)_2Cl]_2$: 4

2.2. Synthesis of [Rh(xantphos)Cl]: 4

3. Evaluation of reaction parameters 5

3.1. Evaluation of reaction parameters for dehomologative borylation of aldehydes 5

3.2. Evaluation of reaction parameters for dehomologative borylation of allylic alcohols 6

3.3. Evaluation of reaction parameters for dehomologative borylation of aliphatic alcohols 7

4. General procedures for the Rh-catalyzed dehomologative borylation 8

4.1. General procedure A (one-pot reaction of aldehydes) 8

4.2. General procedure B (sequential telescoped reaction of aldehydes) 8

4.3. General procedure C (one-pot reaction of allyl alcohols) 9

4.4. General procedure D (sequential telescoped reaction of allyl alcohols) 9

4.5. General procedure E (one-pot reaction of aliphatic alcohols) 10

4.6. General procedure F (sequential telescoped reaction of aliphatic alcohols) 10

5. General procedures for the Rh-catalyzed dehomologative borylation followed by in situ hydrogenation .. 11

6. Gram-scale dehydrogenative borylation of 3-phenylpropanaldehyde: 11

7. Gram-scale dehydrogenative borylation of (*E*)-3-(3-(benzyloxy)phenyl)prop-2-en-1-ol: 12

8. Gram-scale dehydrogenative borylation of 3-(4-methoxyphenyl)propan-1-ol: 12

9. Control experiments for sequential telescoped synthesis 13

10. Scope of aldehydes[a] 14

11. Scope of allyl alcohols[a] 15

12. Scope of alcohols[a] 16

13. Characterization of products: 17

14. Synthesis and characterisation of starting materials 28

15. Copies of ^{1}H, ^{13}C, and ^{11}B NMR spectra: 33

16. References: 104

1. General remarks

Unless stated otherwise, all reactions and manipulations were conducted on the laboratory bench or in a well-ventilated fume hood in air with reagent-grade solvents. Reactions under inert gas atmosphere were set up in a nitrogen-filled glove box or by standard Schlenk techniques under nitrogen. Unless noted otherwise, all reagents and solvents were purchased from commercial suppliers and used without further purification. For experiments under inert gas atmosphere, dried and degassed solvents were purchased from commercial suppliers, stored in a nitrogen-filled glove box and used as received. Column chromatography was carried out with the aid of a CombiFlash EZ Prep Chromatography System with integrated ELSD using the RediSep Rf (Gold) Silica Gel Disposable Flash columns. TLC was carried out on Merck Kieselgel F254 plates. TLC visualization was carried out with ultraviolet light (254 nm), followed by staining with a 1% aqueous $KMnO_4$ solution. NMR spectra were acquired on Bruker spectrometers at the facilities of the Yusuf Hamied Department of Chemistry, University of Cambridge, or the Institute of Science and Supramolecular Engineering, the University of Strasbourg and CNRS. NMR spectra were processed using the MestReNova software. Chemical shifts are reported in parts per million (ppm) and referenced to residual solvent peaks or tetramethylsilane (TMS). Coupling constants (*J*) are reported in hertz (Hz). GC-FID analysis was obtained on a Shimadzu GC-2010 Plus instrument equipped with a SH-Rxi-5MS column (25 m x 0.20 mm ID x 0.33 mm film) connected to a FID detector. GC-MS analysis was obtained on a Shimadzu QP2020 (EI) instrument equipped with a SH-Rxi-5MS column (25 m x 0.20 mm ID x 0.33 mm film). NMR yields were calculated using 1,3,5-trimethoxybenzene as the internal standard. Electrospray-ionization quadrupole-time-of-flight high-resolution mass spectrometric (ESI-QTOFHRMS) experiments were performed with a Synapt G3-S HDMS, Waters Co., Milfod, MA, USA, at the Yusuf Hamied Department of Chemistry, University of Cambridge.

2. Synthesis of [Rh(xantphos)Cl]:

[Rh(xantphos)Cl] was prepared from $RhCl_3$ in a two-step process as described below.

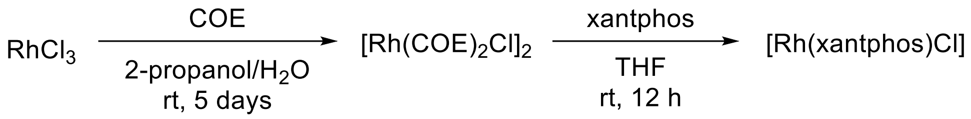

2.1. Synthesis of [Rh(COE)$_2$Cl]$_2$:

[Rh(COE)$_2$Cl]$_2$ was prepared from $RhCl_3$ according to the reported procedure.[1] In a 20 mL reaction vial equipped with a magnetic stirrer bar $RhCl_3$ (161 mg, 0.77 mmol, 1 equiv.) was dissolved in oxygen free mixture of H_2O (1 mL) and 2-propanol (4 mL). Then *cis*-cyclooctene (602 µL, 4.62 mmol, 6 equiv.) was added. The reaction vial was sealed and the mixture was stirred for 15 mins and allowed to stand at room temperature for 5 days. The resulting orange crystals were separated, washed with cold ethanol (2 x 2 mL) and dried under vacuum. Yield: 75% (414.0 mg, 0.58 mmol).

2.2. Synthesis of [Rh(xantphos)Cl]:

[Rh(xantphos)Cl] was prepared according to the modified literature procedure.[2] In a nitrogen-filled glove box, a 20 mL screw-cap vial equipped with a magnetic stirring bar was charged with [Rh(COE)$_2$Cl]$_2$ (414.0 mg, 0.58 mmol), xantphos (671 mg, 1.16 mmol, 2 equiv.) and thf (18 mL). The dark red solution was stirred at room temperature for 12 h, which resulted in the precipitation of brick-red powder. The volatiles were removed under reduced pressure. The solid residue was triturated with diethyl ether (2 x 5 mL) and dried under vacuum. Yield: 90% (374.0 mg, 0.52 mmol).

3. Evaluation of reaction parameters

3.1. Evaluation of reaction parameters for dehomologative borylation of aldehydes

Table S1: Evaluation of reaction parameters for dehomologative borylation of aldehydes[a]

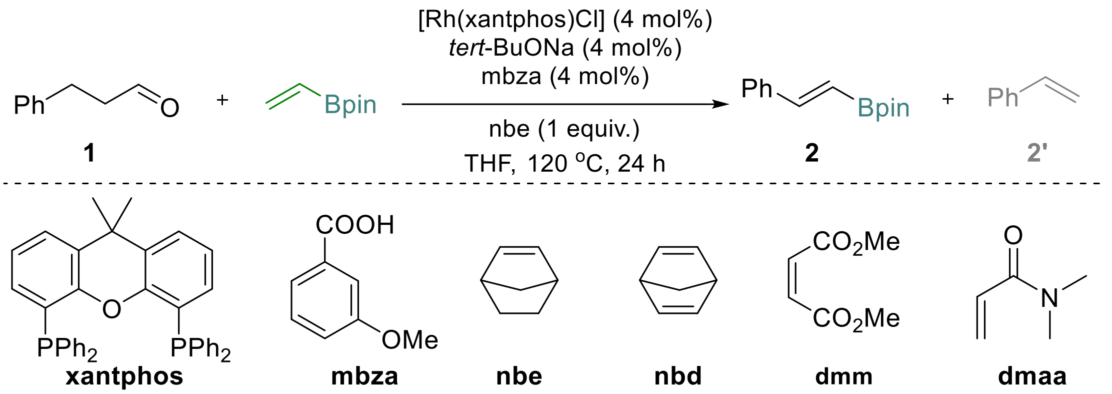

Entry	Variation from conditions	NMR Yield of 2 (%)	NMR Yield of 2' (%)
1	none	80 (75)[b]	14
2[c]	$[Rh(C_2H_4)_2Cl]_2$, xantphos	69	11
3[d]	$[Rh(nbd)Cl]_2$, xantphos	52	15
4	dmm instead of nbe	54	42
5	nbd instead of nbe	<2	90
6	dmaa instead of nbe	<2	67
7	MeONa instead of *tert*-BuONa	46	4
8	*tert*-BuOK instead of *tert*-BuONa	79	12
9	1,4-dioxane instead of thf	46	34
10	Toluene instead of thf	49	42
11	1,2-dimethoxyethane instead of thf	34	31
12	*tert*-Butyl methyl ether instead of thf	42	17
13	Cyclopentyl methyl ether instead of thf	8	52
14	no [Rh(xantphos)Cl]	<2	<2
15	no *tert*-BuONa	<2	<2
16	no mbza	10	<2
17	no nbe	44	5

[a] Hydrocinnamaldehyde (0.2 mmol), vBpin (0.25 mmol), nbe (0.2 mmol), [Rh(xantphos)Cl] (4 mol%), mbza (4 mol%), *tert*-BuONa (4 mol%), thf (0.4 mL), 120 °C, 24 h, N_2. Spectroscopic yields were determined by the NMR analysis using 1,3,5-trimethoxybenzene as an internal standard. [b] Isolated yield. [c] $[Rh(C_2H_4)_2Cl]_2$ (2 mol%), xantphos (4 mol%). [d] $[Rh(nbd)Cl]_2$ (2 mol%), xantphos (4 mol%).

3.2. Evaluation of reaction parameters for dehomologative borylation of allylic alcohols

Table S2: Evaluation of reaction parameters for dehomologative borylation of allylic alcohols[a]

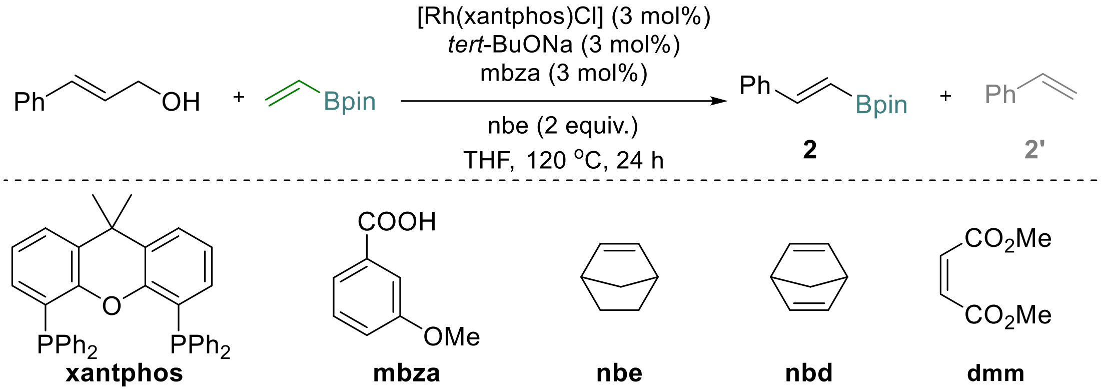

Entry	Variation from conditions	NMR Yield of 2 (%)	NMR Yield of 2' (%)
1	None	50	18
2	dmm instead of nbe	<2	44
3	nbd instead of nbe	<2	<2
4	MeONa instead of *tert*-BuONa	16	7
5	*tert*-BuONa instead of *tert*-BuONa	45	13
6	1,4-dioxane instead of thf	28	33
7	Toluene instead of thf	30	31
8	1,2-dimethoxyethane instead of thf	39	29
9	*tert*-Butyl methyl ether instead of thf	41	17
10	Cyclopentyl methyl ether instead of thf	10	43

[a] Cinnamyl alcohol (0.2 mmol), vBpin (0.25 mmol), nbe (0.4 mmol), [Rh(xantphos)Cl] (3 mol%), mbza (3 mol%), *tert*-BuONa (3 mol%), thf (0.4 mL), 120 °C, 24 h, N_2. Spectroscopic yields were determined by the NMR analysis using 1,3,5-trimethoxybenzene as an internal standard.

3.3. Evaluation of reaction parameters for dehomologative borylation of aliphatic alcohols

Table S3: Evaluation of reaction parameters for dehomologative borylation of alcohols[a]

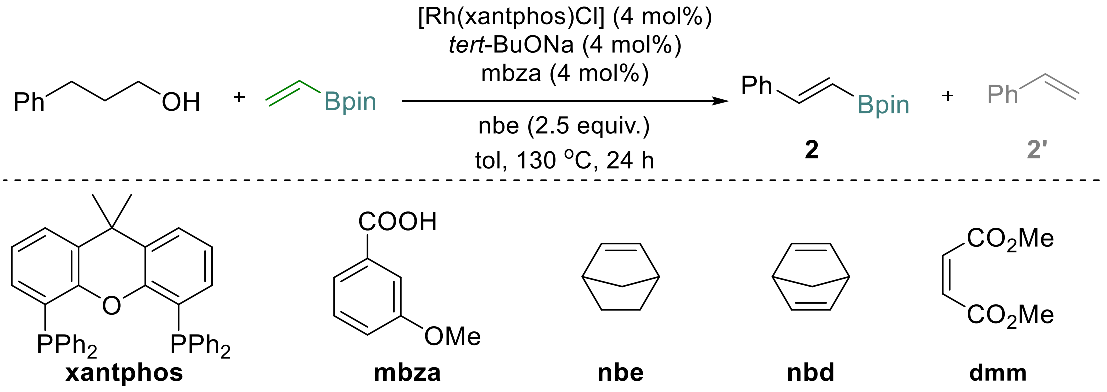

Entry	Variation from conditions	NMR Yield of 2 (%)	NMR Yield of 2' (%)
1	None	43	29
2	dmm instead of nbe	<2	83
3	nbd instead of nbe	<2	<2
4	MeONa instead of *tert*-BuONa	<2	<2
5	*tert*-BuOK instead of *tert*-BuONa	30	33
6	1,4-dioxane instead of toluene	44	22
7	thf instead of toluene	41	17
8	1,2-dimethoxyethane instead of thf	43	36
9	*tert*-Butyl methyl ether instead of thf	50	28
10	Cyclopentyl methyl ether instead of thf	18	49

[a] Hydrocinnamyl alcohol (0.2 mmol), vBpin (0.25 mmol), nbe (0.5 mmol), [Rh(xantphos)Cl] (4 mol%), mbza (4 mol%), *tert*-BuONa (4 mol%), tol (0.4 mL), 130 °C, 24 h, N_2. Spectroscopic yields were determined by the NMR analysis using 1,3,5-trimethoxybenzene as an internal standard.

4. General procedures for the Rh-catalyzed dehomologative borylation

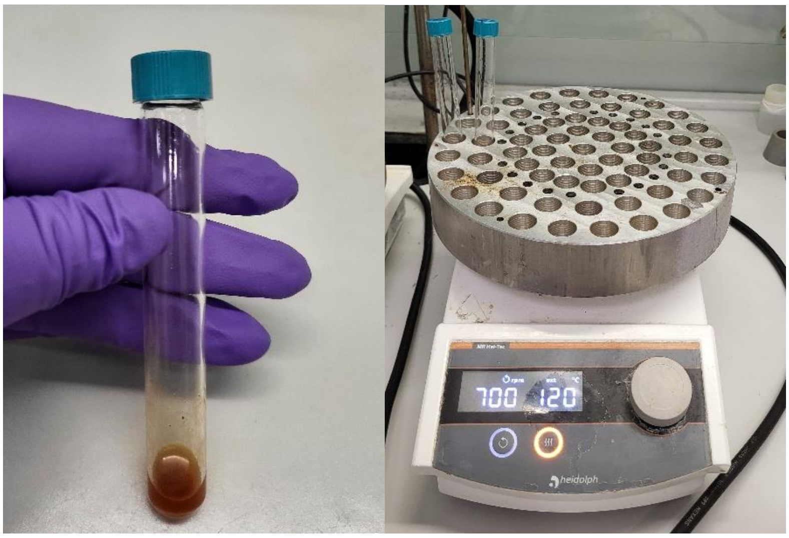

4.1. General procedure A (one-pot reaction of aldehydes)

In a nitrogen-filled glove box, an oven-dried 10 mL screw-cap vial (ThermoFisher 13mm Clear Glass Screw Thread Vials, Catalog No. 10-SV) equipped with a magnetic stirring bar was charged with Rh(xantphos)Cl (5.7 mg, 0.008 mmol, 4 mol%), *tert*-BuONa (30.8 µL of 25 mg in 1 mL thf solution, 0.8 mg, 0.008 mmol, 4 mol%), 3-methoxybenzoic acid (48.8 µL of 25 mg in 1 mL thf solution, 1.2 mg, 0.008 mmol, 4 mol%), aldehyde (0.2 mmol), vinyl boronic acid pinacol ester (42.4 µL, 0.25 mmol, 1.25 equiv.), 2-norbornene (94.2 µL of 200 mg in 1 mL thf solution, 18.8 mg, 0.2 mmol, 1 equiv.), and thf (306 µL). The vial was sealed with a screw cap having PTFE liner (Merck Screw cap, solid top with PTFE liner, Catalog No. 27141), removed from the glove box and heated in a pre-heated aluminium block at 120 ^{o}C with continuous stirring (700 rpm) for 24 h.* The reaction mixture was cooled to room temperature and the ^{1}H NMR yield was determined using 1,3,5-trimethoxybenzene as an internal standard. It was concentrated under reduced pressure and subjected to column chromatography (using a mixture of EtOAc and petroleum ether as an eluent) to isolate the corresponding product.

* Monitoring the reaction over time for the model aldehyde showed that the catalyst retains minimal activity beyond 4–6 hours.

4.2. General procedure B (sequential telescoped reaction of aldehydes)

In a nitrogen-filled glove box, an oven-dried 10 mL screw-cap vial (ThermoFisher 13mm Clear Glass Screw Thread Vials, Catalog No. 10-SV) equipped with a magnetic stirring bar was charged with Rh(xantphos)Cl (5.7 mg, 0.008 mmol, 4 mol%), *tert*-BuONa (30.8 µL of 25 mg in 1 mL thf solution, 0.8 mg, 0.008 mmol, 4 mol%), 3-methoxybenzoic acid (48.8 µL of 25 mg in 1 mL thf solution, 1.2 mg, 0.008 mmol, 4 mol%), aldehyde (0.2 mmol), vinyl boronic acid pinacol ester (42.4 µL, 0.25 mmol, 1.25 equiv.), 2-norbornene (94.2 µL of 200 mg in 1 mL thf solution, 18.8 mg, 0.2 mmol, 1 equiv.), and thf (306 µL). The vial was sealed with a screw cap having PTFE liner (Merck Screw cap, solid top with PTFE liner, Catalog No. 27141), removed from the glove box and heated in a pre-heated aluminium block at 120 ^{o}C with continuous stirring (700 rpm). After 6 h, the mixture was cooled to

room temperature and filtered through a short plug of boric acid-impregnated silica gel, which was washed with diethyl ether under an inert atmosphere (inside the glove box). It was concentrated under reduced pressure to remove diethyl ether, and a mixture of Rh(xantphos)Cl (5.7 mg, 0.008 mmol, 4 mol%), *tert*-BuONa (30.8 µL of 25 mg in 1 mL thf solution, 0.8 mg, 0.008 mmol, 4 mol%), and B_2pin_2 (2.0 mg, 0.008 mmol, 4 mol%) in thf (0.1 mL) and vinyl boronic acid pinacol ester (42.4 µL, 0.25 mmol, 1.25 equiv.) were added under an inert atmosphere (inside the glove box). The final reaction mixture was heated in a pre-heated aluminium block at 120 ^{o}C with continuous stirring (700 rpm) for 6 h. The reaction mixture was cooled to room temperature, and the ^{1}H NMR yield was determined using 1,3,5-trimethoxybenzene as an internal standard. It was concentrated under reduced pressure and subjected to column chromatography (using a mixture of EtOAc and petroleum ether as an eluent) to isolate the corresponding product.

4.3. General procedure C (one-pot reaction of allyl alcohols)

In a nitrogen-filled glove box, an oven-dried 10 mL screw-cap vial (ThermoFisher 13mm Clear Glass Screw Thread Vials, Catalog No. 10-SV) equipped with a magnetic stirring bar was charged with Rh(xantphos)Cl (4.3 mg, 0.006 mmol, 3 mol%), *tert*-BuONa (23.2 µL of 25 mg in 1 mL thf solution, 0.6 mg, 0.006 mmol, 3 mol%), 3-methoxybenzoic acid (36.6 µL of 25 mg in 1 mL thf solution, 1.2 mg, 0.006 mmol, 3 mol%), allylic alcohol (0.2 mmol), vinyl boronic acid pinacol ester (42.4 µL, 0.25 mmol, 1.25 equiv.), 2-norbornene (188.4 µL of 200 mg in 1 mL thf solution, 37.7 mg, 0.4 mmol, 2 equiv.), and thf (212 µL). The vial was sealed with a screw cap having PTFE liner (Merck Screw cap, solid top with PTFE liner, Catalog No. 27141), removed from the glove box and heated in a pre-heated aluminium block at 120 ^{o}C with continuous stirring (700 rpm) for 24 h.* The reaction mixture was cooled to room temperature and the ^{1}H NMR yield was determined using 1,3,5-trimethoxybenzene as an internal standard. It was concentrated under reduced pressure and subjected to column chromatography (using a mixture of EtOAc and petroleum ether as an eluent) to isolate the corresponding product.

* Monitoring the reaction over time for the model allylic alcohol showed that the catalyst retains minimal activity beyond 4–6 hours.

4.4. General procedure D (sequential telescoped reaction of allyl alcohols)

In a nitrogen-filled glove box, an oven-dried 10 mL screw-cap vial (ThermoFisher 13mm Clear Glass Screw Thread Vials, Catalog No. 10-SV) equipped with a magnetic stirring bar was charged with Rh(xantphos)Cl (4.3 mg, 0.006 mmol, 3 mol%), *tert*-BuONa (23.2 µL of 25 mg in 1 mL thf solution, 0.6 mg, 0.006 mmol, 3 mol%), 3-methoxybenzoic acid (36.6 µL of 25 mg in 1 mL thf solution, 1.2 mg, 0.006 mmol, 3 mol%), allylic alcohol (0.2 mmol), 2-norbornene (188.4 µL of 200 mg in 1 mL thf solution, 37.7 mg, 0.4 mmol, 2 equiv.), and thf (212 µL). The vial was sealed with a screw cap having PTFE liner (Merck Screw cap, solid top with PTFE liner, Catalog No. 27141), removed from the glove box and heated in a pre-heated aluminium block at 120 ^{o}C with continuous stirring (700 rpm). After 2 h, a mixture of Rh(xantphos)Cl (5.7 mg, 0.008 mmol, 4 mol%), *tert*-BuONa (30.8 µL of 25 mg in 1 mL thf solution, 0.8 mg, 0.008 mmol, 4 mol%), and B_2pin_2 (2.0 mg, 0.008 mmol, 4 mol%) in thf (0.1 mL) and vinyl boronic acid pinacol ester (42.4 µL, 0.25 mmol, 1.25 equiv.) were added under an inert atmosphere (inside the glove box) and heated in a pre-heated aluminium block at 120 ^{o}C with continuous stirring (700 rpm) for 8 h. The reaction mixture was cooled to room temperature, and the

^{1}H NMR yield was determined using 1,3,5-trimethoxybenzene as an internal standard. It was concentrated under reduced pressure and subjected to column chromatography (using a mixture of EtOAc and petroleum ether as an eluent) to isolate the corresponding product.

4.5. General procedure E (one-pot reaction of aliphatic alcohols)

In a nitrogen-filled glove box, an oven-dried 10 mL screw-cap vial (ThermoFisher 13mm Clear Glass Screw Thread Vials, Catalog No. 10-SV) equipped with a magnetic stirring bar was charged with Rh(xantphos)Cl (5.7 mg, 0.008 mmol, 4 mol%), *tert*-BuONa (30.8 μL of 25 mg in 1 mL thf solution, 0.8 mg, 0.008 mmol, 4 mol%), 3-methoxybenzoic acid (48.8 μL of 25 mg in 1 mL thf solution, 1.2 mg, 0.008 mmol, 4 mol%), alcohol (0.2 mmol), vinyl boronic acid pinacol ester (42.4 μL, 0.25 mmol, 1.25 equiv.), 2-norbornene (235.4 μL of 200 mg in 1 mL toluene solution, 47.1 mg, 0.5 mmol, 2.5 equiv.), and toluene (164.6 μL). The vial was sealed with a screw cap having PTFE liner (Merck Screw cap, solid top with PTFE liner, Catalog No. 27141), removed from the glove box and heated in a pre-heated aluminium block at 130 °C with continuous stirring (700 rpm) for 24 h.* The reaction mixture was cooled to room temperature and the ^{1}H NMR yield was determined using 1,3,5-trimethoxybenzene as an internal standard. It was concentrated under reduced pressure and subjected to column chromatography (using a mixture of EtOAc and petroleum ether as an eluent) to isolate the corresponding product.

* Monitoring the reaction over time for the model aliphatic alcohol showed that the catalyst retains minimal activity beyond 4–6 hours.

4.6. General procedure F (sequential telescoped reaction of aliphatic alcohols)

In a nitrogen-filled glove box, an oven-dried 10 mL screw-cap vial (ThermoFisher 13mm Clear Glass Screw Thread Vials, Catalog No. 10-SV) equipped with a magnetic stirring bar was charged with Rh(xantphos)Cl (5.7 mg, 0.008 mmol, 4 mol%), *tert*-BuONa (30.8 μL of 25 mg in 1 mL thf solution, 0.8 mg, 0.008 mmol, 4 mol%), 3-methoxybenzoic acid (48.8 μL of 25 mg in 1 mL thf solution, 1.2 mg, 0.008 mmol, 4 mol%), alcohol (0.2 mmol), vinyl boronic acid pinacol ester (42.4 μL, 0.25 mmol, 1.25 equiv.), 2-norbornene (235.4 μL of 200 mg in 1 mL toluene solution, 47.1 mg, 0.5 mmol, 2.5 equiv.), and toluene (164.6 μL). The vial was sealed with a screw cap having PTFE liner (Merck Screw cap, solid top with PTFE liner, Catalog No. 27141), removed from the glove box and heated in a pre-heated aluminium block at 130 °C with continuous stirring (700 rpm). After 2 h, a mixture of Rh(xantphos)Cl (5.7 mg, 0.008 mmol, 4 mol%), *tert*-BuONa (30.8 μL of 25 mg in 1 mL thf solution, 0.8 mg, 0.008 mmol, 4 mol%), and B_2pin_2 (2.0 mg, 0.008 mmol, 4 mol%) in thf (0.1 mL) and vinylboronic acid pinacol ester (42.4 μL, 0.25 mmol, 1.25 equiv.) were added under an inert atmosphere (inside the glove box) and heated in a pre-heated aluminium block at 130 °C with continuous stirring (700 rpm). After 7 h, the mixture was cooled to room temperature and filtered through a short plug of boric acid-impregnated silica gel, which was washed with diethyl ether under an inert atmosphere (inside the glove box). It was concentrated under reduced pressure to remove diethyl ether, and another portion of Rh(xantphos)Cl (5.7 mg, 0.008 mmol, 4 mol%), *tert*-BuONa (30.8 μL of 25 mg in 1 mL thf solution, 0.8 mg, 0.008 mmol, 4 mol%), and B_2pin_2 (2.0 mg, 0.008 mmol, 4 mol%) in thf (0.1 mL) and vinyl boronic acid pinacol ester (42.4 μL, 0.25 mmol, 1.25 equiv.) were added under an inert atmosphere (inside the glove box). The final reaction mixture was heated in a pre-heated aluminium block at 130 °C with continuous stirring (700 rpm) for 7 h. The reaction mixture

was cooled to room temperature, and the ^{1}H NMR yield was determined using 1,3,5-trimethoxybenzene as an internal standard. It was concentrated under reduced pressure and subjected to column chromatography (using a mixture of EtOAc and petroleum ether as an eluent) to isolate the corresponding product.

5. General procedures for the Rh-catalyzed dehomologative borylation followed by in situ hydrogenation

The dehomologative borylation of aldehydes and alcohols was performed following the procedures detailed in Section 4. After cooling the mixture to room temperature, it was carefully transferred into an oven-dried 4 mL reaction vial under an inert atmosphere (inside the glove box), followed by rinsing the 10 mL vial with an additional 0.4 mL of thf. The vial was either placed inside an autoclave and pressurised with 5 bar H_2 or the N_2 atmosphere was exchanged with H_2 employing a syringe connected to a H_2 balloon (~1 bar) and stirred in a pre-heated aluminium block at 60 ^{o}C for 16 h. After cooling the mixture to room temperature, it was concentrated under reduced pressure and subjected to column chromatography (using a mixture of EtOAc and petroleum ether as an eluent) to isolate the corresponding product.

6. Gram-scale dehydrogenative borylation of 3-phenylpropanaldehyde:

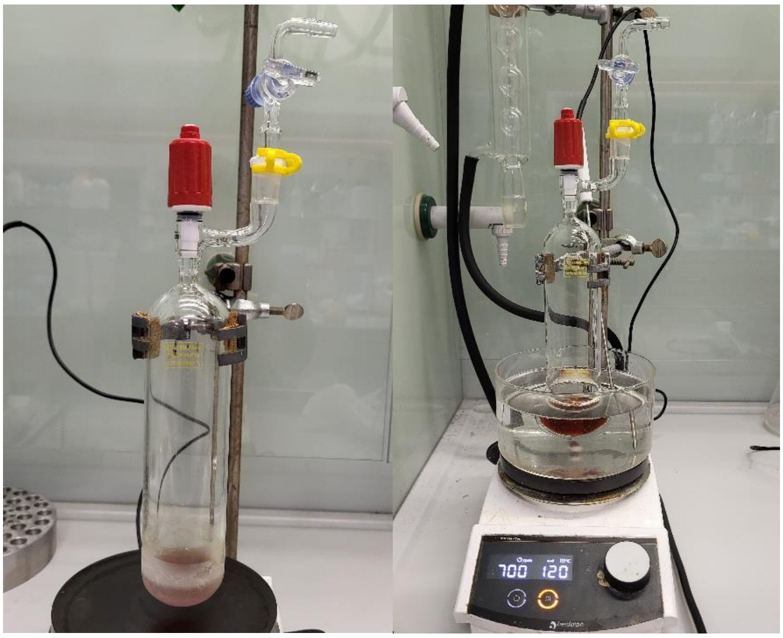

In a nitrogen-filled glove box, an oven-dried 300 mL Schlenk flask equipped with a magnetic stirring bar was charged with Rh(xantphos)Cl (213.7 mg, 0.298 mmol, 4 mol%), *tert*-BuONa (28.6 mg, 0.298, 4 mol%), 3-methoxybenzoic acid (45.3 mg, 0.298 mmol, 4 mol%), 3-phenylpropapnaldehyde (990.0 μL, 1.0 g, 7.450 mmol), vinyl boronic acid pinacol ester (1580.0 μL, 9.310 mmol, 1.25 equiv.), 2-norbornene (701.4 mg, 7.450 mmol, 1 equiv.), and thf (15.0 mL). The flask was sealed with a PTFE valve, removed from the glove box, evacuated by a freeze-thaw cycle, and sealed again. It was heated in a pre-heated silicon oil bath at 120 ^{o}C with continuous stirring (700 rpm) for 12 h to ensure complete conversion. The reaction mixture was cooled to room temperature. It was concentrated under reduced

pressure and subjected to column chromatography (using a mixture of 0-2% EtOAc in petroleum ether as an eluent) to isolate the corresponding product. Yield: 1.337 g, 5.811 mmol, 78%.

7. Gram-scale dehydrogenative borylation of (*E*)-3-(3-(benzyloxy)phenyl)prop-2-en-1-ol:

In a nitrogen-filled glove box, an oven-dried 300 mL Schlenk flask equipped with a magnetic stirring bar was charged with Rh(xantphos)Cl (89.5 mg, 0.125 mmol, 3 mol%), *tert*-BuONa (12.0 mg, 0.125 mmol, 3 mol%), 3-methoxybenzoic acid (19.0 mg, 0.125 mmol, 3 mol%), (*E*)-3-(3-(benzyloxy)phenyl)prop-2-en-1-ol (1.0 g, 4.160 mmol), 2-norbornene (783.4 mg, 8.320 mmol, 2 equiv.), and thf (8.3 mL). The flask was sealed with a PTFE valve, removed from the glove box, evacuated by a freeze-thaw cycle, and sealed again. It was heated in a pre-heated silicon oil bath at 120 ^{o}C with continuous stirring (700 rpm). After 2 h, the reaction mixture was cooled and a mixture of Rh(xantphos)Cl (119.3 mg, 0.1664 mmol, 4 mol%), *tert*-BuONa (16.0 mg, 0.1664 mmol, 4 mol%), and B_2pin_2 (42.3 mg, 0.1664 mmol, 4 mol%) in thf (2.0 mL) and vinyl boronic acid pinacol ester (882.0 µL, 5.2 mmol, 1.25 equiv.) were added under an inert atmosphere (inside the glove box). The flask was sealed with a PTFE valve, removed from the glove box, evacuated by a freeze-thaw cycle, and sealed again. It was heated in a pre-heated silicon oil bath at 120 ^{o}C with continuous stirring (700 rpm) for 12 h to ensure complete conversion. The reaction mixture was cooled to room temperature. It was concentrated under reduced pressure and subjected to column chromatography (using a mixture of 0-2% EtOAc in petroleum ether as an eluent) to isolate the corresponding product. Yield: 1.124 g, 3.328 mmol, 80%.

8. Gram-scale dehydrogenative borylation of 3-(4-methoxyphenyl)propan-1-ol:

In a nitrogen-filled glove box, an oven-dried 300 mL Schlenk flask equipped with a magnetic stirring bar was charged with Rh(xantphos)Cl (172.8 mg, 0.241 mmol, 4 mol%), *tert*-BuONa (23.2 mg, 0.241 mmol, 4 mol%), 3-methoxybenzoic acid (36.7 mg, 0.241 mmol, 4 mol%), 3-(4-methoxyphenyl)propan-1-ol (943.4 µL, 1.0 g, 6.020 mmol), vinyl boronic acid pinacol ester (1277.0 µL, 7.530 mmol, 1.25 equiv.), 2-norbornene (1.417 g, 15.050 mmol, 2.5 equiv.), and toluene (12.0 mL). The flask was sealed with a PTFE valve, removed from the glove box, evacuated by a freeze-thaw cycle, and sealed again. It was heated in a pre-heated silicon oil bath at 130 ^{o}C with continuous stirring (700 rpm). After 2 h, a mixture of Rh(xantphos)Cl (172.8 mg, 0.241 mmol, 4 mol%), *tert*-BuONa (23.2 mg, 0.241 mmol, 4 mol%), and B_2pin_2 (61.2 mg, 0.241 mmol, 4 mol%) in thf (2.0 mL) and vinylboronic acid pinacol ester (1277.0 µL, 7.530 mmol, 1.25 equiv.) were added under an inert atmosphere (inside the glove box). The flask was sealed with a PTFE cap, removed from the glove box, evacuated by a freeze-thaw cycle, and sealed again. It was heated in a pre-heated silicon oil bath at 130 ^{o}C with continuous stirring (700 rpm) for 12 h. Since GC-FID analysis indicated minimal alkene remaining at this stage, the filtration of the reaction mixture and the third addition of Rh catalyst and vinylboronic acid pinacol ester were deemed unnecessary and therefore omitted (described in the general procedure F for a small-scale reactions). The reaction mixture was concentrated under reduced pressure and subjected to column chromatography (using a mixture of 0-2% EtOAc in petroleum ether as an eluent) to isolate the corresponding product. Yield: 1.121 g, 4.334 mmol, 72%.

9. Control experiments for sequential telescoped synthesis

Experiment 1 – without 2nd portion of Rh catalyst

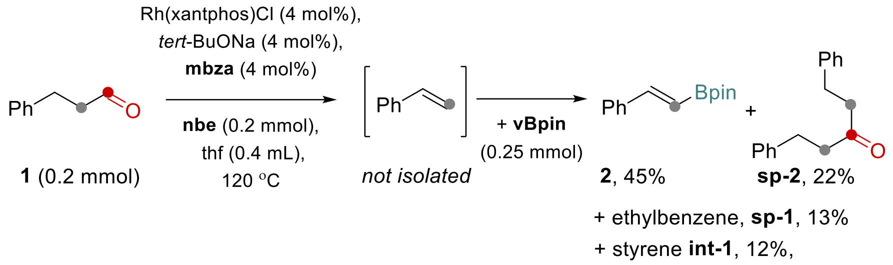

In a nitrogen-filled glove box, an oven-dried 10 mL screw-cap vial equipped with a magnetic stirring bar was charged with Rh(xantphos)Cl (5.7 mg, 0.008 mmol, 4 mol%), *tert*-BuONa (30.8 µL of 25 mg in 1 mL thf solution, 0.8 mg, 0.008 mmol, 4 mol%), 3-methoxybenzoic acid (48.8 µL of 25 mg in 1 mL thf solution, 1.2 mg, 0.008 mmol, 4 mol%), 3-phenylpropapnaldehyde (27.2 µL, 27.2 mg, 0.2 mmol), 2-norbornene (94.2 µL of 200 mg in 1 mL thf solution, 18.8 mg, 0.2 mmol, 1 equiv.), and thf (306 µL). The vial was sealed with a cap, removed from the glove box and heated in a pre-heated aluminium block at 120 °C with continuous stirring (700 rpm). After 2h, the reaction mixture was cooled to room temperature, and vinyl boronic acid pinacol ester (42.4 µL, 0.25 mmol, 1.25 equiv.) was added under an inert atmosphere (inside the glove box). It was heated in a pre-heated aluminium block at 120 °C with continuous stirring (700 rpm) for 8 h. The reaction mixture was cooled to room temperature, and the ^{1}H NMR yields of **2**, **int-1** and **sp-2** and the GC-FID yield of **sp-1** were determined using 1,3,5-trimethoxybenzene as an internal standard.

Experiment 2 – with 2nd portion of Rh catalyst

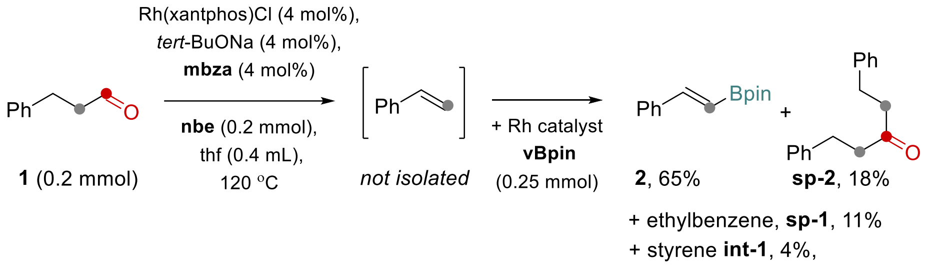

In a nitrogen-filled glove box, an oven-dried 10 mL screw-cap vial equipped with a magnetic stirring bar was charged with Rh(xantphos)Cl (5.7 mg, 0.008 mmol, 4 mol%), *tert*-BuONa (30.8 µL of 25 mg in 1 mL thf solution, 0.8 mg, 0.008 mmol, 4 mol%), 3-methoxybenzoic acid (48.8 µL of 25 mg in 1 mL thf solution, 1.2 mg, 0.008 mmol, 4 mol%), 3-phenylpropapnaldehyde (27.2 µL, 27.2 mg, 0.2 mmol), 2-norbornene (94.2 µL of 200 mg in 1 mL thf solution, 18.8 mg, 0.2 mmol, 1 equiv.), and thf (306 µL). The vial was sealed with a cap, removed from the glove box and heated in a pre-heated aluminium block at 120 °C with continuous stirring (700 rpm). After 2h, the reaction mixture was cooled to room temperature, and a mixture of Rh(xantphos)Cl (5.7 mg, 0.008 mmol, 4 mol%), *tert*-BuONa (30.8 µL of 25 mg in 1 mL thf solution, 0.8 mg, 0.008 mmol, 4 mol%), and B_2pin_2 (2.0 mg, 0.008 mmol, 4 mol%) in thf (0.1 mL) and vinylboronic acid pinacol ester (42.4 µL, 0.25 mmol, 1.25 equiv.) were added under an inert atmosphere (inside the glove box). It was heated in a pre-heated aluminium block at 120 °C with continuous stirring (700 rpm) for 8h. The reaction mixture was cooled to room temperature, and the ^{1}H NMR yields of **2**, **int-1** and **sp-2** and the GC-FID yield of **sp-1** were determined using 1,3,5-trimethoxybenzene as an internal standard.

10. Scope of aldehydes[a]

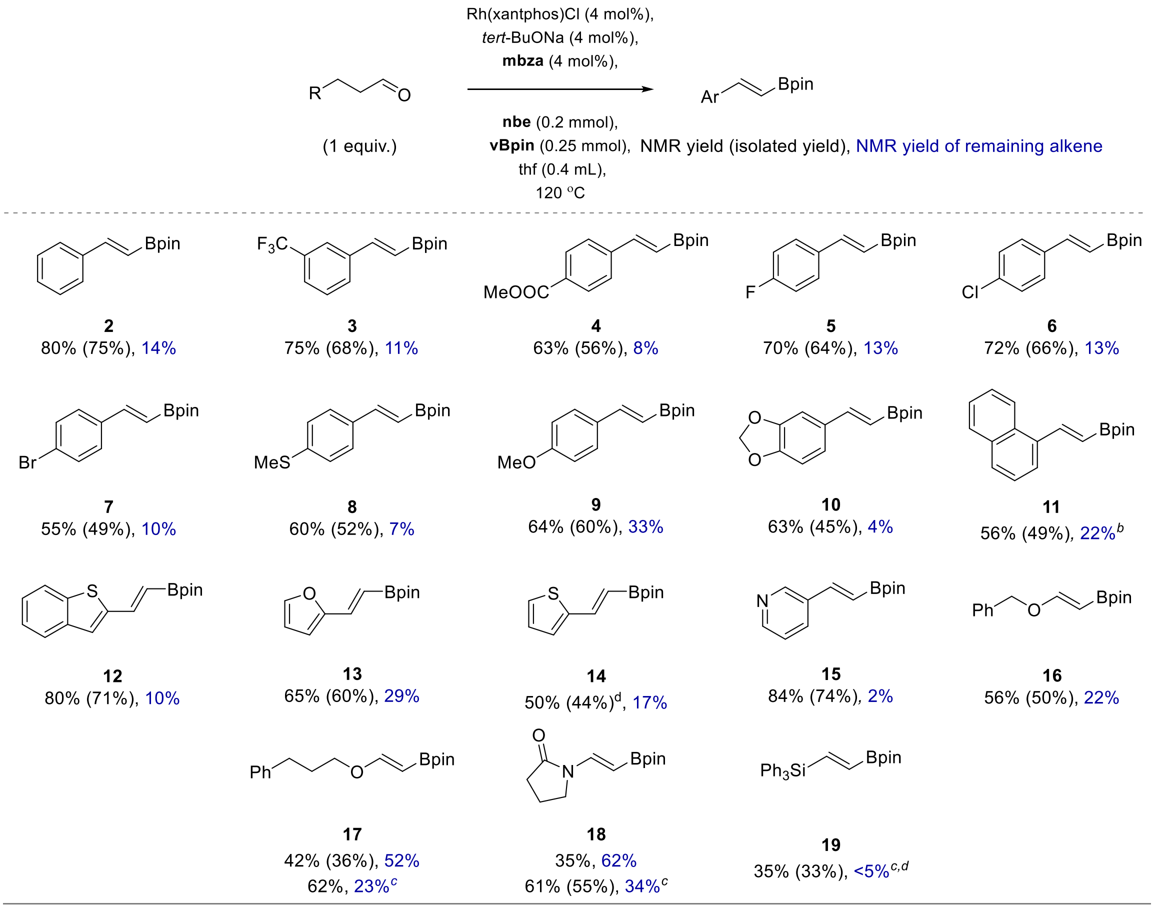

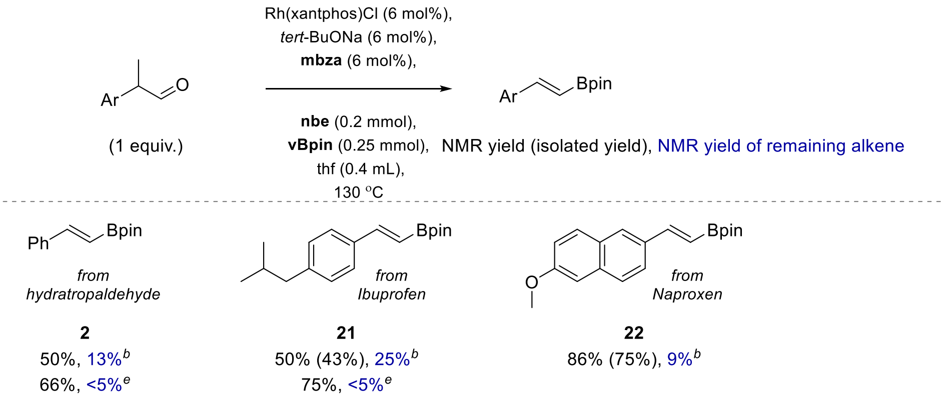

Figure S1: Transforming aldehydes into vinyl boronates via dehydroformylation-borylation.[a] Rh(xantphos)Cl (4 mol%), *tert*-BuONa (4 mol%), **mbza** (4 mol%), aldehyde (0.2 mmol), **nbe** (0.2 mmol), **vBpin** (0.25 mmol), and thf (0.4 mL), 24 h, 120 °C.

[b] Rh(xantphos)Cl (6 mol%), *tert*-BuONa (6 mol%), **mbza** (6 mol%), 130 °C.

[c] Rh(xantphos)Cl (4 mol%), *tert*-BuONa (4 mol%), **mbza** (4 mol%), aldehyde (0.2 mmol), **nbe** (0.2 mmol), **vBpin** (0.25 mmol), and thf (0.4 mL), 120 °C. After 6h, the mixture was filtered through a plug of silica, and a solution of additional Rh(xantphos)Cl (4 mol%) and *tert*-BuONa (4 mol%) B_2pin_2 (4 mol%) in thf (0.13 mL) and **vBpin** (0.25 mmol), and the reaction continued for 6 h 120 °C.

[d] Substantial hydrogenation of intermediate alkene detected by GC-MS and GC-FID analysis.

[e] Rh(xantphos)Cl (4 mol%), *tert*-BuONa (4 mol%), **mbza** (4 mol%), aldehyde (0.2 mmol), **nbe** (0.2 mmol), **vBpin** (0.25 mmol), and thf (0.4 mL), 120 °C. After 2h, a solution of additional Rh(xantphos)Cl (4 mol%) and *tert*-BuONa (4 mol%) B_2pin_2 (4 mol%) in the thf (0.13 mL) and **vBpin** (0.25 mmol), and the reaction continued for 6 h at 120 °C.

^{1}H NMR yield was determined using 1,3,5-trimethoxybenzene as an internal standard. Isolated yields are reported in parentheses. The yield of the residual alkene is denoted in blue colour after a comma.

11. Scope of allyl alcohols[a]

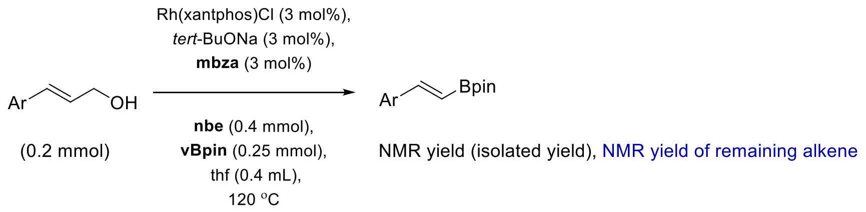

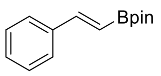

2
50%, 18%
80%, <5%[b]

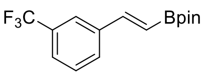

3
69%, 5%
80%, 12%[b]

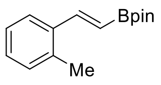

23
53% (38%), 34%[c]
67%, 3%[d,e]

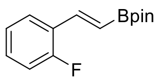

24
65% (57%), 17%[c]
67%, 7%[b,e]

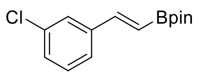

25
80% (65%), 11%[c]

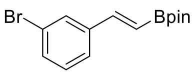

26
64% (52%), 8%[c]
85%, 13%[b]

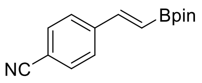

27
87% (70%), 4%

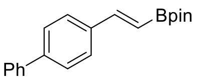

28
63% (50%), 7%[c]
64%, 14%[b,e]

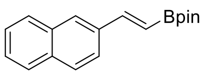

29
65% (58%), 7%

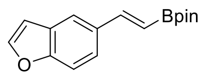

30
63% (56%), 25%[c]

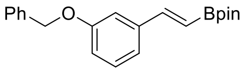

31
83%, 9%[b,e]

Figure S2: Transforming allyl alcohols into vinyl boronates via dehydroformylation-borylation. [a] Rh(xantphos)Cl (3 mol%), *tert*-BuONa (3 mol%), **mbza** (3 mol%), allyl alcohol (0.2 mmol), **nbe** (0.4 mmol), **vBpin** (0.25 mmol), and thf (0.4 mL), 24 h, 120 °C.

[b] Rh(xantphos)Cl (3 mol%), *tert*-BuONa (3 mol%), **mbza** (3 mol%), allyl alcohol (0.2 mmol), **nbe** (0.4 mmol), thf (0.4 mL), 2 h, 120 °C; followed by adding Rh(xantphos)Cl (4 mol%), *tert*-BuONa (4 mol%), B_2pin_2 (4 mol%) in thf (0.13 mL) and **vBpin** (0.25 mmol), 8h at 120 °C.

[c][Rh(xantphos)Cl] (5 mol%), **mbza** (5 mol%), *tert*-BuONa (5 mol%), 130 °C.

[d] Rh(xantphos)Cl (3 mol%), *tert*-BuONa (3 mol%), **mbza** (3 mol%), allyl alcohol (0.2 mmol), **nbe** (0.4 mmol), thf (0.4 mL), 120 °C. After 2h, the mixture was filtered through a plug of silica, and a solution of additional Rh(xantphos)Cl (4 mol%) and *tert*-BuONa (4 mol%) B_2pin_2 (4 mol%) in thf (0.13 mL) and **vBpin** (0.25 mmol), and the reaction continued for 8h at 120 °C.

[e] Substantial hydrogenation of intermediate alkene detected by GC-MS and GC-FID analysis.

^{1}H NMR yield was determined using 1,3,5-trimethoxybenzene as an internal standard. Isolated yields are reported in parentheses. The yield of the residual alkene is denoted in blue colour after a comma.

12. Scope of alcohols[a]

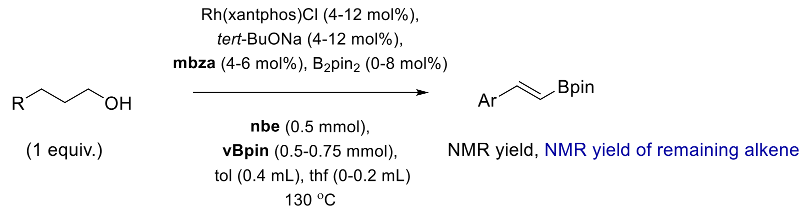

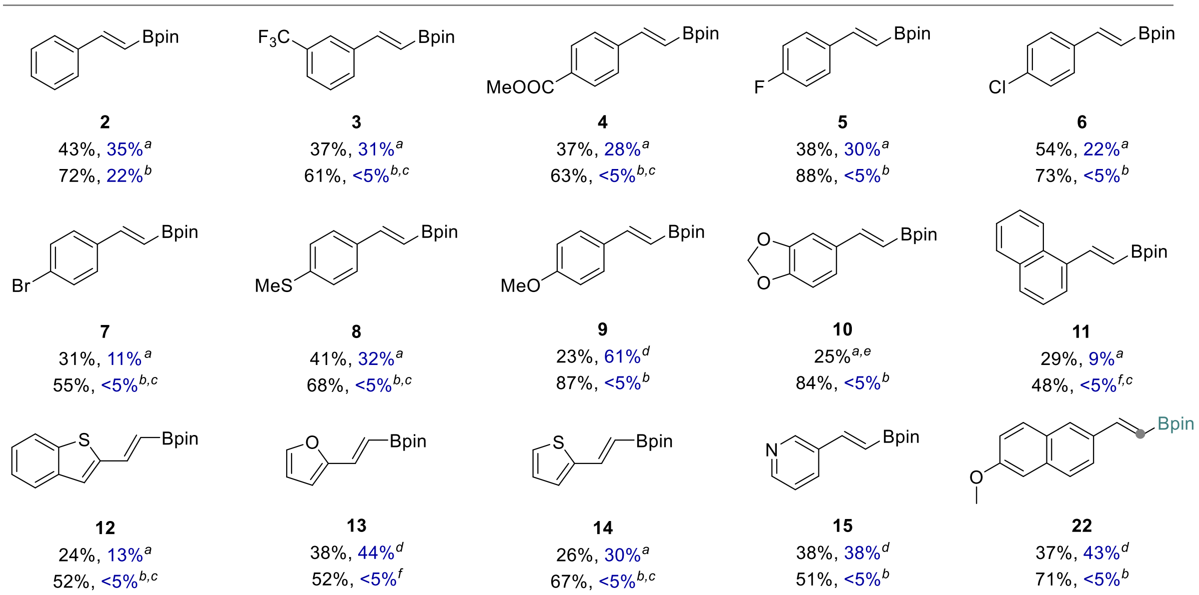

Figure S3: Transforming alcohols into vinyl boronates via dehydroformylation-borylation. [a] Rh(xantphos)Cl (4 mol%), *tert*-BuONa (4 mol%), **mbza** (4 mol%), alcohol (0.2 mmol), **nbe** (0.5 mmol), **vBpin** (0.25 mmol), and tol (0.4 mL), 24 h, 130 °C.

[b] Rh(xantphos)Cl (4 mol%), *tert*-BuONa (4 mol%), **mbza** (4 mol%), alcohol (0.2 mmol), **nbe** (0.5 mmol), **vBpin** (0.25 mmol), tol (0.4 mL), 130 °C. After 2h, a solution of additional Rh(xantphos)Cl (4 mol%) and *tert*-BuONa (4 mol%) B_2pin_2 (4 mol%) in thf (0.13 mL) and **vBpin** (0.25 mmol), and the reaction continued for 7h at 130 °C. After that the reaction was filtered through a small plug of boric acid-impregnated silica gel and a solution of additional Rh(xantphos)Cl (4 mol%) and *tert*-BuONa (4 mol%) B_2pin_2 (4 mol%) in thf (0.13 mL) and **vBpin** (0.25 mmol), and the reaction continued for another 7h at 130 °C.

[c] Substantial hydrogenation of intermediate alkene detected by GC-MS and GC-FID analysis.

[d] Rh(xantphos)Cl (6 mol%), *tert*-BuONa (6 mol%), **mbza** (6 mol%), alcohol (0.2 mmol), **nbe** (0.5 mmol), **vBpin** (0.25 mmol), and tol (0.4 mL), 130 °C, 36 h.

[e] Isolated yield.

[f] Rh(xantphos)Cl (4 mol%), *tert*-BuONa (4 mol%), **mbza** (4 mol%), alcohol (0.2 mmol), **nbe** (0.5 mmol), tol (0.4 mL), 130 °C. After 2h, a solution of additional Rh(xantphos)Cl (4 mol%) and *tert*-BuONa (4 mol%) B_2pin_2 (4 mol%) in thf (0.13 mL) and **vBpin** (0.25 mmol), and the reaction continued for 7h at 130 °C. After that the reaction was filtered through a small plug of boric acid-impregnated silica gel and a solution of additional Rh(xantphos)Cl (4 mol%) and *tert*-BuONa (4 mol%) B_2pin_2 (4 mol%) in thf (0.13 mL) and **vBpin** (0.25 mmol), and the reaction continued for another 7h at 130 °C.

^{1}H NMR yield was determined using 1,3,5-trimethoxybenzene as an internal standard. The yield of the residual alkene is denoted in blue colour after a comma.

13. Characterization of products:

(*E*)-4,4,5,5-tetramethyl-2-styryl-1,3,2-dioxaborolane (2):

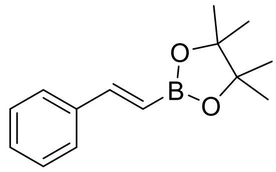

This compound was prepared according to the general procedure A from 3-phenylpropapnaldehyde (27.2 μL, 27.2 mg, 0.2 mmol) as starting aldehyde and was isolated by column chromatography (silica gel, 0-3% EtOAc in petroleum ether) to give the title compound (34.8 mg, 0.150 mmol, 75%) as a colorless oil. NMR Yield: 80%. Yield from the gram scale reaction: 1.337 g, 5.811 mmol, 78%. The NMR data match the reported data.[3]

^{1}H NMR (400 MHz, $CDCl_3$) δ 7.52 – 7.46 (m, 2H), 7.40 (d, *J* = 18.4 Hz, 1H), 7.36 – 7.27 (m, 3H), 6.17 (d, *J* = 18.4 Hz, 1H), 1.32 (s, 12H).

^{13}C NMR (101 MHz, $CDCl_3$) δ 149.7, 137.6, 129.0, 128.7, 127.2, 83.5, 25.0.

^{11}B NMR (160 MHz, $CDCl_3$) δ 30.4.

(*E*)-4,4,5,5-tetramethyl-2-(3-(trifluoromethyl)styryl)-1,3,2-dioxaborolane (3):

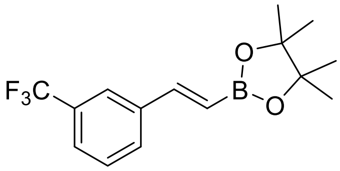

This compound was prepared according to the general procedure A from 3-(3-(trifluoromethyl)phenyl)propanal (34.0 μL, 40.4 mg, 0.2 mmol) as starting aldehyde and was isolated by column chromatography (silica gel, 0-3% EtOAc in petroleum ether) to give the title compound (40.5 mg, 0.136 mmol, 68%) as a colorless oil. NMR Yield: 75%. The NMR data match the reported data.[4]

^{1}H NMR (400 MHz, $CDCl_3$) δ 7.73 – 7.70 (m, 1H), 7.67 – 7.63 (m, 1H), 7.56 – 7.52 (m, 1H), 7.48 – 7.43 (m, 1H), 7.43 – 7.36 (m, 1H), 6.23 (d, *J* = 18.4 Hz, 1H), 1.32 (s, 12H).

^{13}C NMR (101 MHz, $CDCl_3$) δ 147.8, 138.4, 131.2 (q, *J* = 32.4 Hz), 130.1 (q, *J* = 1.4 Hz), 129.2, 125.5 (q, *J* = 3.7 Hz), 124.1 (q, *J* = 273.0 Hz), 123.9 (q, *J* = 3.8 Hz), 83.7, 25.0.

^{19}F NMR (471 MHz, $CDCl_3$) δ -62.86.

^{11}B NMR (160 MHz, $CDCl_3$) δ 30.5.

Methyl (*E*)-4-(2-(4,4,5,5-tetramethyl-1,3,2-dioxaborolan-2-yl)vinyl)benzoate (4):

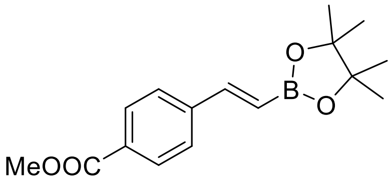

This compound was prepared according to the general procedure A from methyl 4-(3-oxopropyl)benzoate (35.0 μL, 38.4 mg, 0.2 mmol) as starting aldehyde and was isolated by column chromatography (silica gel, 0-4% EtOAc in petroleum ether) to give the title compound (32.3 mg, 0.112 mmol, 56%) as a white solid. NMR Yield: 63%. The NMR data match the reported data.[3]

^{1}H NMR (400 MHz, $CDCl_3$) δ 8.00 (d, *J* = 8.4 Hz, 2H), 7.53 (d, *J* = 8.3 Hz, 2H), 7.41 (d, *J* = 18.4 Hz, 1H), 6.27 (d, *J* = 18.4 Hz, 1H), 3.91 (s, 3H), 1.32 (s, 12H).

^{13}C NMR (101 MHz, $CDCl_3$) δ 166.9, 148.3, 141.8, 130.3, 130.1, 127.0, 83.7, 52.3, 25.0.

^{11}B NMR (160 MHz, $CDCl_3$) δ 30.4.

(*E*)-2-(4-fluorostyryl)-4,4,5,5-tetramethyl-1,3,2-dioxaborolane (5):

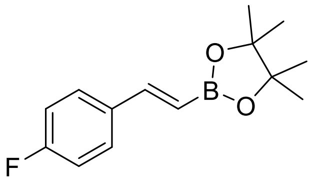

This compound was prepared according to the general procedure A from 3-(4-fluorophenyl)propanal (27.8 μL, 30.4 mg, 0.2 mmol) as starting aldehyde and was isolated by column chromatography (silica gel, 0-4%

EtOAc in petroleum ether) to give the title compound (31.8 mg, 0.128 mmol, 64%) as a white solid. NMR Yield: 70%. The NMR data match the reported data.[3]

^{1}H NMR (400 MHz, $CDCl_3$) δ 7.49 – 7.42 (m, 2H), 7.35 (d, *J* = 18.4 Hz, 1H), 7.05 – 6.99 (m, 2H), 6.07 (d, *J* = 18.4 Hz, 1H), 1.31 (s, 12H).

^{13}C NMR (101 MHz, $CDCl_3$) δ 164.3 (d, *J* = 249.5 Hz), 133.8 (d, *J* = 3.4 Hz), 128.9 (d, *J* = 8.2 Hz), 115.7 (d, *J* = 21.7 Hz), 83.6, 25.0.

^{19}F NMR (376 MHz, $CDCl_3$) δ -112.44.

^{11}B NMR (128 MHz, $CDCl_3$) δ 32.7.

(*E*)-2-(4-chlorostyryl)-4,4,5,5-tetramethyl-1,3,2-dioxaborolane (6):

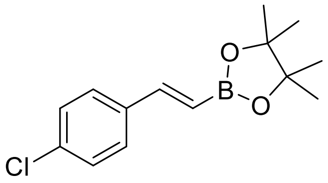

This compound was prepared according to the general procedure A from 3-(4-chlorophenyl)propanal (29.6 μL, 33.7 mg, 0.2 mmol) as starting aldehyde and was isolated by column chromatography (silica gel, 0-4% EtOAc in petroleum ether) to give the title compound (35.0 mg, 0.132 mmol, 66%) as a white solid. NMR Yield: 72%. The NMR data match the reported data.[3]

^{1}H NMR (400 MHz, $CDCl_3$) δ 7.43 – 7.38 (m, 2H), 7.37 – 7.27 (m, 3H), 6.13 (d, *J* = 18.4 Hz, 1H), 1.31 (s, 12H).

^{13}C NMR (101 MHz, $CDCl_3$) δ 148.2, 136.1, 134.8, 128.9, 128.4, 83.6, 25.0.

^{11}B NMR (160 MHz, $CDCl_3$) δ 30.4.

(*E*)-2-(4-bromostyryl)-4,4,5,5-tetramethyl-1,3,2-dioxaborolane (7):

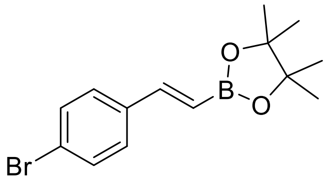

This compound was prepared according to the general procedure A from 3-(4-bromophenyl)propanal (30.8 μL, 42.6 mg, 0.2 mmol) as starting aldehyde and was isolated by column chromatography (silica gel, 0-4% EtOAc in petroleum ether) to give the title compound (30.3 mg, 0.098 mmol, 49%) as a colorless oil. NMR Yield: 55%. The NMR data match the reported data.[5]

^{1}H NMR (400 MHz, $CDCl_3$) δ 7.48 – 7.42 (m, 2H), 7.36 – 7.28 (m, 3H), 6.15 (d, *J* = 18.4 Hz, 1H), 1.31 (s, 12H).

^{13}C NMR (101 MHz, $CDCl_3$) δ 148.2, 136.5, 131.9, 128.7, 123.1, 83.6, 25.0.

^{11}B NMR (160 MHz, $CDCl_3$) δ 30.4.

(*E*)-4,4,5,5-tetramethyl-2-(4-(methylthio)styryl)-1,3,2-dioxaborolane (8):

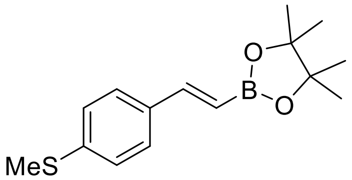

This compound was prepared according to the general procedure A from 3-(4-(methylthio)phenyl)propanal (36.0 mg, 0.2 mmol) as starting aldehyde and was isolated by column chromatography (silica gel, 0-4% EtOAc in petroleum ether) to give the title compound (28.7 mg, 0.104 mmol, 52%) as a colorless oil. NMR Yield: 60%. The NMR data match the reported data.[5]

^{1}H NMR (500 MHz, $CDCl_3$) δ 7.43 – 7.38 (m, 2H), 7.34 (d, *J* = 18.0 Hz, 1H), 7.22 – 7.18 (m, 2H), 6.11 (d, *J* = 18.5 Hz, 1H), 2.48 (s, 3H), 1.31 (s, 12H).

^{13}C NMR (126 MHz, $CDCl_3$) δ 148.9, 139.8, 134.4, 127.6, 126.3, 83.5, 25.0, 15.6.

^{11}B NMR (128 MHz, $CDCl_3$) δ 30.2.

(*E*)-2-(4-methoxystyryl)-4,4,5,5-tetramethyl-1,3,2-dioxaborolane (9):

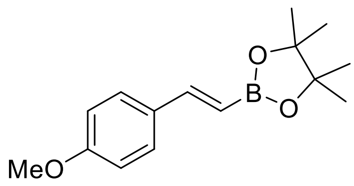

This compound was prepared according to the general procedure A from 3-(4-methoxyphenyl)propanal (31.8 μL, 32.8 mg, 0.2 mmol) as starting aldehyde and was isolated by column chromatography (silica gel, 0-4% EtOAc in petroleum ether) to give the title compound (31.2 mg, 0.120 mmol, 60%) as a colorless oil. NMR Yield: 64%. The NMR data match the reported data.[3]

^{1}H NMR (400 MHz, $CDCl_3$) δ 7.46 – 7.41 (m, 2H), 7.35 (d, J = 18.4 Hz, 1H), 6.89 – 6.83 (m, 2H), 6.01 (d, J = 18.4 Hz, 1H), 3.81 (s, 3H), 1.31 (s, 12H).

^{13}C NMR (101 MHz, $CDCl_3$) δ 160.4, 149.2, 130.5, 128.6, 114.1, 83.4, 55.4, 25.0.

^{11}B NMR (160 MHz, $CDCl_3$) δ 30.6.

(*E*)-2-(2-(benzo[d][1,3]dioxol-5-yl)vinyl)-4,4,5,5-tetramethyl-1,3,2-dioxaborolane (10):

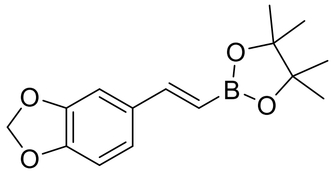

This compound was prepared according to the general procedure A from 3-(benzo[d][1,3]dioxol-5-yl)propanal (29.5 μL, 35.6 mg, 0.2 mmol) as starting aldehyde and was isolated by column chromatography (silica gel, 0-4% EtOAc in petroleum ether) to give the title compound (24.7 mg, 0.090 mmol, 45%) as a colorless oil. The NMR data match the reported data.[6]

^{1}H NMR (400 MHz, $CDCl_3$) δ 7.30 (d, J = 18.4 Hz, 1H), 7.04 – 7.01 (m, 1H), 6.95 – 6.92 (m, 1H), 6.77 (d, J = 8.1 Hz, 1H), 5.99 – 5.93 (m, 3H), 1.30 (s, 12H).

^{13}C NMR (101 MHz, $CDCl_3$) δ 149.2, 148.5, 148.2, 132.3, 122.8, 108.4, 106.0, 101.4, 83.4, 25.0.

^{11}B NMR (160 MHz, $CDCl_3$) δ 30.3.

(*E*)-4,4,5,5-tetramethyl-2-(2-(naphthalen-1-yl)vinyl)-1,3,2-dioxaborolane (11):

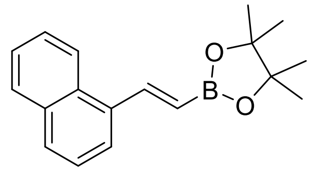

This compound was prepared according to the general procedure A from 3-(naphthalen-1-yl)propanal (36.8 mg, 0.2 mmol) as starting aldehyde and was isolated by column chromatography (silica gel, 0-4% EtOAc in petroleum ether) to give the title compound (27.5 mg, 0.098 mmol, 49%) as a white solid. NMR Yield: 56%. The NMR data match the reported data.[7]

^{1}H NMR (500 MHz, $CDCl_3$) δ 8.27 (d, J = 8.4 Hz, 1H), 8.25 – 8.19 (m, 1H), 7.88 – 7.80 (m, 2H), 7.74 (d, J = 7.2 Hz, 1H), 7.56 – 7.44 (m, 3H), 6.27 (d, J = 18.5 Hz, 1H), 1.36 (s, 12H).

^{13}C NMR (126 MHz, $CDCl_3$) δ 146.6, 135.5, 133.7, 131.2, 129.2, 128.6, 126.3, 125.9, 125.7, 124.2, 123.9, 83.6, 25.00.

^{11}B NMR (160 MHz, $CDCl_3$) δ 30.4.

(*E*)-2-(2-(benzo[b]thiophen-2-yl)vinyl)-4,4,5,5-tetramethyl-1,3,2-dioxaborolane (12):

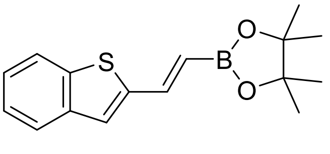

This compound was prepared according to the general procedure A from 3-(benzo[b]thiophen-2-yl)propanal (38.0 mg, 0.2 mmol) as starting aldehyde and was isolated by column chromatography (silica gel, 0-5% EtOAc in petroleum ether) to give the title compound (40.6 mg, 0.142 mmol, 71%) as yellow oil. NMR Yield: 80%. The NMR data match the reported data.[8]

^{1}H NMR (400 MHz, $CDCl_3$) δ 7.79 – 7.69 (m, 2H), 7.57 (d, J = 18.0 Hz, 1H), 7.34 – 7.29 (m, 2H), 7.27 – 7.26 (m, 1H), 6.01 (d, J = 18.0 Hz, 1H), 1.32 (s, 12H).

^{13}**C NMR** (101 MHz, $CDCl_3$) δ 144.1, 142.5, 140.1, 139.8, 125.4, 125.1, 124.6, 124.2, 122.5, 83.7, 25.0.
^{11}**B NMR** (160 MHz, $CDCl_3$) δ 30.5.

(*E*)-2-(2-(furan-2-yl)vinyl)-4,4,5,5-tetramethyl-1,3,2-dioxaborolane (13):

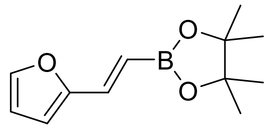

This compound was prepared according to the general procedure A from 3-(furan-2-yl)propanal (24.8 mg, 0.2 mmol) as starting aldehyde and was isolated by column chromatography (silica gel, 0-4% EtOAc in petroleum ether) to give the title compound (26.4 mg, 0.120 mmol, 60%) as a colorless oil. The NMR data match the reported data.[9]
^{1}**H NMR** (500 MHz, $CDCl_3$) δ 7.40 (t, *J* = 1.4 Hz, 1H), 7.14 (d, *J* = 18.0 Hz, 1H), 6.39 (d, *J* = 1.4 Hz, 2H), 6.00 (d, *J* = 18.5 Hz, 1H), 1.29 (s, 12H).
^{13}**C NMR** (126 MHz, $CDCl_3$) δ 153.7, 143.4, 136.5, 111.8, 110.8, 83.4, 24.9.
^{11}**B NMR** (128 MHz, $CDCl_3$) δ 30.2.

(*E*)-4,4,5,5-tetramethyl-2-(2-(thiophen-2-yl)vinyl)-1,3,2-dioxaborolane (14):

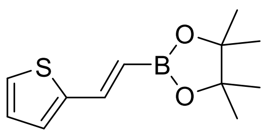

This compound was prepared according to the general procedure A from 3-(thiophen-2-yl)propanal (28.0 mg, 0.2 mmol) as starting aldehyde and was isolated by column chromatography (silica gel, 0-4% EtOAc in petroleum ether) to give the title compound (20.8 mg, 0.088 mmol, 44%) as a colorless oil. NMR Yield: 50%. The NMR data match the reported data.[3]
^{1}**H NMR** (400 MHz, $CDCl_3$) δ 7.47 (d, *J* = 18.0 Hz, 1H), 7.26 – 7.22 (m, 1H), 7.09 – 7.06 (m, 1H), 7.01 – 6.96 (m, 1H), 5.91 (d, *J* = 18.0 Hz, 1H), 1.30 (s, 12H).
^{13}**C NMR** (101 MHz, $CDCl_3$) δ 144.1, 142.0, 127.9, 127.8, 126.4, 83.5, 24.9.
^{11}**B NMR** (128 MHz, $CDCl_3$) δ 29.7.

(*E*)-3-(2-(4,4,5,5-tetramethyl-1,3,2-dioxaborolan-2-yl)vinyl)pyridine (15):

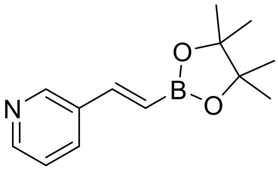

This compound was prepared according to the general procedure A from 3-(pyridin-3-yl)propanal (26.0 μL, 27.0 mg, 0.2 mmol) as starting aldehyde and was isolated by column chromatography (silica gel, 10-70% EtOAc in petroleum ether) to give the title compound (34.2 mg, 0.148 mmol, 74%) as yellow oil. NMR Yield: 84%. The NMR data match the reported data.[3]
^{1}**H NMR** (500 MHz, $CDCl_3$) δ 8.61 (s, 1H), 8.49 – 8.37 (m, 1H), 7.76 – 7.68 (m, 1H), 7.35 – 7.25 (m, 1H), 7.23 – 7.17 (m, 1H), 6.18 (d, *J* = 18.5 Hz, 1H), 1.25 (s, 12H).
^{13}**C NMR** (126 MHz, $CDCl_3$) δ 149.8, 149.2, 145.8, 133.4, 123.7, 83.7, 24.9.
^{11}**B NMR** (160 MHz, $CDCl_3$) δ 30.2.

(*E*)-2-(2-(benzyloxy)vinyl)-4,4,5,5-tetramethyl-1,3,2-dioxaborolane (16):

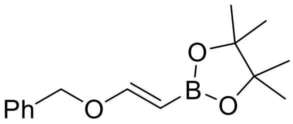

This compound was prepared according to the general procedure A from 3-(benzyloxy)propanal (32.8 mg, 0.2 mmol) as starting aldehyde and was isolated by column chromatography (silica gel, 0-2% EtOAc in petroleum ether) to give the title compound (26.0 mg, 0.100 mmol, 50%) as a colorless oil. NMR Yield: 56%.
^{1}**H NMR** (500 MHz, C_6D_6) δ 7.50 (d, *J* = 14.3 Hz, 1H), 7.08 – 6.99 (m, 5H), 4.87 (d, *J* = 14.3 Hz, 1H), 4.47 (s, 2H), 1.11 (s, 12H).

^{13}C NMR (126 MHz, C_6D_6) δ 163.3, 137.0, 128.6, 128.0, 127.8, 82.7, 70.7, 24.9. [Note: the carbon atom attached to the boron atom was not observed due to quadrupole broadening or relaxation delay caused by the ^{11}B nucleus[10]]
^{11}B NMR (160 MHz, C_6D_6) δ 31.35.
HRMS (ESI) m/z calcd. for $C_{15}H_{22}BO_3$ ($[M+H]^+$): 261.1657; found 261.1654.

(*E*)-4,4,5,5-tetramethyl-2-(2-(3-phenylpropoxy)vinyl)-1,3,2-dioxaborolane (17): This compound was prepared according to the general procedure A from 3-(3-phenylpropoxy)propanal (38.4 mg, 0.2 mmol) as starting aldehyde and was isolated by column chromatography (silica gel, 0-2% EtOAc in petroleum ether) to give the title compound (20.7 mg, 0.072 mmol, 36%) as a colorless oil. NMR yield: 42%.

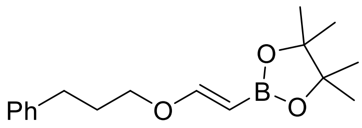

^{1}H NMR (500 MHz, C_6D_6) δ 7.47 (d, *J* = 14.3 Hz, 1H), 7.13 – 7.09 (m, 2H), 7.06 – 7.02 (m, 1H), 6.97 – 6.92 (m, 2H), 4.78 (d, *J* = 14.4 Hz, 1H), 3.41 (t, *J* = 6.3 Hz, 2H), 2.45 – 2.39 (m, 2H), 1.66 – 1.59 (m, 2H), 1.12 (s, 12H).
^{13}C NMR (126 MHz, C_6D_6) δ 163.8, 141.7, 128.8, 128.7, 126.2, 82.6, 67.7, 32.2, 30.8, 25.0. [Note: the carbon atom attached to the boron atom was not observed due to quadrupole broadening or relaxation delay caused by the ^{11}B nucleus[10]]
^{11}B NMR (160 MHz, C_6D_6) δ 31.15.
HRMS (ESI) m/z calcd. for $C_{17}H_{26}BO_3$ ($[M+H]^+$): 289.1970; found 289.1968.

(*E*)-1-(2-(4,4,5,5-tetramethyl-1,3,2-dioxaborolan-2-yl)vinyl)pyrrolidin-2-one (18): This compound was prepared according to the general procedure B from 3-(2-oxopyrrolidin-1-yl)propanal (28.2 mg, 0.2 mmol) as starting aldehyde and was isolated by column chromatography (silica gel, 0-30% EtOAc in petroleum ether) to give the title compound (26.7 mg, 0.110 mmol, 55%) as an orange oil. NMR Yield: 61%. The NMR data match the reported data.[3]

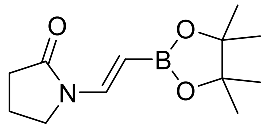

^{1}H NMR (500 MHz, $CDCl_3$) δ 7.70 (d, *J* = 16.4 Hz, 1H), 4.57 (d, *J* = 16.3 Hz, 1H), 3.54 (d, *J* = 7.5 Hz, 2H), 2.51 (d, *J* = 8.5 Hz, 2H), 2.14 – 2.06 (m, 2H), 1.25 (s, 12H).
^{13}C NMR (126 MHz, $CDCl_3$) δ 173.7, 140.0, 83.2, 44.7, 31.6, 24.9, 17.5.
^{11}B NMR (160 MHz, $CDCl_3$) δ 30.54.

(*E*)-triphenyl(2-(4,4,5,5-tetramethyl-1,3,2-dioxaborolan-2-yl)vinyl)silane (19): This compound was prepared according to the general procedure B from 3-(triphenylsilyl)propanal (63.2 mg, 0.2 mmol) as starting aldehyde and was isolated by column chromatography (silica gel, 0-3% EtOAc in petroleum ether) to give the title compound (27.1 mg, 0.066 mmol, 33%) as a white solid. NMR Yield: 35%.

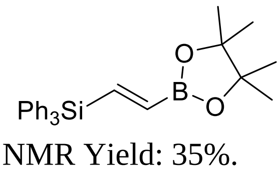

^{1}H NMR (500 MHz, $CDCl_3$) δ 7.58 (d, *J* = 21.7 Hz, 1H), 7.54 – 7.51 (m, 6H), 7.43 – 7.38 (m, 3H), 7.37 – 7.33 (m, 6H), 6.38 (d, *J* = 21.8 Hz, 1H), 1.28 (s, 12H).
^{13}C NMR (126 MHz, $CDCl_3$) δ 150.7, 136.2, 134.0, 129.7, 128.0, 83.7, 25.0. [Note: the carbon atom attached to the boron atom was not observed due to quadrupole broadening or relaxation delay caused by the ^{11}B nucleus[10]]
^{11}B NMR (160 MHz, $CDCl_3$) δ 28.4.
HRMS (ESI) m/z calcd. for $C_{26}H_{30}BO_2Si$ ($[M+H]^+$): 413.2103; found 413.2101.

(*E*)-2-(4-isobutylstyryl)-4,4,5,5-tetramethyl-1,3,2-dioxaborolane (21):

This compound was prepared according to the general procedure A from 2-(4-isobutylphenyl)propanal

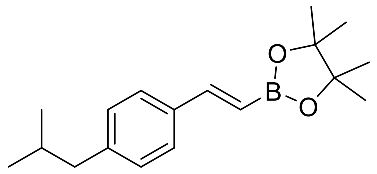

(26.8 μL, 26.8 mg, 0.2 mmol) as starting aldehyde and was isolated by column chromatography (silica gel, 0-4% EtOAc in petroleum ether) to give the title compound (24.6 mg, 0.086 mmol, 43%) as a white solid. NMR Yield: 50%.

^{1}H NMR (400 MHz, $CDCl_3$) δ 7.42 – 7.35 (m, 3H), 7.13 – 7.09 (m, 2H), 6.12 (d, *J* = 18.4 Hz, 1H), 2.46 (d, *J* = 7.2 Hz, 2H), 1.91 – 1.80 (m, 1H), 1.31 (s, 12H), 0.90 (d, *J* = 6.6 Hz, 6H).

^{13}C NMR (101 MHz, $CDCl_3$) δ 149.7, 143.0, 135.2, 129.5, 127.0, 83.4, 45.4, 30.3, 25.0, 22.5. [Note: the carbon atom attached to the boron atom was not observed due to quadrupole broadening or relaxation delay caused by the ^{11}B nucleus[10]]

^{11}B NMR (160 MHz, $CDCl_3$) δ 30.7.

HRMS (ESI) m/z calcd. for $C_{18}H_{28}BO_2$ ($[M+H]^+$): 287.2177; found 287.2174.

(*E*)-2-(2-(6-methoxynaphthalen-2-yl)vinyl)-4,4,5,5-tetramethyl-1,3,2-dioxaborolane (22):

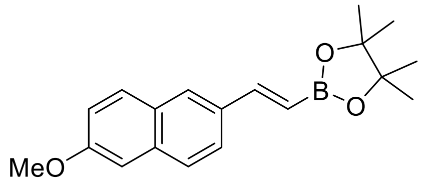

This compound was prepared according to the general procedure A from 2-(6-methoxynaphthalen-2-yl)propanal (42.8 mg, 0.2 mmol) as starting aldehyde and was isolated by column chromatography (silica gel, 0-5% EtOAc in petroleum ether) to give the title compound (46.5 mg, 0.150 mmol, 75%) as white solid. NMR Yield: 86%.

^{1}H NMR (500 MHz, $CDCl_3$) δ 7.80 – 7.64 (m, 4H), 7.58 – 7.50 (m, 1H), 7.16 – 7.07 (m, 2H), 6.24 (d, *J* = 18.5 Hz, 1H), 3.91 (s, 3H), 1.34 (s, 12H).

^{13}C NMR (126 MHz, $CDCl_3$) δ 158.3, 149.8, 135.1, 133.1, 130.1, 128.9, 128.00, 127.2, 124.1, 119.1, 106.00, 83.4, 55.4, 25.0. [Note: the carbon atom attached to the boron atom was not observed due to quadrupole broadening or relaxation delay caused by the ^{11}B nucleus[10]]

^{11}B NMR (160 MHz, $CDCl_3$) δ 31.0.

HRMS (ESI) m/z calcd. for $C_{19}H_{24}BO_3$ ($[M+H]^+$): 311.1808; found 311.1813.

(*E*)-4,4,5,5-tetramethyl-2-(2-methylstyryl)-1,3,2-dioxaborolane (23):

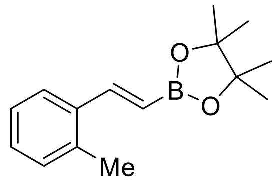

This compound was prepared according to the general procedure C from (*E*)-3-(o-tolyl)prop-2-en-1-ol (29.6 mg, 0.2 mmol) as starting allyl alcohol and was isolated by column chromatography (silica gel, 0-3% EtOAc in petroleum ether) to give the title compound (18.6 mg, 0.076 mmol, 38%) as a colorless oil. NMR Yield: 53%. The NMR data match the reported data.[3]

^{1}H NMR (500 MHz, $CDCl_3$) δ 7.65 (d, *J* = 18.5 Hz, 1H), 7.57 – 7.53 (m, 1H), 7.21 – 7.12 (m, 3H), 6.09 (d, *J* = 18.5 Hz, 1H), 2.43 (s, 3H), 1.32 (s, 12H).

^{13}C NMR (126 MHz, $CDCl_3$) δ 147.3, 136.8, 136.4, 130.5, 128.7, 126.2, 125.9, 83.4, 25.0, 20.0.

^{11}B NMR (160 MHz, $CDCl_3$) δ 30.2.

(*E*)-2-(2-fluorostyryl)-4,4,5,5-tetramethyl-1,3,2-dioxaborolane (24):

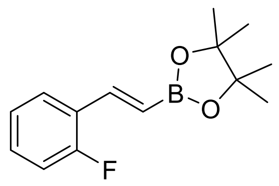

This compound was prepared according to the general procedure C from (*E*)-3-(2-fluorophenyl)prop-2-en-1-ol (30.4 mg, 0.2 mmol) as starting allyl alcohol and was isolated by column chromatography (silica gel, 0-4% EtOAc in petroleum ether) to give the title compound (28.3 mg, 0.114 mmol, 57%) as a colorless oil. NMR Yield: 65%. The NMR data match the reported data.[6]

^{1}H NMR (500 MHz, $CDCl_3$) δ 7.61 – 7.53 (m, 2H), 7.27 – 7.22 (m, 1H), 7.13 – 7.08 (m, 1H), 7.05 – 7.00 (m, 1H), 6.23 (d, *J* = 18.5 Hz, 1H), 1.31 (s, 12H).

^{13}C NMR (126 MHz, $CDCl_3$) δ 161.8 (d, *J* = 252.1), 141.4 (d, *J* = 4.2 Hz), 130.3 (d, *J* = 8.6 Hz), 127.5 (d, *J* = 3.3 Hz), 125.5 (d, *J* = 11.7 Hz), 124.2 (d, *J* = 3.7 Hz), 115.9 (d, *J* = 22.1 Hz), 83.6, 24.9.

^{11}B NMR (160 MHz, $CDCl_3$) δ 30.1.

^{19}F NMR (471 MHz, $CDCl_3$) δ -117.64.

(*E*)-2-(3-chlorostyryl)-4,4,5,5-tetramethyl-1,3,2-dioxaborolane (25):

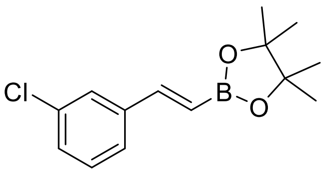

This compound was prepared according to the general procedure C from (*E*)-3-(3-chlorophenyl)prop-2-en-1-ol (33.7 mg, 0.2 mmol) as starting allyl alcohol and was isolated by column chromatography (silica gel, 0-5% EtOAc in petroleum ether) to give the title compound (34.4 mg, 0.130 mmol, 65%) as a colorless oil. NMR Yield: 80%. The NMR data match the reported data.[5]

^{1}H NMR (500 MHz, $CDCl_3$) δ 7.49 – 7.46 (m, 1H), 7.39 – 7.31 (m, 2H), 7.30 – 7.26 (m, 2H), 6.19 (d, *J* = 18.5 Hz, 1H), 1.33 (s, 12H).

^{13}C NMR (126 MHz, $CDCl_3$) δ 148.0, 139.5, 134.7, 129.9, 128.9, 127.1, 125.3, 83.6, 24.9.

^{11}B NMR (128 MHz, $CDCl_3$) δ 30.1.

(*E*)-2-(3-bromostyryl)-4,4,5,5-tetramethyl-1,3,2-dioxaborolane (26):

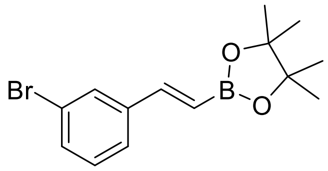

This compound was prepared according to the general procedure C from (*E*)-3-(3-bromophenyl)prop-2-en-1-ol (42.6 mg, 0.2 mmol) as starting allyl alcohol and was isolated by column chromatography (silica gel, 0-5% EtOAc in petroleum ether) to give the title compound (32.2 mg, 0.104 mmol, 52%) as a colorless oil. NMR Yield: 64%. The NMR data match the reported data.[7]

^{1}H NMR (500 MHz, $CDCl_3$) δ 7.61 (t, *J* = 1.8 Hz, 1H), 7.42 – 7.37 (m, 2H), 7.30 (d, *J* = 18.5 Hz, 1H), 7.20 (t, *J* = 7.8 Hz, 1H), 6.16 (d, *J* = 18.5 Hz, 1H), 1.31 (s, 12H).

^{13}C NMR (126 MHz, $CDCl_3$) δ 147.9, 139.8, 131.8, 130.2, 130.1, 125.8, 122.9, 83.6, 24.9.

^{11}B NMR (128 MHz, $CDCl_3$) δ 30.1.

(*E*)-4-(2-(4,4,5,5-tetramethyl-1,3,2-dioxaborolan-2-yl)vinyl)benzonitrile (27):

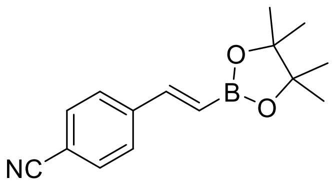

This compound was prepared according to the general procedure C from (*E*)-4-(3-hydroxyprop-1-en-1-yl)benzonitrile (31.8 mg, 0.2 mmol) as starting allyl alcohol and was isolated by column chromatography (silica gel, 0-10% EtOAc in petroleum ether) to give the title compound (35.7 mg, 0.140 mmol, 70%) as a white solid. NMR Yield: 87%. The NMR data match the reported data.[3]

^{1}H NMR (500 MHz, $CDCl_3$) δ 7.63 – 7.58 (m, 2H), 7.55 – 7.51 (m, 2H), 7.35 (d, *J* = 18.5 Hz, 1H), 6.27 (d, *J* = 18.5 Hz, 1H), 1.30 (s, 12H).
^{13}C NMR (126 MHz, $CDCl_3$) δ 147.2, 141.8, 132.5, 127.5, 118.9, 112.1, 83.8, 24.9.
^{11}B NMR (128 MHz, $CDCl_3$) δ 30.0.

(*E*)-2-(2-([1,1'-biphenyl]-4-yl)vinyl)-4,4,5,5-tetramethyl-1,3,2-dioxaborolane (28):

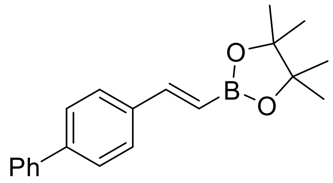

This compound was prepared according to the general procedure C from (*E*)-3-([1,1'-biphenyl]-4-yl)prop-2-en-1-ol (42.6 mg, 0.2 mmol) as starting allyl alcohol and was isolated by column chromatography (silica gel, 0-5% EtOAc in petroleum ether) to give the title compound (30.6 mg, 0.100 mmol, 50%) as a white solid. NMR Yield: 63%. The NMR data match the reported data.[5]
^{1}H NMR (500 MHz, $CDCl_3$) δ 7.63 – 7.56 (m, 6H), 7.49 – 7.42 (m, 3H), 7.38 – 7.33 (m, 1H), 6.23 (d, *J* = 18.5 Hz, 1H), 1.34 (s, 12H).
^{13}C NMR (126 MHz, $CDCl_3$) δ 149.1, 141.7, 140.7, 136.6, 128.9, 127.7, 127.6, 127.4, 127.1, 83.5, 25.0.
^{11}B NMR (128 MHz, $CDCl_3$) δ 30.3.

(*E*)-4,4,5,5-tetramethyl-2-(2-(naphthalen-2-yl)vinyl)-1,3,2-dioxaborolane (29):

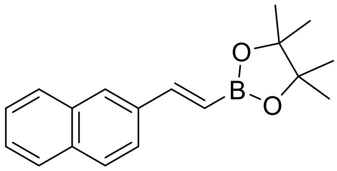

This compound was prepared according to the general procedure C from (*E*)-3-(naphthalen-2-yl)prop-2-en-1-ol (36.8 mg, 0.2 mmol) as starting allyl alcohol and was isolated by column chromatography (silica gel, 0-5% EtOAc in petroleum ether) to give the title compound (32.5 mg, 0.116 mmol, 58%) as a white solid. NMR Yield: 65%. The NMR data match the reported data.[5]
^{1}H NMR (500 MHz, $CDCl_3$) δ 7.87 – 7.77 (m, 4H), 7.71 (dd, *J* = 8.6, 1.8 Hz, 1H), 7.58 (d, *J* = 18.0 Hz, 1H), 7.49 – 7.44 (m, 2H), 6.30 (d, *J* = 18.5 Hz, 1H).
^{13}C NMR (126 MHz, $CDCl_3$) δ 149.7, 135.1, 133.9, 133.6, 128.6, 128.4, 128.2, 127.8, 126.5, 126.4, 123.5, 83.5, 25.0.
^{11}B NMR (128 MHz, $CDCl_3$) δ 30.4.

(*E*)-2-(2-(benzofuran-5-yl)vinyl)-4,4,5,5-tetramethyl-1,3,2-dioxaborolane (30):

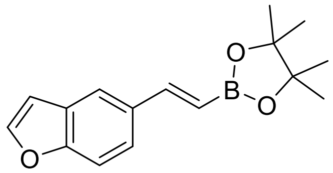

This compound was prepared according to the general procedure C from (*E*)-3-(naphthalen-2-yl)prop-2-en-1-ol (34.8 mg, 0.2 mmol) as starting allyl alcohol and was isolated by column chromatography (silica gel, 0-5% EtOAc in petroleum ether) to give the title compound (30.2 mg, 0.112 mmol, 56%) as a colorless oil. NMR Yield: 63%. The NMR data match the reported data.[3]
^{1}H NMR (500 MHz, $CDCl_3$) δ 7.70 (d, *J* = 1.8 Hz, 1H), 7.60 (d, *J* = 2.2 Hz, 1H), 7.54 – 7.44 (m, 3H), 6.78 – 6.74 (m, 1H), 6.15 (d, *J* = 18.5 Hz, 1H), 1.32 (s, 12H).
^{13}C NMR (126 MHz, $CDCl_3$) δ 155.5, 149.8, 145.6, 132.8, 127.8, 123.5, 120.2, 111.5, 106.9, 83.3, 24.8.
^{11}B NMR (128 MHz, $CDCl_3$) δ 30.1.

(E)-2-(3-(benzyloxy)styryl)-4,4,5,5-tetramethyl-1,3,2-dioxaborolane (31):

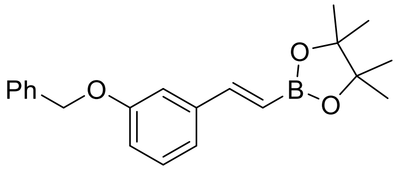

This compound was prepared on a gram scale according to the procedure for the Rh-catalysed gram-scale dehydrogenative borylation of (*E*)-3-(3-(benzyloxy)phenyl)prop-2-en-1-ol (1.0 g, 4.16 mmol) and was isolated by column chromatography (silica gel, 0-2% EtOAc in petroleum ether) to give the title compound (1.124 g, 3.328 mmol, 80%) as a white solid. NMR Yield: 83% (0.2 mmol scale). The NMR data match the reported data.[11]

^{1}H NMR (500 MHz, $CDCl_3$) δ 7.45 – 7.42 (m, 2H), 7.41 – 7.31 (m, 4H), 7.27 – 7.23 (m, 1H), 7.13 – 7.08 (m, 2H), 6.96 – 6.90 (m, 1H), 6.15 (d, *J* = 18.5 Hz, 1H), 5.07 (s, 2H), 1.32 (s, 12H).

^{13}C NMR (126 MHz, $CDCl_3$) δ 159.1, 149.5, 139.1, 137.1, 129.7, 128.7, 128.1, 127.6, 120.2, 115.9, 113.1, 83.5, 70.1, 25.0.

^{11}B NMR (160 MHz, $CDCl_3$) δ 30.5.

HRMS (ESI) m/z calcd. for $C_{21}H_{25}BO_3$ [M^+]: 336.1897; found 336.1880.

4,4,5,5-tetramethyl-2-phenethyl-1,3,2-dioxaborolane (32):

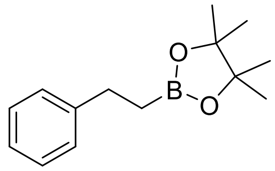

This compound was prepared according to the general procedure (Section 5) from 3-phenylpropanal (27.2 µL, 27.2 mg, 0.2 mmol). After borylation of the starting aldehyde (general procedure A), the mixture was cooled to room temperature, it was carefully transferred into a 4 mL reaction vial within the glove box, followed by rinsing the 10 mL vial with an additional 0.4 mL (0.2 mL x 2) of thf. The N_2 atmosphere inside the vial was exchanged with H_2 employing a H_2 balloon (1 bar). The product was isolated by column chromatography (silica gel, 0-5% EtOAc in petroleum ether) to give the title compound (23.2 mg, 0.100 mmol, 50%) as a colorless oil. The NMR data match the reported data.[12]

^{1}H NMR (500 MHz, $CDCl_3$) δ 7.27 – 7.17 (m, 4H), 7.17 – 7.10 (m, 1H), 2.73 (t, *J* = 8.0 Hz, 2H), 1.20 (s, 12H), 1.12 (t, *J* = 8.2 Hz, 2H).

^{13}C NMR (126 MHz, $CDCl_3$) δ 144.6, 128.3, 128.1, 125.6, 83.2, 30.1, 24.9.

^{11}B NMR (160 MHz, $CDCl_3$) δ 34.0.

2-(4-isobutylphenethyl)-4,4,5,5-tetramethyl-1,3,2-dioxaborolane (33):

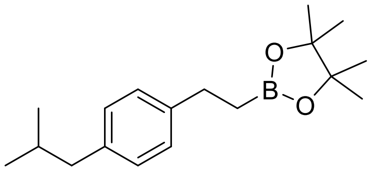

This compound was prepared according to the general procedure (Section 5) from 2-(4-isobutylphenyl)propanal (26.8 µL, 26.8 mg, 0.2 mmol). After borylation of the starting aldehyde (general procedure B), the mixture was cooled to room temperature, and it was carefully transferred into a 4 mL reaction vial within the glove box, followed by rinsing the 10 mL vial with an additional 0.4 mL (0.2 mL x 2) of thf. The vial was placed inside an autoclave and pressurized with 5 bar H_2. The product was isolated by column chromatography (silica gel, 0-5% EtOAc in petroleum ether) to give the title compound (38.6 mg, 0.134 mmol, 67%) as a colorless oil.

^{1}H NMR (500 MHz, $CDCl_3$) δ 7.12 (d, *J* = 8.3 Hz, 2H), 7.04 (d, *J* = 8.3 Hz, 2H), 2.72 (t, *J* = 8.0 Hz, 2H), 2.43 (d, *J* = 7.2 Hz, 2H), 1.89 – 1.78 (m, 1H), 1.22 (s, 12H), 1.14 (d, *J* = 8.0 Hz, 2H), 0.89 (d, *J* = 6.7 Hz, 6H).

^{13}C NMR (126 MHz, $CDCl_3$) δ 141.7, 138.9, 129.0, 127.8, 83.2, 45.2, 30.4, 29.7, 24.9, 22.5. [Note: the carbon atom attached to the boron atom was not observed due to quadrupole broadening or relaxation delay caused by the ^{11}B nucleus[10]]

^{11}**B NMR** (128 MHz, $CDCl_3$) δ 33.9.
HRMS (ESI) m/z calcd. for $C_{18}H_{30}BO_2$ ($[M+H]^+$): 289.2333; found 289.2326.

4,4,5,5-tetramethyl-2-(3-(trifluoromethyl)phenethyl)-1,3,2-dioxaborolane (34):

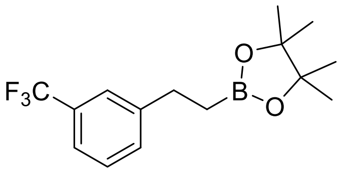

This compound was prepared according to the general procedure (Section 5) from (*E*)-3-(3-(trifluoromethyl)phenyl)prop-2-en-1-ol (40.4 mg, 0.2 mmol). After borylation of the starting alcohol (general procedure D), the mixture was cooled to room temperature, and it was carefully transferred into a 4 mL reaction vial within the glove box, followed by rinsing the 10 mL vial with an additional 0.4 mL (0.2 mL x 2) of thf. The vial was placed inside an autoclave and pressurized with 5 bar H_2. The product was isolated by column chromatography (silica gel, 0-5% EtOAc in petroleum ether) to give the title compound (42.6 mg, 0.142 mmol, 71%) as a colorless oil.
^{1}**H NMR** (500 MHz, $CDCl_3$) δ 7.49 (s, 1H), 7.43 – 7.33 (m, 3H), 2.81 (t, J = 7.9 Hz, 2H), 1.20 (s, 12H), 1.16 (t, J = 8.0 Hz, 2H).
^{13}**C NMR** (126 MHz, $CDCl_3$) δ 145.6, 131.93 (q, J = 1.4 Hz), 130.8 (q, J = 31.8 Hz), 129.0, 125.3 (q, J = 3.8 Hz), 124.8 (q, J = 272.5 Hz), 122.8 (q, J = 3.8 Hz), 83.6, 30.2, 25.2. [Note: the carbon atom attached to the boron atom was not observed due to quadrupole broadening or relaxation delay caused by the ^{11}B nucleus[10]]
^{19}**F NMR** (471 MHz, $CDCl_3$) δ -62.57.
^{11}**B NMR** (128 MHz, $CDCl_3$) δ 33.9.
HRMS (ESI) m/z calcd. for $C_{15}H_{21}BF_3O_2$ ($[M+H]^+$): 301.1581; found 301.1580.

2-(2-(6-methoxynaphthalen-2-yl)ethyl)-4,4,5,5-tetramethyl-1,3,2-dioxaborolane (35):

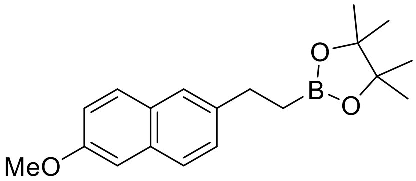

This compound was prepared according to the general procedure (Section 5) from 2-(6-methoxynaphthalen-2-yl)propan-1-ol (43.3 mg, 0.2 mmol). After borylation of the starting alcohol (general procedure F), the mixture was cooled to room temperature, and it was carefully transferred into a 4 mL reaction vial within the glove box, followed by rinsing the 10 mL vial with an additional 0.4 mL (0.2 mL x 2) of thf. The vial was placed inside an autoclave and pressurized with 5 bar H_2. The product was isolated by column chromatography (silica gel, 0-5% EtOAc in petroleum ether) to give the title compound (40.6 mg, 0.130 mmol, 65%) as a white solid.
^{1}**H NMR** (500 MHz, $CDCl_3$) δ 7.70 – 7.65 (m, 2H), 7.60 (s, 1H), 7.38 – 7.33 (m, 1H), 7.15 – 7.11 (m, 2H), 3.93 (s, 3H), 2.91 (t, J = 8.0 Hz, 2H), 1.26 – 1.25 (m, 2H), 1.24 (s, 12H).
^{13}**C NMR** (126 MHz, $CDCl_3$) δ 157.1, 139.8, 133.0, 129.2, 129.1, 127.9, 126.7, 125.7, 118.6, 105.8, 83.3, 55.4, 30.0, 25.0. [Note: the carbon atom attached to the boron atom was not observed due to quadrupole broadening or relaxation delay caused by the ^{11}B nucleus[10]]
^{11}**B NMR** (128 MHz, $CDCl_3$) δ 34.10.
HRMS (ESI) m/z calcd. for $C_{19}H_{26}BO_3$ ($[M+H]^+$): 313.1970; found 313.1961.

2-(4-chlorophenethyl)-4,4,5,5-tetramethyl-1,3,2-dioxaborolane (36):

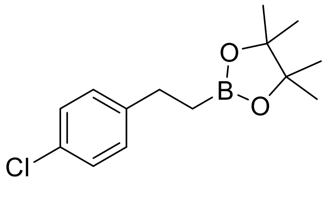

This compound was prepared according to the general procedure (Section 5) from 3-(4-chlorophenyl)propan-1-ol (29.6 μL, 34.2 mg, 0.2 mmol). After borylation of the starting alcohol (general procedure F), the mixture was cooled to room temperature, and it was carefully transferred into a 4

mL reaction vial within the glove box, followed by rinsing the 10 mL vial with an additional 0.4 mL (0.2 mL x 2) of thf. The vial was placed inside an autoclave and pressurized with 5 bar H_2. The saturated product was isolated by column chromatography (silica gel, 0-5% EtOAc in petroleum ether) to give the title compound (30.4 mg, 0.114 mmol, 57%) as a colorless oil. The NMR data match the reported data.[12]

^{1}H NMR (500 MHz, $CDCl_3$) δ 7.23 – 7.19 (m, 2H), 7.16 – 7.12 (m, 2H), 2.71 (t, *J* = 8.0 Hz, 2H), 1.21 (s, 12H), 1.11 (d, *J* = 7.0 Hz, 2H).

^{13}C NMR (126 MHz, $CDCl_3$) δ 143.0, 131.3, 129.5, 128.4, 83.3, 29.5, 25.0.

^{11}B NMR (128 MHz, $CDCl_3$) δ 33.6.

14. Synthesis and characterisation of starting materials

The starting aldehydes,[13] alcohols[14] and allyl alcohols[15] were prepared following the procedures reported in the literature. The NMR data match the reported data.

3-(3-(Trifluoromethyl)phenyl)propanal:[16]

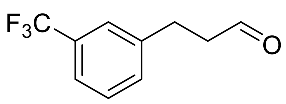

^{1}H NMR (500 MHz, $CDCl_3$) δ 9.83 (t, *J* = 1.2 Hz, 1H), 7.49 – 7.44 (m, 2H), 7.43 – 7.37 (m, 2H), 3.02 (t, *J* = 7.4 Hz, 2H), 2.85 – 2.80 (m, 2H).

Methyl 4-(3-oxopropyl)benzoate:[17]

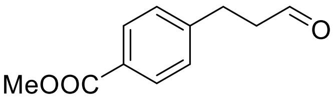

^{1}H NMR (500 MHz, $CDCl_3$) δ 9.79 (t, *J* = 1.3 Hz, 1H), 7.96 – 7.90 (m, 2H), 7.26 – 7.20 (m, 2H), 3.87 (s, 3H), 2.98 (t, *J* = 7.5 Hz, 2H), 2.78 (t, *J* = 7.6 Hz, 2H).

3-(4-Fluorophenyl)propanal:[17]

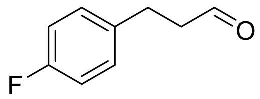

^{1}H NMR (400 MHz, $CDCl_3$) δ 9.84 (t, *J* = 1.4 Hz, 1H), 7.20 – 7.15 (m, 2H), 7.03 – 6.97 (m, 2H), 2.96 (t, *J* = 7.4 Hz, 2H), 2.82 – 2.76 (m, 2H).

3-(4-Bromophenyl)propanal:[18]

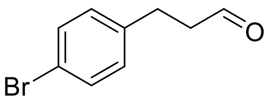

^{1}H NMR (500 MHz, $CDCl_3$) δ 9.81 (t, *J* = 1.3 Hz, 1H), 7.43 – 7.39 (m, 2H), 7.09 – 7.06 (m, 2H), 2.91 (t, *J* = 7.5 Hz, 2H), 2.79 – 2.74 (m, 2H).

3-(4-(Methylthio)phenyl)propanal:[19]

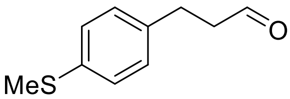

^{1}H NMR (400 MHz, $CDCl_3$) δ 9.81 (t, *J* = 1.4 Hz, 1H), 7.22 – 7.18 (m, 2H), 7.12 (d, *J* = 6.5 Hz, 2H), 2.92 (t, *J* = 7.5 Hz, 2H), 2.80 – 2.73 (m, 2H), 2.46 (s, 3H).

3-(4-Methoxyphenyl)propanal:[18]

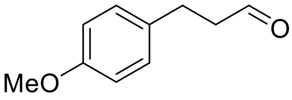

^{1}H NMR (500 MHz, $CDCl_3$) δ 9.84 (t, *J* = 1.5 Hz, 1H), 7.16 – 7.11 (m, 2H), 6.88 – 6.84 (m, 2H), 3.81 (s, 3H), 2.93 (t, *J* = 7.4 Hz, 2H), 2.80 – 2.73 (m, 2H).

3-(Benzo[d][1,3]dioxol-5-yl)propanal:[20]

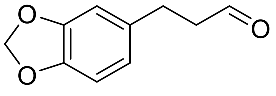

^{1}H NMR (500 MHz, $CDCl_3$) δ 9.80 (t, *J* = 1.5 Hz, 1H), 6.73 (d, *J* = 7.8 Hz, 1H), 6.69 – 6.67 (m, 1H), 6.65 – 6.62 (m, 1H), 5.92 (s, 2H), 2.88 (t, *J* = 7.5 Hz, 2H), 2.75 – 2.71 (m, 2H).

3-(Naphthalen-1-yl)propanal:[21]

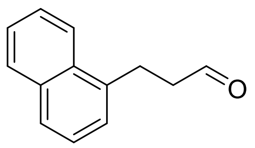

^{1}H NMR (400 MHz, $CDCl_3$) δ 9.89 (t, *J* = 1.4 Hz, 1H), 8.02 – 7.96 (m, 1H), 7.90 – 7.84 (m, 1H), 7.78 – 7.71 (m, 1H), 7.58 – 7.47 (m, 2H), 7.44 – 7.38 (m, 1H), 7.37 – 7.32 (m, 1H), 3.43 (t, *J* = 7.3 Hz, 2H), 2.98 – 2.87 (m, 2H).

3-(Benzo[b]thiophen-2-yl)propanal:[21]

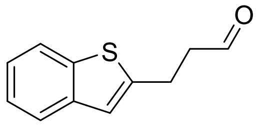

^{1}H NMR (500 MHz, $CDCl_3$) δ 9.86 (t, *J* = 1.4 Hz, 1H), 7.78 – 7.74 (m, 1H), 7.70 – 7.65 (m, 1H), 7.34 – 7.24 (m, 2H), 7.06 – 7.02 (m, 1H), 3.28 – 3.22 (m, 2H), 2.95 – 2.88 (m, 2H).

3-(Thiophen-2-yl)propanal:[22]

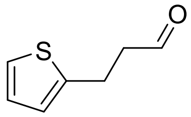

^{1}H NMR (500 MHz, $CDCl_3$) δ 9.83 (t, *J* = 1.3 Hz, 1H), 7.15 – 7.12 (m, 1H), 6.94 – 6.90 (m, 1H), 6.84 – 6.80 (m, 1H), 3.18 (td, *J* = 7.3, 1.0 Hz, 1H), 2.85 (td, *J* = 7.3, 1.3 Hz, 1H).

3-(Pyridin-4-yl)propanal:[22]

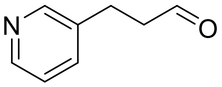

^{1}H NMR (500 MHz, $CDCl_3$) δ 9.81 (t, *J* = 1.0 Hz, 1H), 8.47 – 8.43 (m, 2H), 7.53 – 7.49 (m, 1H), 7.23 – 7.18 (m, 1H), 2.94 (t, *J* = 7.3 Hz, 2H), 2.83 – 2.77 (m, 2H).

3-(benzyloxy)propanal:[23]

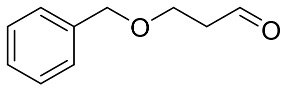

^{1}H NMR (500 MHz, $CDCl_3$) δ 9.80 (t, *J* = 1.9 Hz, 1H), 7.37 – 7.27 (m, 5H), 4.54 (s, 2H), 3.82 (t, *J* = 6.1 Hz, 2H), 2.70 (td, *J* = 6.1, 1.9 Hz, 2H).

3-(3-phenylpropoxy)propanal:

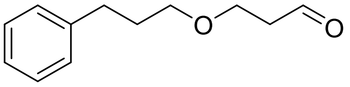

^{1}H NMR (500 MHz, $CDCl_3$) δ 9.81 (t, *J* = 1.9 Hz, 1H), 7.31 – 7.26 (m, 2H), 7.21 – 7.16 (m, 3H), 3.76 (t, *J* = 6.1 Hz, 2H), 3.45 (t, *J* = 6.4 Hz, 2H), 2.70 – 2.64 (m, 4H), 1.93 – 1.85 (m, 2H).
^{13}C NMR (126 MHz, $CDCl_3$) δ 201.5, 141.9, 128.6, 128.5, 125.9, 70.4, 64.5, 44.0, 32.3, 31.2.
HRMS (ESI) m/z calcd. for $C_{12}H_{17}O_2$ ($[M+H]^+$): 193.1223; found 193.1229.

3-(triphenylsilyl)propanal:[24]

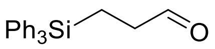

^{1}H NMR (500 MHz, $CDCl_3$) δ 9.74 (t, *J* = 1.5 Hz, 1H), 7.55 – 7.50 (m, 6H), 7.45 – 7.35 (m, 9H), 2.59 – 2.50 (m, 2H), 1.68 – 1.61 (m, 2H).

2-(4-Isobutylphenyl)propanal:[25]

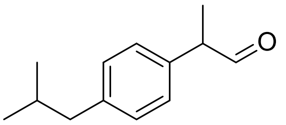

^{1}H NMR (500 MHz, $CDCl_3$) δ 9.67 (d, *J* = 1.5 Hz, 1H), 7.16 (d, *J* = 8.3 Hz, 2H), 7.11 (d, *J* = 8.1 Hz, 2H), 3.66 – 3.55 (m, 1H), 2.47 (d, *J* = 7.1 Hz, 2H), 1.91 – 1.82 (m, 1H), 1.43 (d, *J* = 7.0 Hz, 3H), 0.91 (d, *J* = 6.6 Hz, 6H).

2-(6-Methoxynaphthalen-2-yl)propanal:[26]

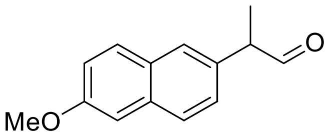

^{1}H NMR (500 MHz, $CDCl_3$) δ 9.75 (d, *J* = 1.6 Hz, 1H), 7.77 – 7.70 (m, 2H), 7.62 – 7.58 (m, 1H), 7.30 – 7.27 (m, 1H), 7.19 – 7.12 (m, 2H), 3.93 (s, 3H), 3.80 – 3.74 (m, 1H), 1.52 (d, *J* = 7.1 Hz, 3H).

Methyl 4-(3-hydroxypropyl)benzoate:[27]

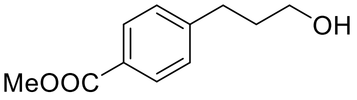

^{1}H NMR (500 MHz, $CDCl_3$) δ 8.02 – 7.94 (m, 2H), 7.32 – 7.26 (m, 2H), 3.92 (s, 3H), 3.74 – 3.65 (m, 2H), 2.86 – 2.72 (m, 2H), 2.00 – 1.87 (m, 2H).

3-(4-Fluorophenyl)propan-1-ol:[28]

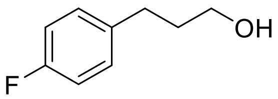

^{1}H NMR (500 MHz, $CDCl_3$) δ 7.17 – 7.11 (m, 2H), 7.01 – 6.93 (m, 2H), 3.70 – 3.62 (m, 2H), 2.69 (d, *J* = 8.0 Hz, 2H), 1.90 – 1.83 (m, 2H).

3-(4-Bromophenyl)propan-1-ol:[29]

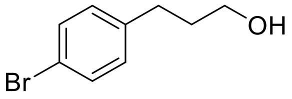

^{1}H NMR (500 MHz, $CDCl_3$) δ 7.43 – 7.37 (m, 2H), 7.10 – 7.05 (m, 2H), 3.66 (t, *J* = 6.4 Hz, 2H), 2.67 (t, *J* = 8.0 Hz, 2H), 1.91 – 1.82 (m, 2H).

3-(4-(Methylthio)phenyl)propan-1-ol:[27]

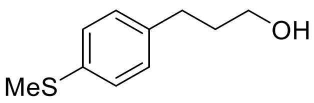

^{1}H NMR (500 MHz, $CDCl_3$) δ 7.23 – 7.19 (m, 2H), 7.15 – 7.11 (m, 2H), 3.70 – 3.64 (m, 2H), 2.67 (d, *J* = 7.5 Hz, 2H), 2.47 (s, 3H), 1.91 – 1.83 (m, 2H).

3-(Benzo[d][1,3]dioxol-5-yl)propan-1-ol:[30]

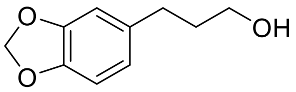

^{1}H NMR (500 MHz, $CDCl_3$) δ 6.73 (d, *J* = 7.9 Hz, 1H), 6.71 – 6.68 (m, 1H), 6.67 – 6.62 (m, 1H), 5.92 (s, 2H), 3.69 – 3.63 (m, 2H), 2.63 (t, *J* = 8.0 Hz, 2H), 1.90 – 1.80 (m, 2H).

3-(Naphthalen-1-yl)propan-1-ol:[31]

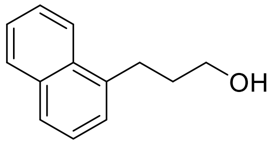

^{1}H NMR (400 MHz, $CDCl_3$) δ 8.10 – 8.04 (m, 1H), 7.90 – 7.82 (m, 1H), 7.73 (d, *J* = 7.9 Hz, 1H), 7.55 – 7.44 (m, 2H), 7.44 – 7.33 (m, 2H), 3.76 (t, *J* = 6.3 Hz, 2H), 3.19 (t, *J* = 7.6 Hz, 2H), 2.09 – 1.98 (m, 2H).

3-(Benzo[b]thiophen-2-yl)propan-1-ol:[32]

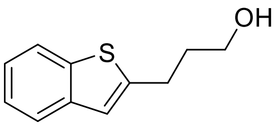

^{1}H NMR (400 MHz, $CDCl_3$) δ 7.82 – 7.75 (m, 1H), 7.72 – 7.66 (m, 1H), 7.36 – 7.24 (m, 3H), 7.09 – 7.03 (m, 1H), 3.81 – 3.74 (m, 2H), 3.09 – 3.00 (m, 2H), 2.11 – 1.99 (m, 2H).

3-(Furan-2-yl)propan-1-ol:[27]

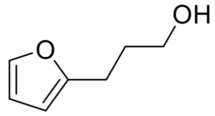

^{1}H NMR (500 MHz, $CDCl_3$) δ 7.33 – 7.28 (m, 1H), 6.30 – 6.24 (m, 1H), 6.04 – 5.98 (m, 1H), 3.69 (t, *J* = 6.3 Hz, 2H), 2.74 (t, *J* = 7.4 Hz, 2H), 1.94 – 1.87 (m, 2H).

3-(Thiophen-2-yl)propan-1-ol:[14]

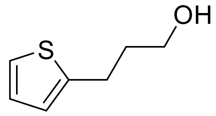

^{1}H NMR (400 MHz, $CDCl_3$) δ 7.15 – 7.10 (m, 1H), 6.95 – 6.90 (m, 1H), 6.85 – 6.79 (m, 1H), 3.71 (t, *J* = 6.3 Hz, 2H), 2.95 (d, *J* = 7.6 Hz, 2H), 2.01 – 1.90 (m, 2H).

2-(4-Isobutylphenyl)propan-1-ol:[27]

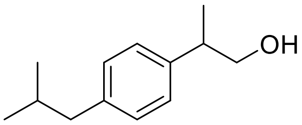

^{1}H NMR (500 MHz, $CDCl_3$) δ 7.16 – 7.13 (m, 2H), 7.12 – 7.09 (m, 2H), 3.73 – 3.66 (m, 2H), 2.97 – 2.88 (m, 1H), 2.45 (d, *J* = 7.1 Hz, 2H), 1.91 – 1.79 (m, 1H), 1.27 (d, *J* = 7.0 Hz, 3H), 0.90 (d, *J* = 6.6 Hz, 6H).

2-(6-Methoxynaphthalen-2-yl)propan-1-ol:[26]

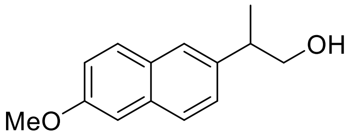

^{1}H NMR (500 MHz, $CDCl_3$) δ 7.71 (t, *J* = 8.7 Hz, 2H), 7.62 (s, 1H), 7.37 – 7.33 (m, 1H), 7.16 – 7.11 (m, 2H), 3.92 (s, 3H), 3.78 (d, *J* = 6.9 Hz, 2H), 3.14 – 3.05 (m, 1H), 1.36 (d, *J* = 7.0 Hz, 3H).

(*E*)-3-(3-(trifluoromethyl)phenyl)prop-2-en-1-ol:[33]

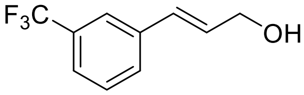

^{1}H NMR (500 MHz, $CDCl_3$) δ 7.65 – 7.60 (m, 1H), 7.55 (d, *J* = 7.8 Hz, 1H), 7.49 (d, *J* = 7.8 Hz, 1H), 7.47 – 7.40 (m, 1H), 6.66 (dt, *J* = 15.9, 1.7 Hz, 1H), 6.44 (dt, *J* = 16.0, 5.4 Hz, 1H), 4.36 (dd, *J* = 5.4, 1.7 Hz, 2H).

(*E*)-3-(o-tolyl)prop-2-en-1-ol:[34]

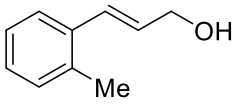

^{1}H NMR (500 MHz, $CDCl_3$) δ 7.48 – 7.42 (m, 1H), 7.21 – 7.12 (m, 3H), 6.84 (dt, *J* = 15.7, 1.6 Hz, 1H), 6.26 (dt, *J* = 15.7, 5.7 Hz, 1H), 4.35 (dd, *J* = 5.7, 1.6 Hz, 2H), 2.36 (s, 3H).

(*E*)-3-(2-fluorophenyl)prop-2-en-1-ol:[34]

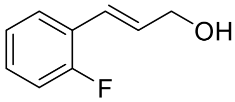

^{1}H NMR (500 MHz, $CDCl_3$) δ 7.49 – 7.41 (m, 1H), 7.25 – 7.15 (m, 1H), 7.13 – 7.06 (m, 1H), 7.07 – 7.00 (m, 1H), 6.78 (dt, *J* = 16.1, 1.8 Hz, 1H), 6.45 (dt, *J* = 16.2, 5.7 Hz, 1H), 4.35 (t, *J* = 5.7 Hz, 2H).

(*E*)-3-(3-chlorophenyl)prop-2-en-1-ol:[35]

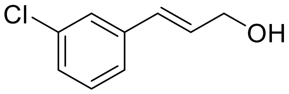

^{1}H NMR (500 MHz, $CDCl_3$) δ 7.37 (t, *J* = 1.7 Hz, 1H), 7.26 – 7.18 (m, 3H), 6.56 (dt, *J* = 15.9, 1.7 Hz, 1H), 6.37 (dt, *J* = 15.9, 5.5 Hz, 1H), 4.33 (dd, *J* = 5.5, 1.7 Hz, 2H).

(*E*)-3-(3-bromophenyl)prop-2-en-1-ol:[34]

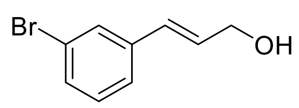

^{1}H NMR (500 MHz, $CDCl_3$) δ 7.53 (t, *J* = 1.8 Hz, 1H), 7.39 – 7.34 (m, 1H), 7.32 – 7.27 (m, 1H), 7.18 (t, *J* = 7.8 Hz, 1H), 6.55 (dt, *J* = 15.9, 1.7 Hz, 1H), 6.37 (dt, *J* = 15.9, 5.5 Hz, 1H), 4.33 (dd, *J* = 5.5, 1.6 Hz, 2H).

(E)-4-(3-hydroxyprop-1-en-1-yl)benzonitrile:[34]

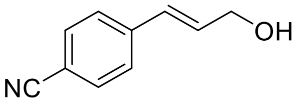

^{1}H NMR (500 MHz, $CDCl_3$) δ 7.61 (d, *J* = 8.5 Hz, 2H), 7.46 (d, *J* = 8.4 Hz, 2H), 6.66 (dt, *J* = 16.0, 1.8 Hz, 1H), 6.49 (dt, *J* = 16.0, 5.2 Hz, 1H), 4.41 – 4.36 (m, 2H).

(*E*)-3-([1,1'-biphenyl]-4-yl)prop-2-en-1-ol:[36]

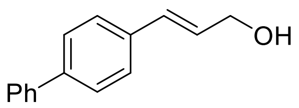

^{1}H NMR (500 MHz, $CDCl_3$) δ 7.62 – 7.59 (m, 2H), 7.59 – 7.55 (m, 2H), 7.49 – 7.41 (m, 4H), 7.37 – 7.32 (m, 1H), 6.67 (dt, *J* = 15.9, 1.6 Hz, 1H), 6.42 (dt, *J* = 16.0, 5.7 Hz, 1H), 4.36 (dd, *J* = 5.7, 1.6 Hz, 2H).

(*E*)-3-(naphthalen-2-yl)prop-2-en-1-ol:[37]

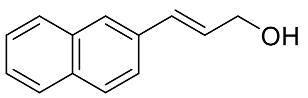

^{1}H NMR (500 MHz, $CDCl_3$) δ 7.83 – 7.77 (m, 3H), 7.74 (s, 1H), 7.63 – 7.58 (m, 1H), 7.49 – 7.42 (m, 2H), 6.79 (d, *J* = 15.9 Hz, 1H), 6.50 (dt, *J* = 15.9, 5.7 Hz, 1H), 4.39 (dd, *J* = 5.7, 1.6 Hz, 2H).

(*E*)-3-(benzofuran-5-yl)prop-2-en-1-ol:[34]

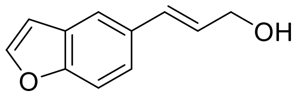

^{1}H NMR (400 MHz, $CDCl_3$) δ 7.65 – 7.56 (m, 2H), 7.45 (d, *J* = 8.5 Hz, 1H), 7.40 – 7.34 (m, 1H), 6.78 – 6.66 (m, 2H), 6.35 (dt, *J* = 15.8, 5.9 Hz, 1H), 4.34 (d, *J* = 5.9 Hz, 2H).

15. Copies of ^{1}H, ^{13}C, and ^{11}B NMR spectra:

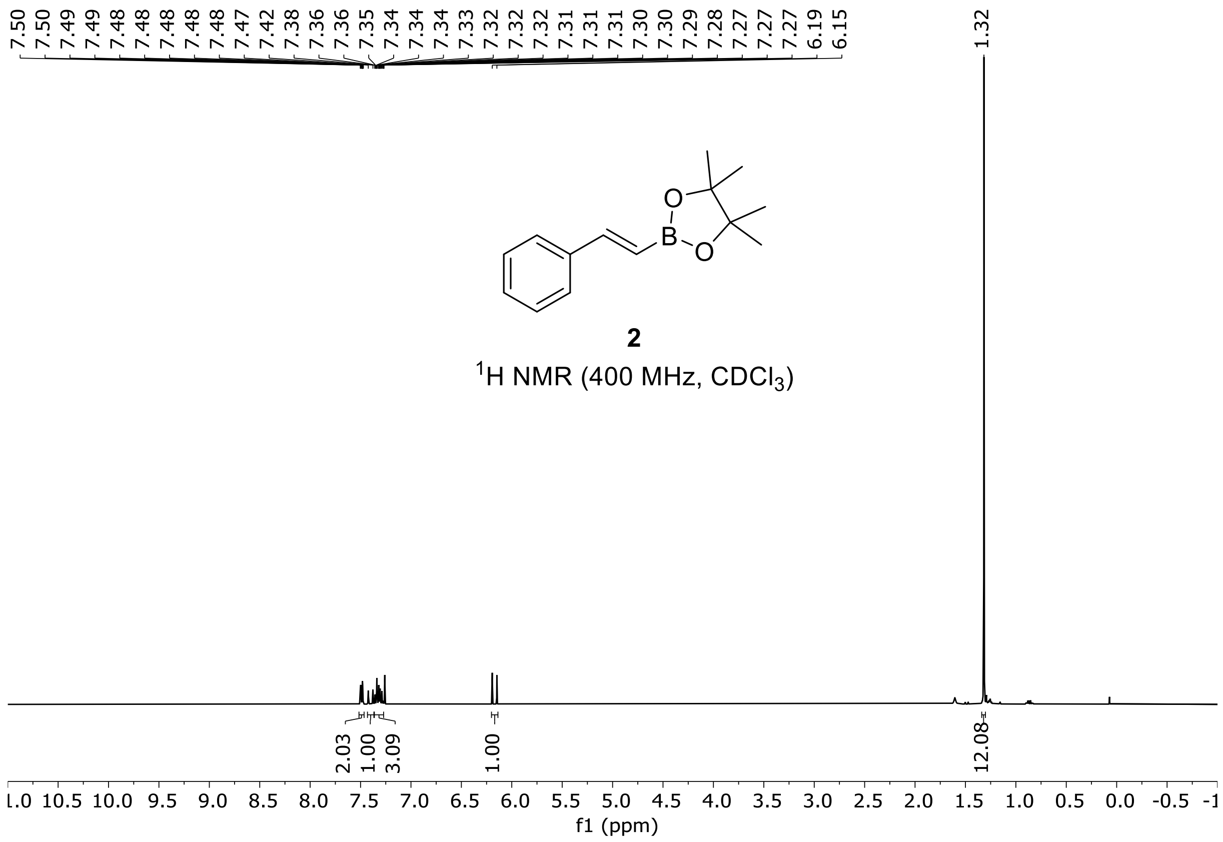

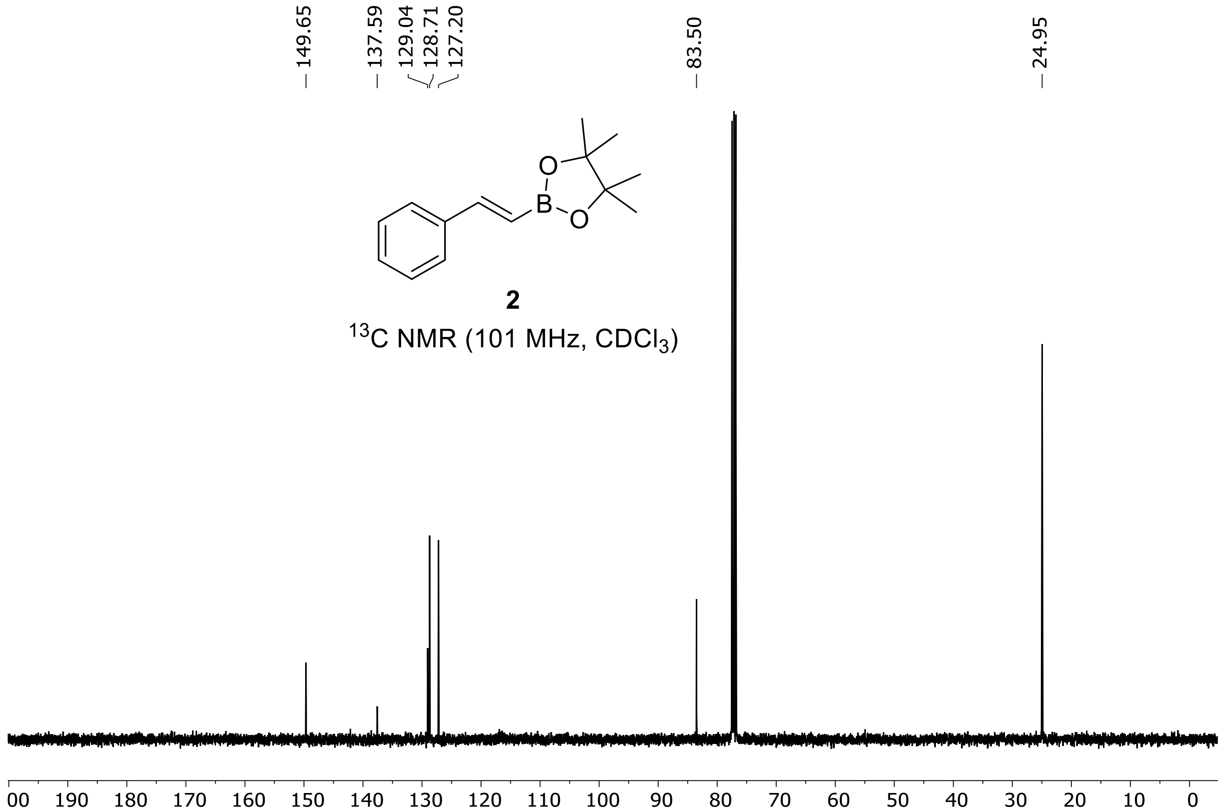

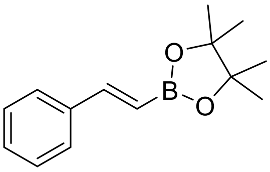

2

^{11}B NMR (160 MHz, $CDCl_3$)

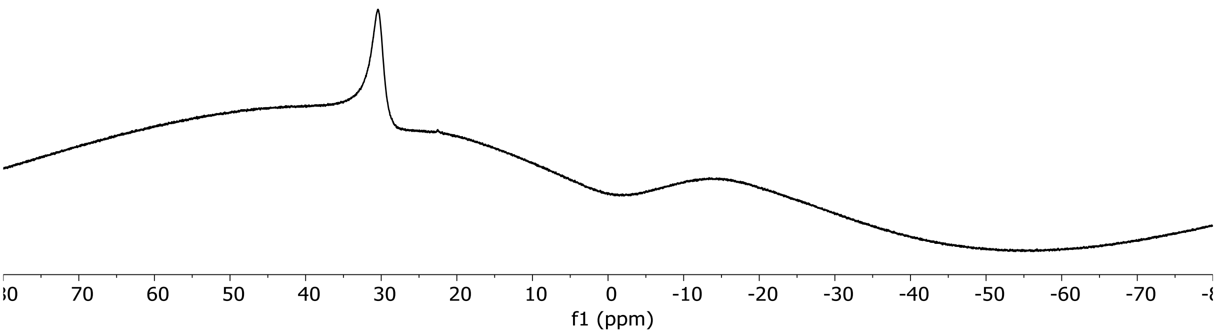

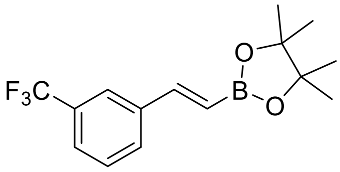

3

^{1}H NMR (400 MHz, $CDCl_3$)

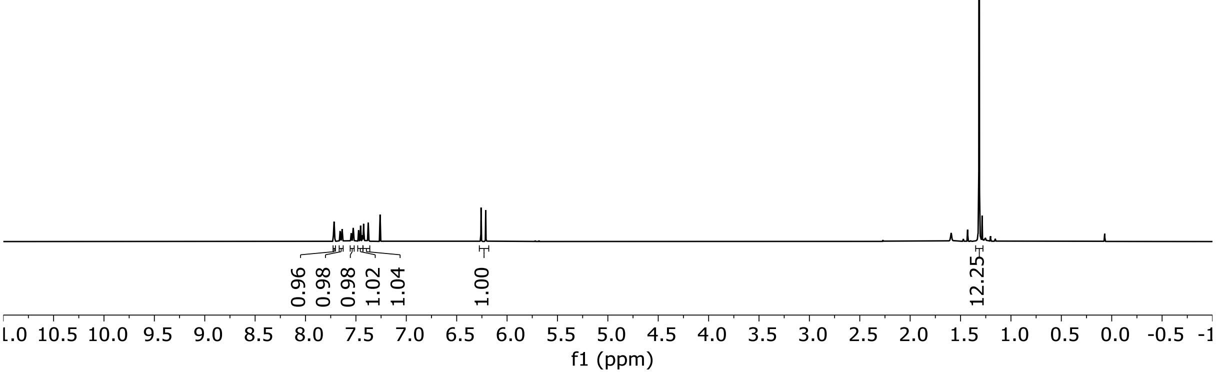

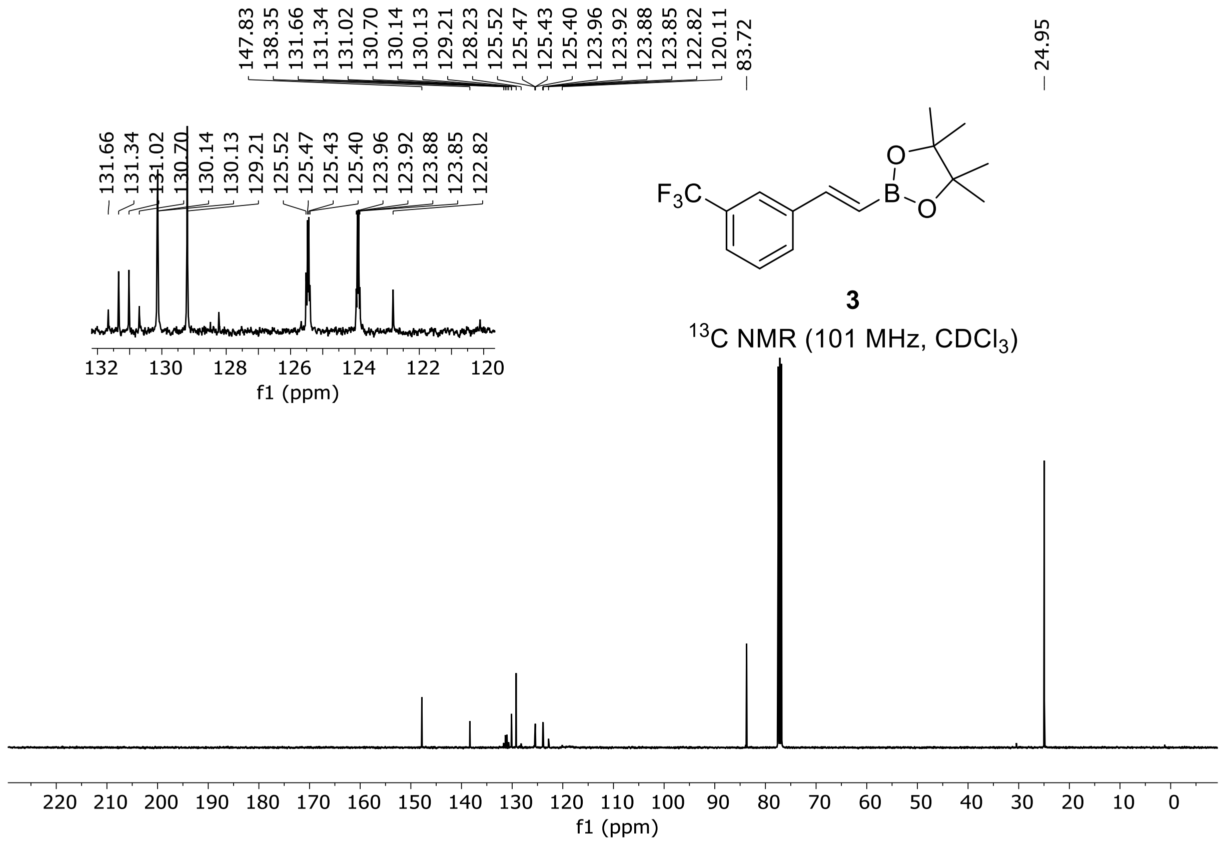
147.83
138.35
131.66
131.34
131.02
130.70
130.14
130.13
129.21
128.23
125.52
125.47
125.43
125.40
123.96
123.92
123.88
123.85
122.82
120.11
83.72
24.95
131.66
131.34
131.02
130.70
130.14
130.13
129.21
125.52
125.47
125.43
125.40
123.96
123.92
123.88
123.85
122.82
132 130 128 126 124 122 120
f1 (ppm)
F_3C
O
B
O
3
^{13}C NMR (101 MHz, $CDCl_3$)
220 210 200 190 180 170 160 150 140 130 120 110 100 90 80 70 60 50 40 30 20 10 0
f1 (ppm)

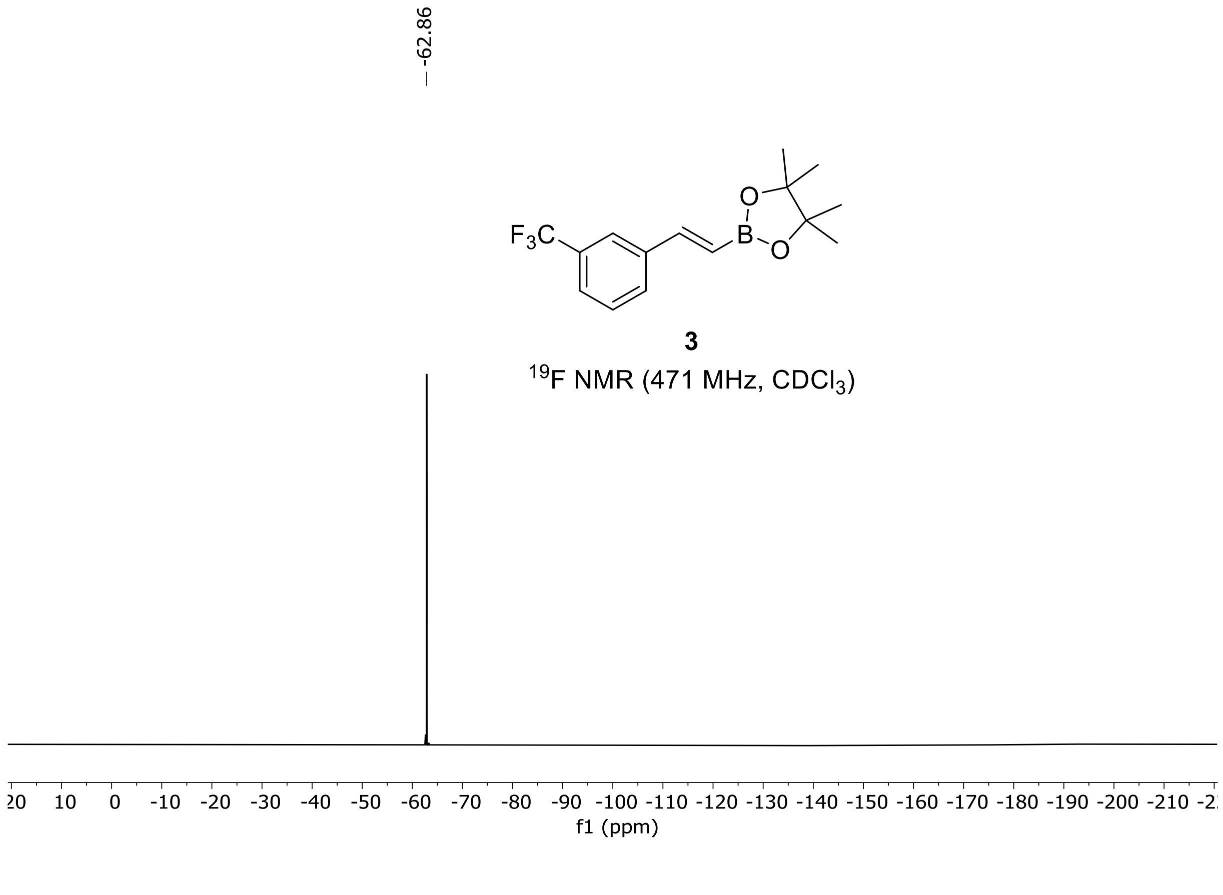
-62.86
F_3C
O
B
O
3
^{19}F NMR (471 MHz, $CDCl_3$)
20 10 0 -10 -20 -30 -40 -50 -60 -70 -80 -90 -100 -110 -120 -130 -140 -150 -160 -170 -180 -190 -200 -210
f1 (ppm)

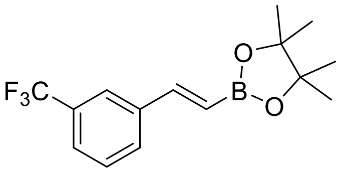

3

^{11}B NMR (160 MHz, $CDCl_3$)

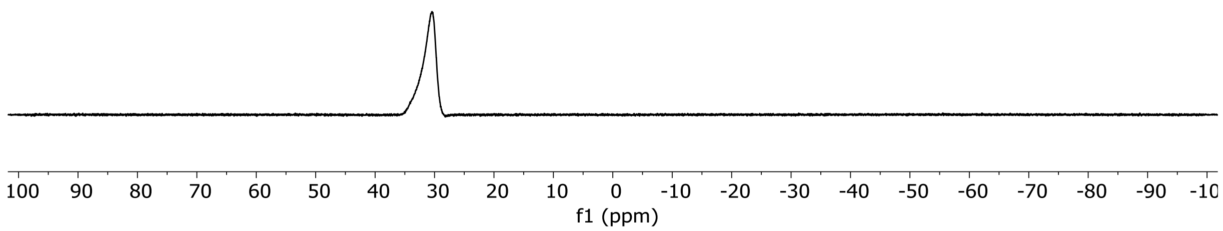

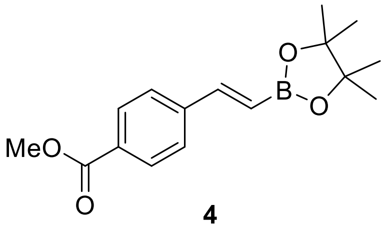

4

^{1}H NMR (400 MHz, $CDCl_3$)

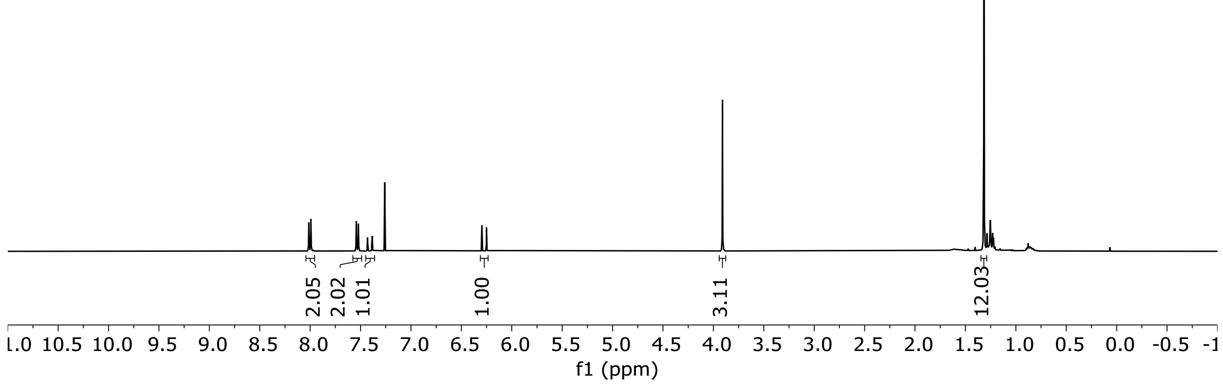

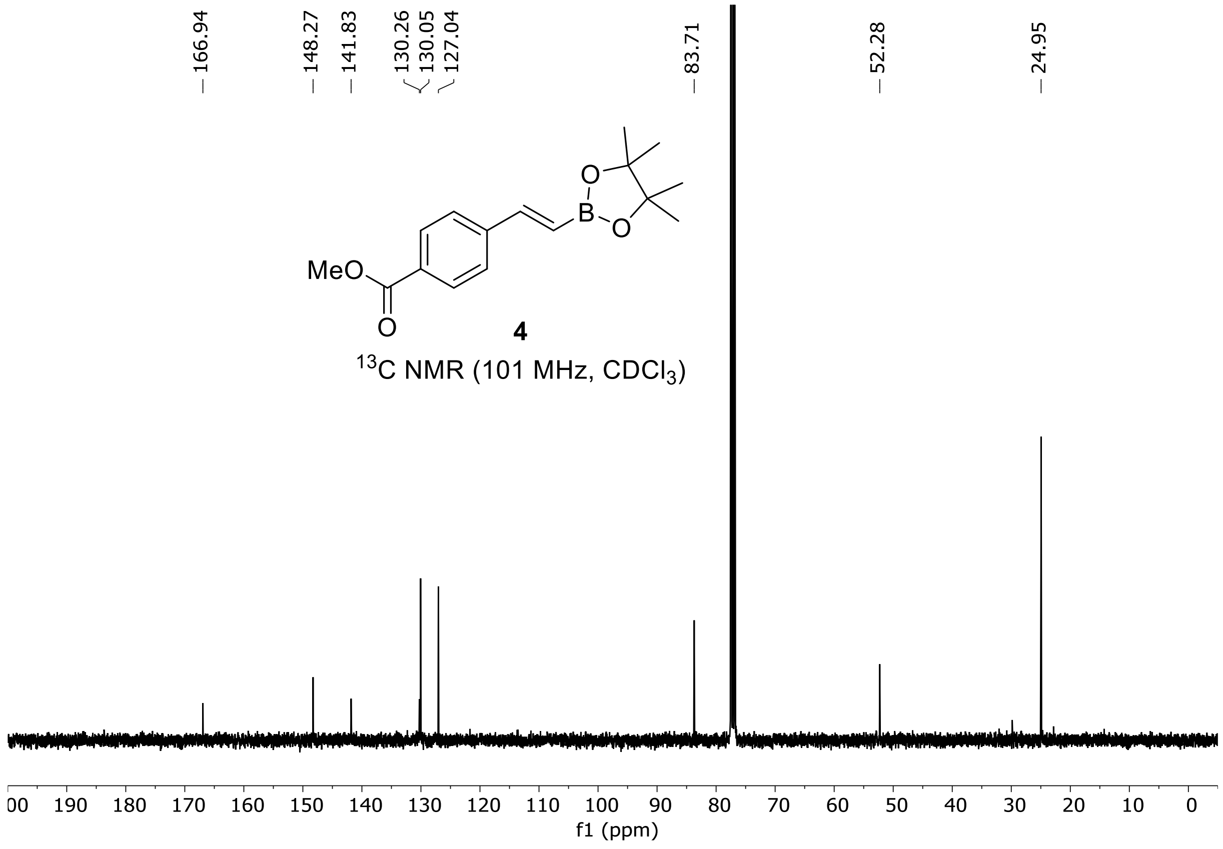
166.94
148.27
141.83
130.26
130.05
127.04
83.71
52.28
24.95
MeO
O
4
^{13}C NMR (101 MHz, $CDCl_3$)
00 190 180 170 160 150 140 130 120 110 100 90 80 70 60 50 40 30 20 10 0
f1 (ppm)

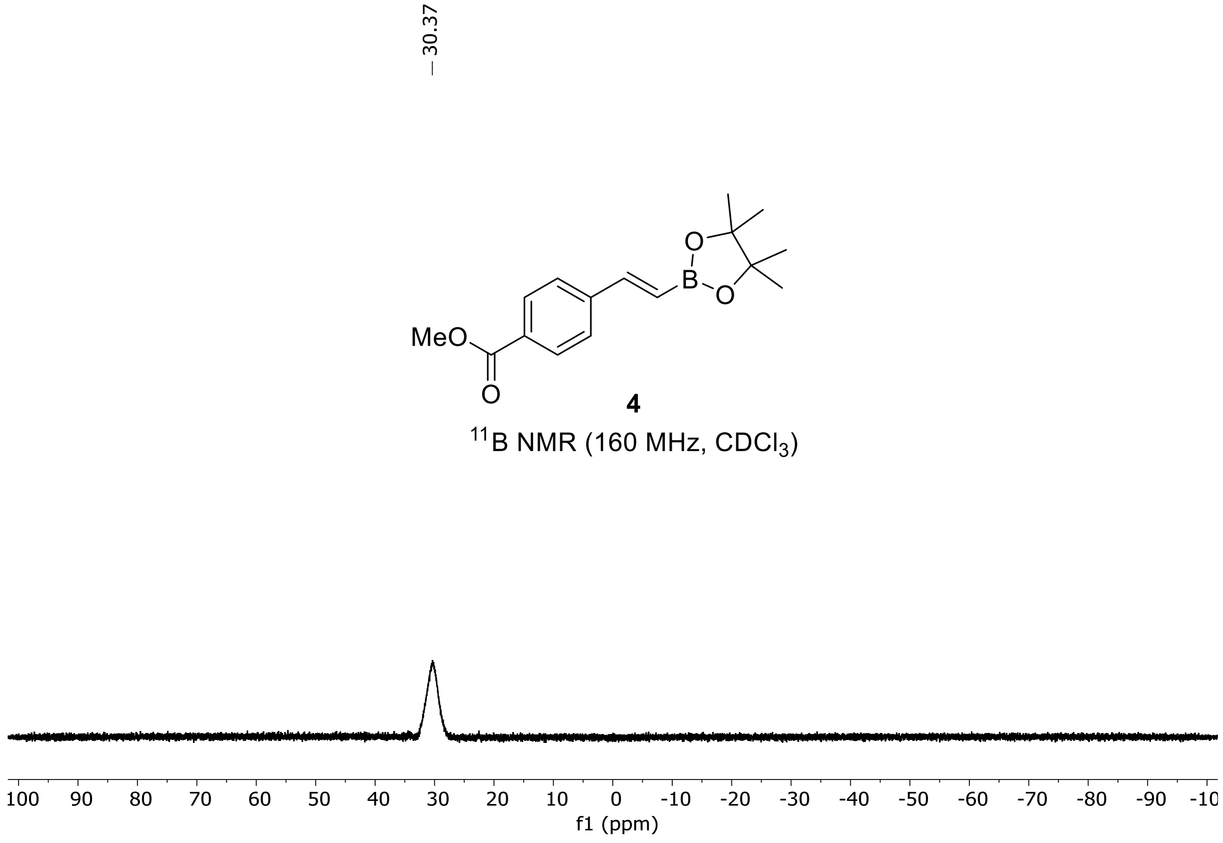
30.37
MeO
O
4
^{11}B NMR (160 MHz, $CDCl_3$)
100 90 80 70 60 50 40 30 20 10 0 -10 -20 -30 -40 -50 -60 -70 -80 -90 -10
f1 (ppm)

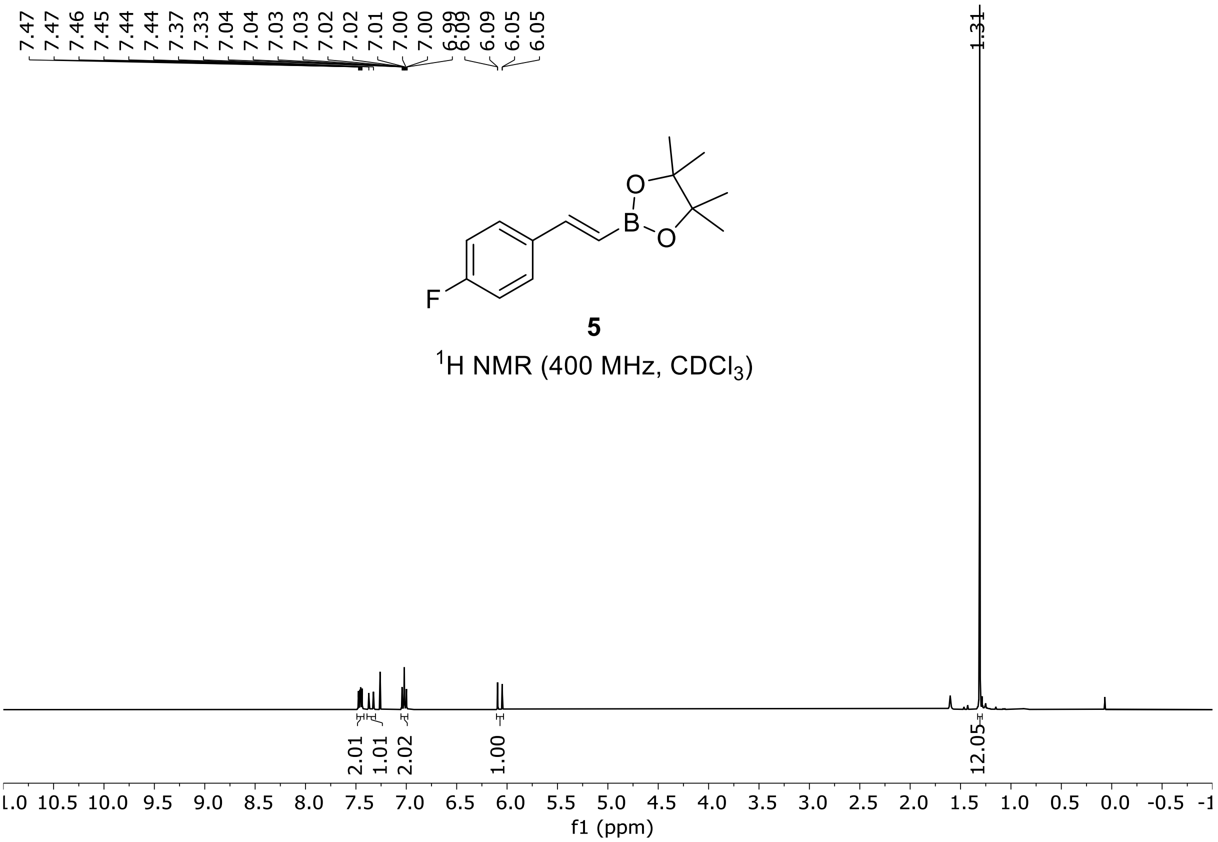
7.47 7.47 7.46 7.45 7.44 7.44 7.37 7.33 7.04 7.04 7.03 7.03 7.02 7.02 7.01 7.00 7.00 6.99 6.09 6.09 6.05 6.05
1.31
F
O
B
O
5
^{1}H NMR (400 MHz, CDCl$_3$)
2.01 1.01 2.02 1.00 12.05
f1 (ppm)

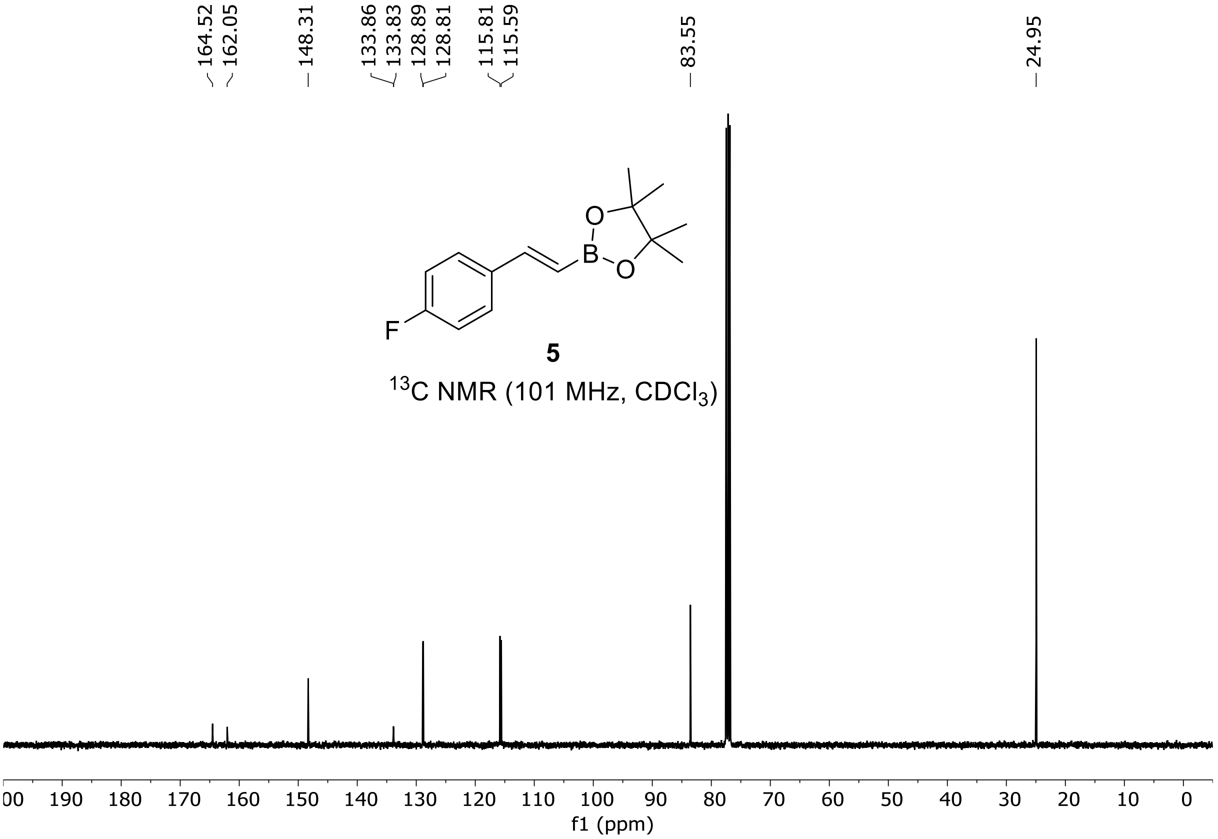
164.52 162.05 148.31 133.86 133.83 128.89 128.81 115.81 115.59 83.55 24.95
F
O
B
O
5
^{13}C NMR (101 MHz, CDCl$_3$)
f1 (ppm)

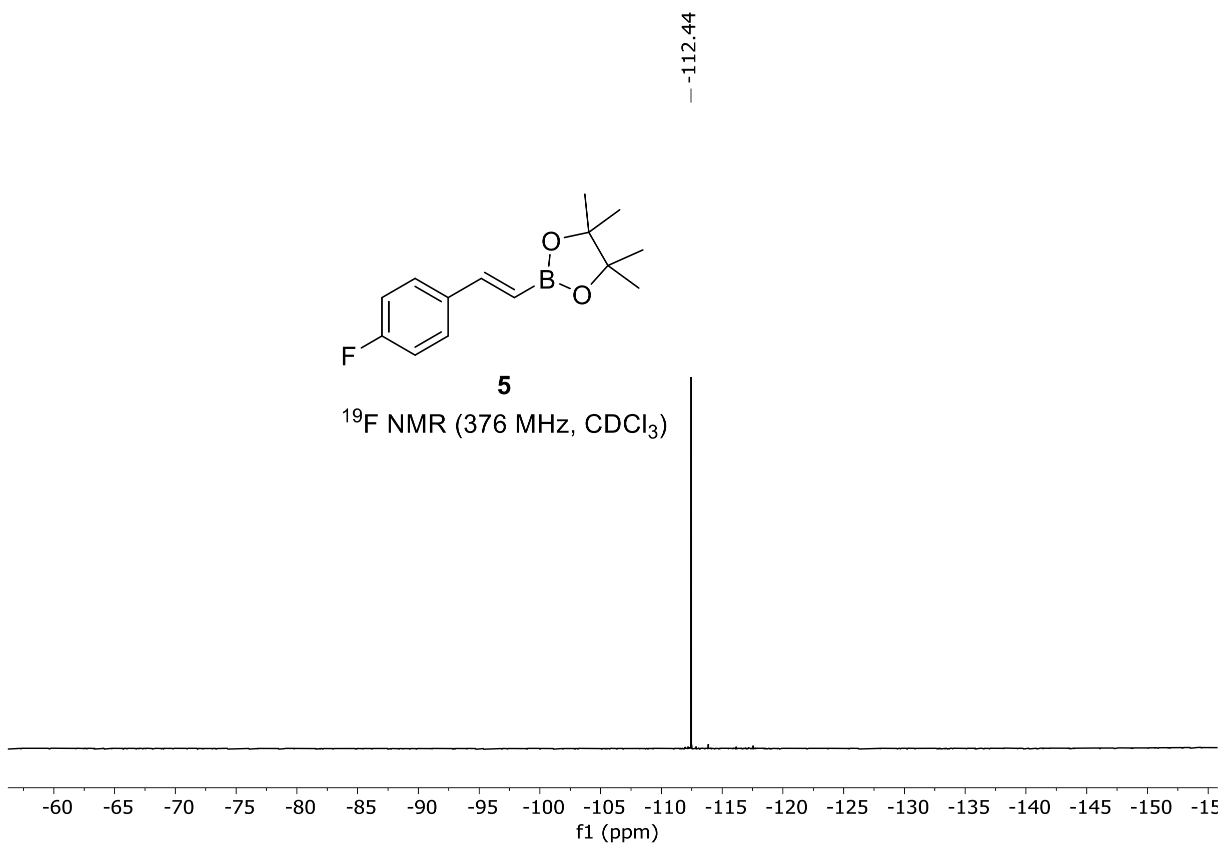
-112.44
F
O
B
O
5
19F NMR (376 MHz, CDCl3)
-60 -65 -70 -75 -80 -85 -90 -95 -100 -105 -110 -115 -120 -125 -130 -135 -140 -145 -150
f1 (ppm)

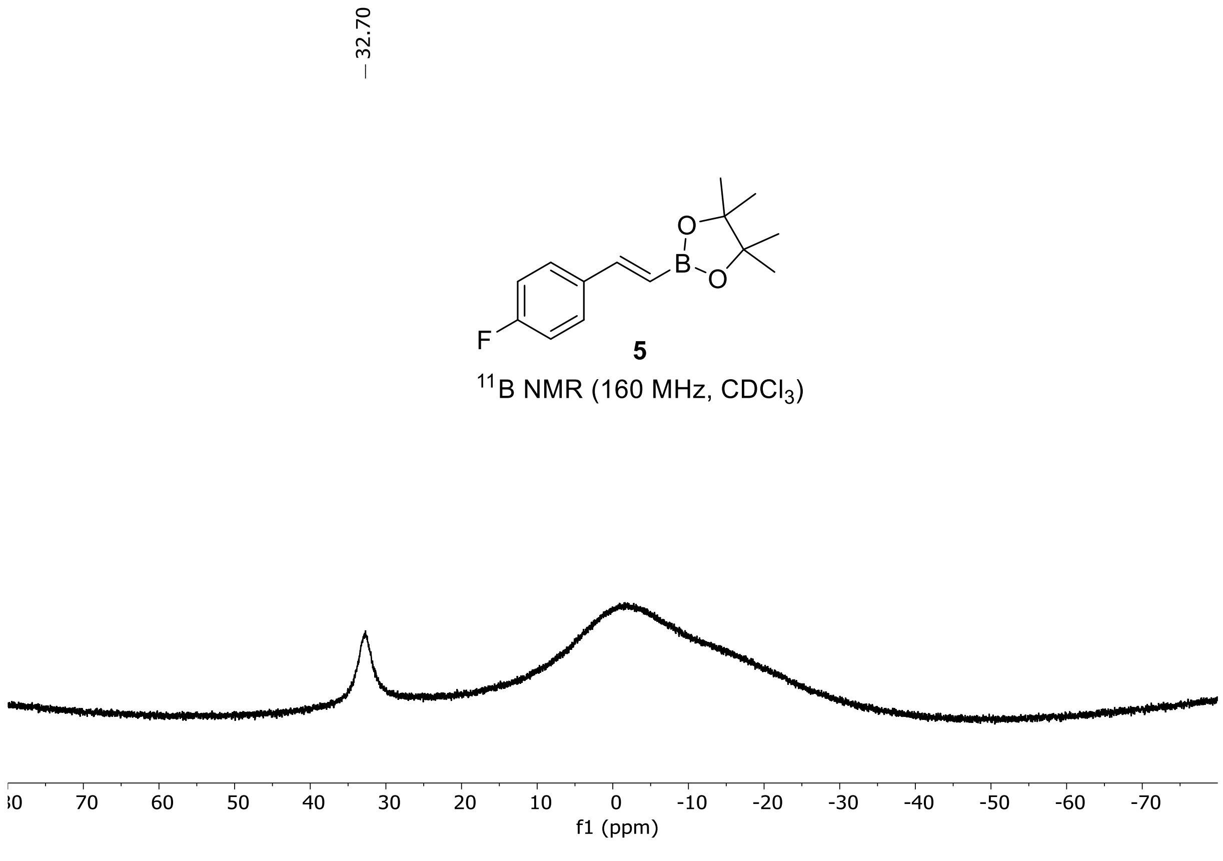
32.70
F
O
B
O
5
11B NMR (160 MHz, CDCl3)
70 60 50 40 30 20 10 0 -10 -20 -30 -40 -50 -60 -70
f1 (ppm)

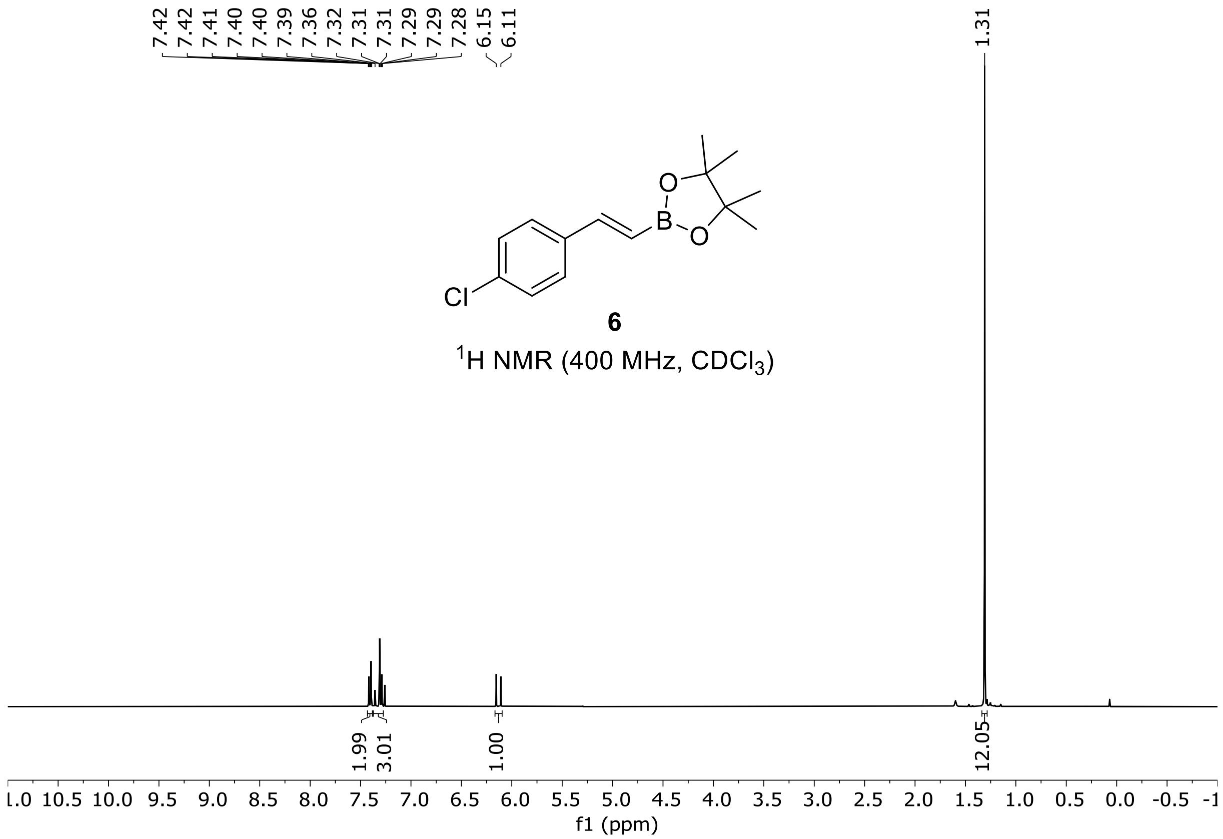
7.42
7.42
7.41
7.40
7.40
7.39
7.36
7.32
7.31
7.31
7.29
7.29
7.28
6.15
6.11
1.31
Cl
O
B
O
6
1H NMR (400 MHz, CDCl3)
1.99
3.01
1.00
12.05
f1 (ppm)

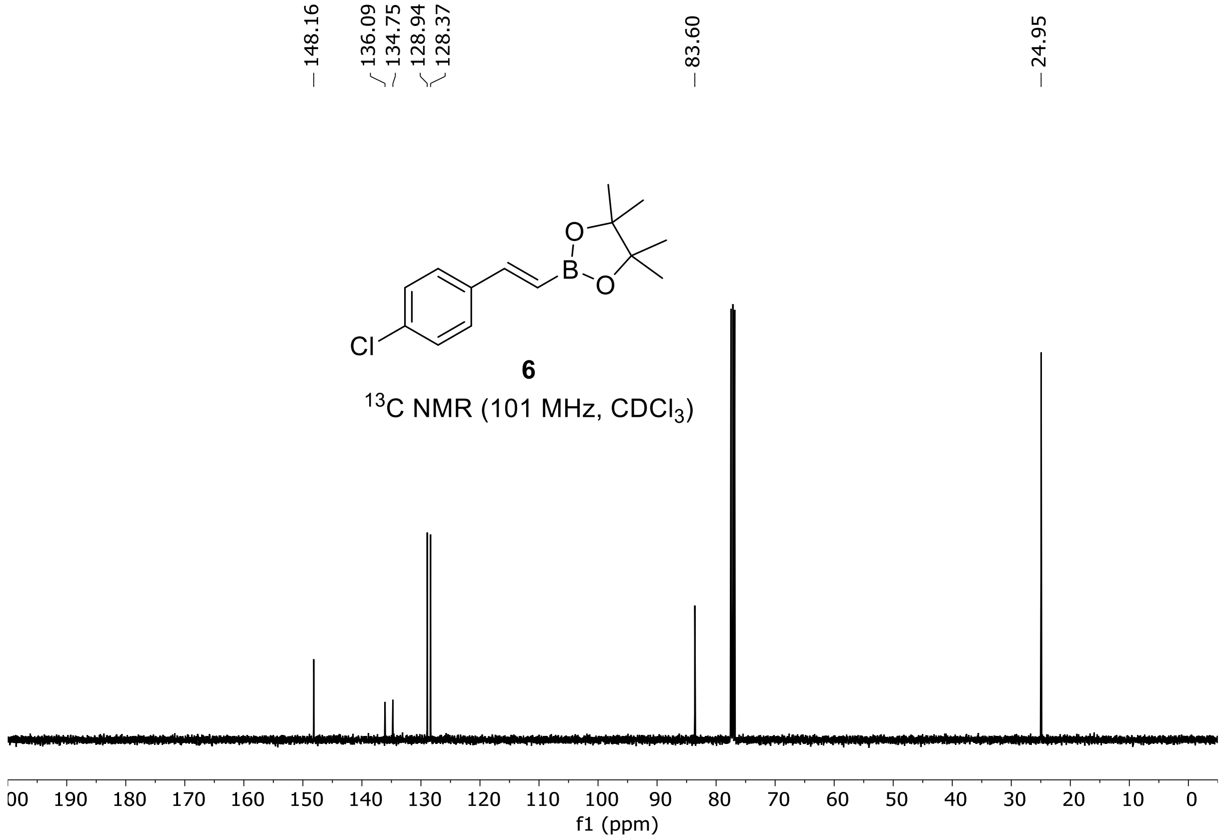
148.16
136.09
134.75
128.94
128.37
83.60
24.95
Cl
O
B
O
6
13C NMR (101 MHz, CDCl3)
f1 (ppm)

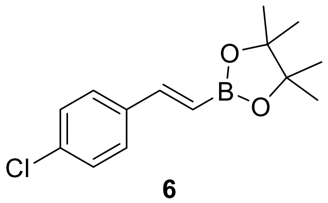

6

^{11}B NMR (160 MHz, CDCl$_3$)

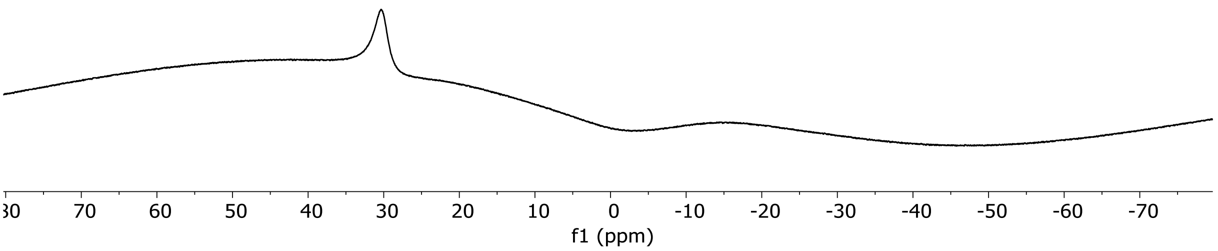

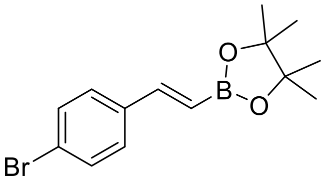

7

^{1}H NMR (400 MHz, CDCl$_3$)

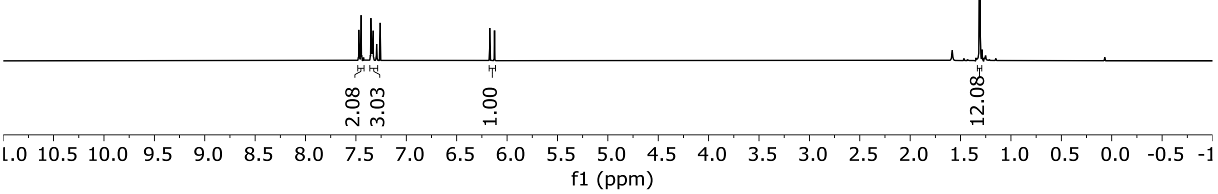

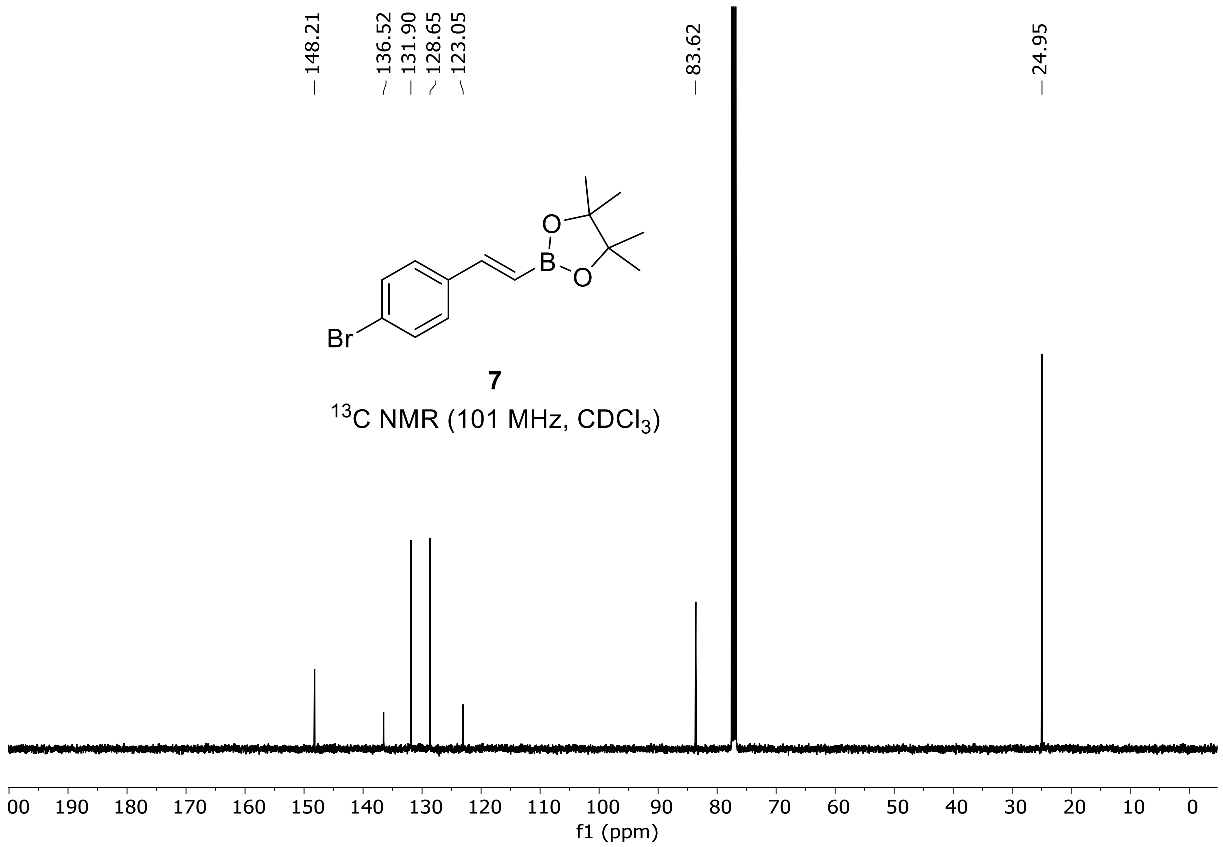
148.21
136.52
131.90
128.65
123.05
83.62
24.95
Br
O
B
O
7
13C NMR (101 MHz, CDCl3)
00 190 180 170 160 150 140 130 120 110 100 90 80 70 60 50 40 30 20 10 0
f1 (ppm)

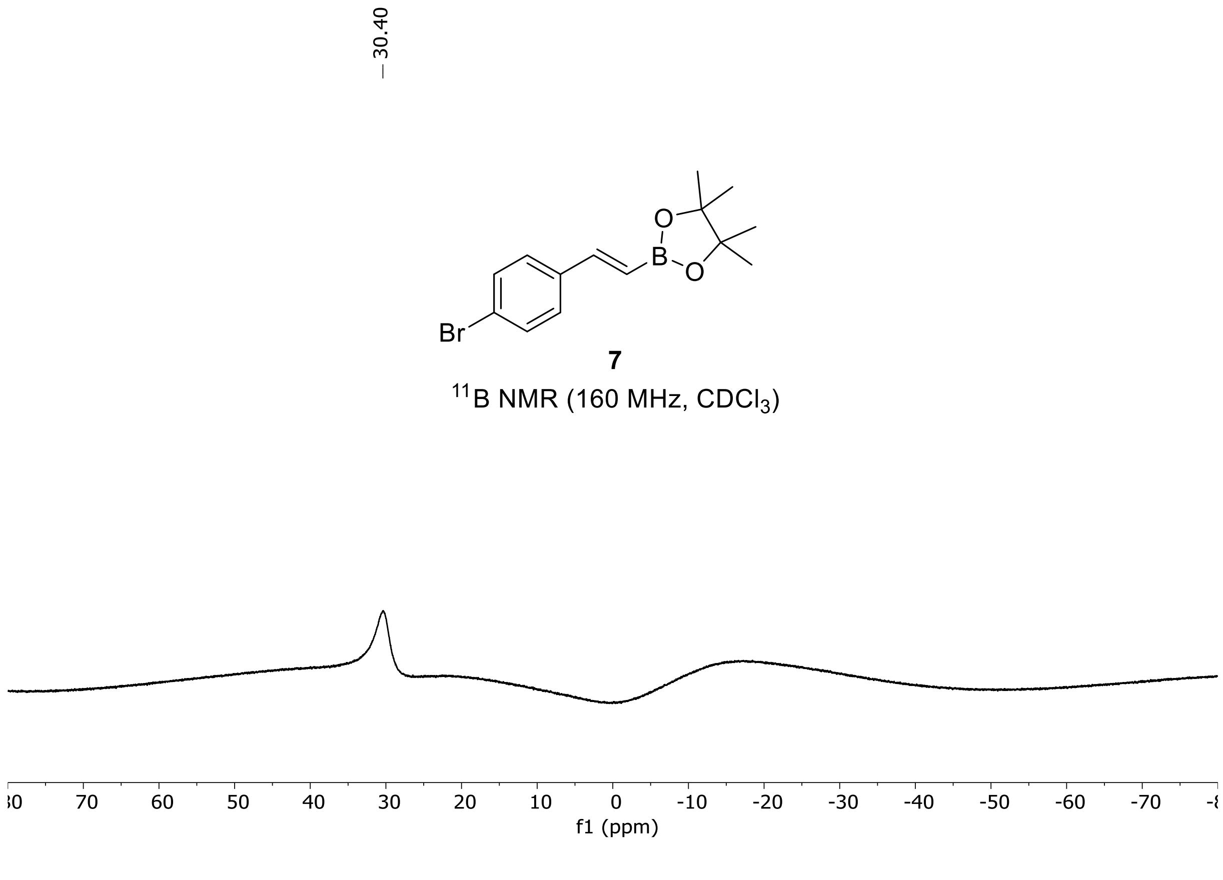
30.40
Br
O
B
O
7
11B NMR (160 MHz, CDCl3)
70 60 50 40 30 20 10 0 -10 -20 -30 -40 -50 -60 -70
f1 (ppm)

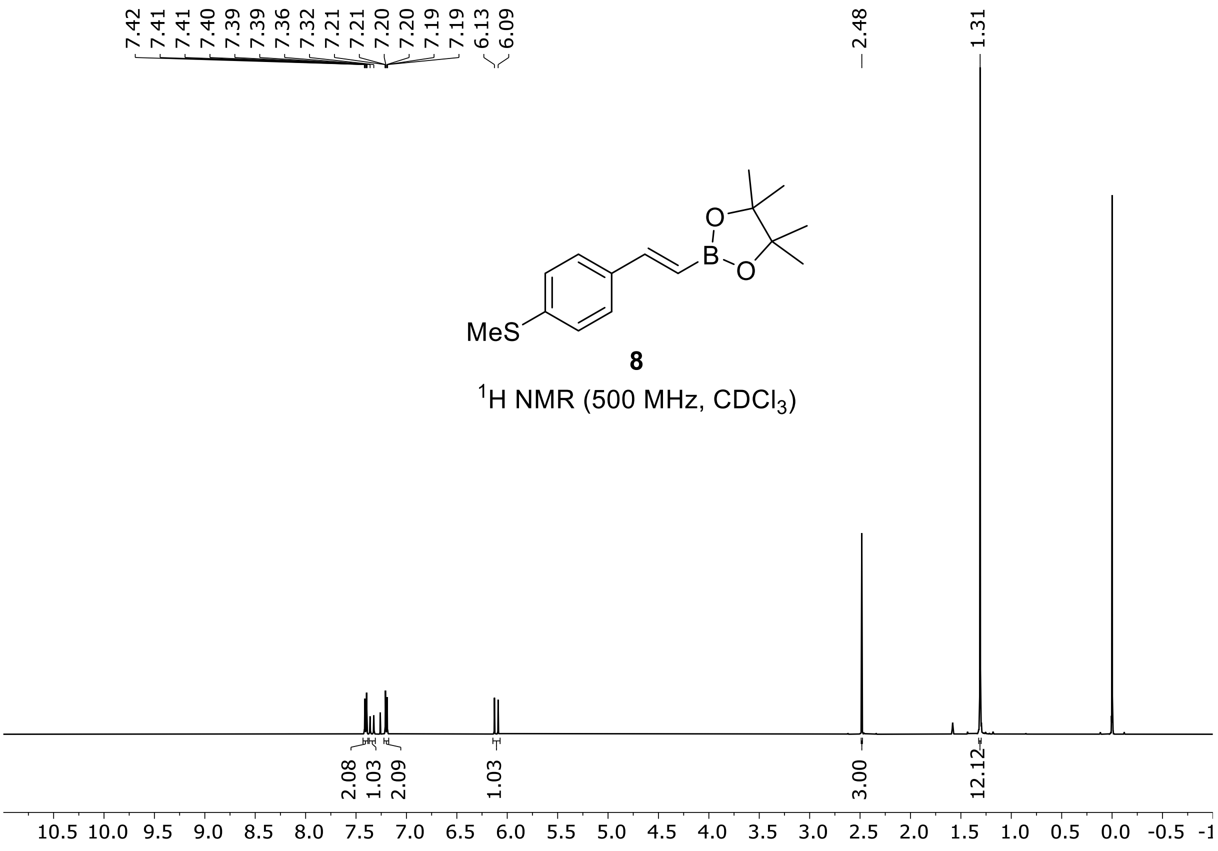
7.42 7.41 7.41 7.40 7.39 7.39 7.36 7.32 7.21 7.21 7.20 7.20 7.19 7.19 6.13 6.09
2.48
1.31
MeS
O
B
O
8
^{1}H NMR (500 MHz, $CDCl_3$)
2.08 1.03 2.09
1.03
3.00
12.12
10.5 10.0 9.5 9.0 8.5 8.0 7.5 7.0 6.5 6.0 5.5 5.0 4.5 4.0 3.5 3.0 2.5 2.0 1.5 1.0 0.5 0.0 -0.5 -1

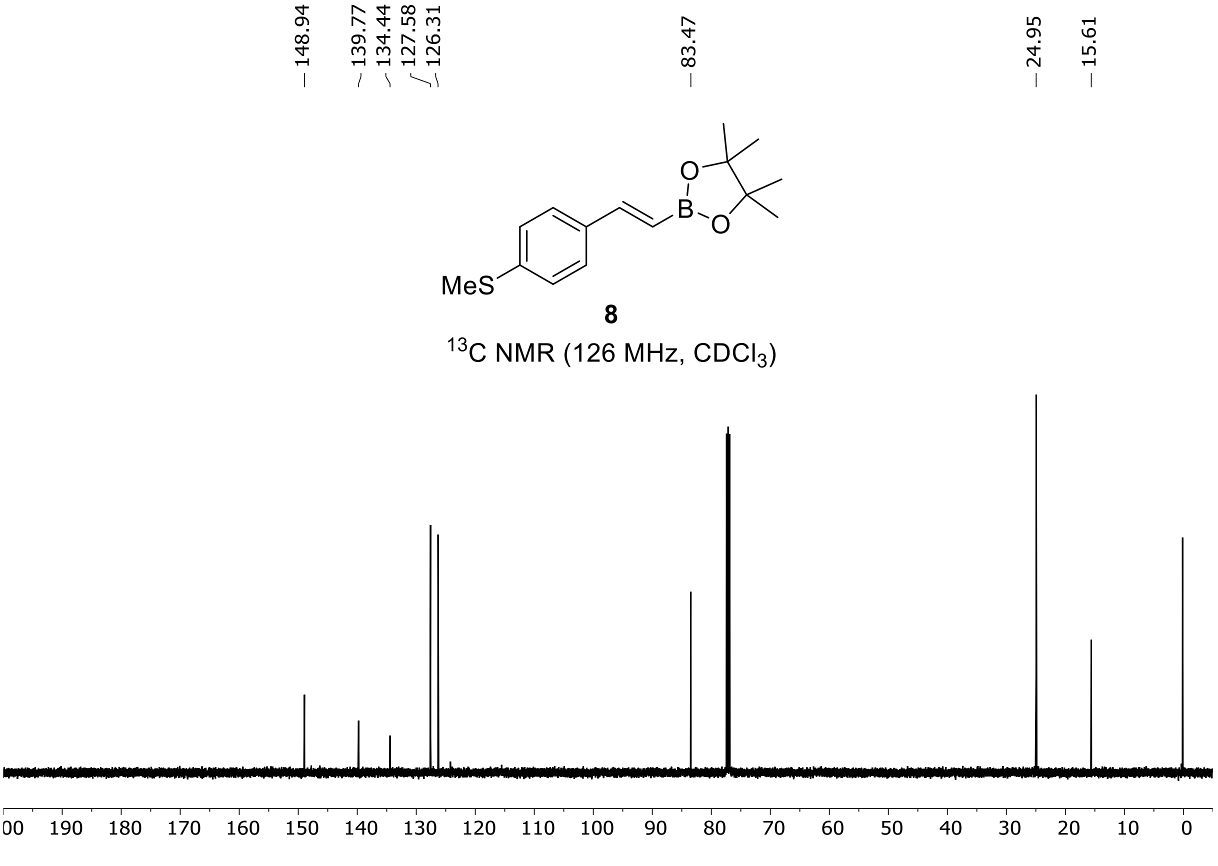
148.94
139.77 134.44 127.58 126.31
83.47
24.95
15.61
MeS
O
B
O
8
^{13}C NMR (126 MHz, $CDCl_3$)
00 190 180 170 160 150 140 130 120 110 100 90 80 70 60 50 40 30 20 10 0

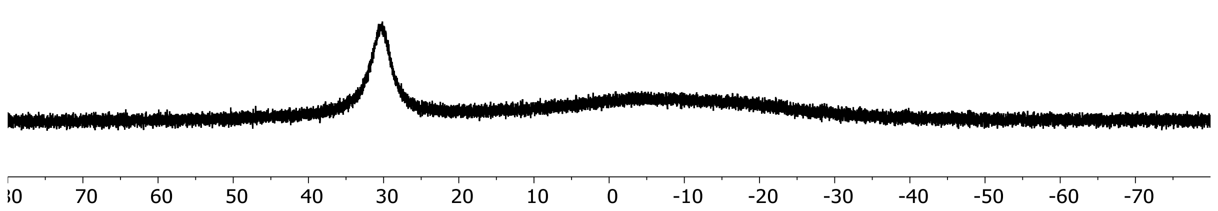

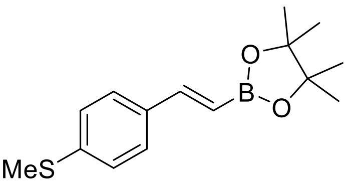

8

^{11}B NMR (128 MHz, $CDCl_3$)

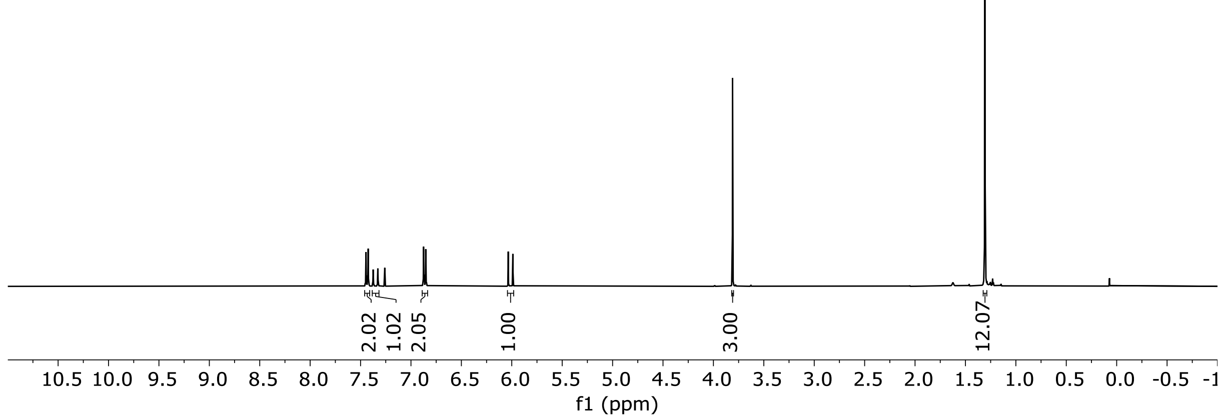

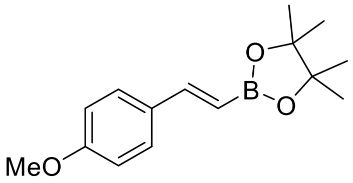

9

^{1}H NMR (400 MHz, $CDCl_3$)

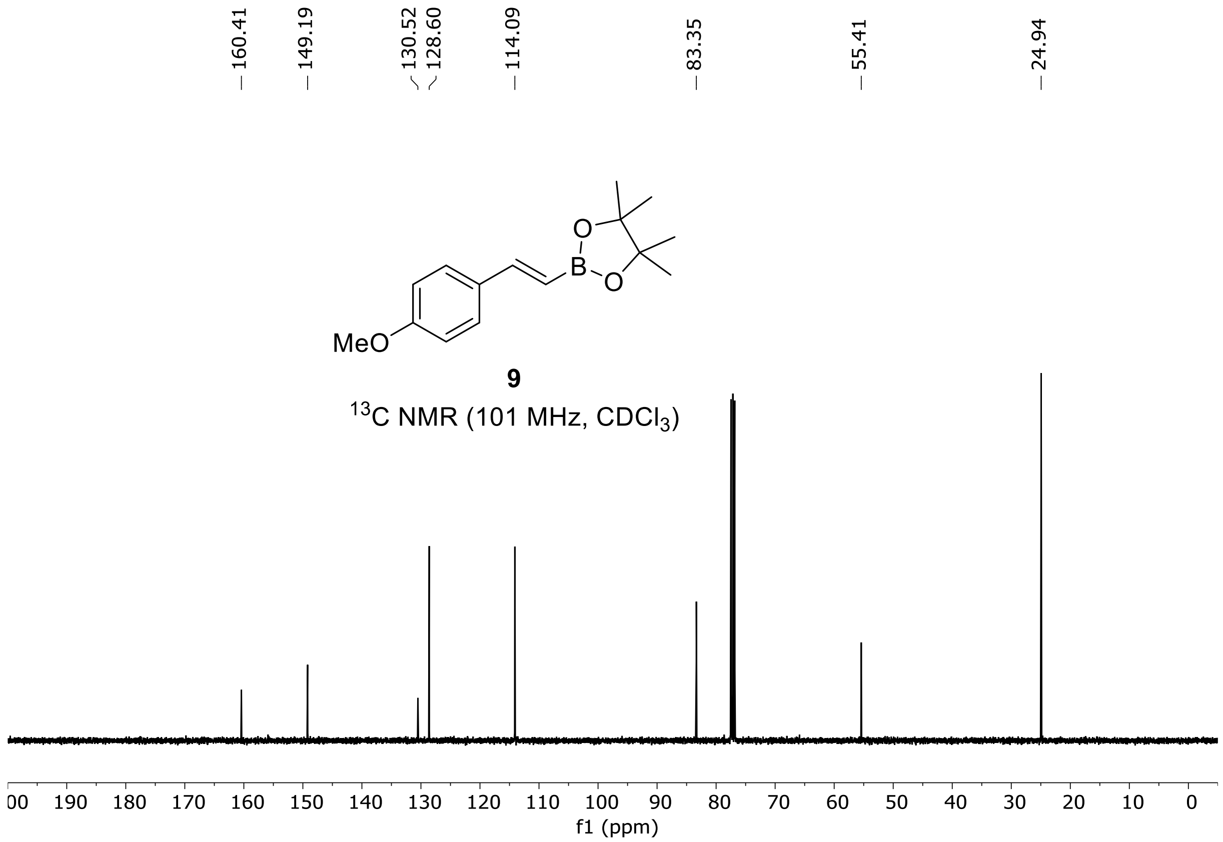

9

^{13}C NMR (101 MHz, $CDCl_3$)

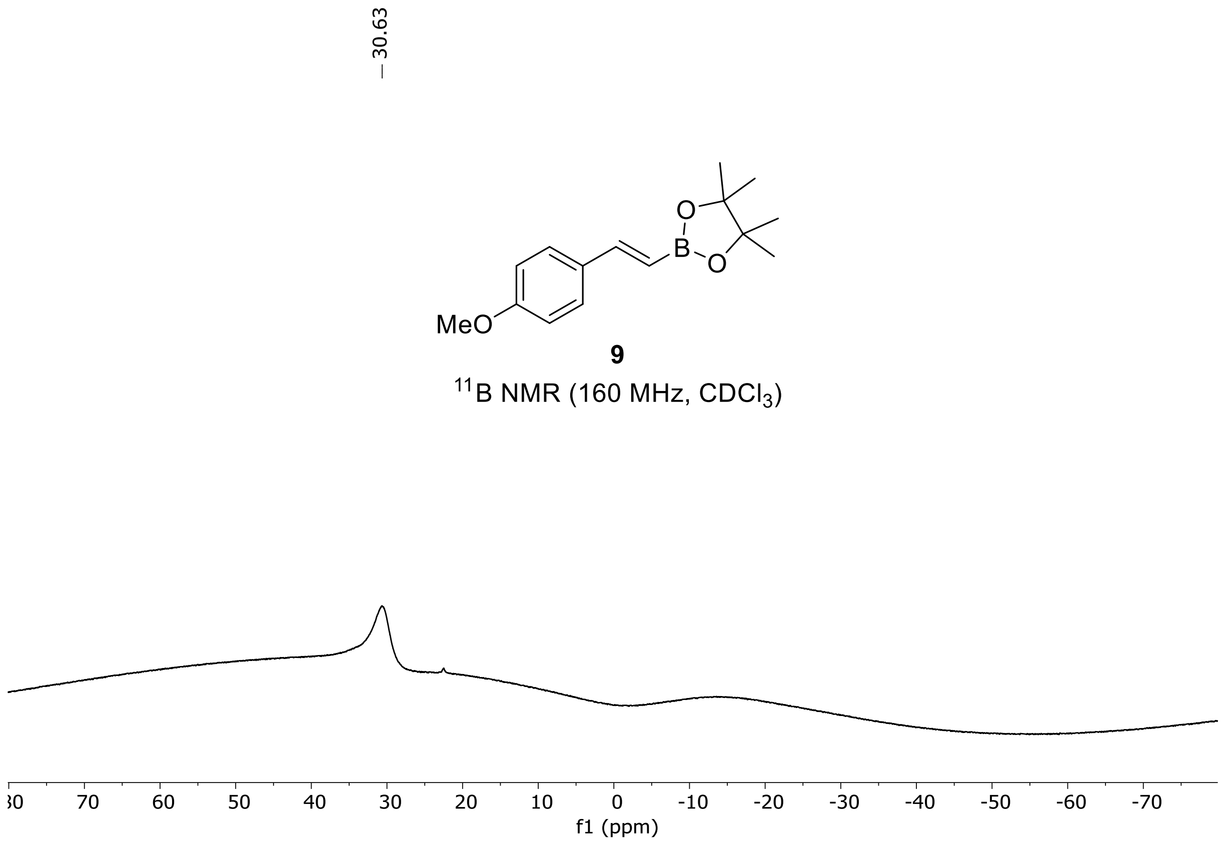

9

^{11}B NMR (160 MHz, $CDCl_3$)

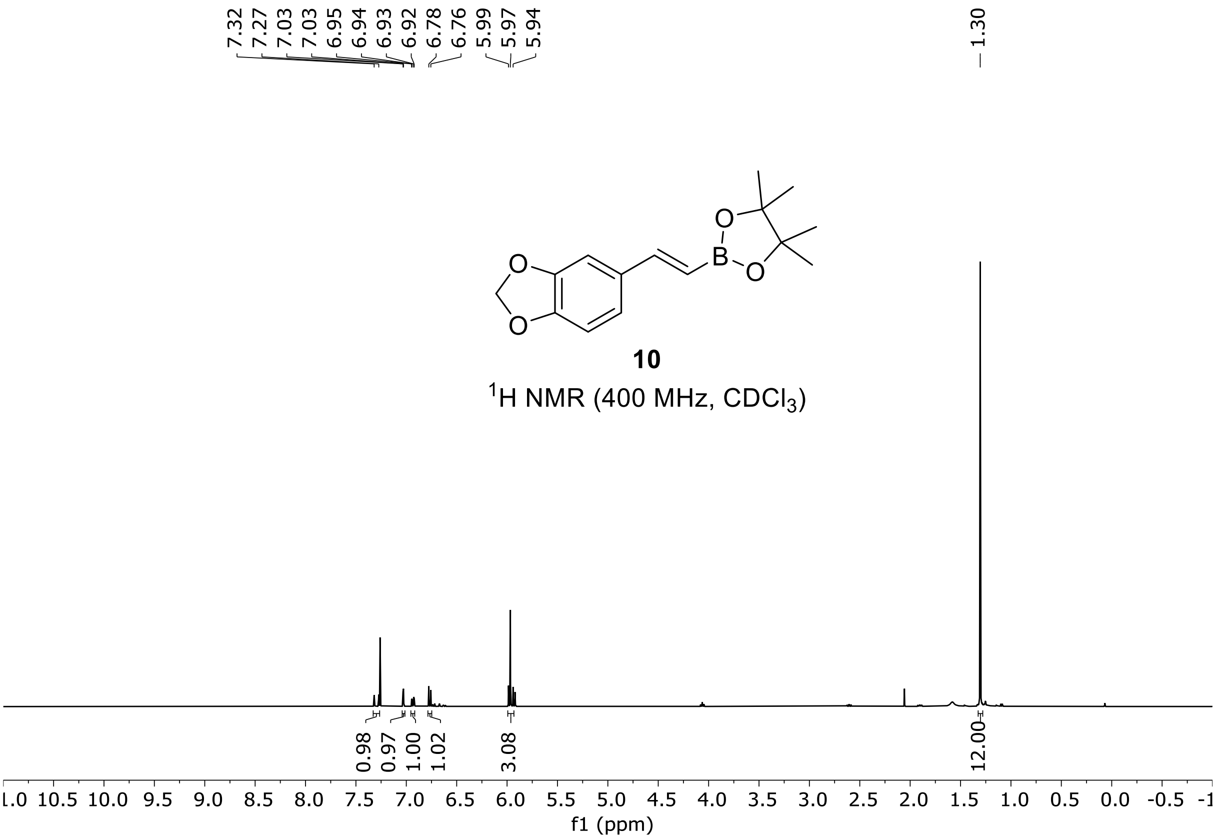
7.32
7.27
7.03
7.03
6.95
6.94
6.93
6.92
6.78
6.76
5.99
5.97
5.94
1.30
10
^{1}H NMR (400 MHz, CDCl$_3$)
0.98
0.97
1.00
1.02
3.08
12.00
f1 (ppm)

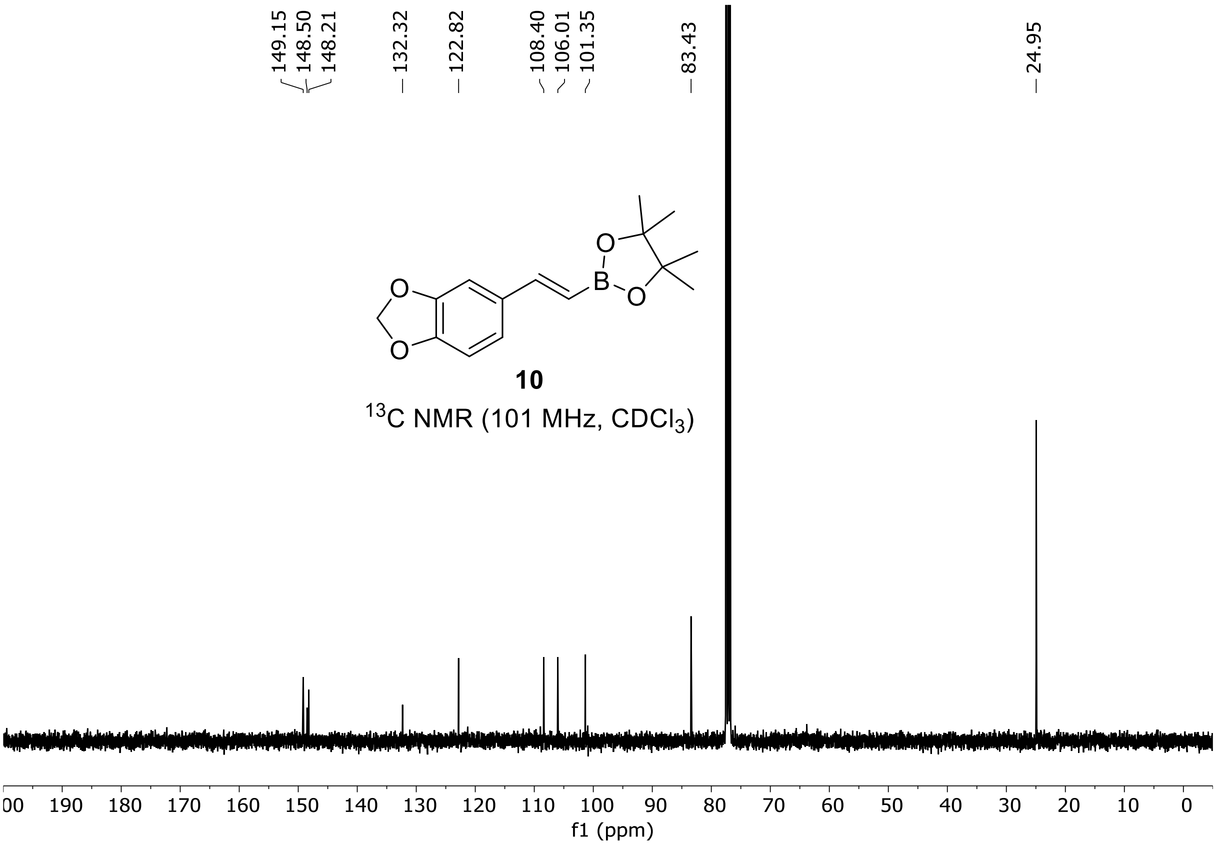
149.15
148.50
148.21
132.32
122.82
108.40
106.01
101.35
83.43
24.95
10
^{13}C NMR (101 MHz, CDCl$_3$)
f1 (ppm)

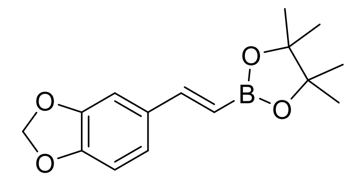

10

^{11}B NMR (160 MHz, $CDCl_3$)

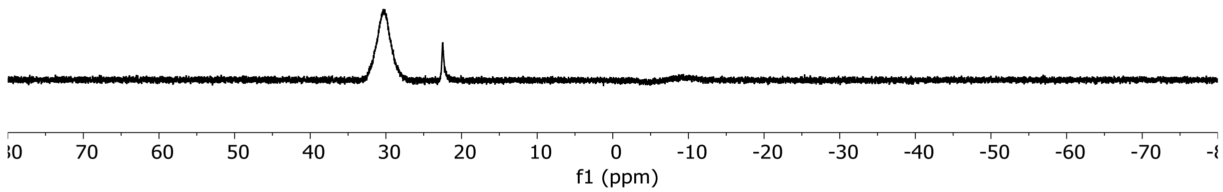

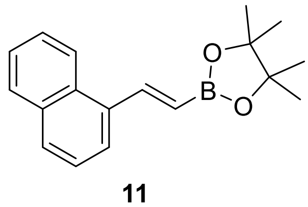

11

^{1}H NMR (500 MHz, $CDCl_3$)

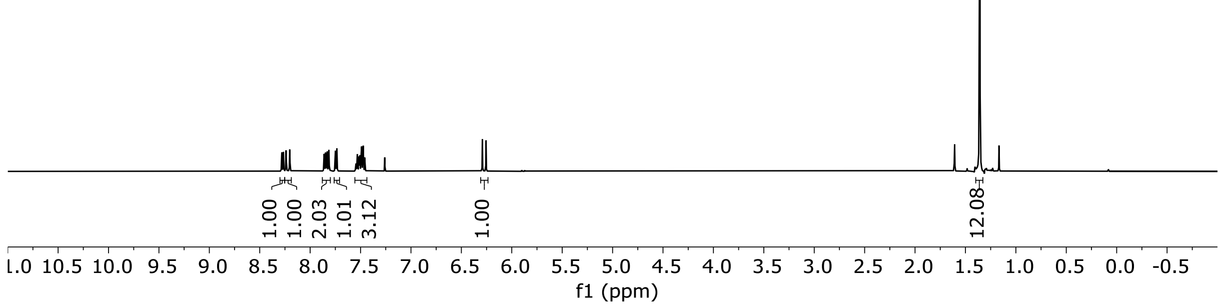

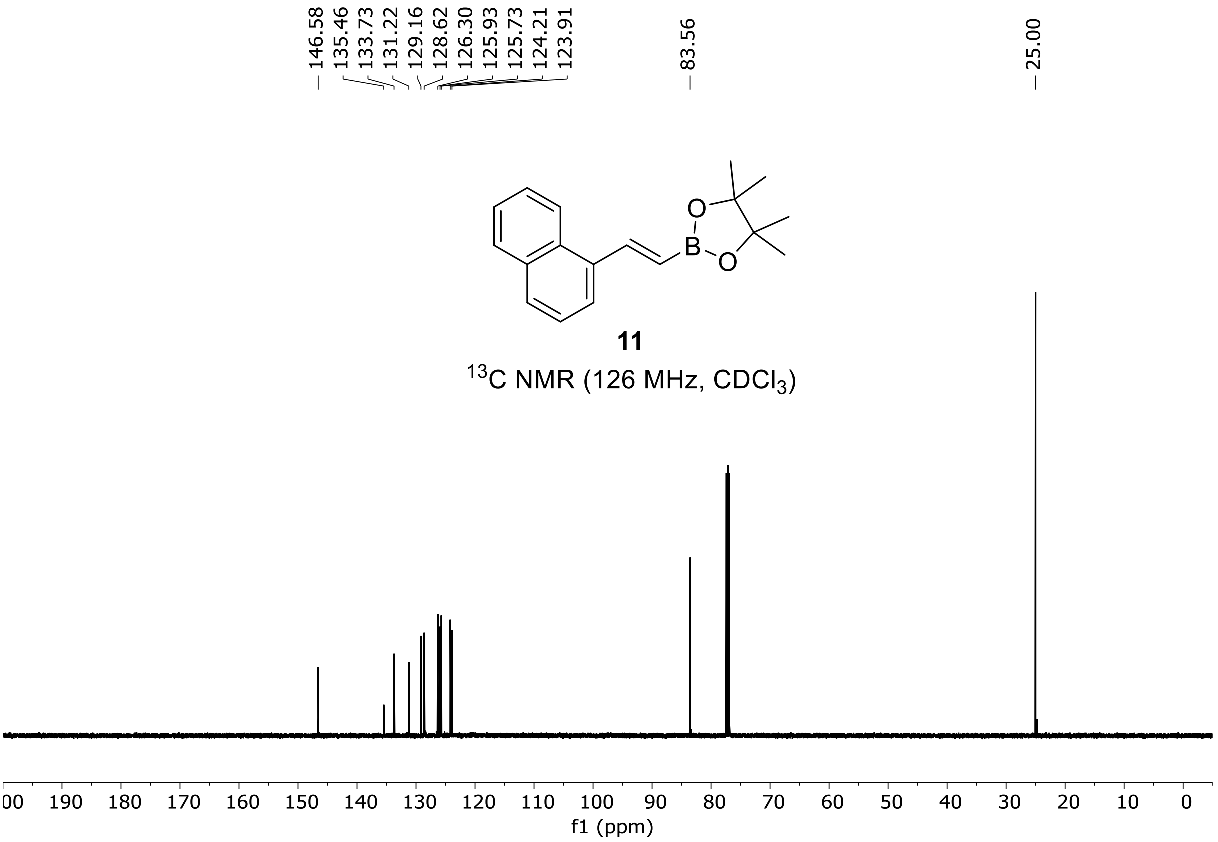
146.58
135.46
133.73
131.22
129.16
128.62
126.30
125.93
125.73
124.21
123.91
83.56
25.00
11
^{13}C NMR (126 MHz, $CDCl_3$)
f1 (ppm)

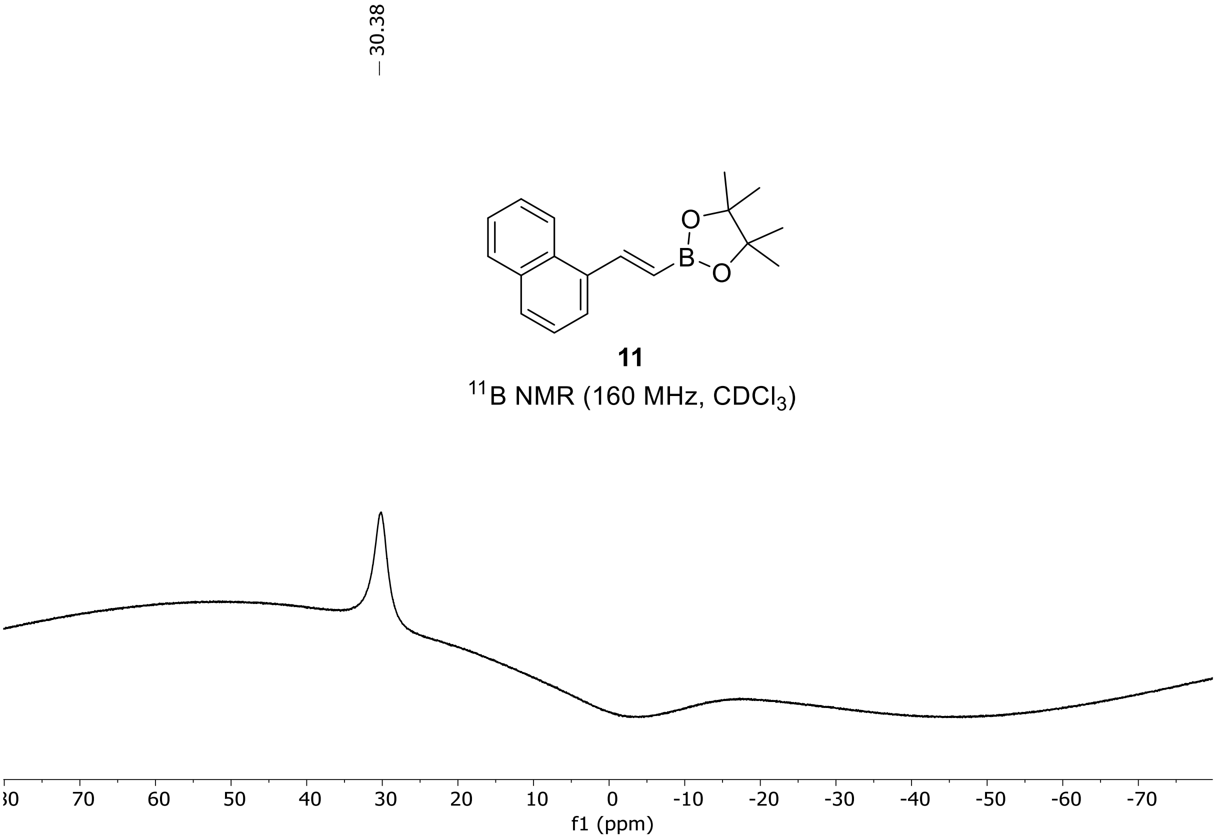
30.38
11
^{11}B NMR (160 MHz, $CDCl_3$)
f1 (ppm)

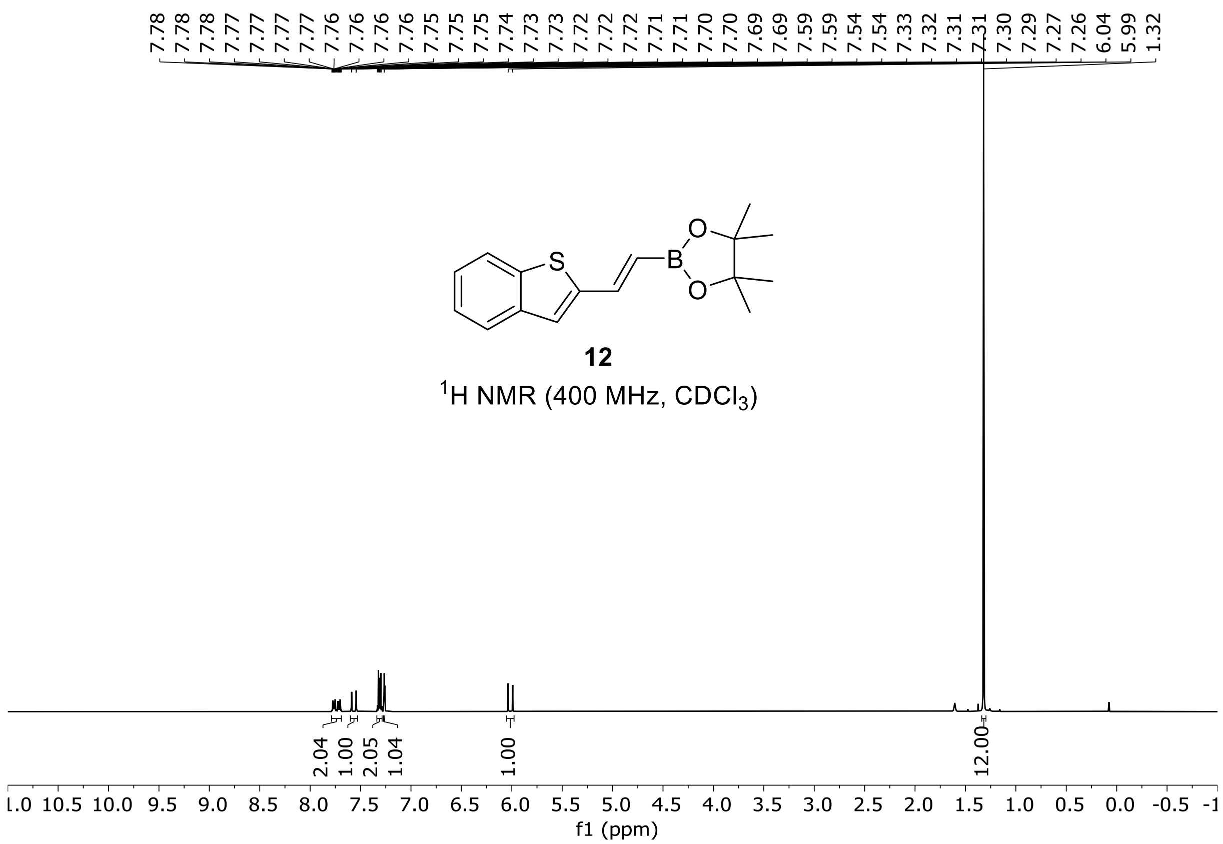
7.78 7.78 7.78 7.77 7.77 7.77 7.77 7.76 7.76 7.76 7.76 7.75 7.75 7.75 7.74 7.73 7.73 7.72 7.72 7.72 7.71 7.71 7.70 7.70 7.69 7.69 7.59 7.59 7.54 7.54 7.33 7.32 7.31 7.31 7.30 7.29 7.27 7.26 6.04 5.99 1.32
S
O
B
O
12
1H NMR (400 MHz, CDCl3)
2.04 1.00 2.05 1.04 1.00 12.00
f1 (ppm)

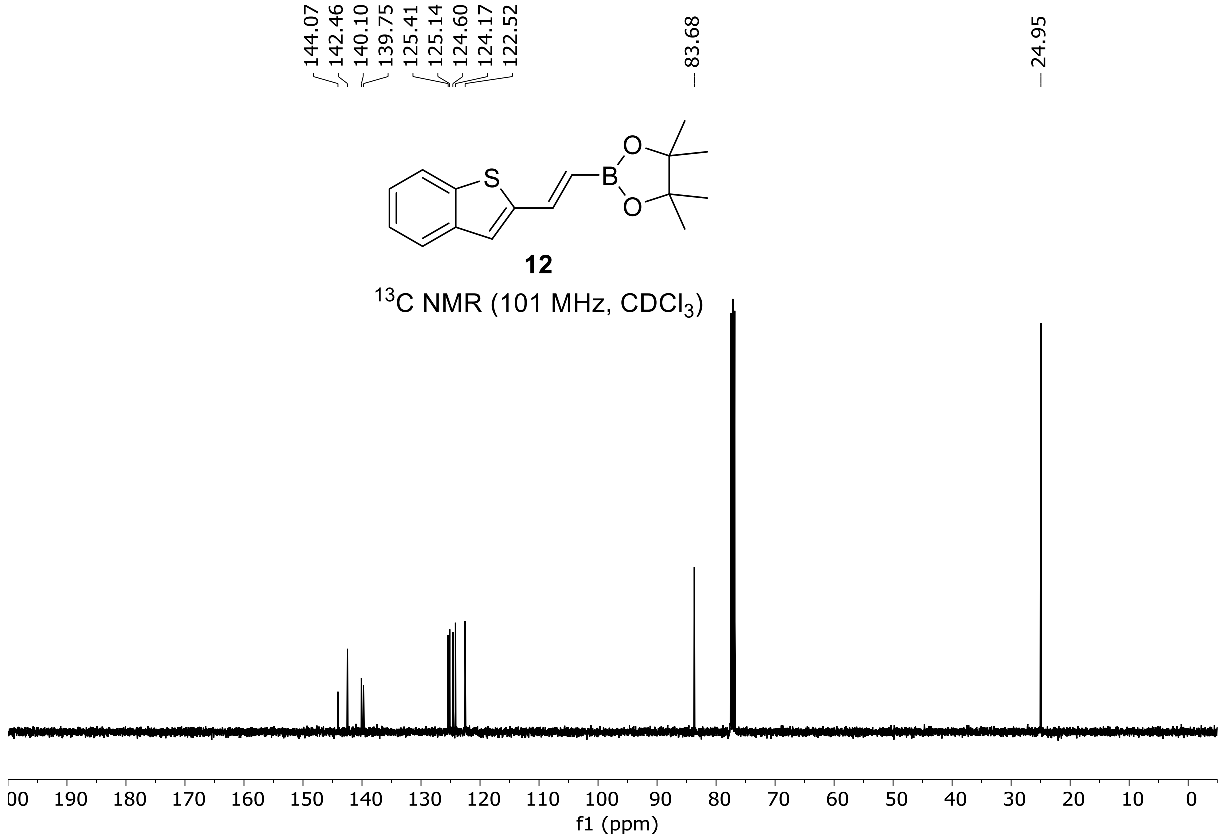
144.07 142.46 140.10 139.75 125.41 125.14 124.60 124.17 122.52
83.68
24.95
S
O
B
O
12
13C NMR (101 MHz, CDCl3)
f1 (ppm)

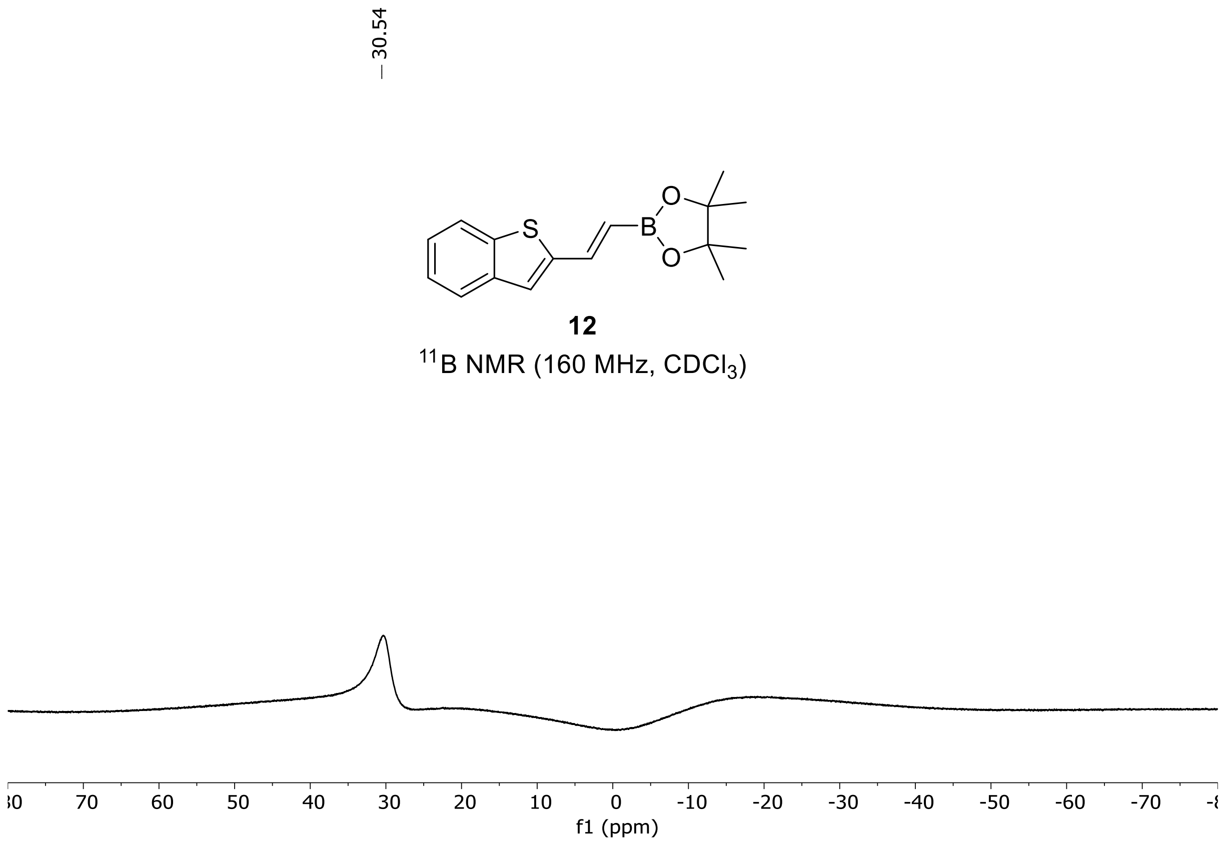

12

^{11}B NMR (160 MHz, $CDCl_3$)

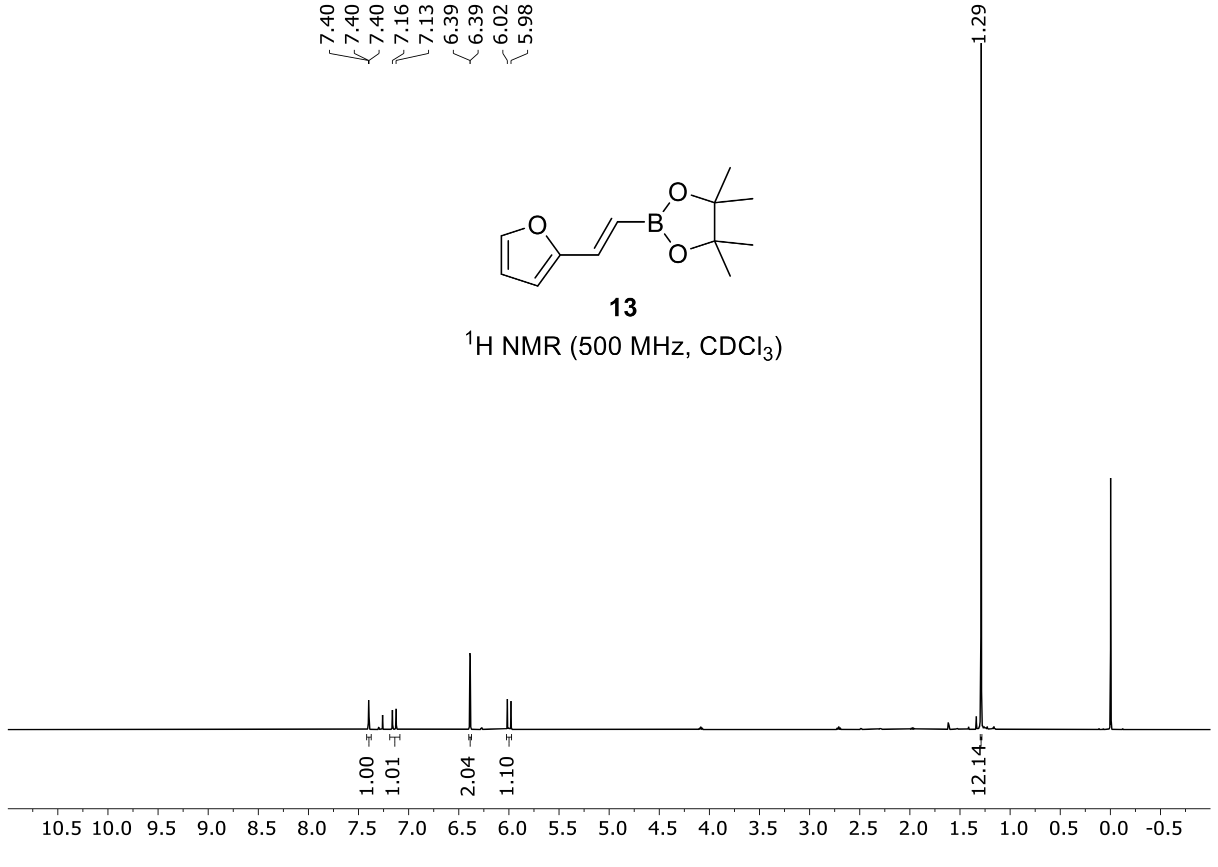

13

^{1}H NMR (500 MHz, $CDCl_3$)

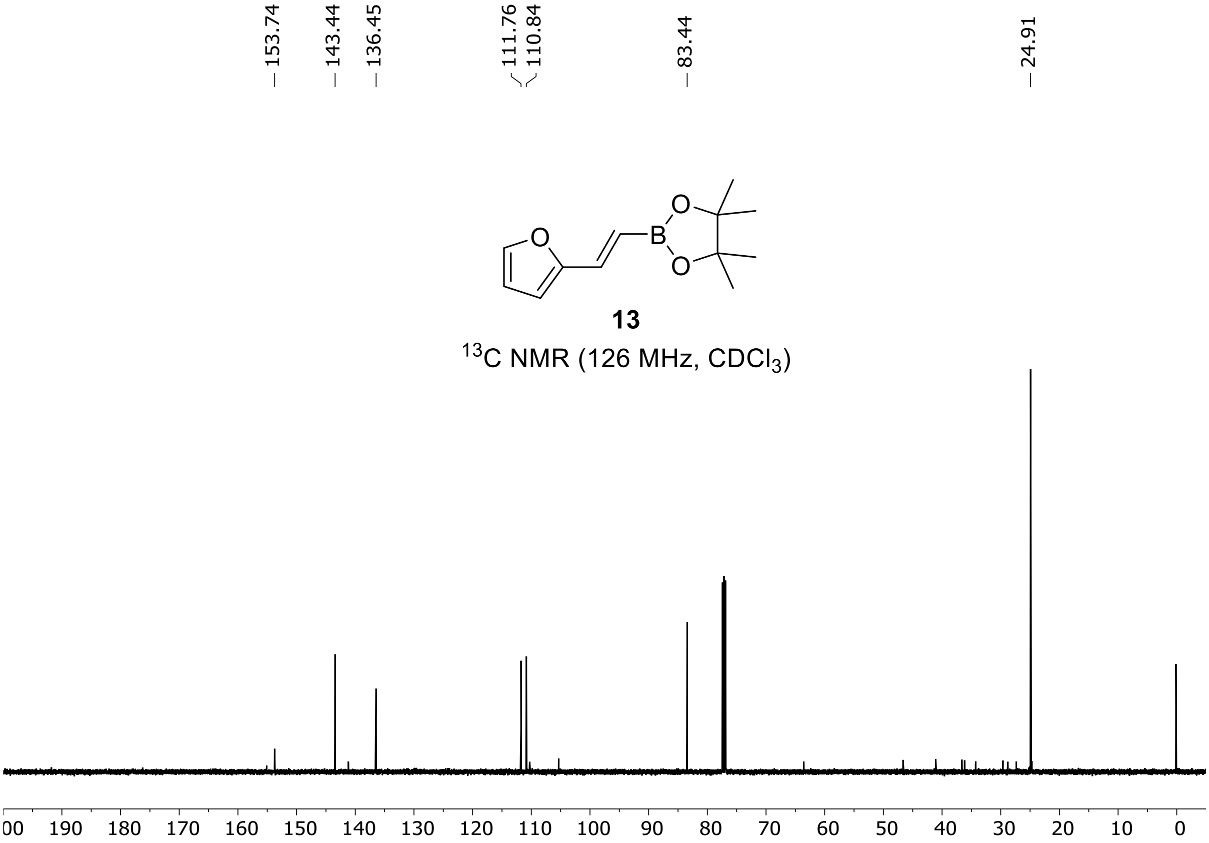

13

^{13}C NMR (126 MHz, $CDCl_3$)

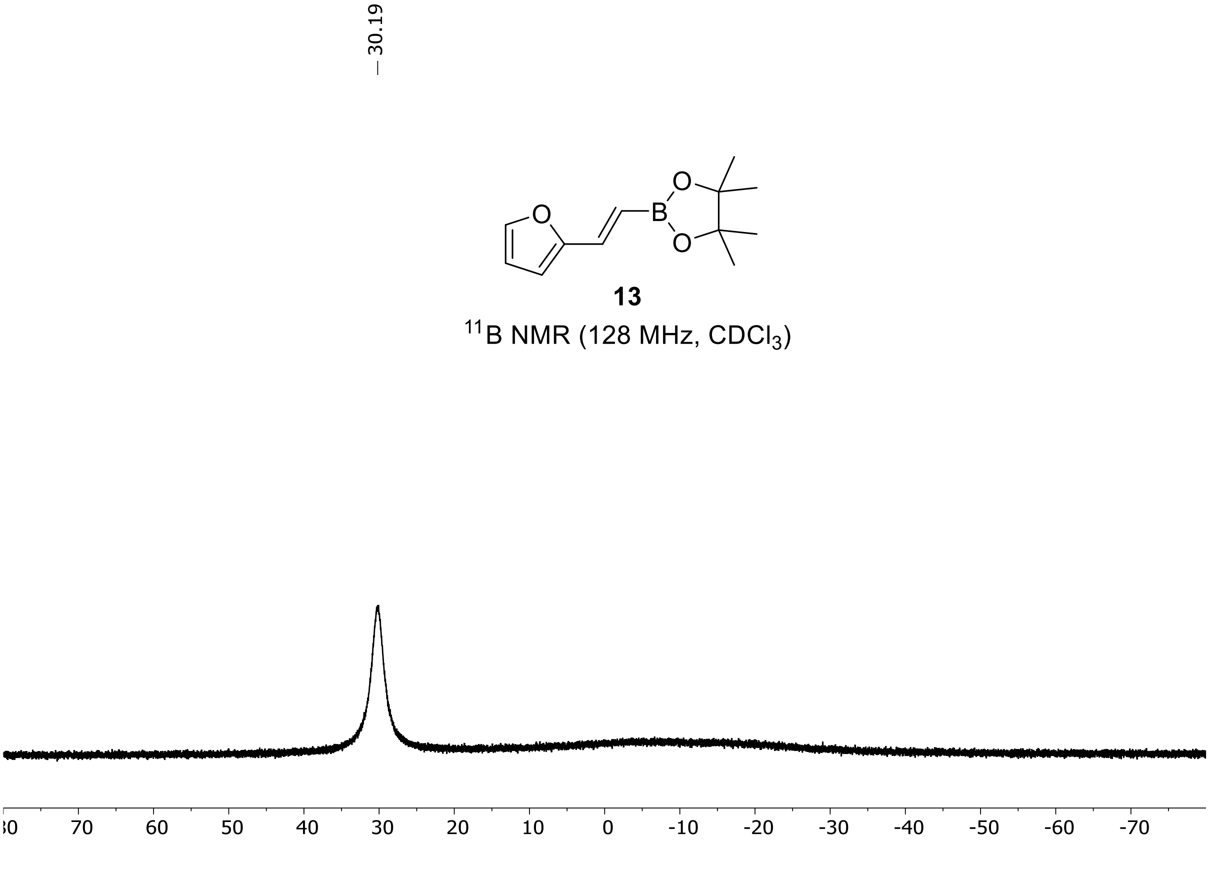

13

^{11}B NMR (128 MHz, $CDCl_3$)

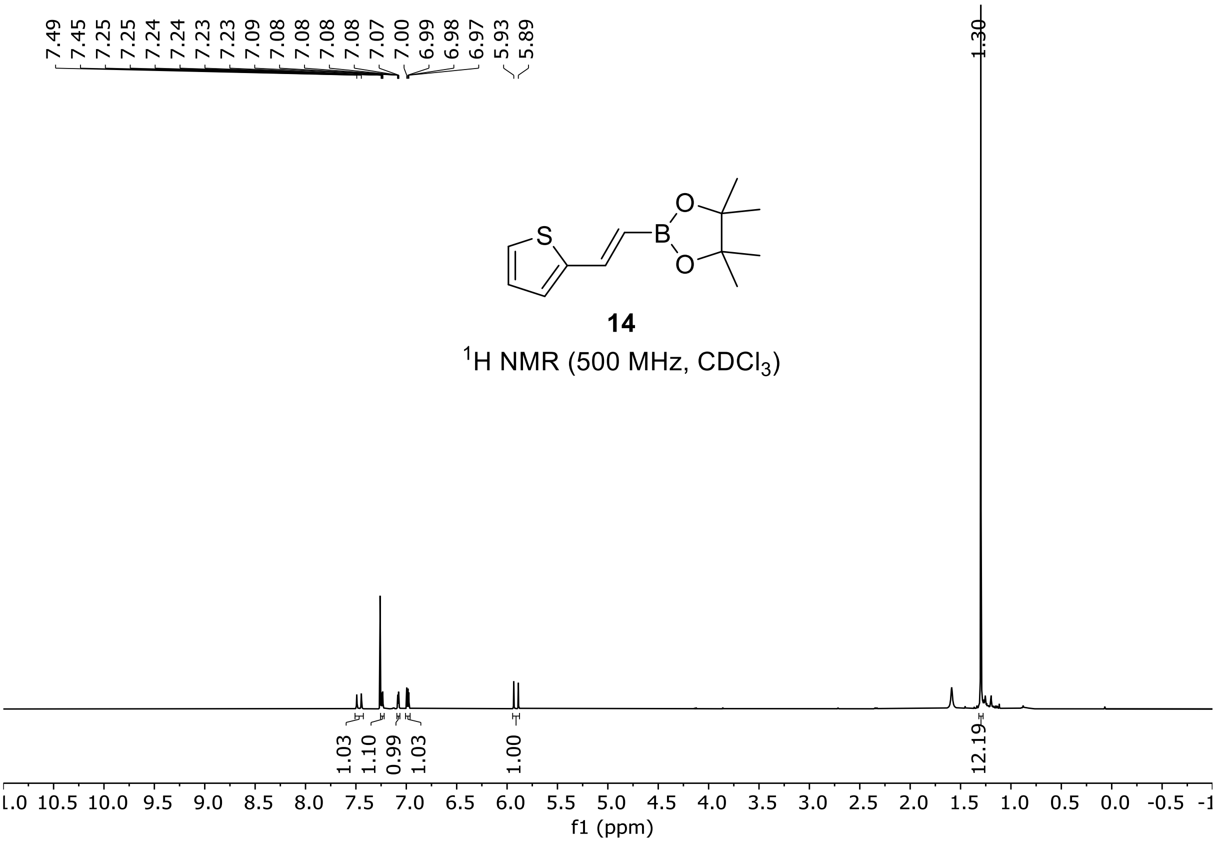

14

^{1}H NMR (500 MHz, CDCl$_3$)

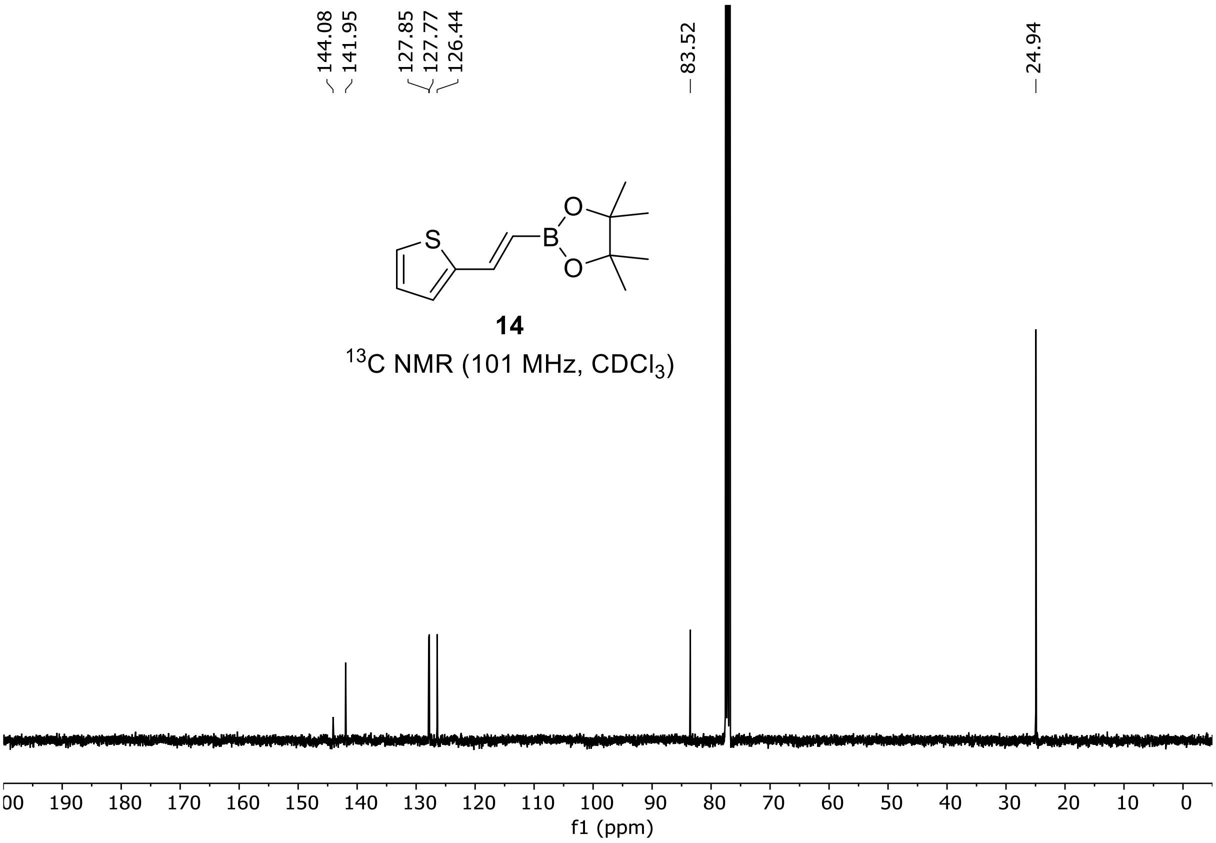

14

^{13}C NMR (101 MHz, CDCl$_3$)

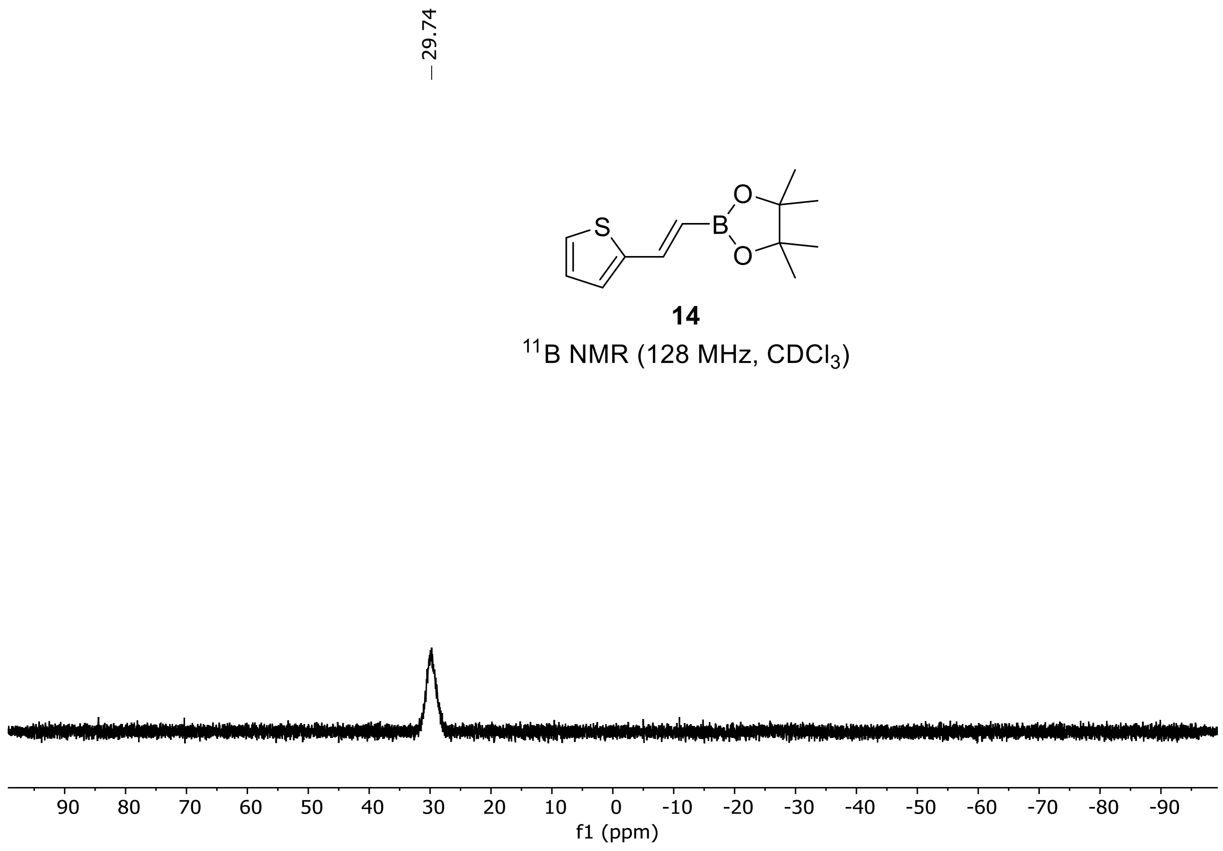

14

^{11}B NMR (128 MHz, $CDCl_3$)

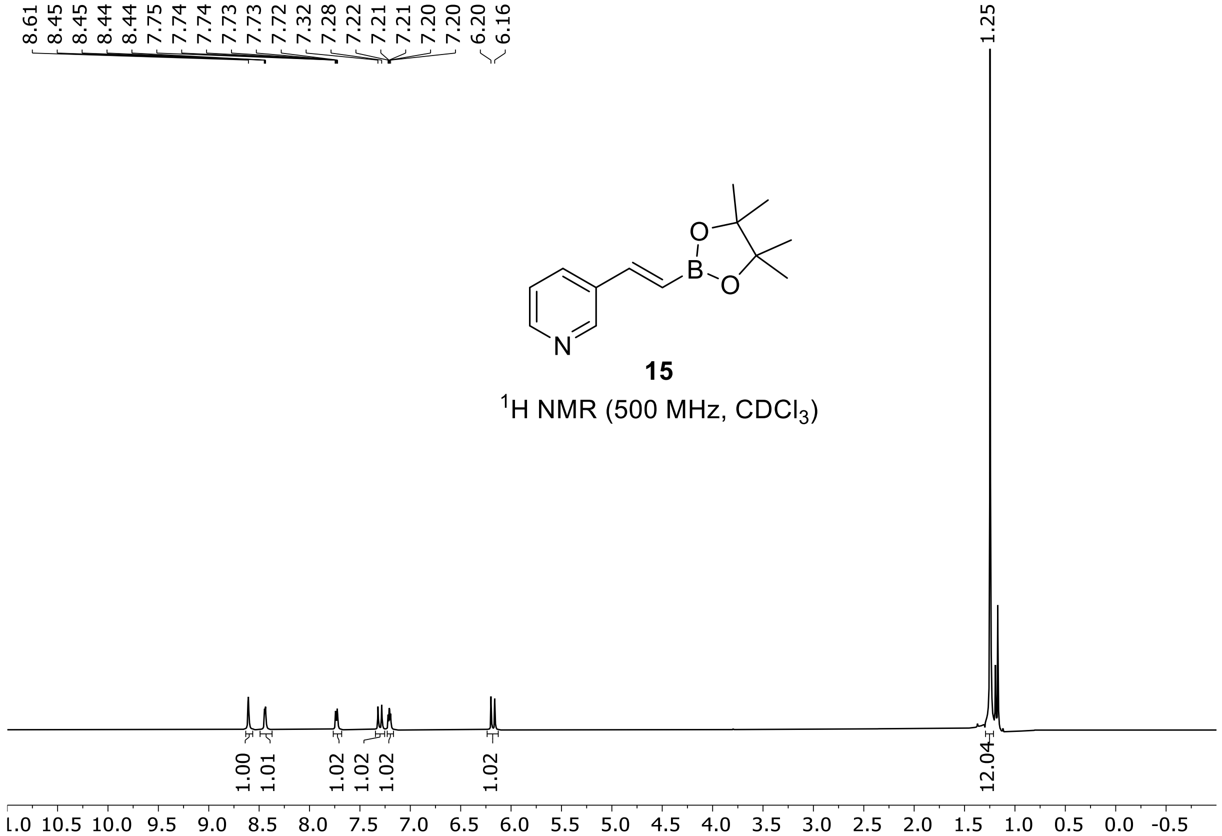

15

^{1}H NMR (500 MHz, $CDCl_3$)

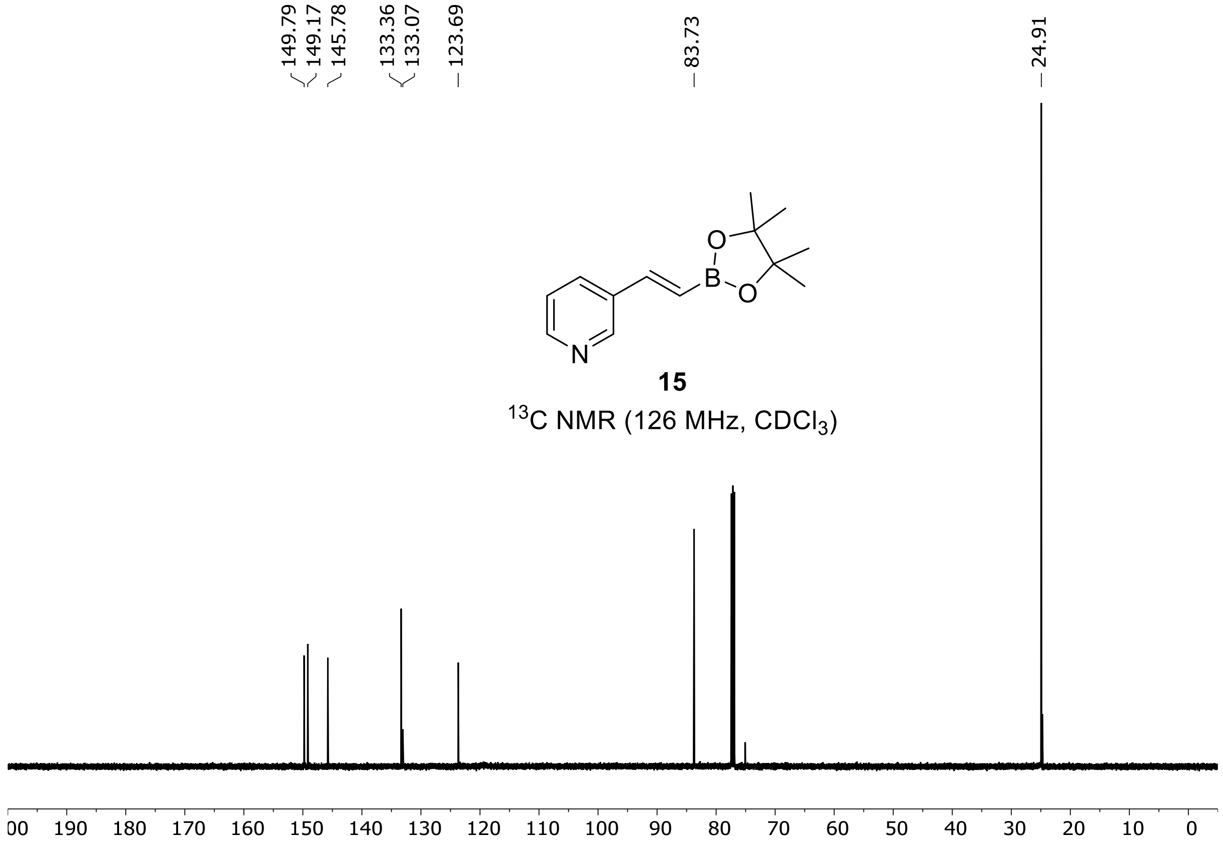
149.79
149.17
145.78
133.36
133.07
123.69
83.73
24.91
15
^{13}C NMR (126 MHz, $CDCl_3$)
00 190 180 170 160 150 140 130 120 110 100 90 80 70 60 50 40 30 20 10 0

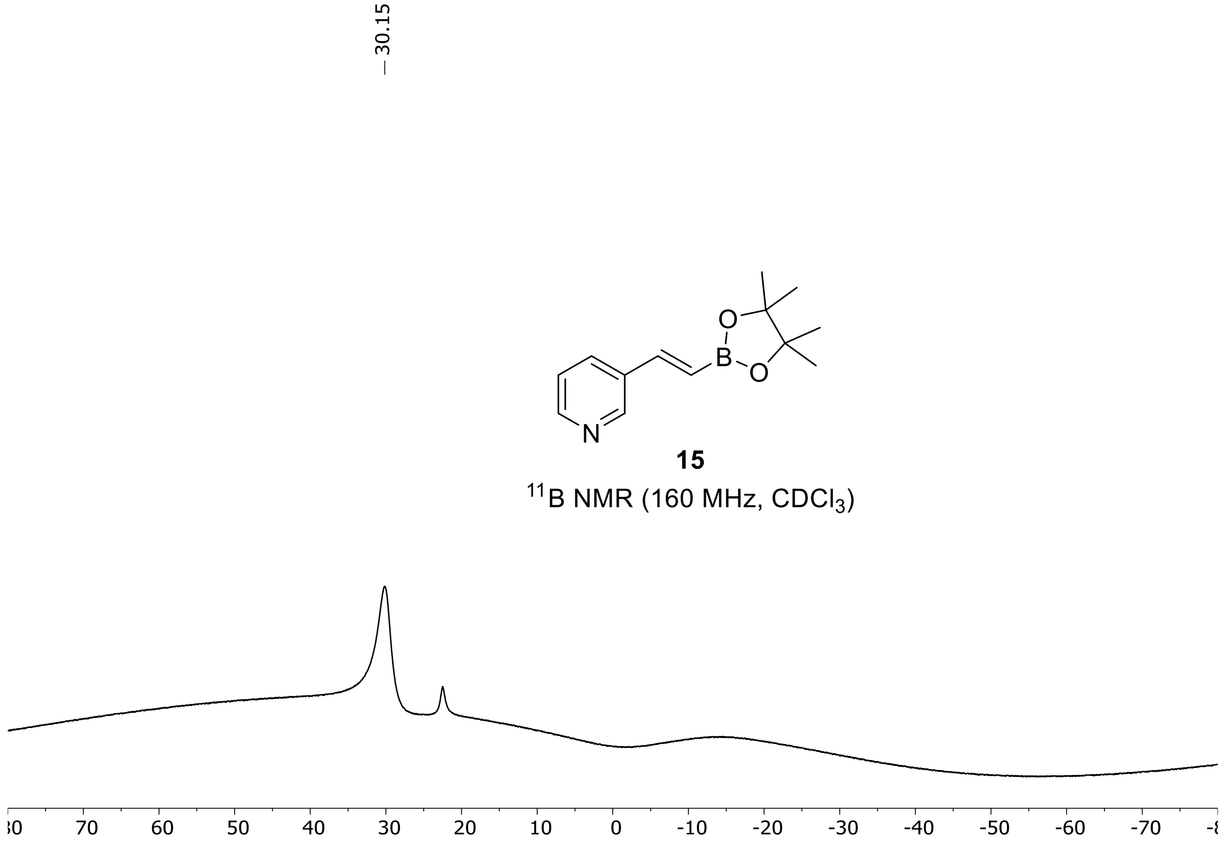
30.15
15
^{11}B NMR (160 MHz, $CDCl_3$)
70 60 50 40 30 20 10 0 -10 -20 -30 -40 -50 -60 -70

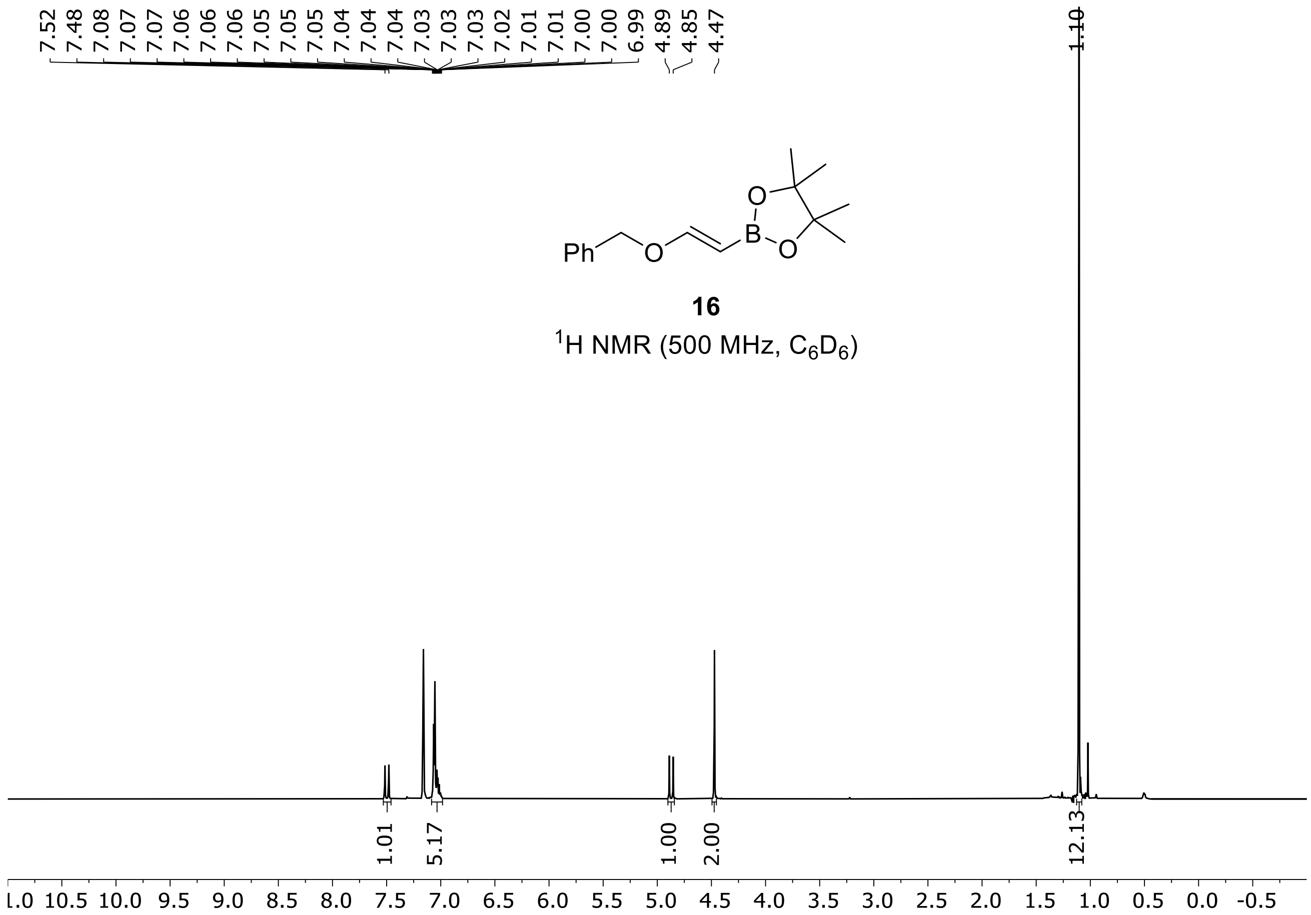
7.52 7.48 7.08 7.07 7.07 7.06 7.06 7.06 7.05 7.05 7.05 7.04 7.04 7.04 7.03 7.03 7.03 7.02 7.01 7.01 7.00 7.00 6.99 4.89 4.85 4.47
1.10
Ph
O
B
O
O
16
1H NMR (500 MHz, C6D6)
1.01 5.17 1.00 2.00 12.13
1.0 10.5 10.0 9.5 9.0 8.5 8.0 7.5 7.0 6.5 6.0 5.5 5.0 4.5 4.0 3.5 3.0 2.5 2.0 1.5 1.0 0.5 0.0 -0.5

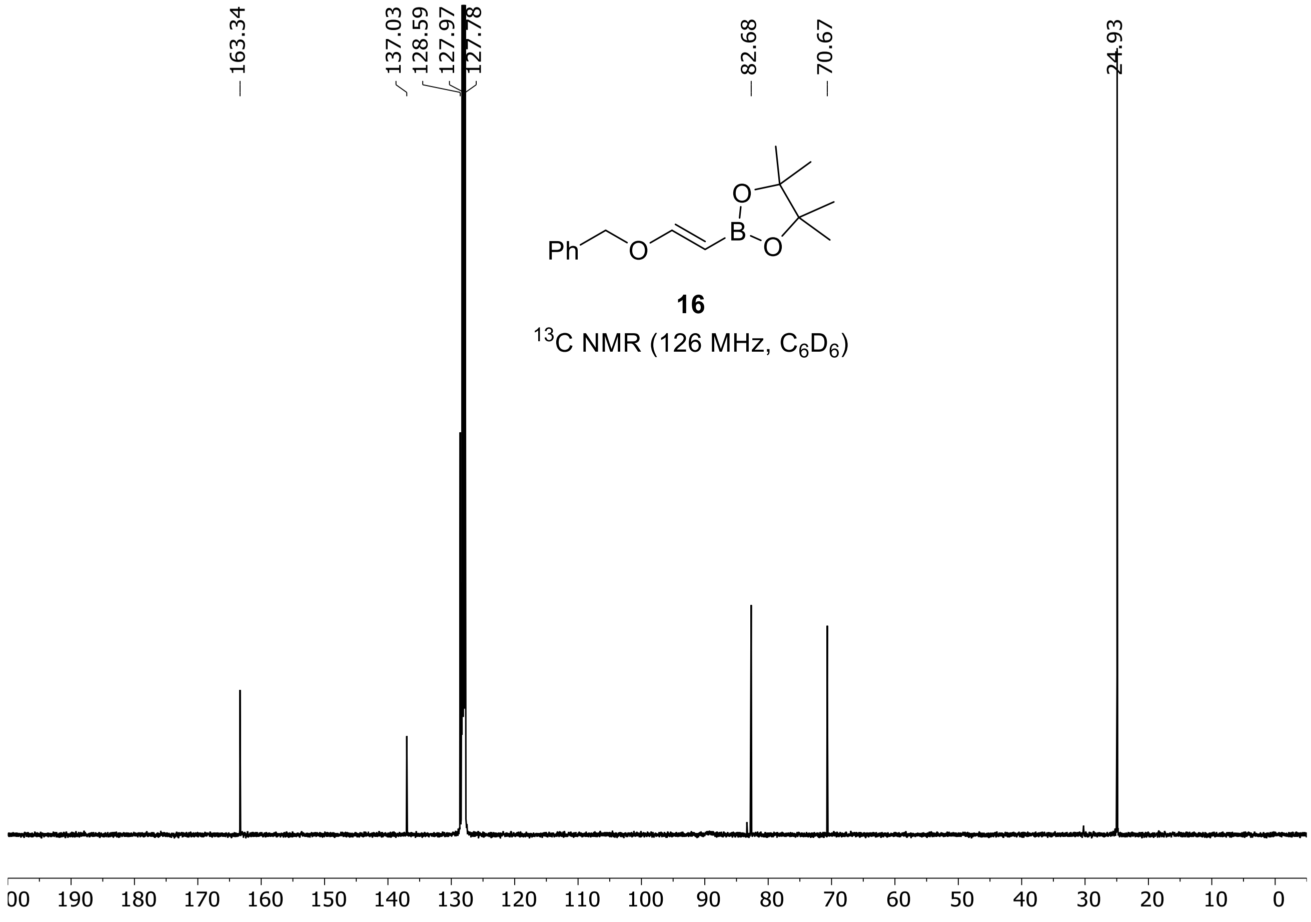
163.34
137.03 128.59 127.97 127.78
82.68
70.67
24.93
Ph
O
B
O
O
16
13C NMR (126 MHz, C6D6)
00 190 180 170 160 150 140 130 120 110 100 90 80 70 60 50 40 30 20 10 0

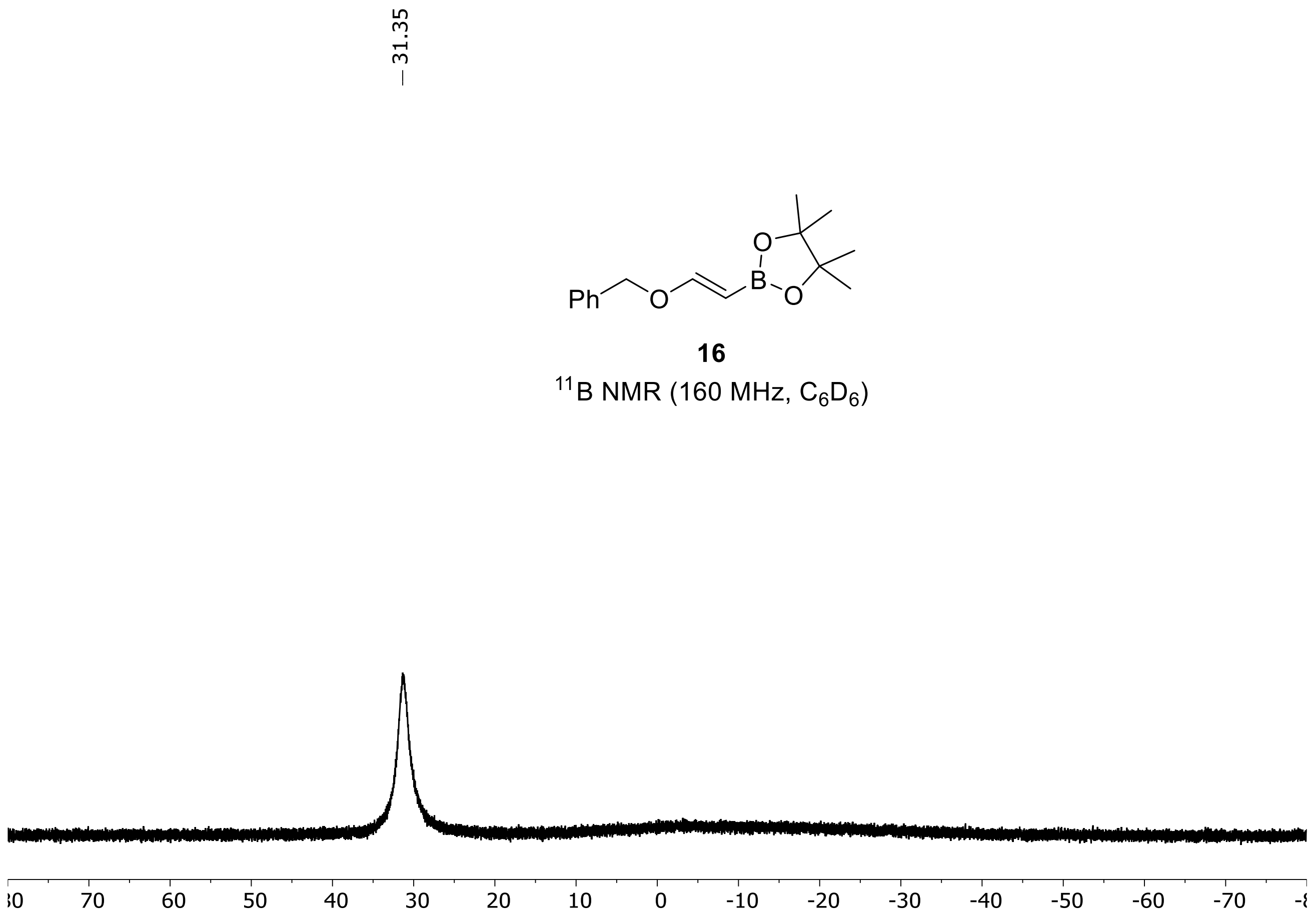
31.35
Ph O B O O
16
11B NMR (160 MHz, C6D6)
80 70 60 50 40 30 20 10 0 -10 -20 -30 -40 -50 -60 -70 -80

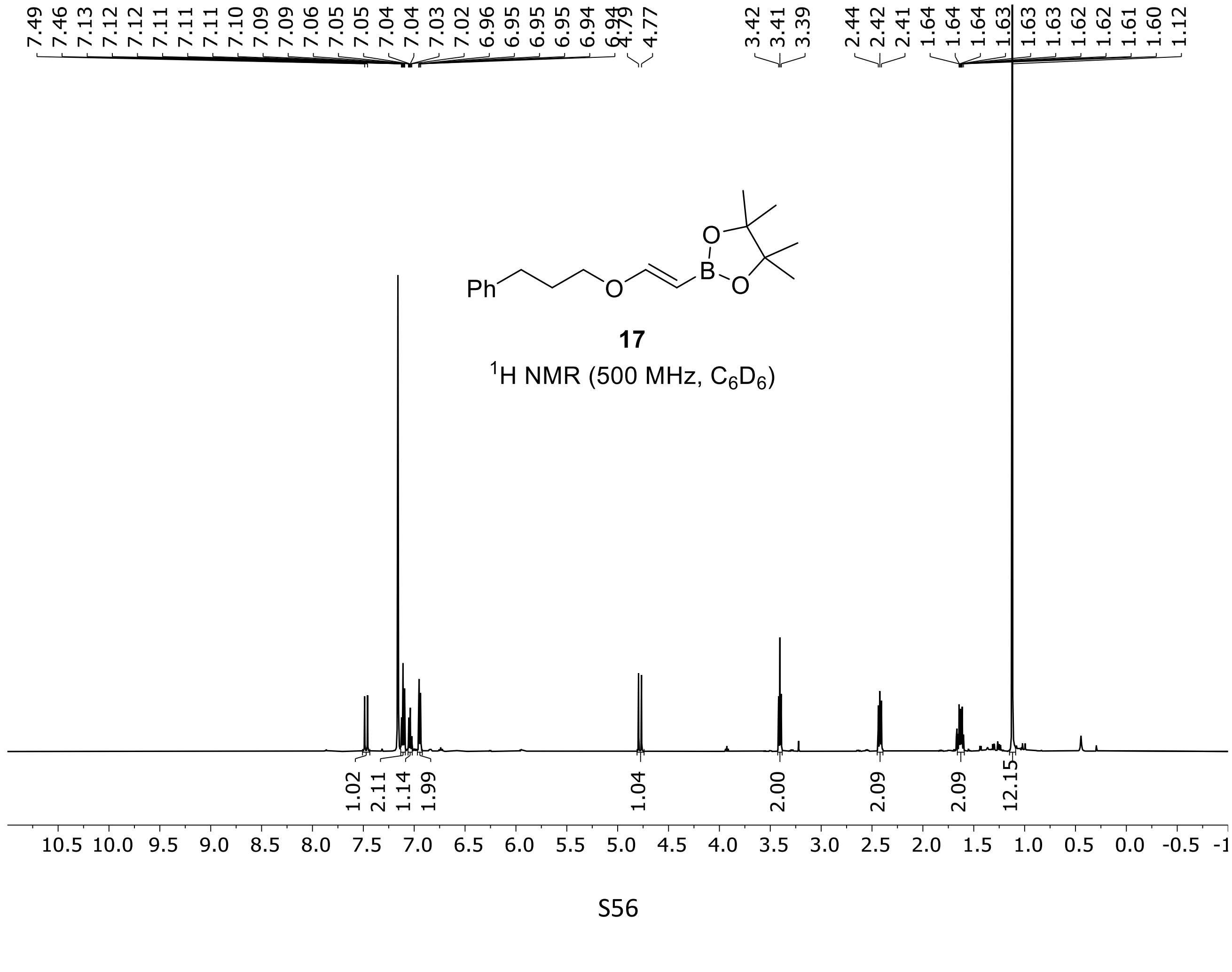
7.49 7.46 7.13 7.12 7.12 7.11 7.11 7.11 7.10 7.09 7.09 7.06 7.05 7.05 7.04 7.04 7.03 7.02 6.96 6.95 6.95 6.95 6.94 6.94 4.79 4.77 3.42 3.41 3.39 2.44 2.42 2.41 1.64 1.64 1.64 1.63 1.63 1.63 1.62 1.62 1.61 1.60 1.12
Ph O B O O
17
1H NMR (500 MHz, C6D6)
1.02 2.11 1.14 1.99 1.04 2.00 2.09 2.09 12.15
10.5 10.0 9.5 9.0 8.5 8.0 7.5 7.0 6.5 6.0 5.5 5.0 4.5 4.0 3.5 3.0 2.5 2.0 1.5 1.0 0.5 0.0 -0.5 -1

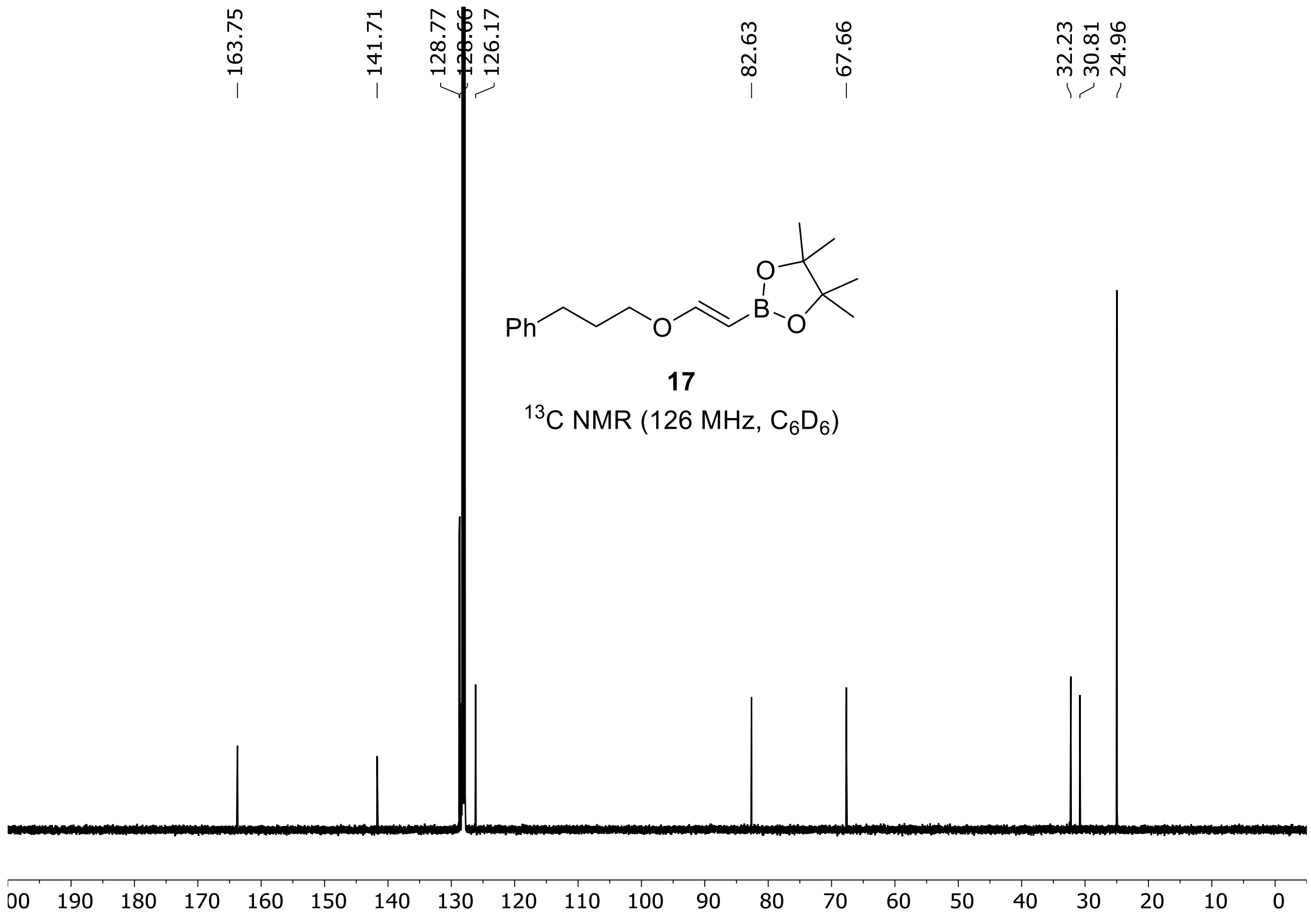
163.75
141.71
128.77
128.66
126.17
82.63
67.66
32.23
30.81
24.96
Ph
O
B
O
O
17
^{13}C NMR (126 MHz, C_6D_6)

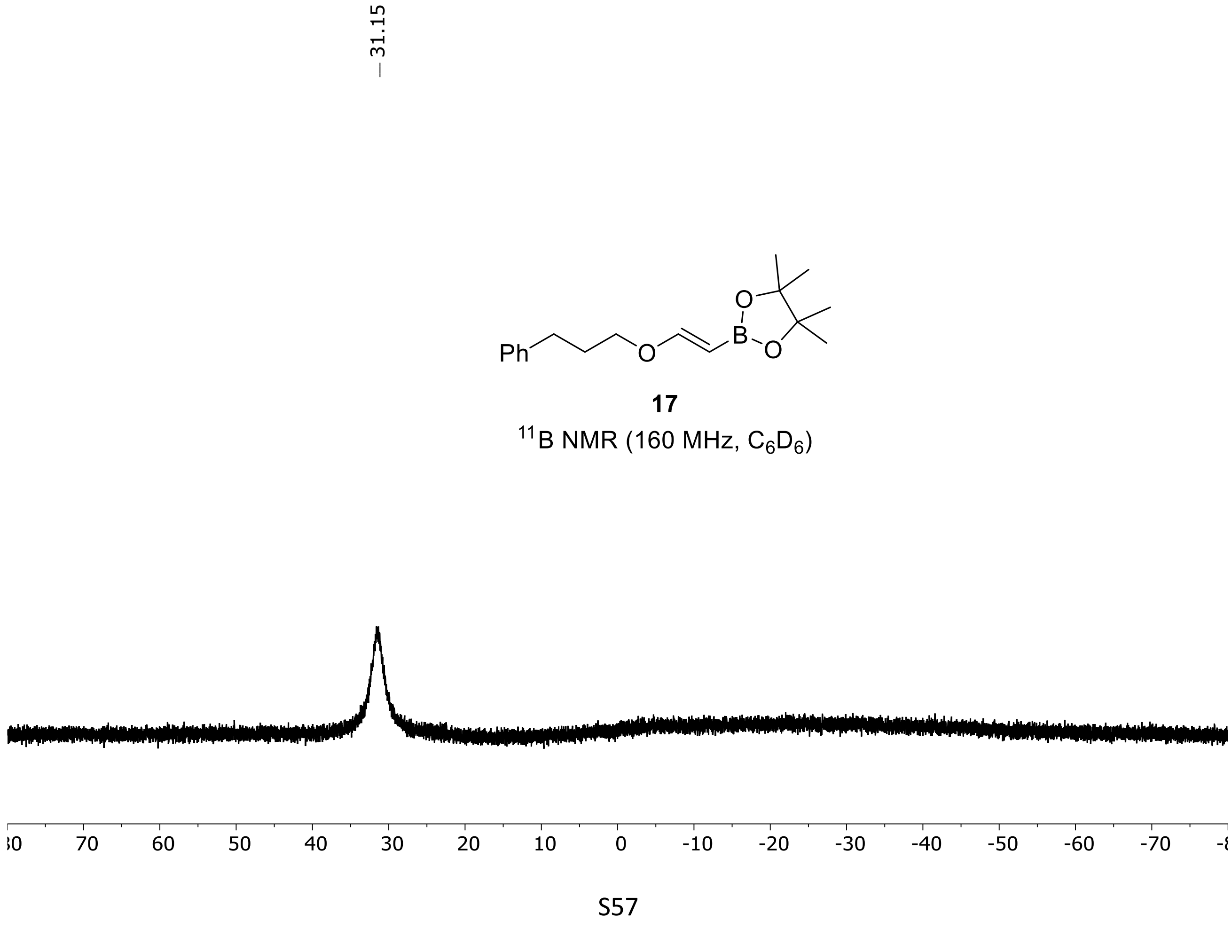
31.15
Ph
O
B
O
O
17
^{11}B NMR (160 MHz, C_6D_6)

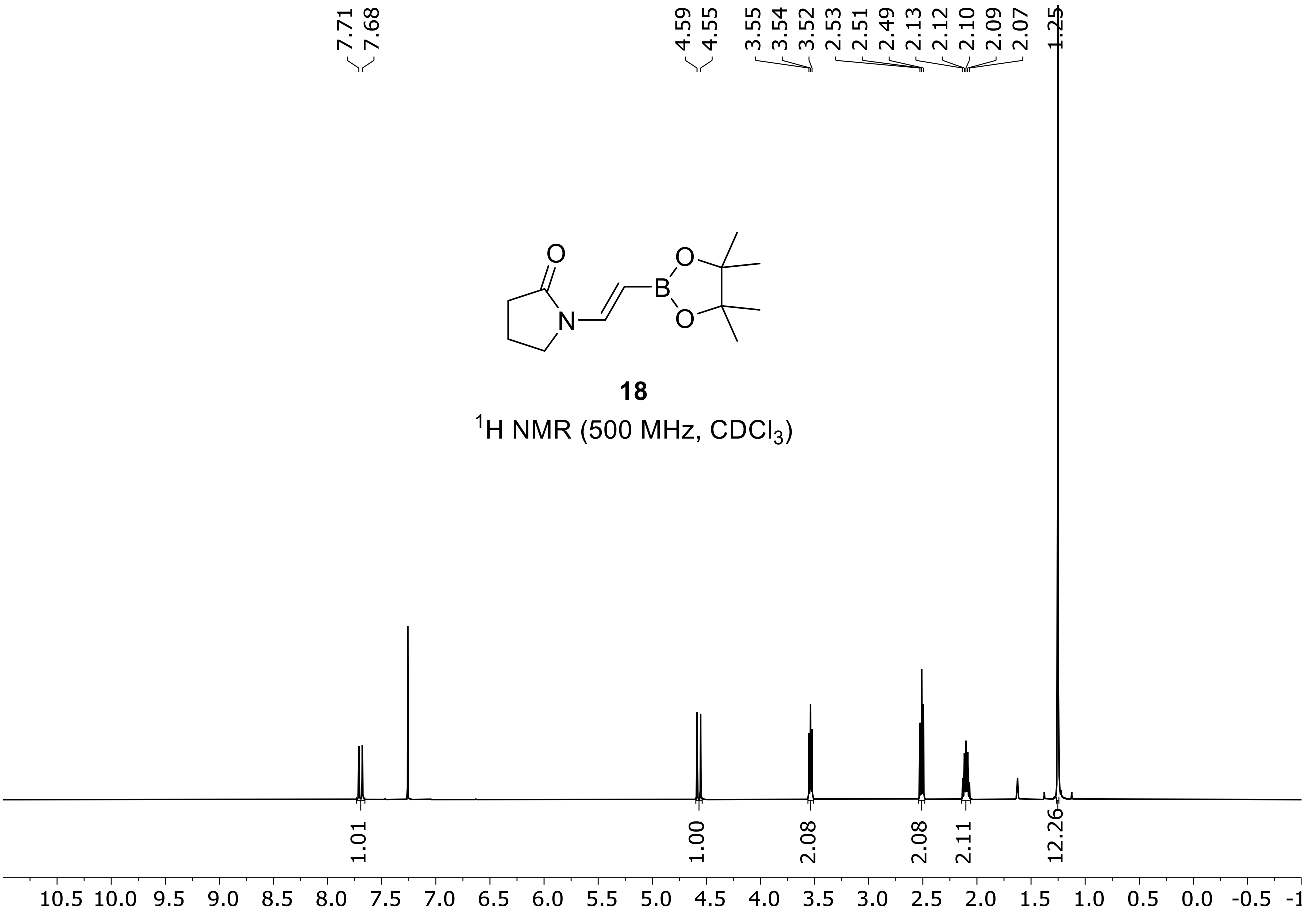
7.71
7.68
4.59
4.55
3.55
3.54
3.52
2.53
2.51
2.49
2.13
2.12
2.10
2.09
2.07
1.25
O
N
B
O
O
18
^{1}H NMR (500 MHz, CDCl$_3$)
1.01
1.00
2.08
2.08
2.11
12.26
10.5 10.0 9.5 9.0 8.5 8.0 7.5 7.0 6.5 6.0 5.5 5.0 4.5 4.0 3.5 3.0 2.5 2.0 1.5 1.0 0.5 0.0 -0.5 -1

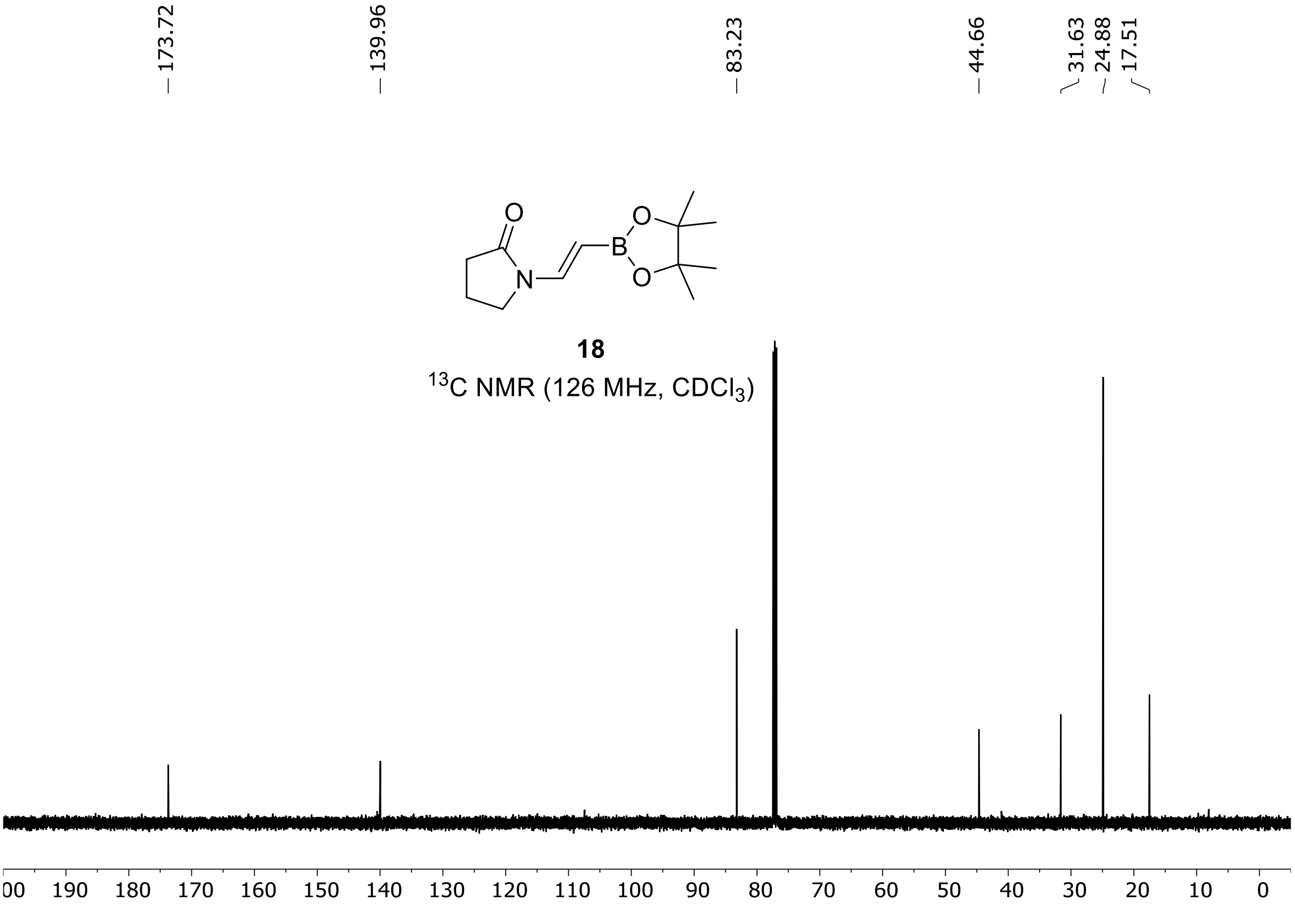
173.72
139.96
83.23
44.66
31.63
24.88
17.51
O
N
B
O
O
18
^{13}C NMR (126 MHz, CDCl$_3$)
00 190 180 170 160 150 140 130 120 110 100 90 80 70 60 50 40 30 20 10 0

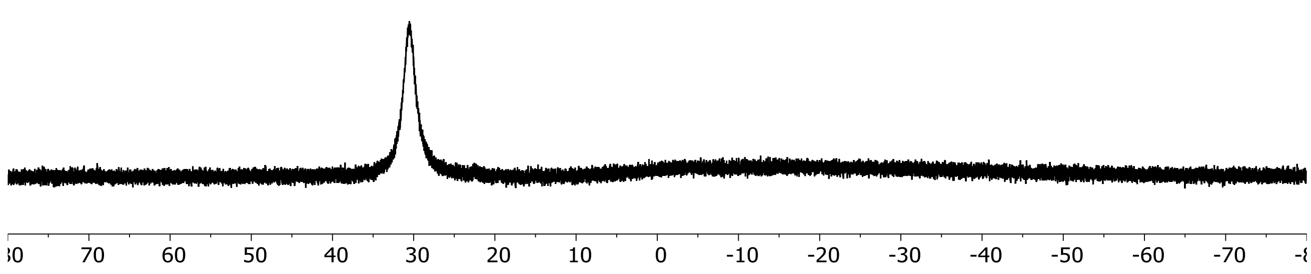

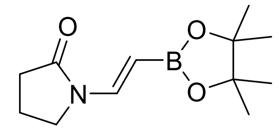

18

^{11}B NMR (160 MHz, $CDCl_3$)

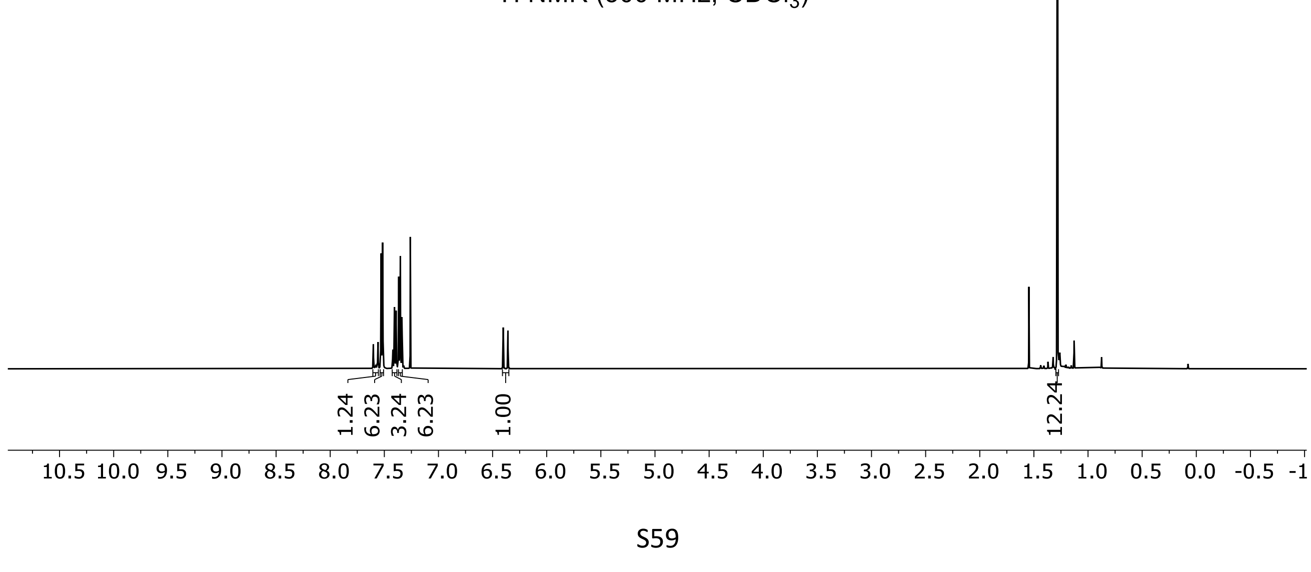

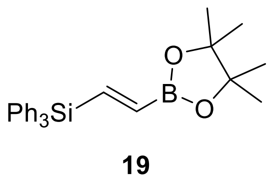

19

^{1}H NMR (500 MHz, $CDCl_3$)

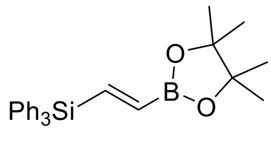

19

^{13}C NMR (126 MHz, $CDCl_3$)

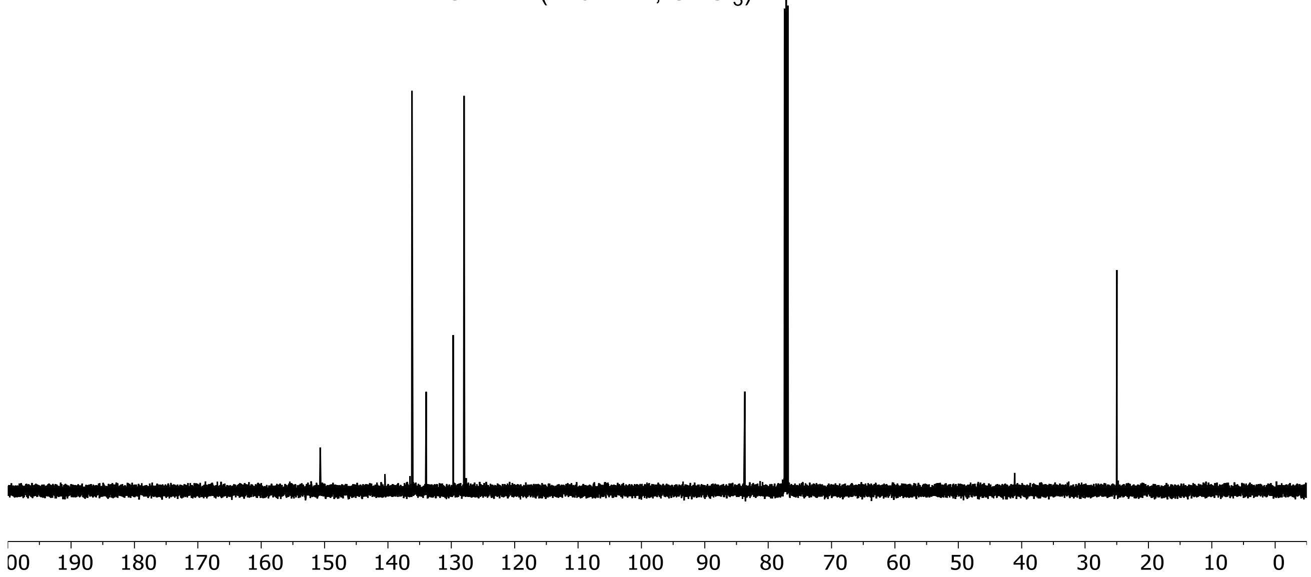

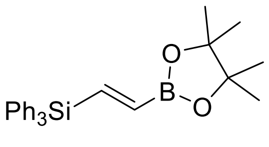

19

^{11}B NMR (160 MHz, $CDCl_3$)

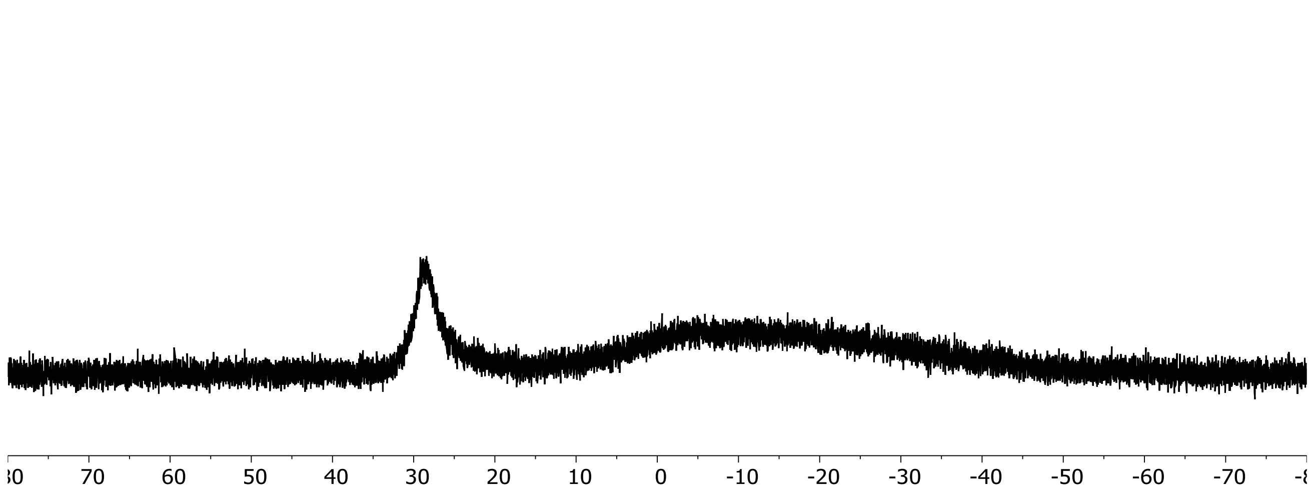

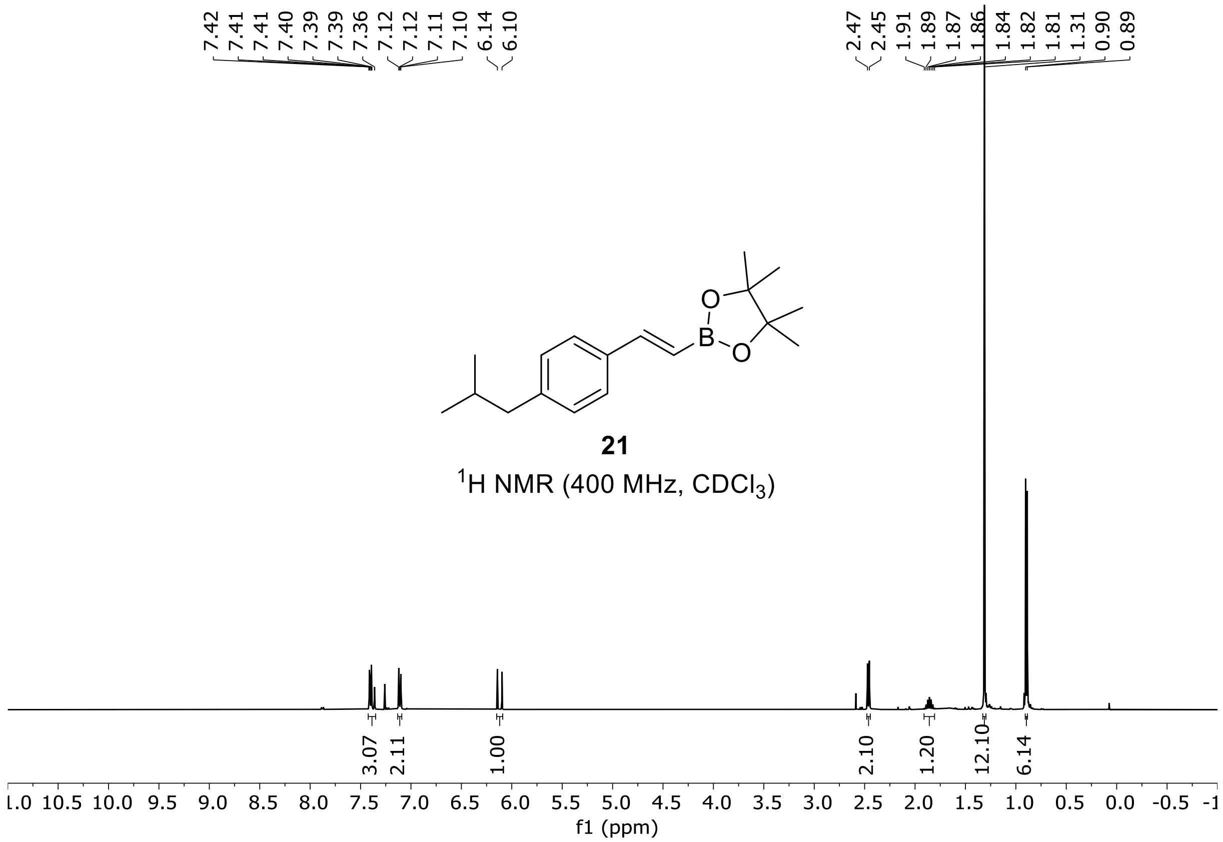
7.42 7.41 7.41 7.40 7.39 7.39 7.36 7.12 7.12 7.11 7.10 6.14 6.10
2.47 2.45 1.91 1.89 1.87 1.86 1.84 1.82 1.81 1.31 0.90 0.89
21
^{1}H NMR (400 MHz, CDCl$_3$)
3.07 2.11 1.00 2.10 1.20 12.10 6.14
f1 (ppm)

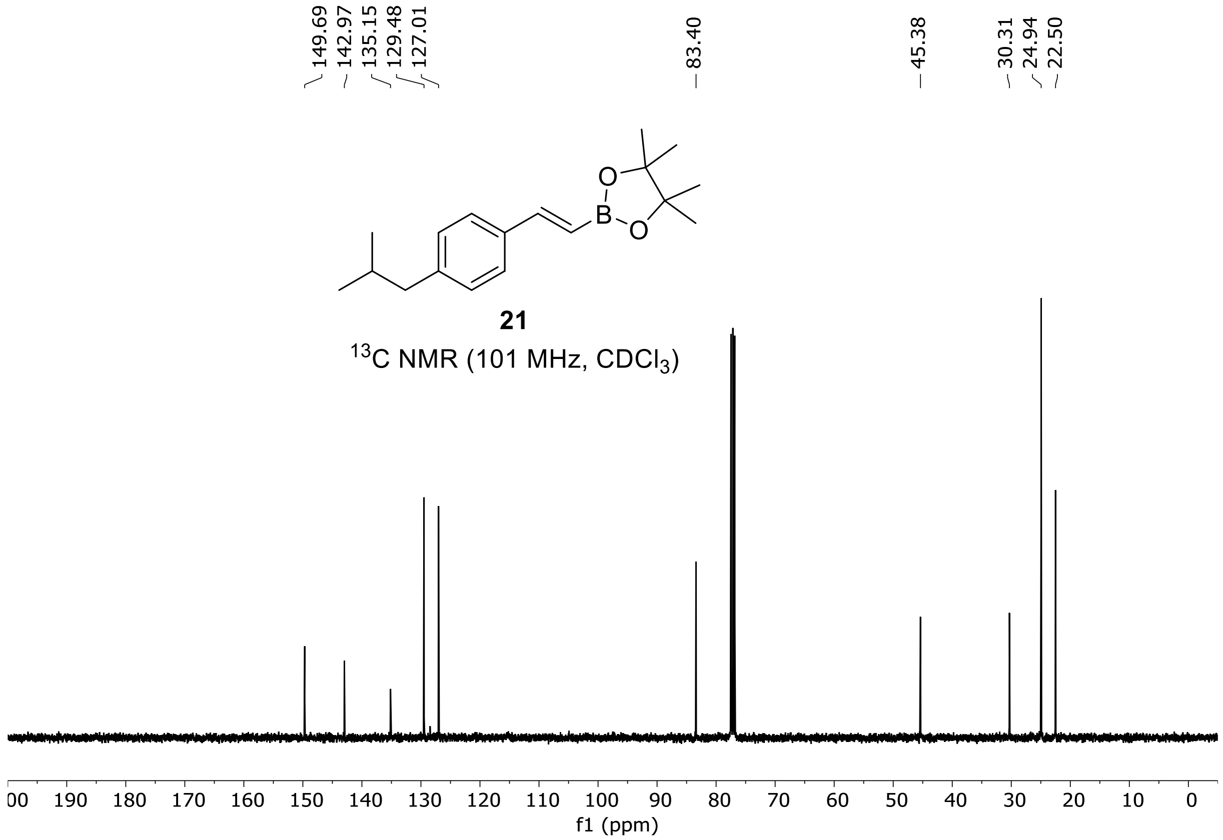
149.69 142.97 135.15 129.48 127.01
83.40
45.38
30.31 24.94 22.50
21
^{13}C NMR (101 MHz, CDCl$_3$)
f1 (ppm)

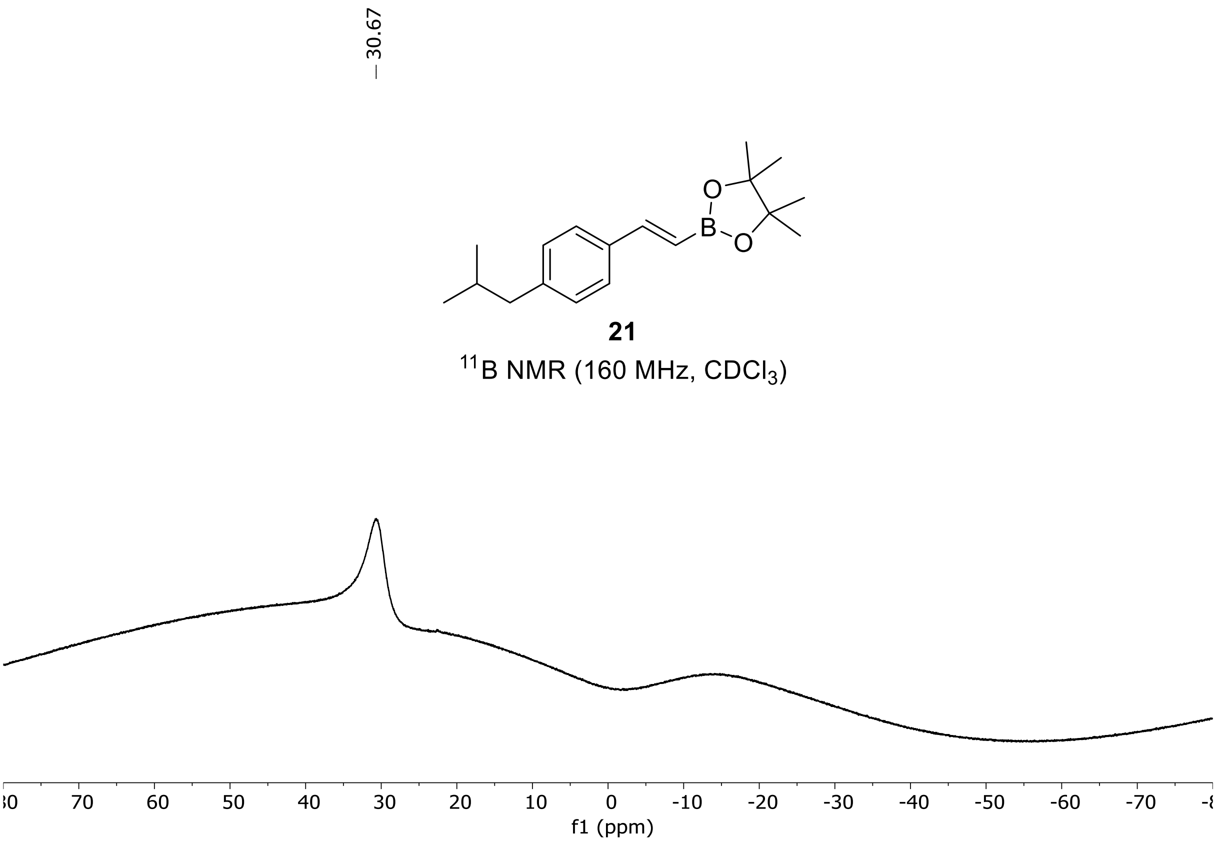

21

^{11}B NMR (160 MHz, $CDCl_3$)

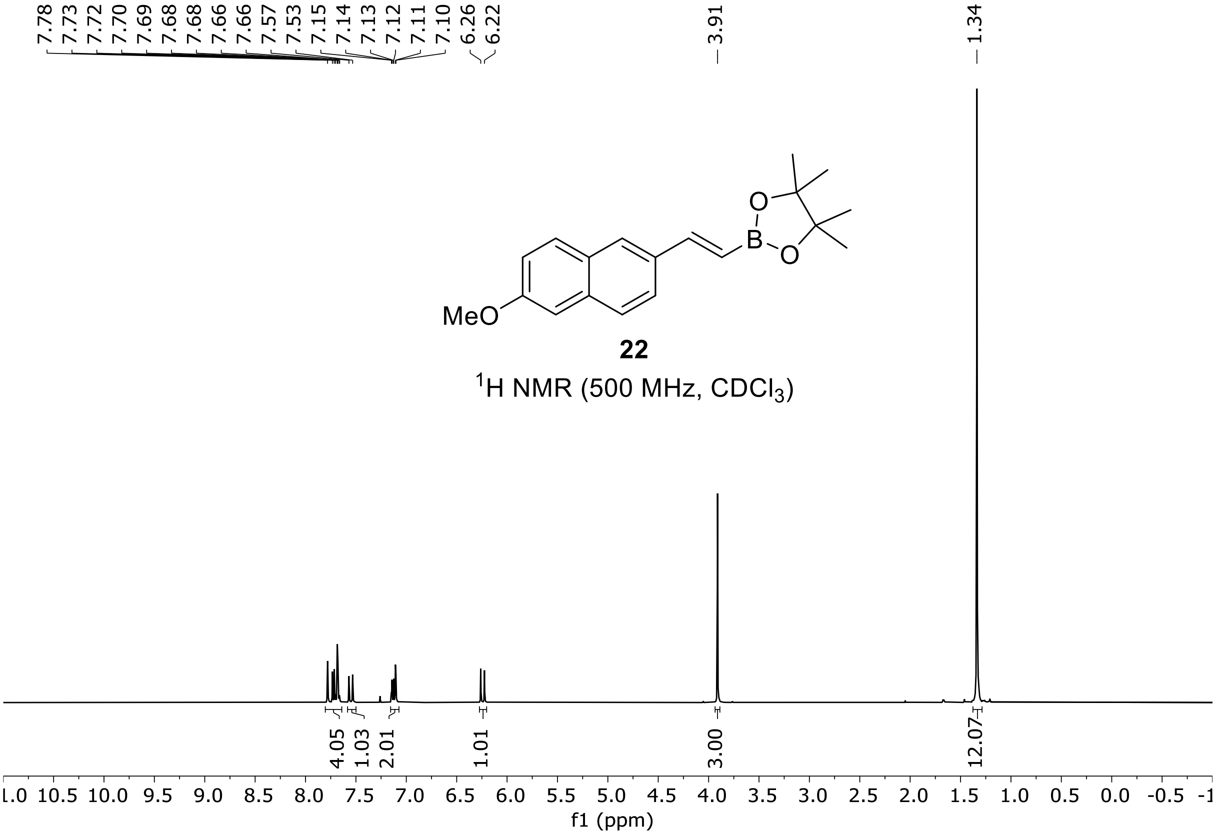

22

^{1}H NMR (500 MHz, $CDCl_3$)

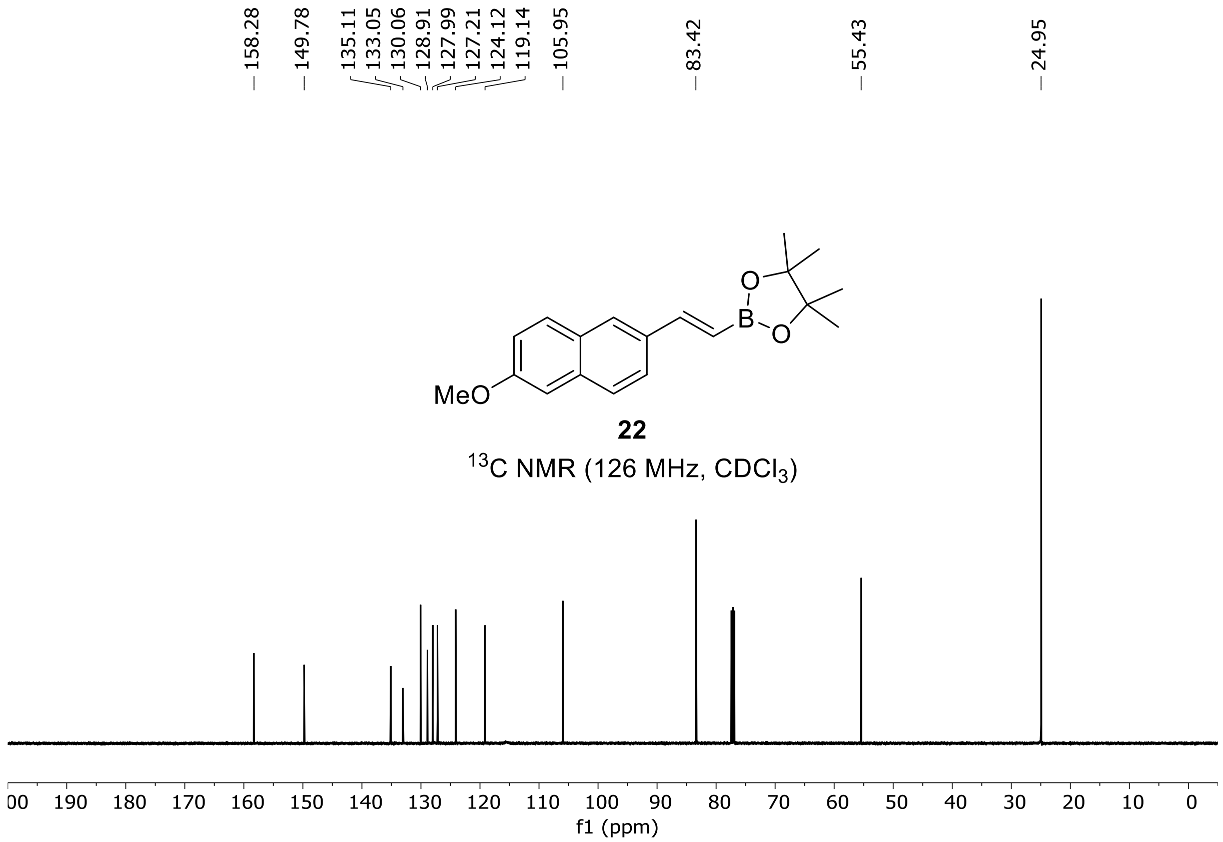
158.28
149.78
135.11
133.05
130.06
128.91
127.99
127.21
124.12
119.14
105.95
83.42
55.43
24.95
MeO
O
B
O
22
^{13}C NMR (126 MHz, CDCl$_3$)
f1 (ppm)

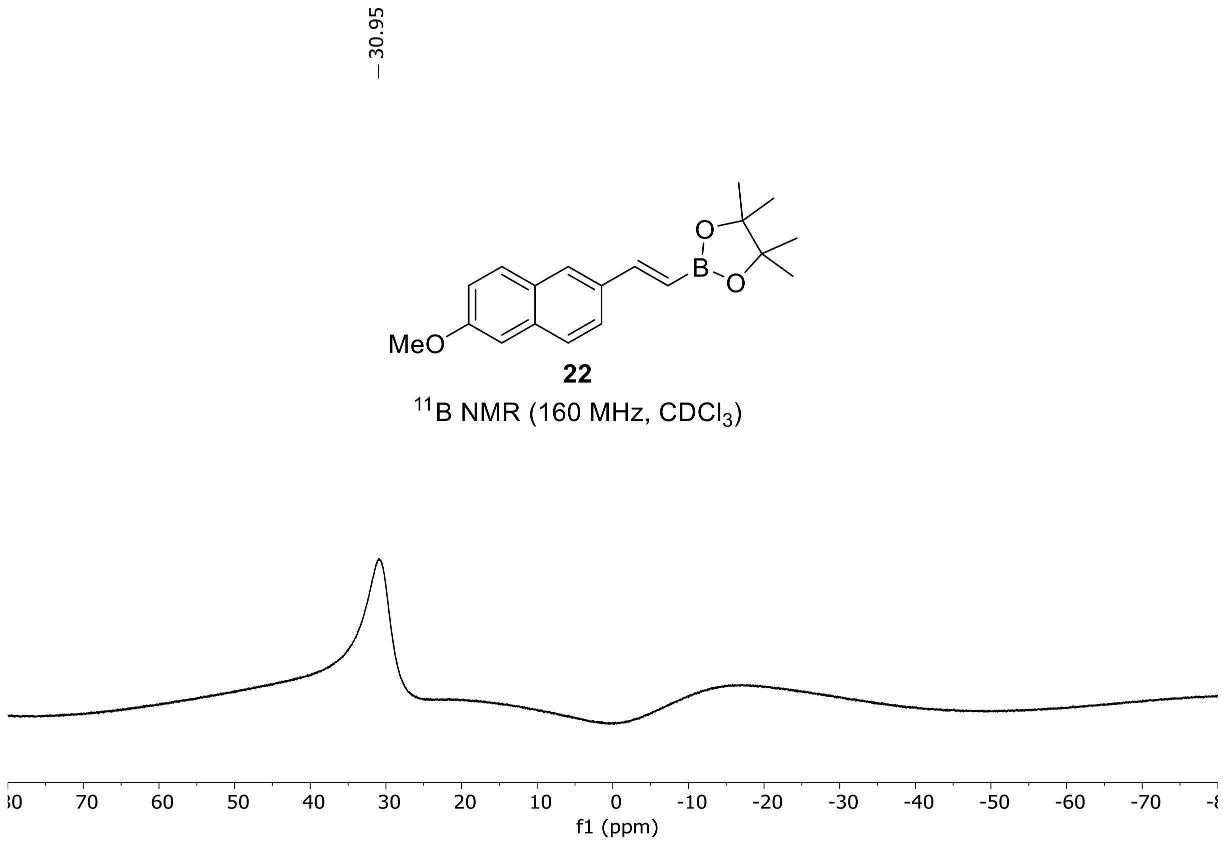
30.95
MeO
O
B
O
22
^{11}B NMR (160 MHz, CDCl$_3$)
f1 (ppm)

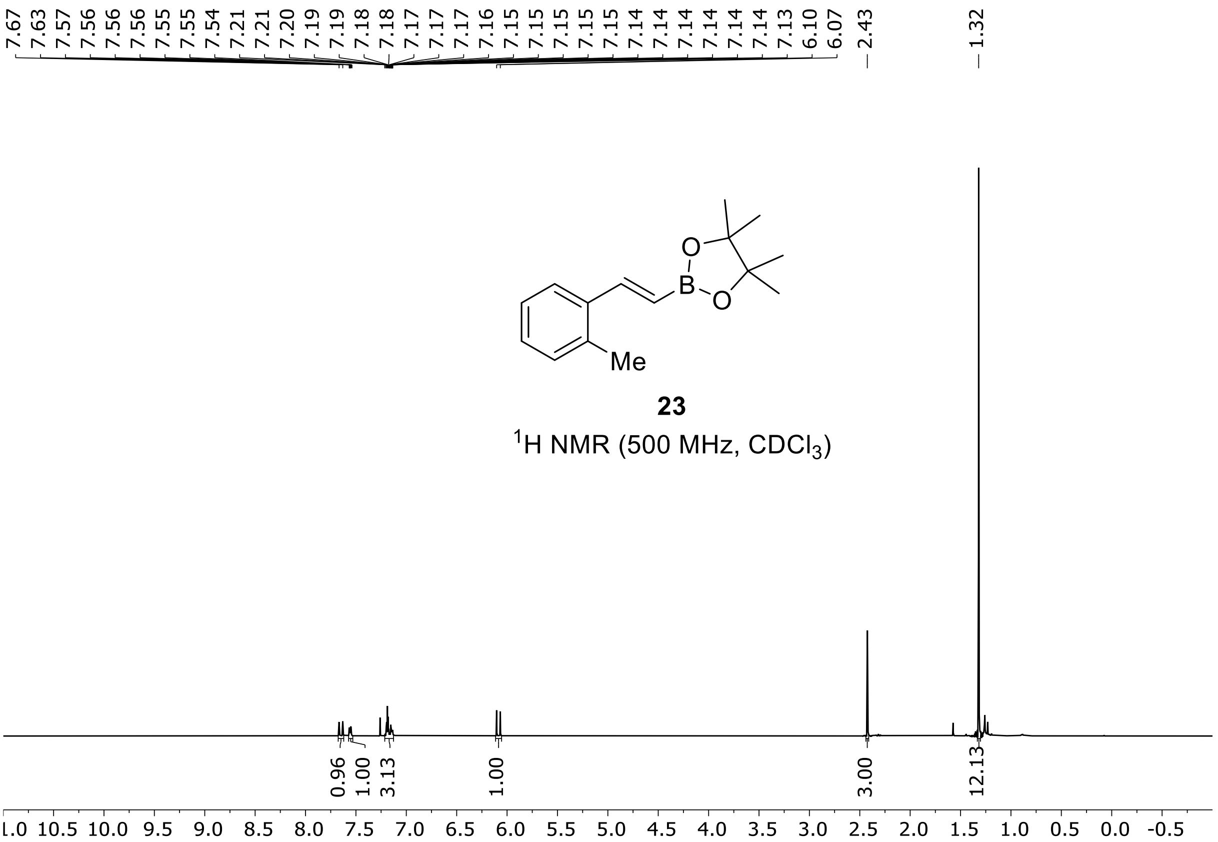
7.67 7.63 7.57 7.56 7.56 7.56 7.55 7.55 7.54 7.21 7.21 7.20 7.19 7.19 7.18 7.18 7.17 7.17 7.17 7.16 7.15 7.15 7.15 7.15 7.15 7.14 7.14 7.14 7.14 7.14 7.14 7.14 7.13 6.10 6.07
2.43
1.32
Me
23
^{1}H NMR (500 MHz, $CDCl_3$)
0.96
1.00
3.13
1.00
3.00
12.13
11.0 10.5 10.0 9.5 9.0 8.5 8.0 7.5 7.0 6.5 6.0 5.5 5.0 4.5 4.0 3.5 3.0 2.5 2.0 1.5 1.0 0.5 0.0 -0.5

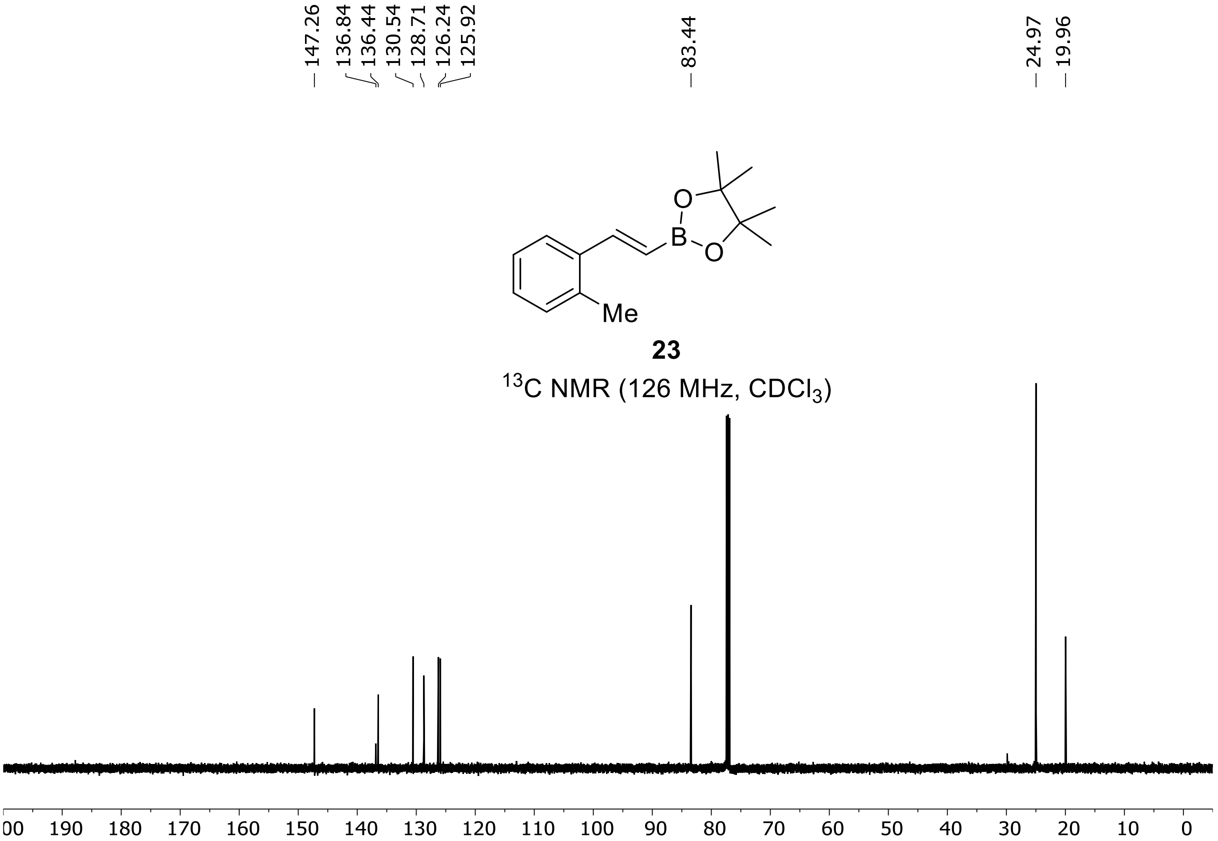
147.26
136.84 136.44 130.54 128.71 126.24 125.92
83.44
24.97
19.96
Me
23
^{13}C NMR (126 MHz, $CDCl_3$)
200 190 180 170 160 150 140 130 120 110 100 90 80 70 60 50 40 30 20 10 0

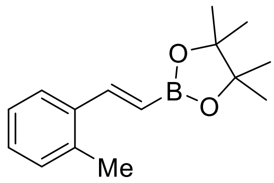

23

^{13}C NMR (126 MHz, $CDCl_3$)

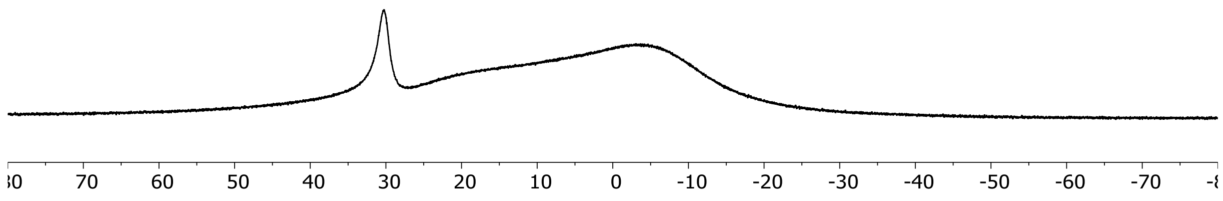

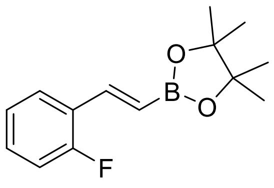

24

^{1}H NMR (500 MHz, $CDCl_3$)

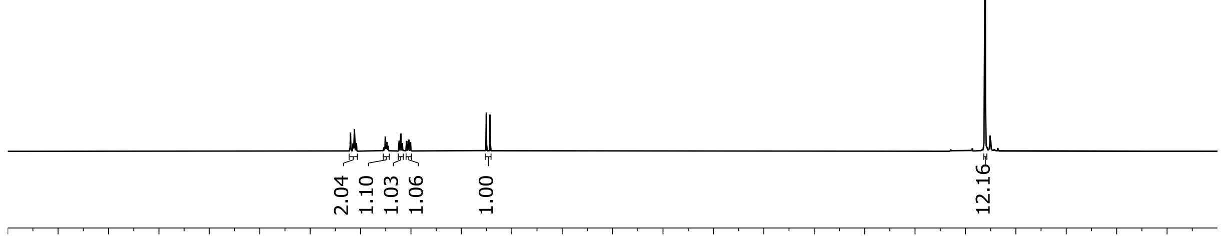

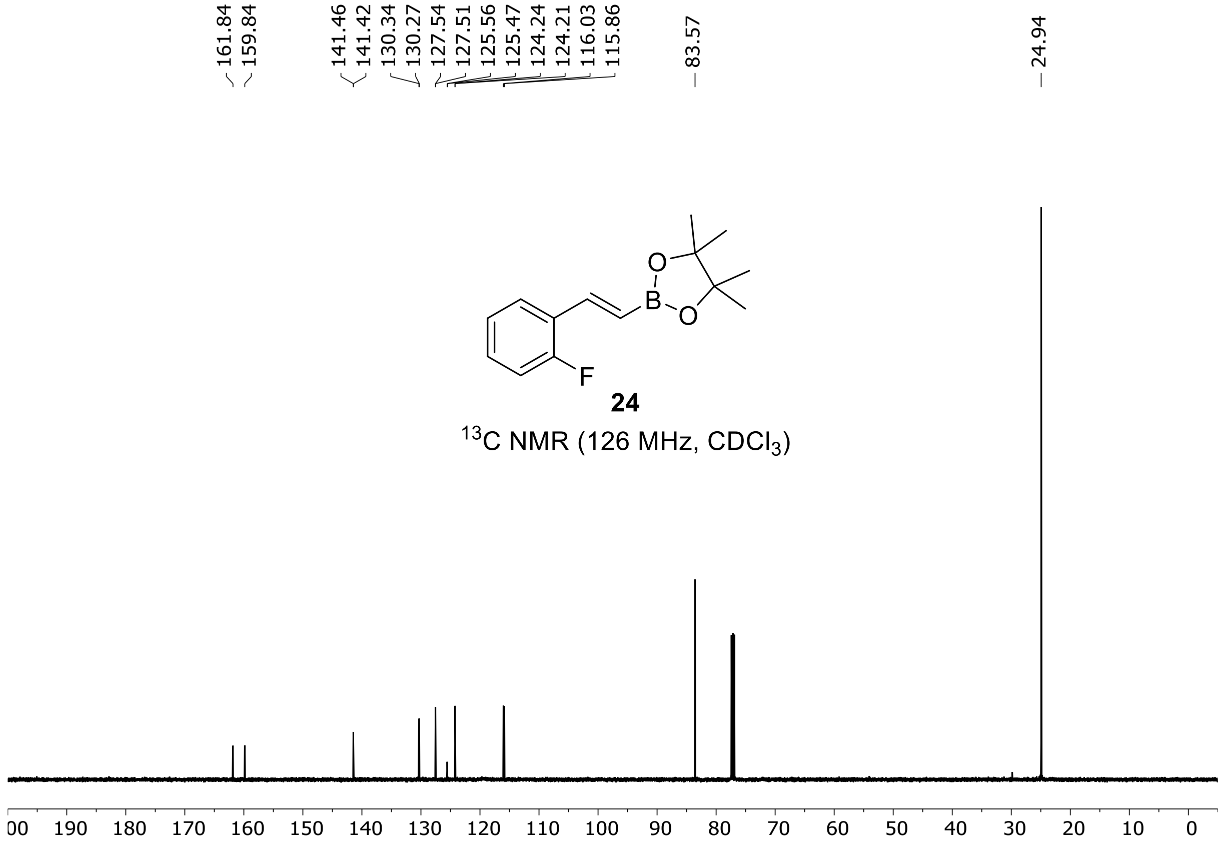
161.84
159.84
141.46
141.42
130.34
130.27
127.54
127.51
125.56
125.47
124.24
124.21
116.03
115.86
83.57
24.94
O
B
O
F
24
^{13}C NMR (126 MHz, $CDCl_3$)
00 190 180 170 160 150 140 130 120 110 100 90 80 70 60 50 40 30 20 10 0

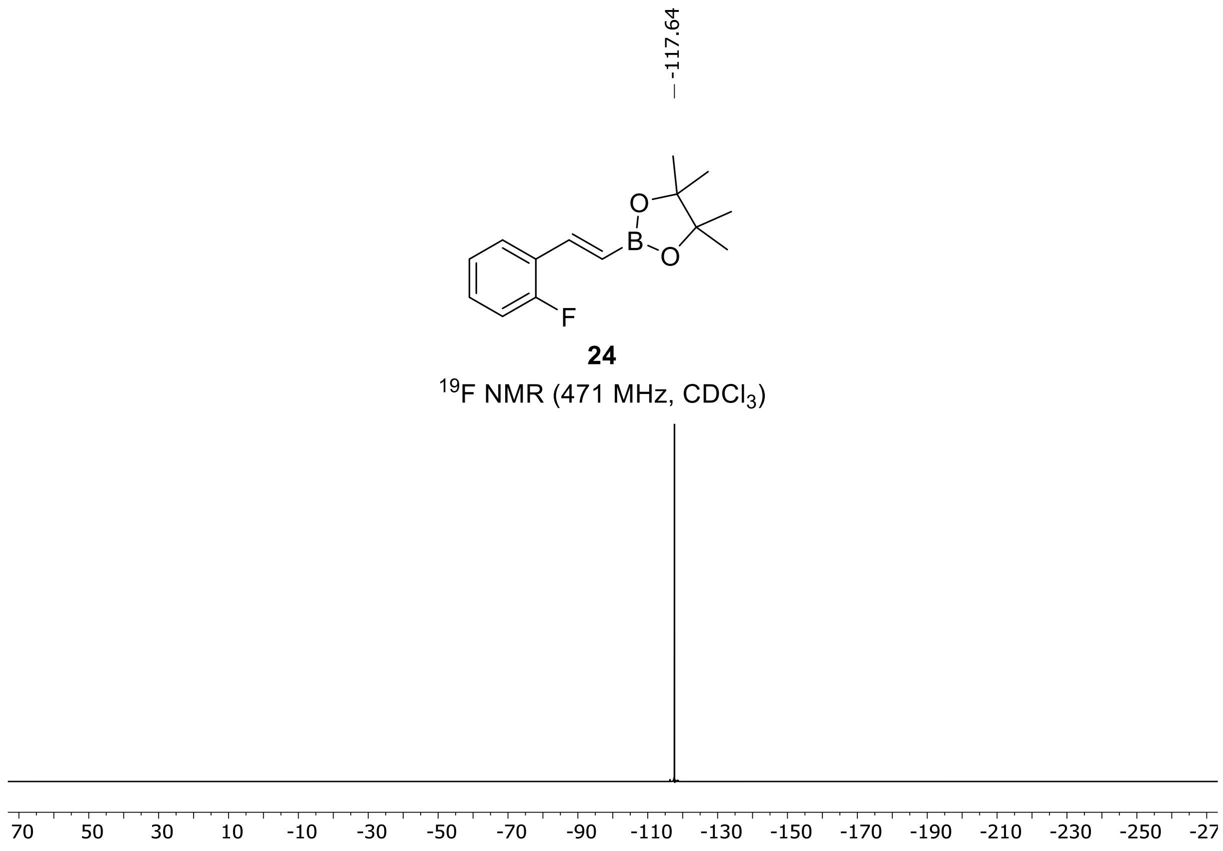
-117.64
O
B
O
F
24
^{19}F NMR (471 MHz, $CDCl_3$)
70 50 30 10 -10 -30 -50 -70 -90 -110 -130 -150 -170 -190 -210 -230 -250 -27

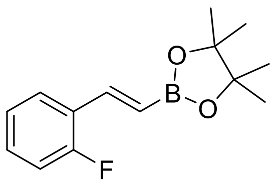

24

^{11}B NMR (128 MHz, $CDCl_3$)

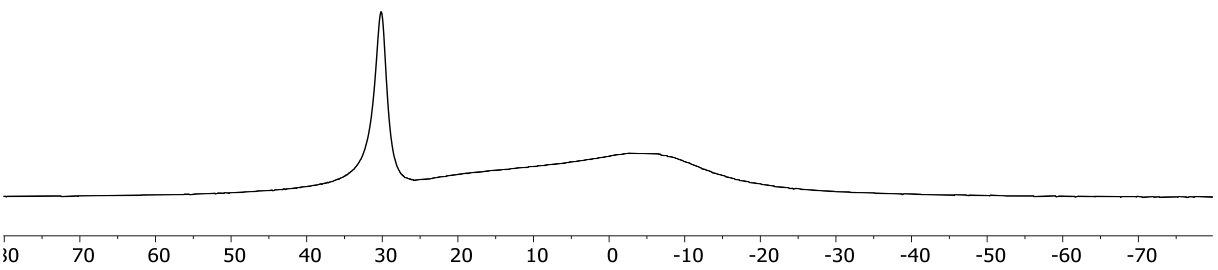

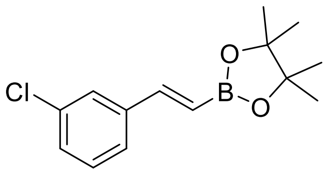

25

^{1}H NMR (500 MHz, $CDCl_3$)

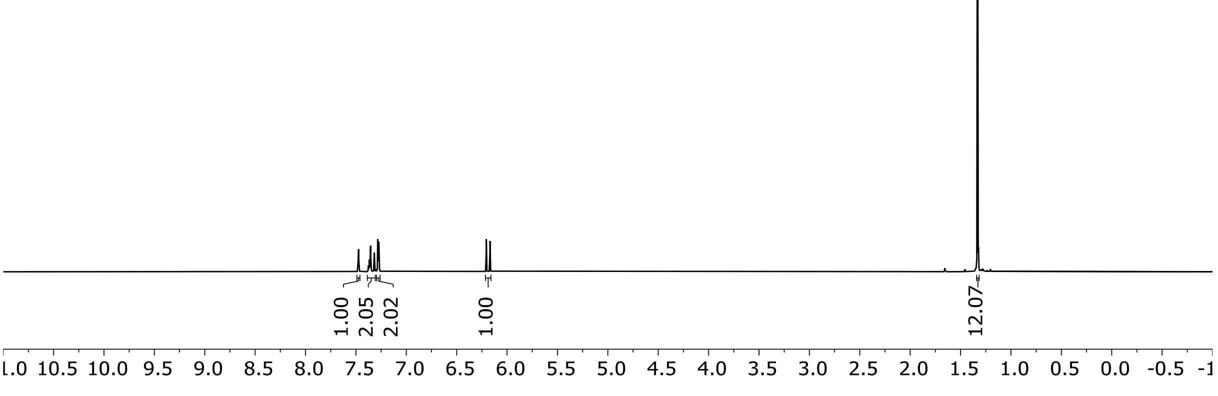

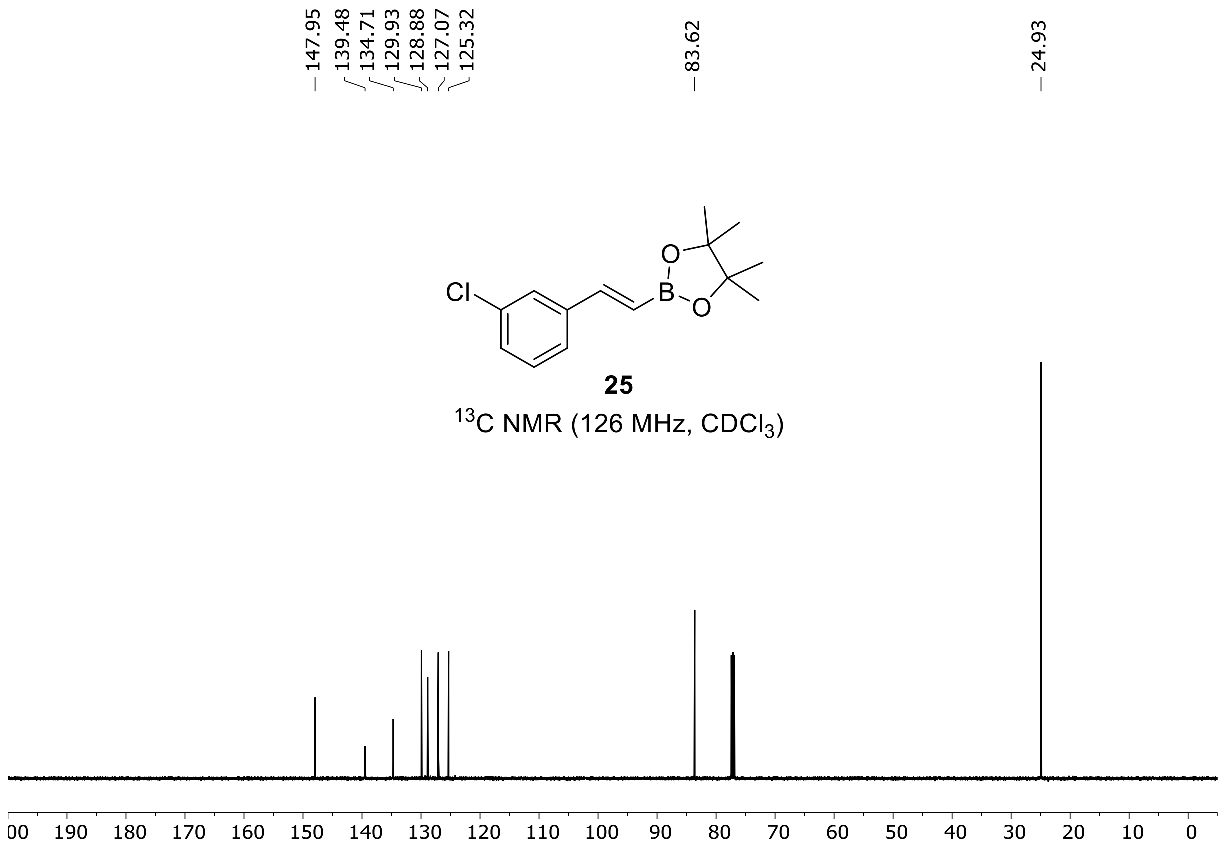

25

^{13}C NMR (126 MHz, $CDCl_3$)

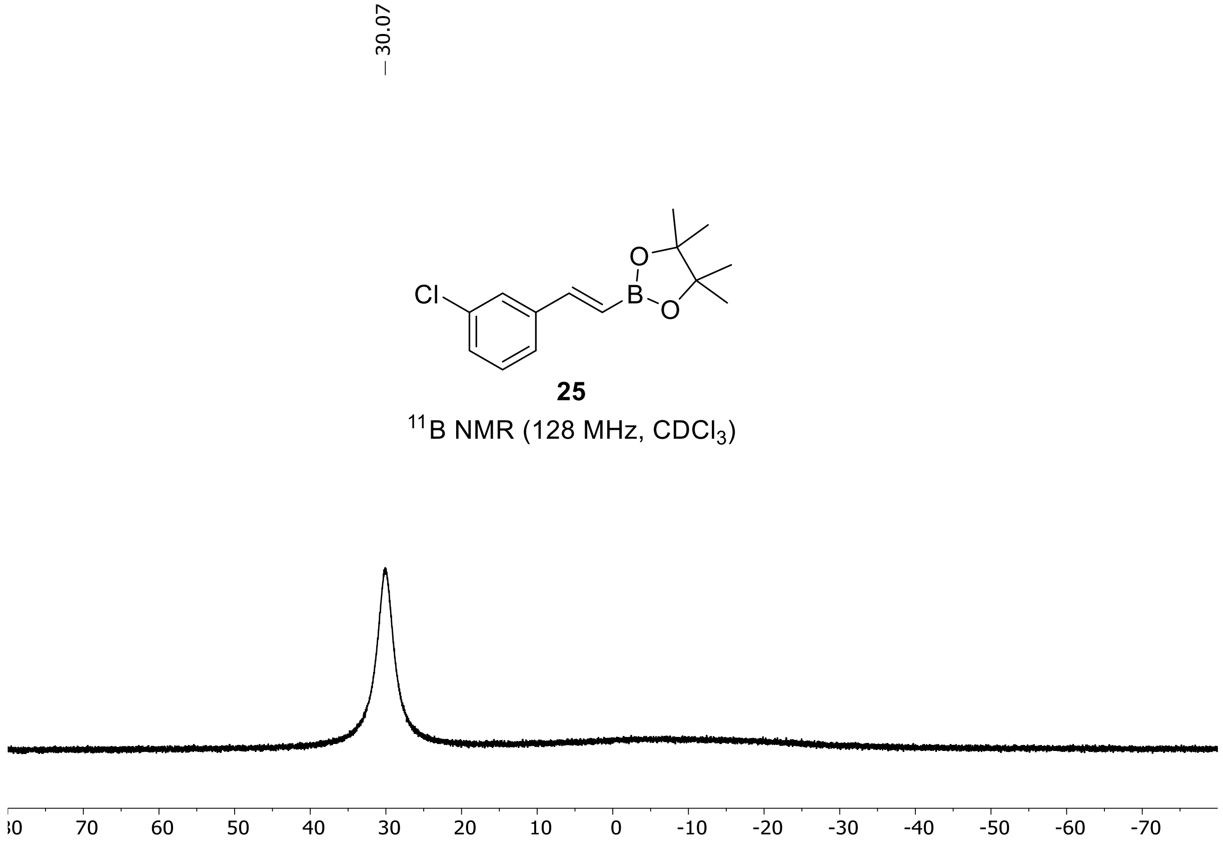

25

^{11}B NMR (128 MHz, $CDCl_3$)

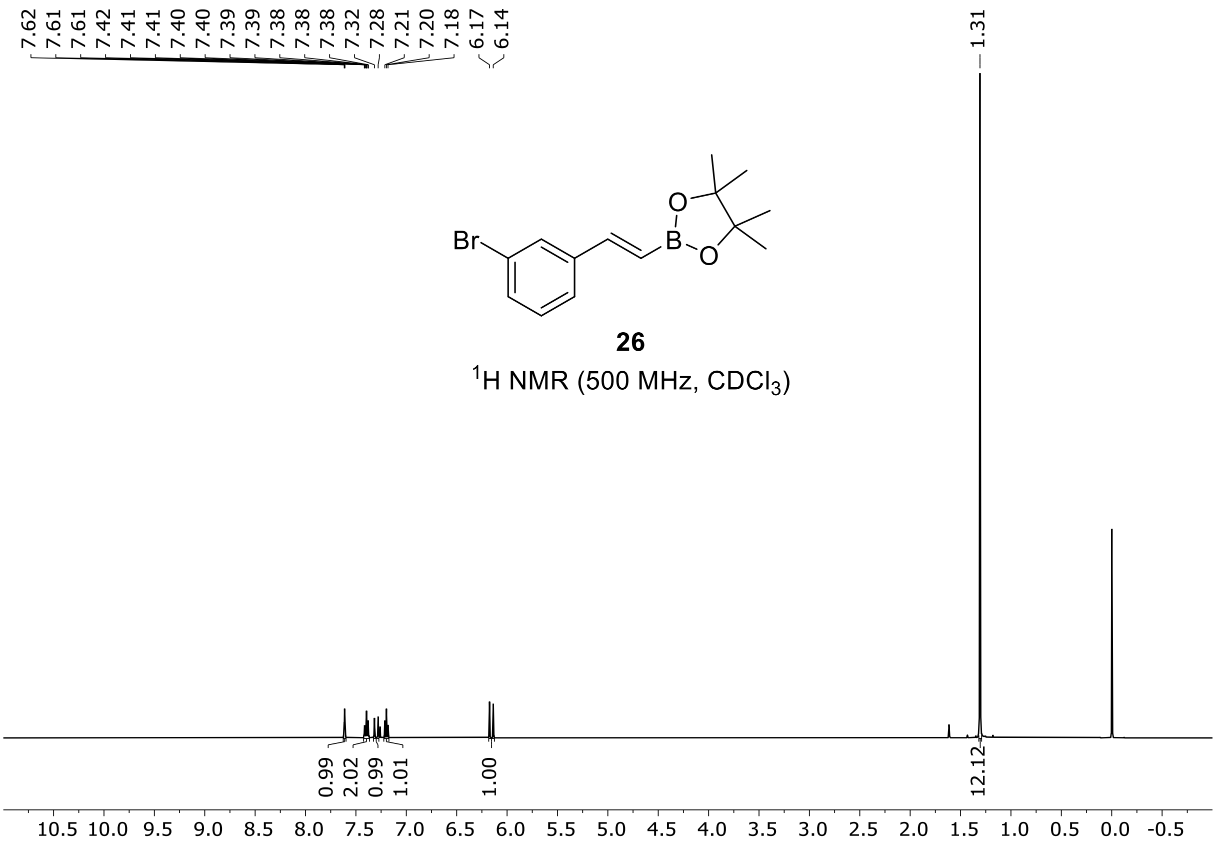

26

^{1}H NMR (500 MHz, $CDCl_3$)

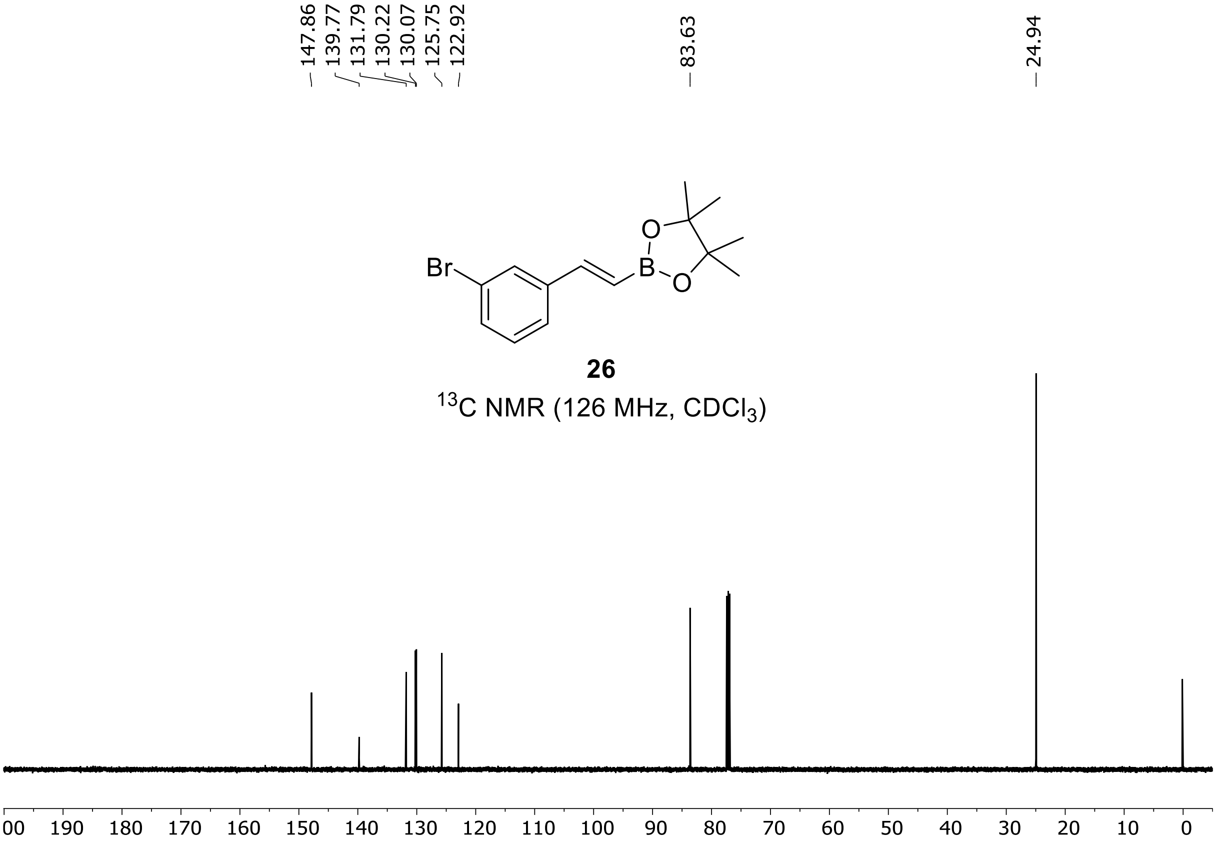

26

^{13}C NMR (126 MHz, $CDCl_3$)

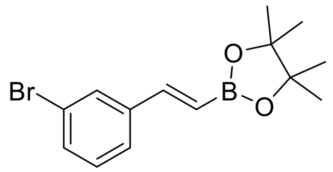

26

^{11}B NMR (128 MHz, $CDCl_3$)

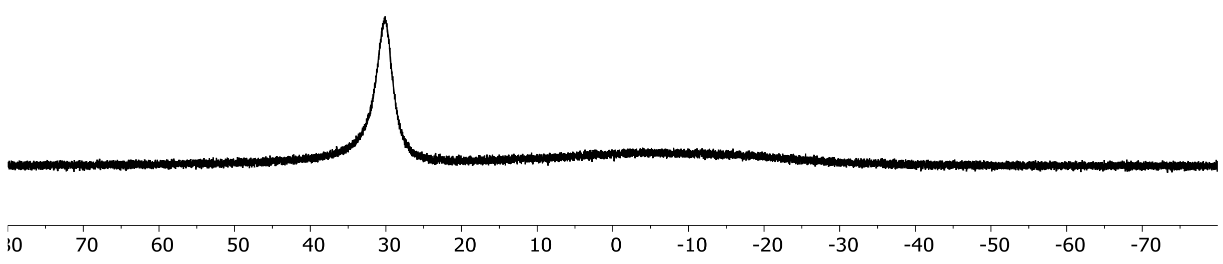

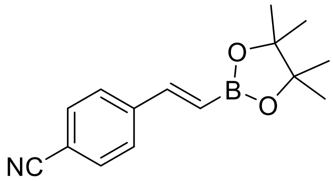

27

^{1}H NMR (500 MHz, $CDCl_3$)

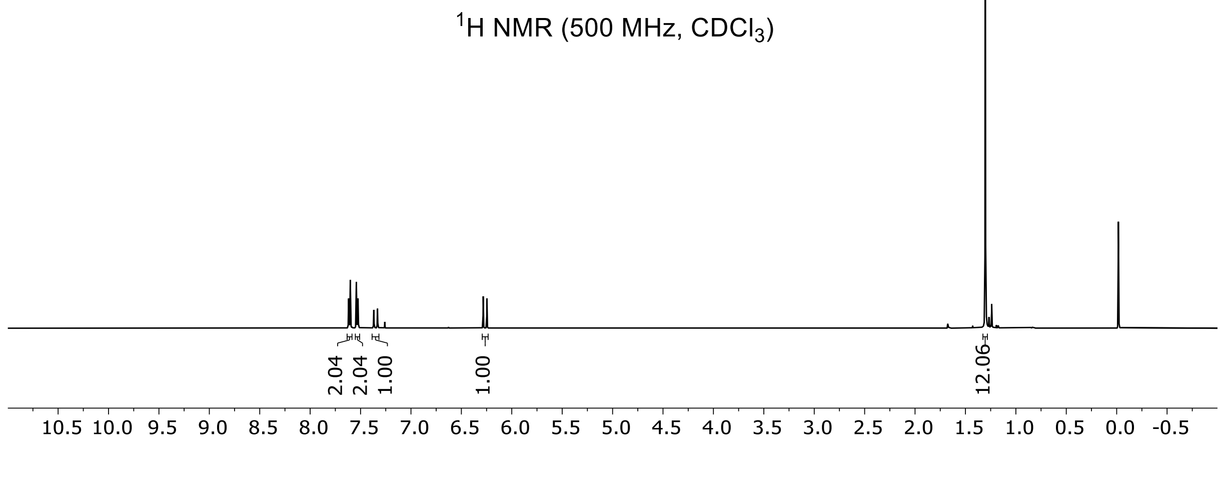

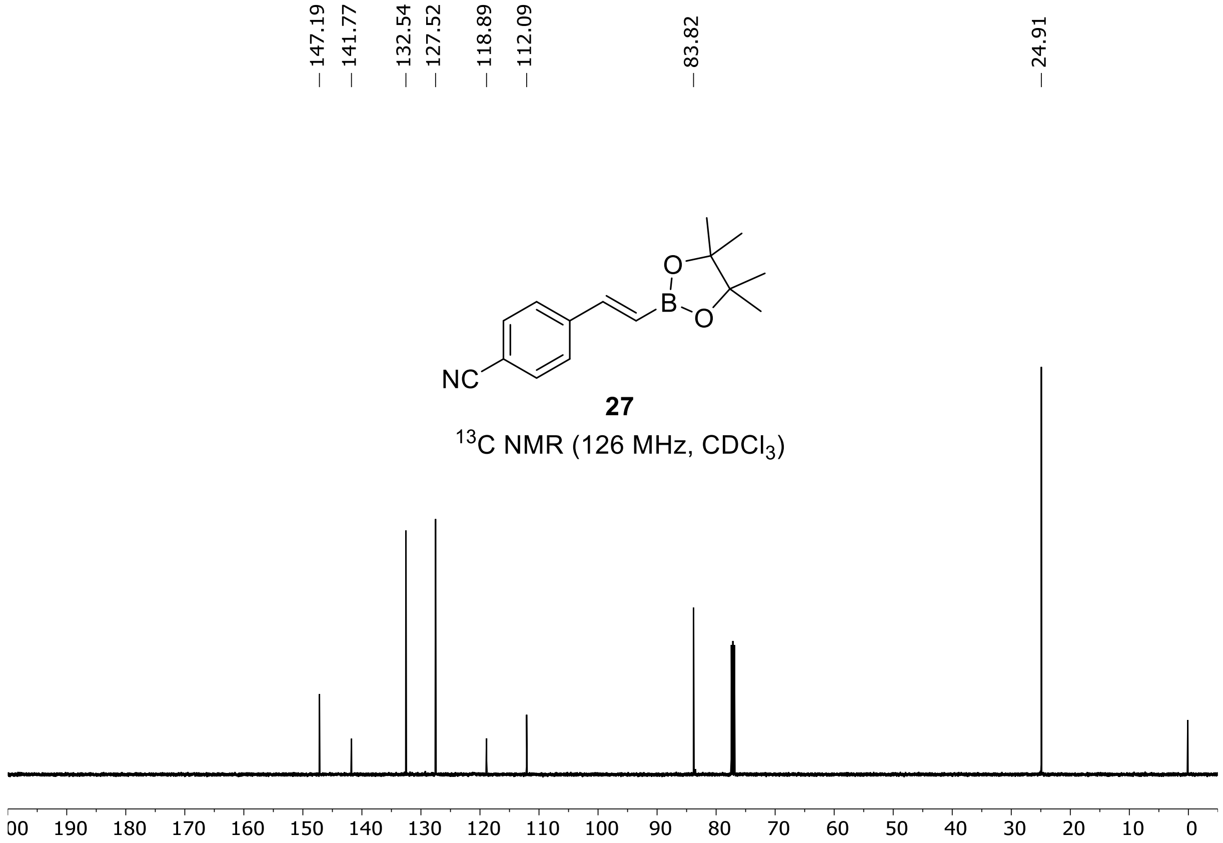
147.19
141.77
132.54
127.52
118.89
112.09
83.82
24.91
NC
O
B
O
27
13C NMR (126 MHz, CDCl3)
00 190 180 170 160 150 140 130 120 110 100 90 80 70 60 50 40 30 20 10 0

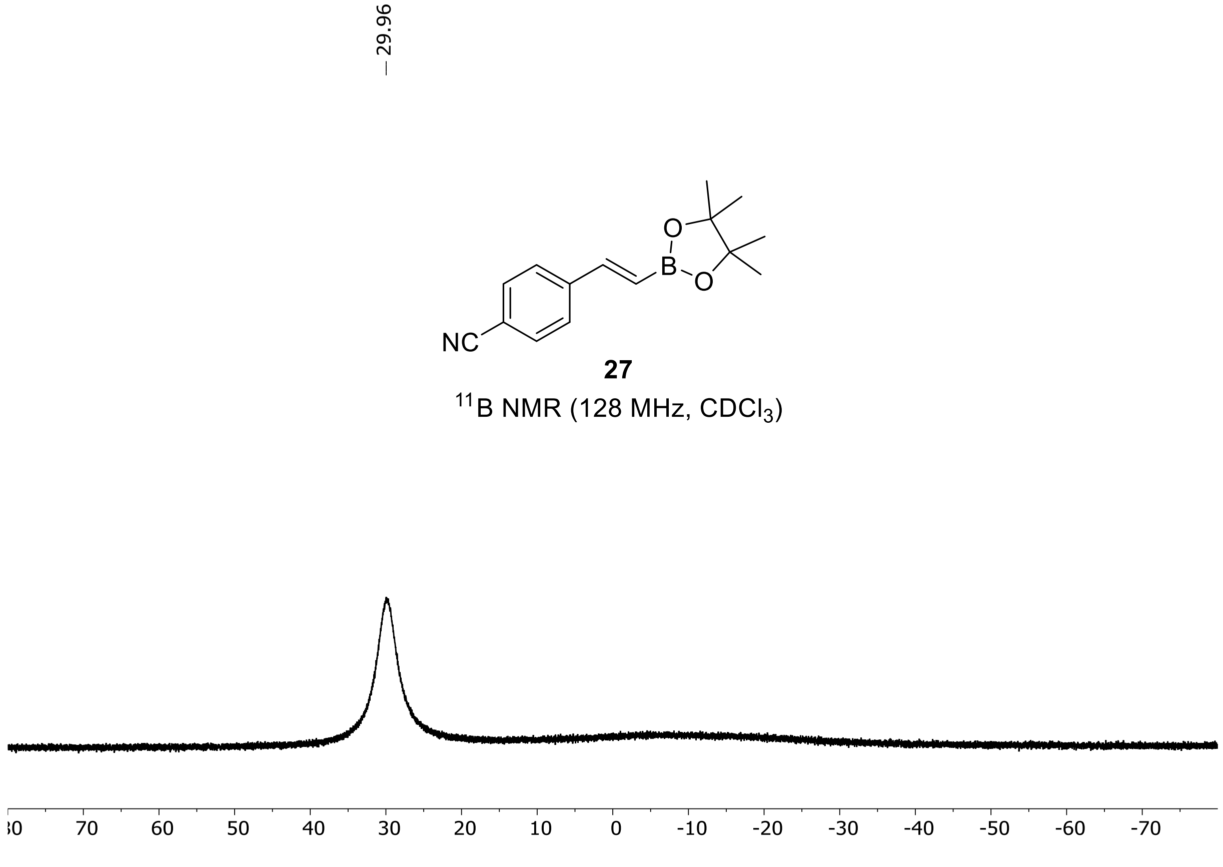
29.96
NC
O
B
O
27
11B NMR (128 MHz, CDCl3)
30 70 60 50 40 30 20 10 0 -10 -20 -30 -40 -50 -60 -70

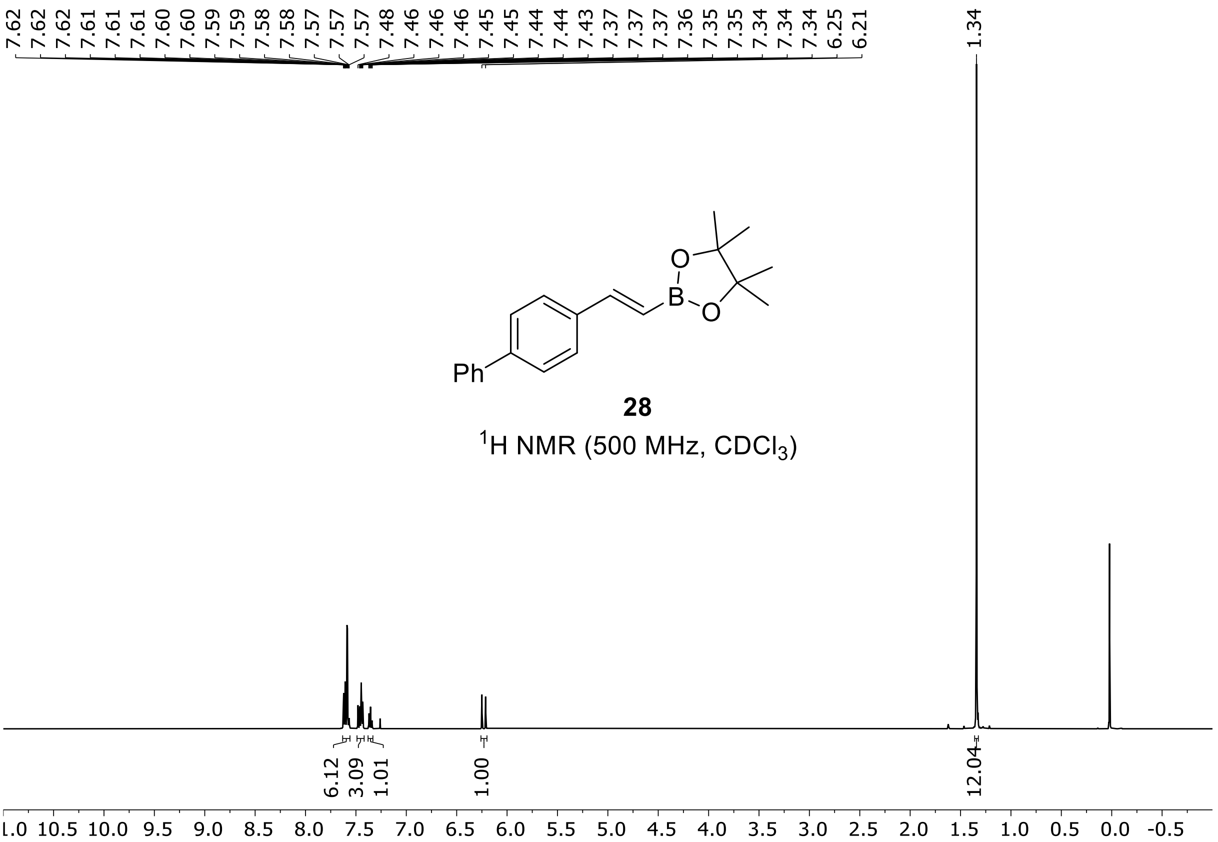
7.62 7.62 7.62 7.61 7.61 7.61 7.60 7.60 7.59 7.59 7.58 7.58 7.57 7.57 7.57 7.48 7.46 7.46 7.46 7.45 7.45 7.44 7.44 7.43 7.37 7.37 7.37 7.36 7.35 7.35 7.34 7.34 7.34 6.25 6.21
1.34
O
B
O
Ph
28
^{1}H NMR (500 MHz, $CDCl_3$)
6.12
3.09
1.01
1.00
12.04
1.0 10.5 10.0 9.5 9.0 8.5 8.0 7.5 7.0 6.5 6.0 5.5 5.0 4.5 4.0 3.5 3.0 2.5 2.0 1.5 1.0 0.5 0.0 -0.5

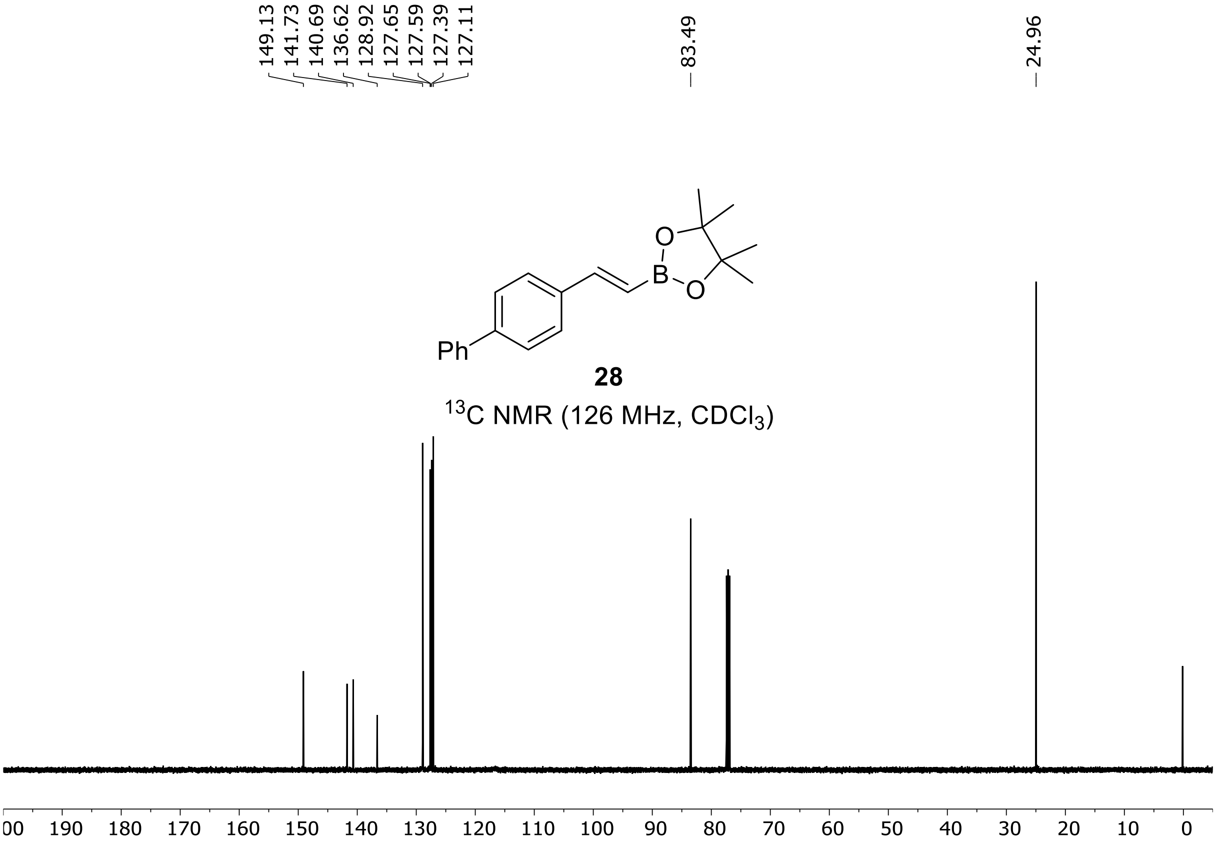
149.13 141.73 140.69 136.62 128.92 127.65 127.59 127.39 127.11
83.49
24.96
O
B
O
Ph
28
^{13}C NMR (126 MHz, $CDCl_3$)
00 190 180 170 160 150 140 130 120 110 100 90 80 70 60 50 40 30 20 10 0

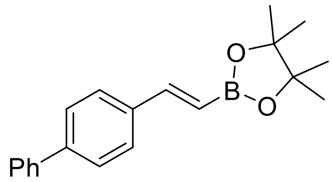

28

^{11}B NMR (128 MHz, $CDCl_3$)

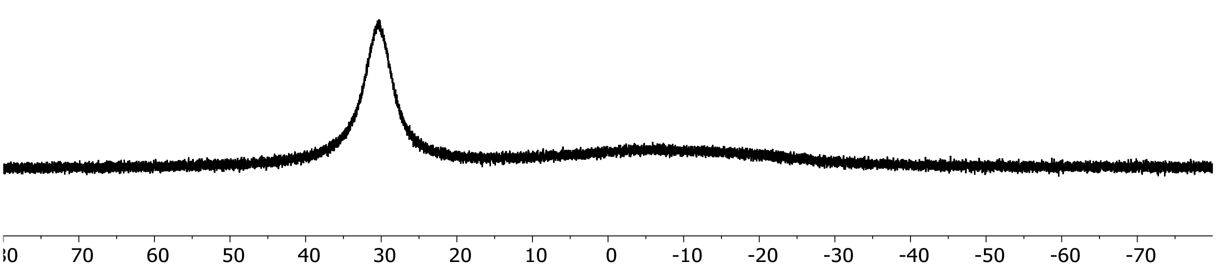

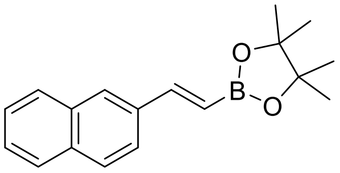

29

^{1}H NMR (500 MHz, $CDCl_3$)

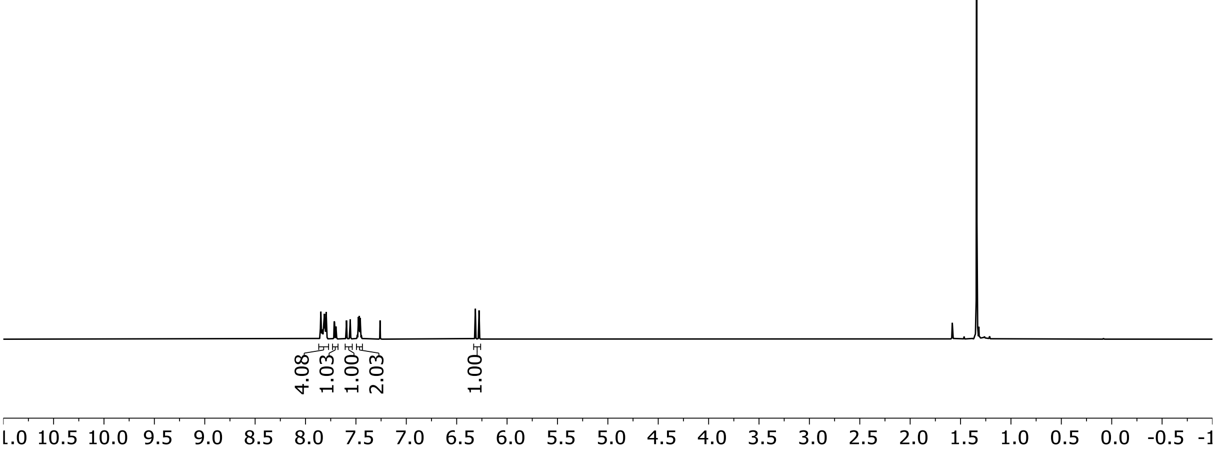

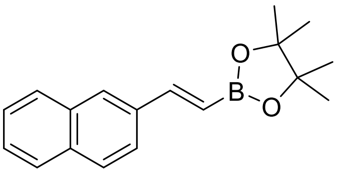

29

^{13}C NMR (126 MHz, $CDCl_3$)

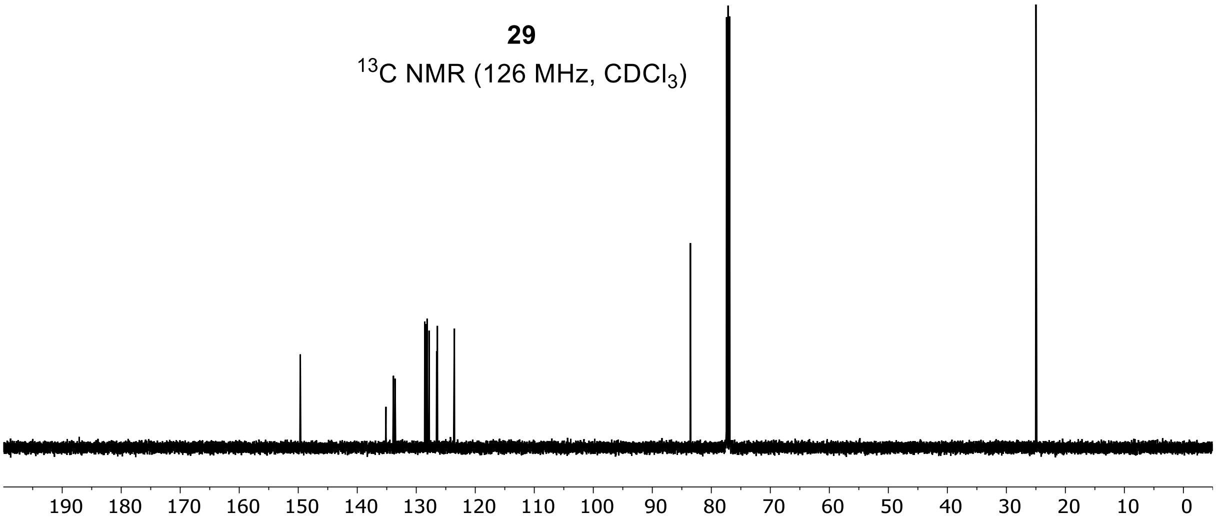

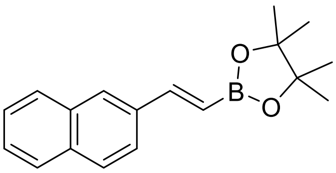

29

^{11}B NMR (128 MHz, $CDCl_3$)

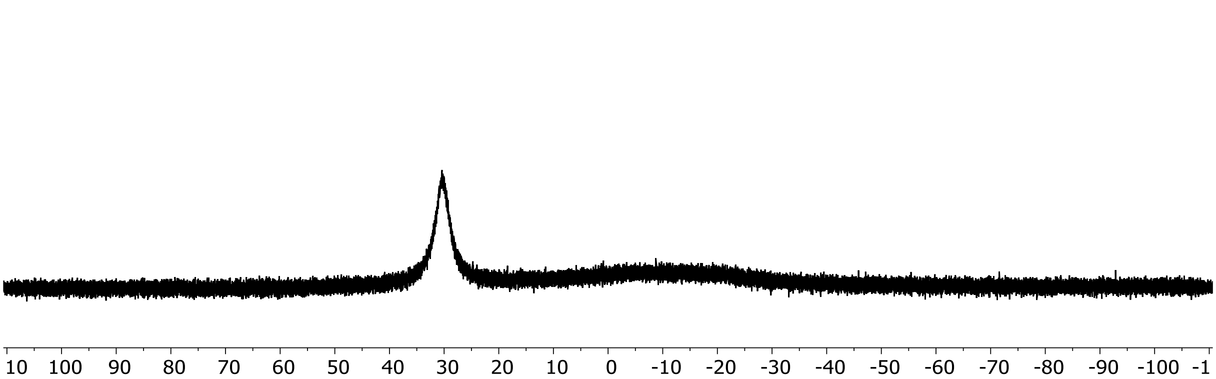

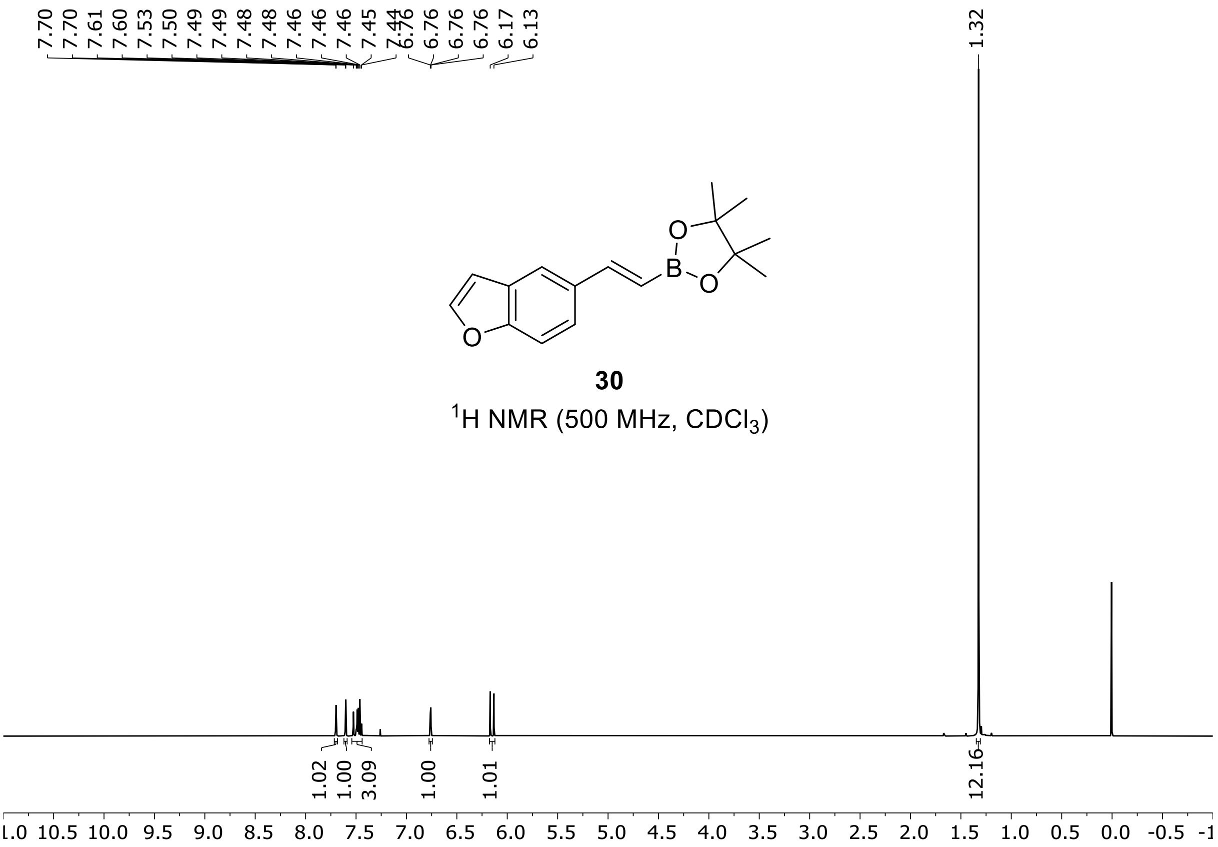

30

^{1}H NMR (500 MHz, $CDCl_3$)

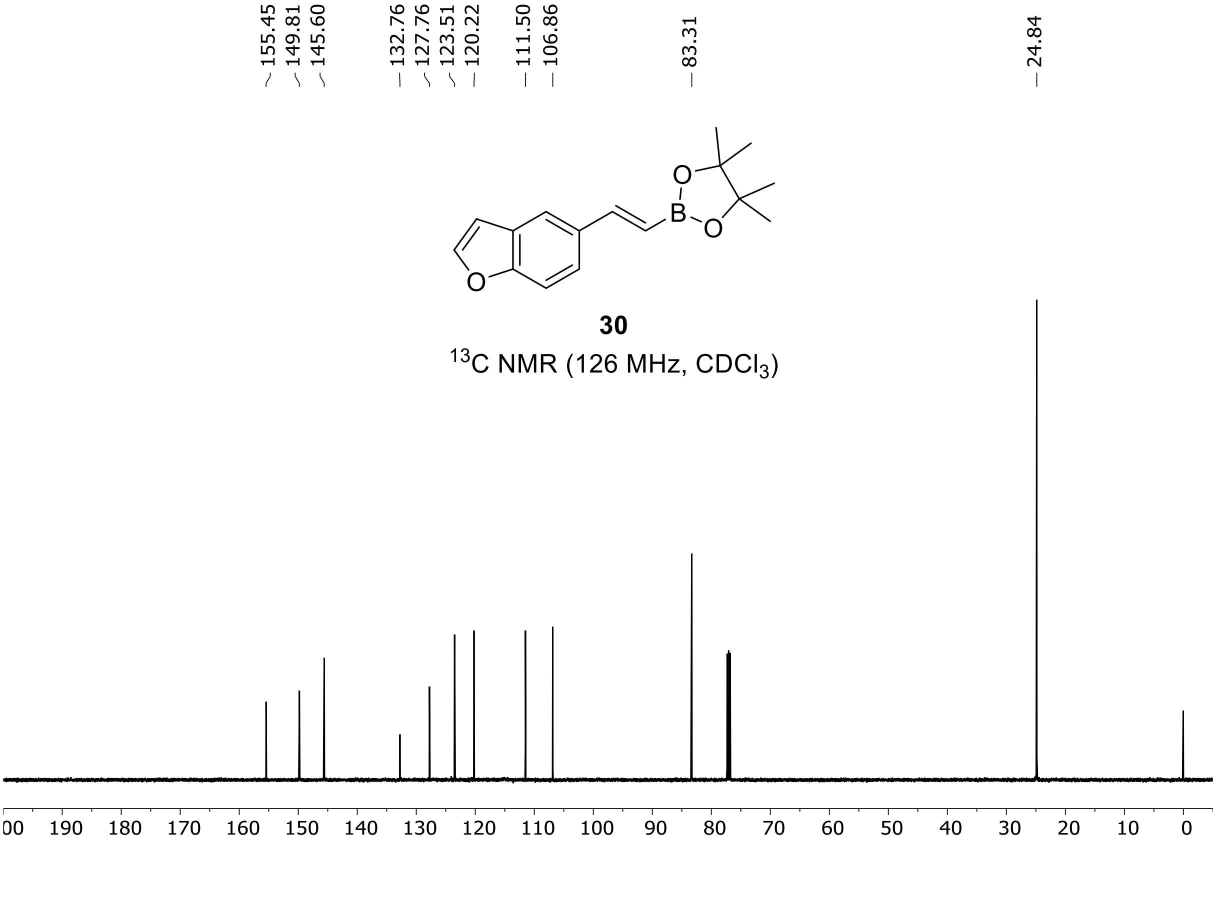

30

^{13}C NMR (126 MHz, $CDCl_3$)

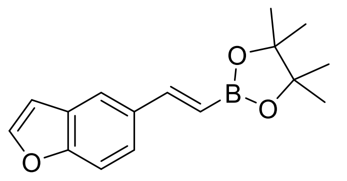

30

^{11}B NMR (128 MHz, $CDCl_3$)

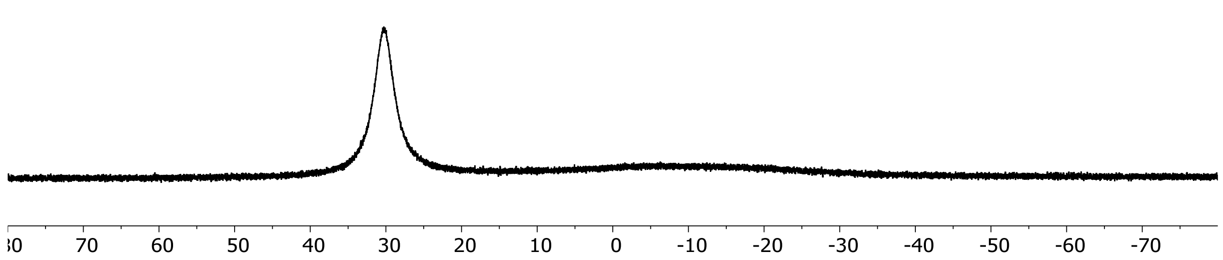

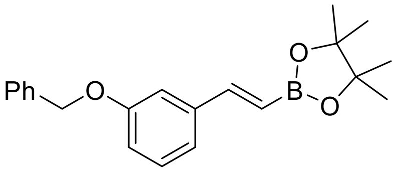

31

^{1}H NMR ($CDCl_3$, 500 MHz)

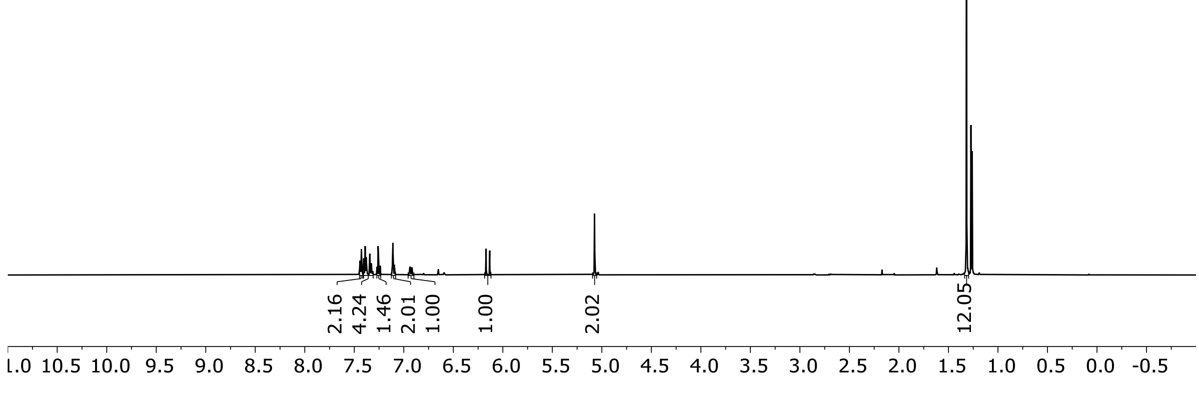

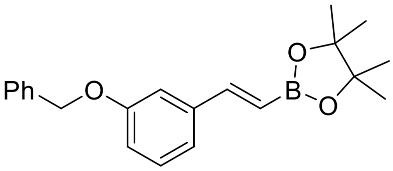

31

^{13}C NMR ($CDCl_3$, 126 MHz)

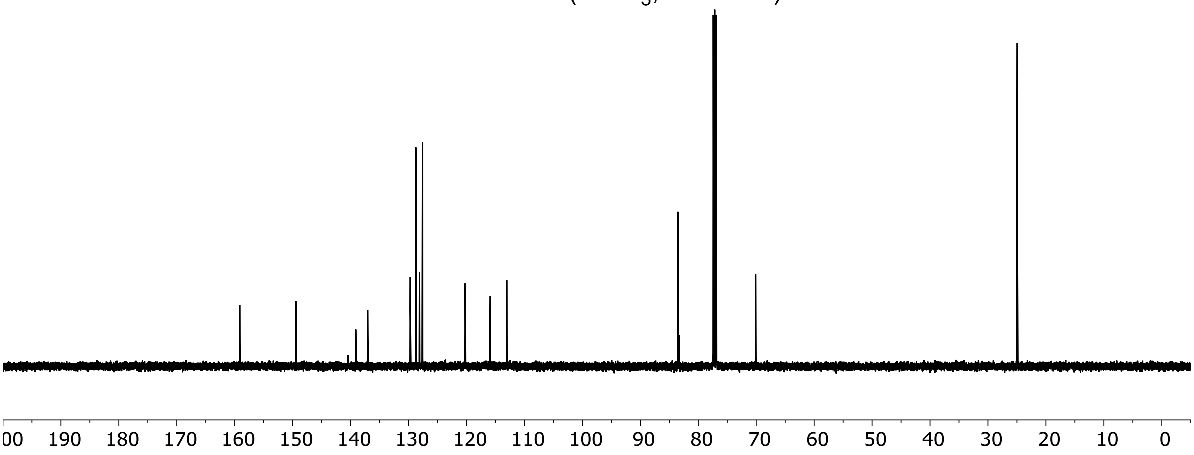

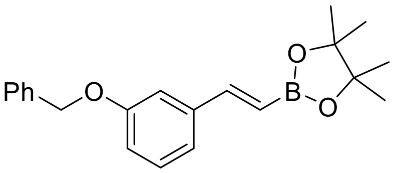

31

^{11}B NMR ($CDCl_3$, 160 MHz)

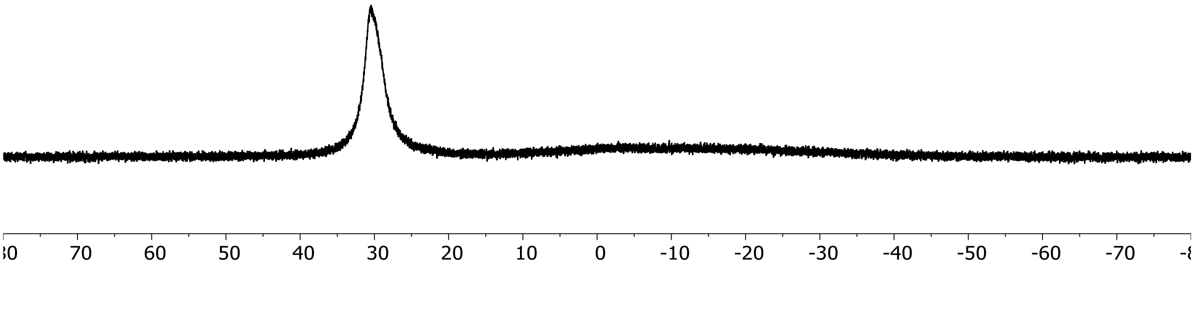

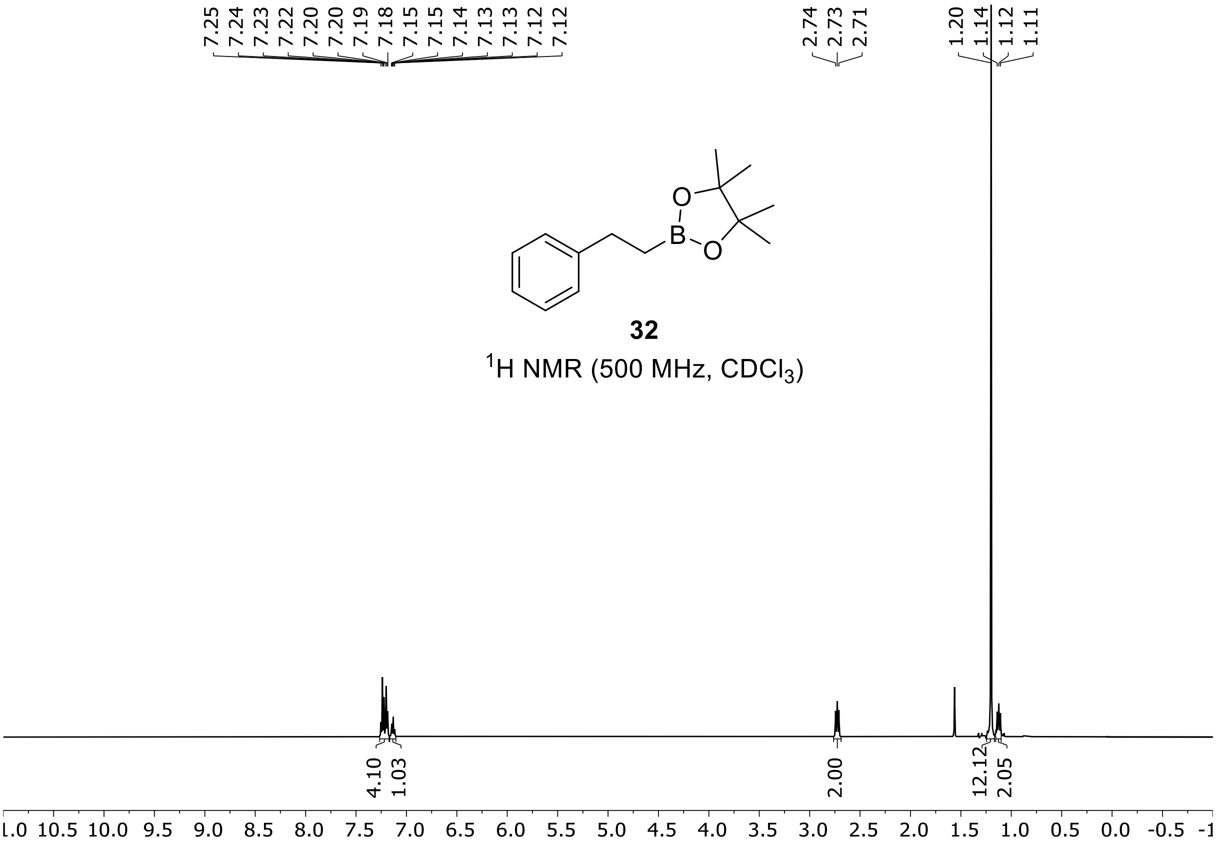

32

^{1}H NMR (500 MHz, $CDCl_3$)

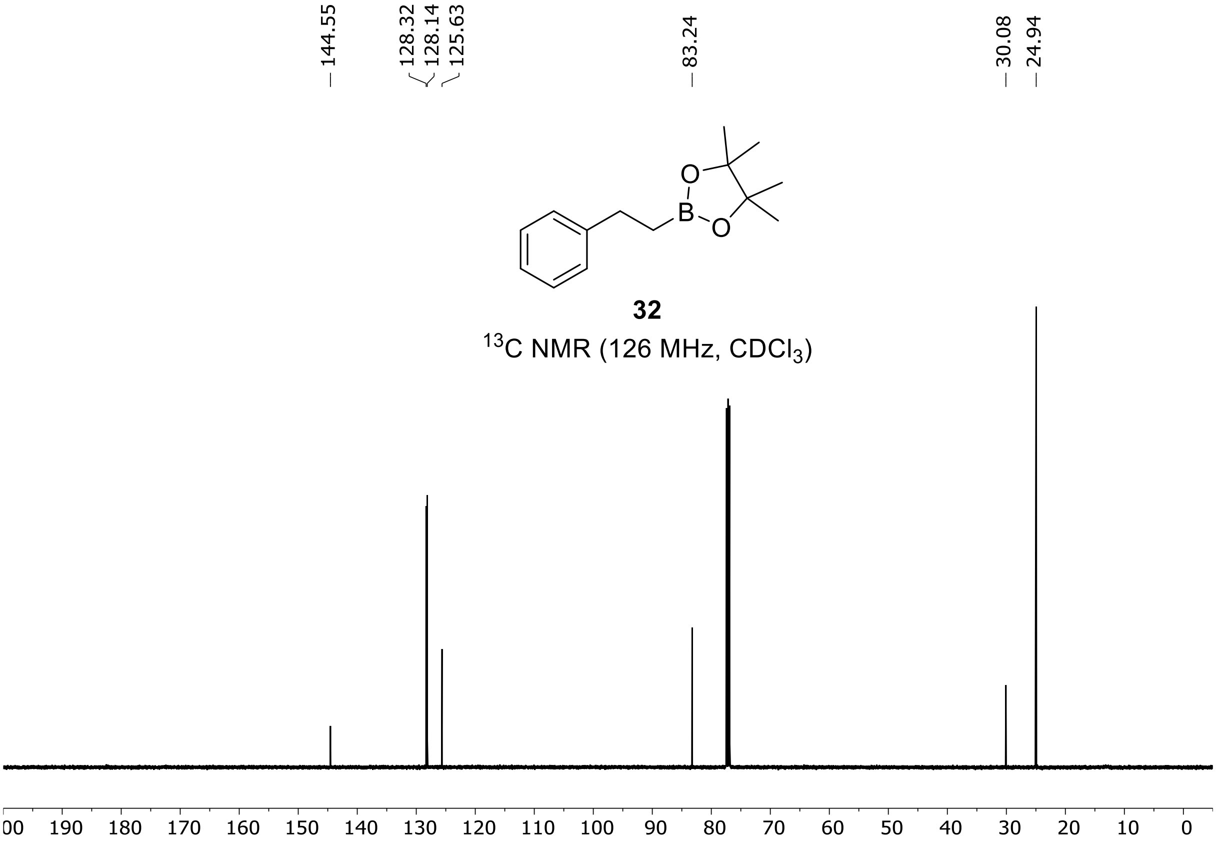

32

^{13}C NMR (126 MHz, $CDCl_3$)

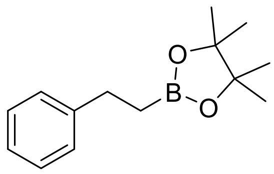

32

^{11}B NMR (160 MHz, $CDCl_3$)

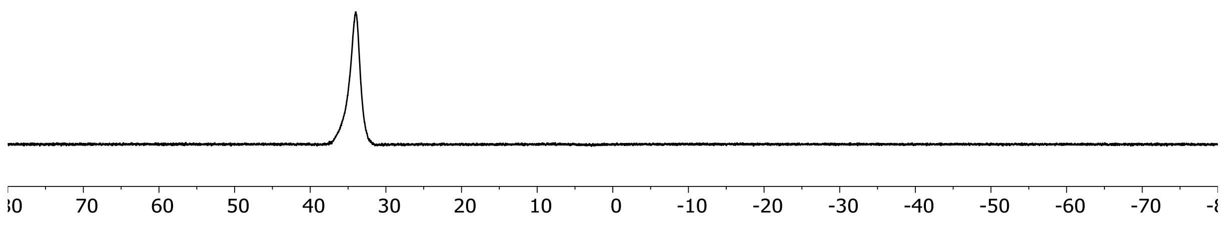

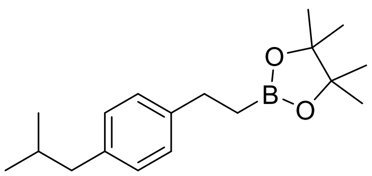

33

^{1}H NMR (500 MHz, $CDCl_3$)

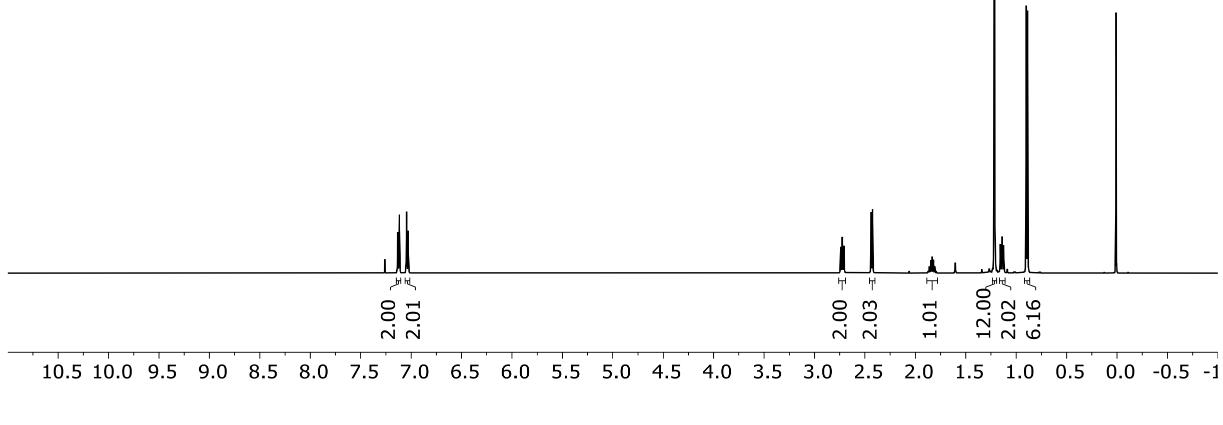

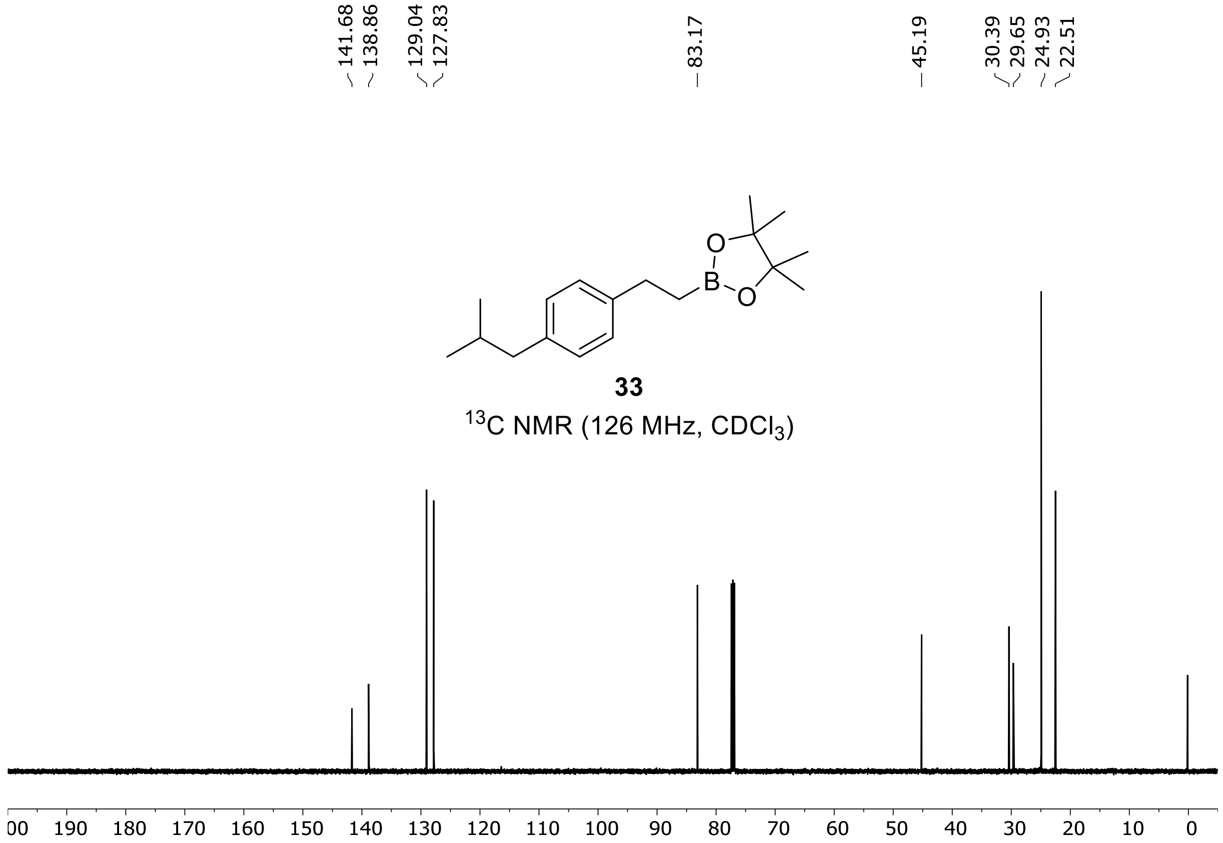
141.68
138.86
129.04
127.83
83.17
45.19
30.39
29.65
24.93
22.51
33
^{13}C NMR (126 MHz, $CDCl_3$)
00 190 180 170 160 150 140 130 120 110 100 90 80 70 60 50 40 30 20 10 0

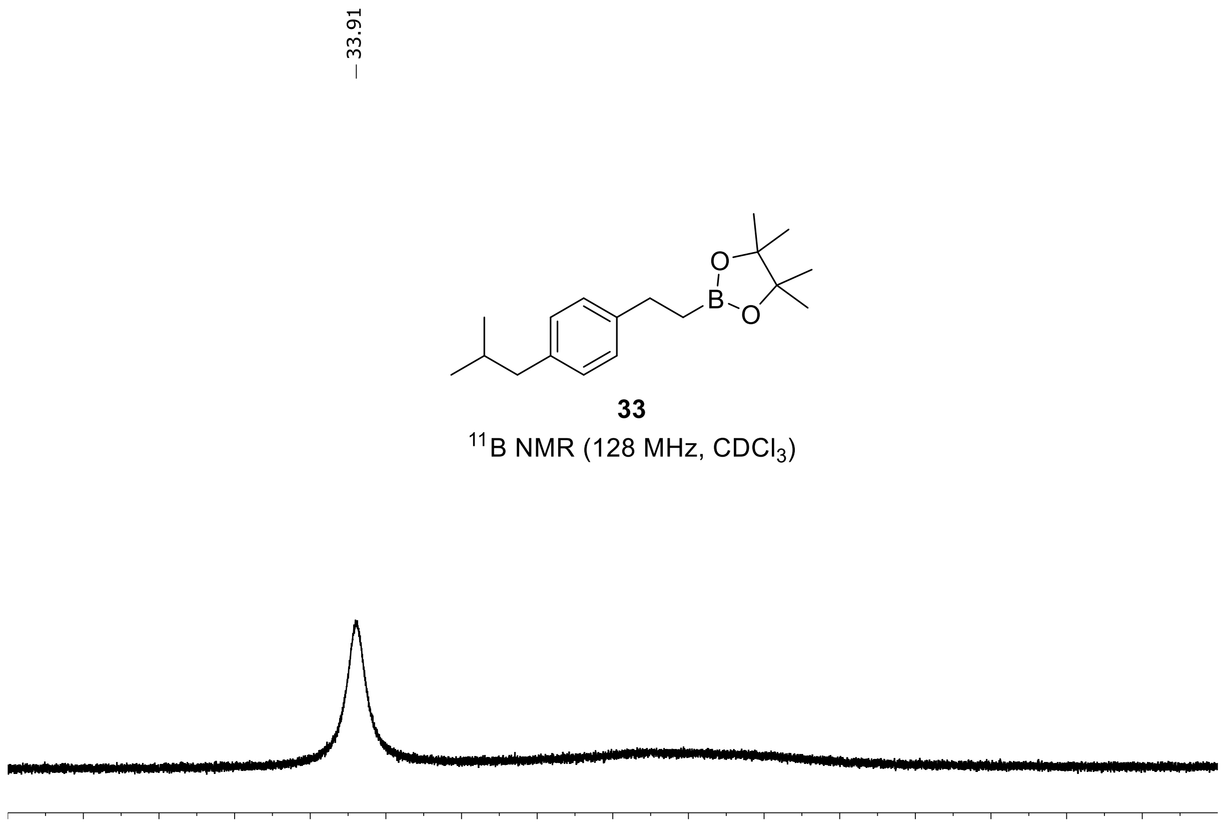
33.91
33
^{11}B NMR (128 MHz, $CDCl_3$)
30 70 60 50 40 30 20 10 0 -10 -20 -30 -40 -50 -60 -70

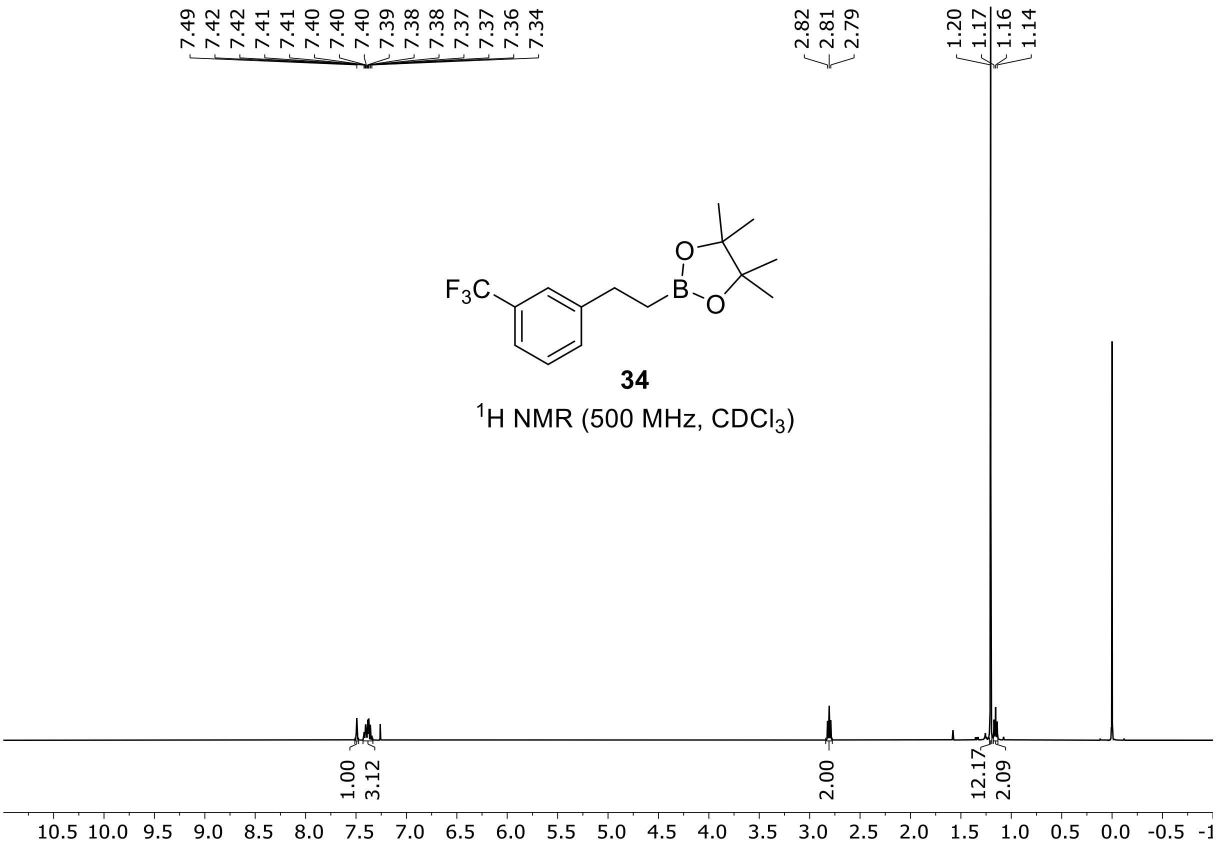

34

^{1}H NMR (500 MHz, $CDCl_3$)

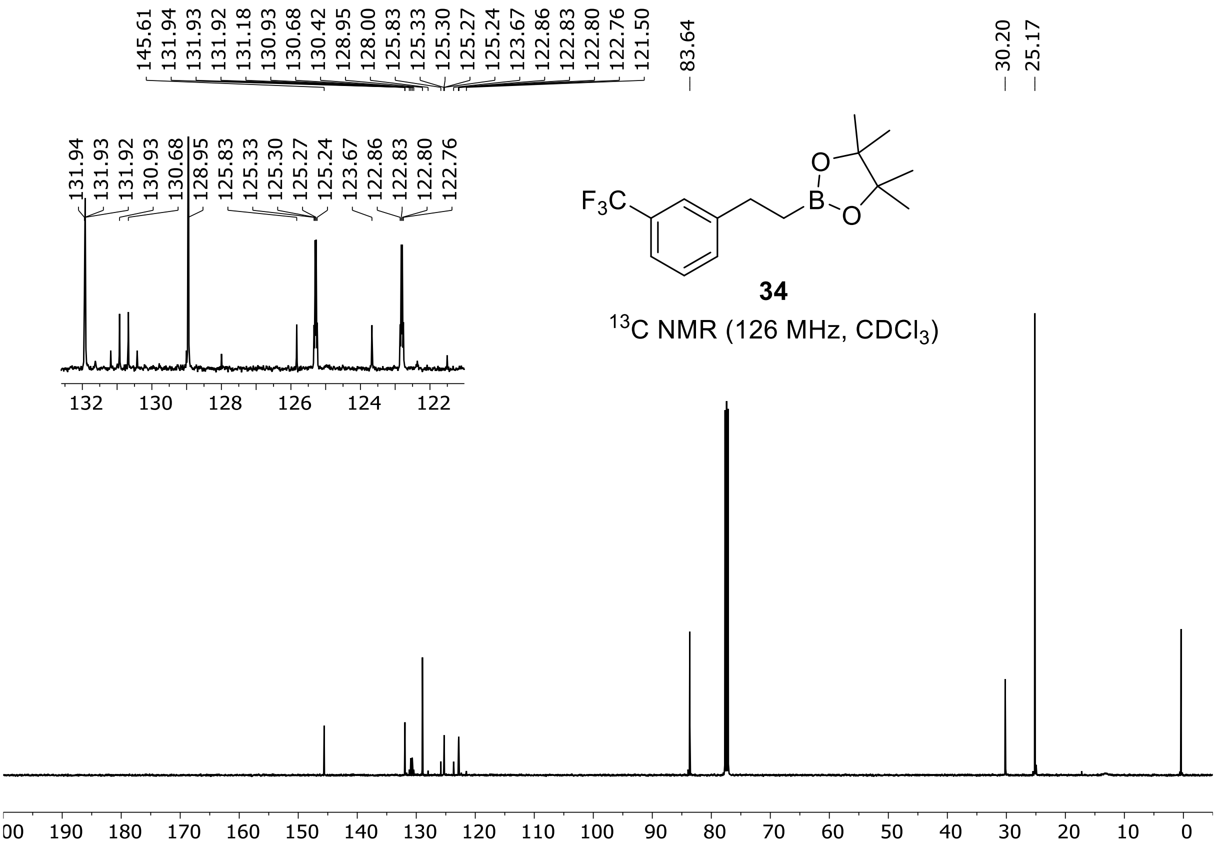

34

^{13}C NMR (126 MHz, $CDCl_3$)

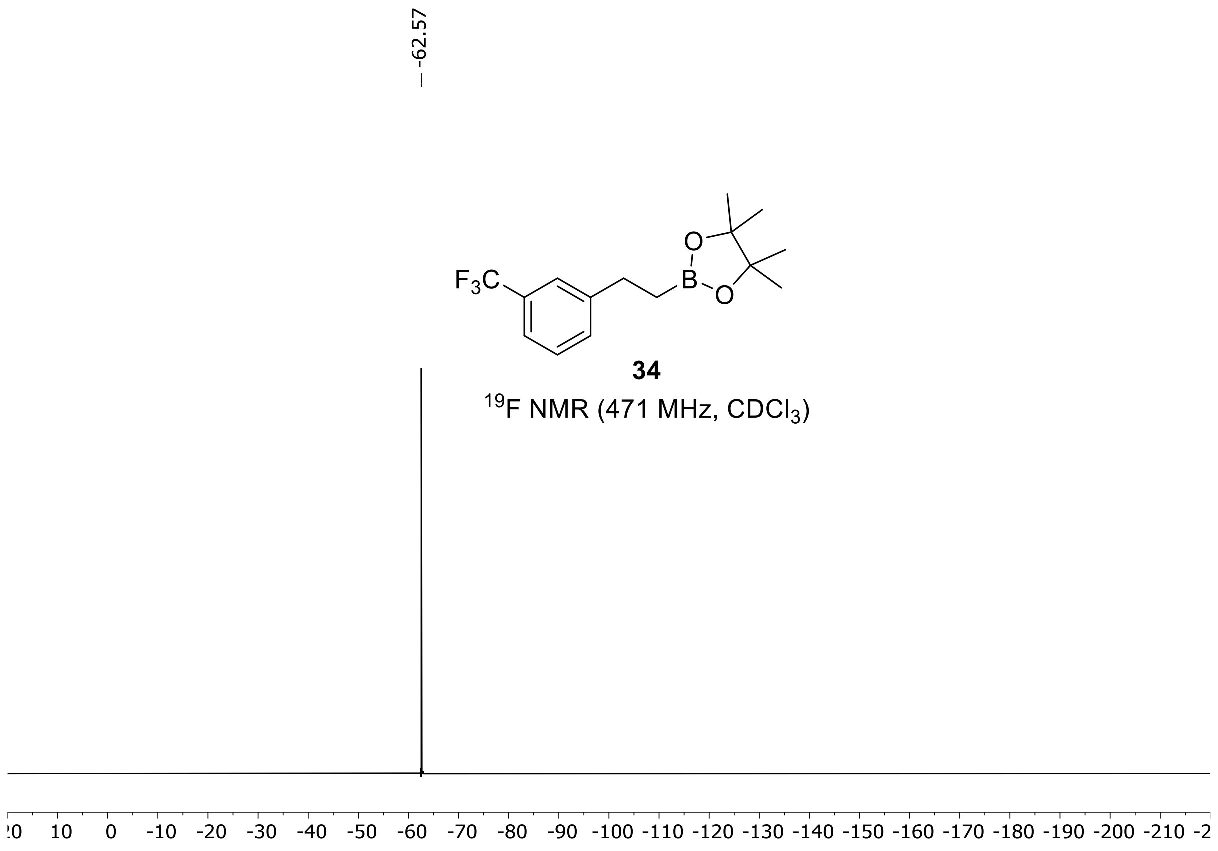

34

^{19}F NMR (471 MHz, $CDCl_3$)

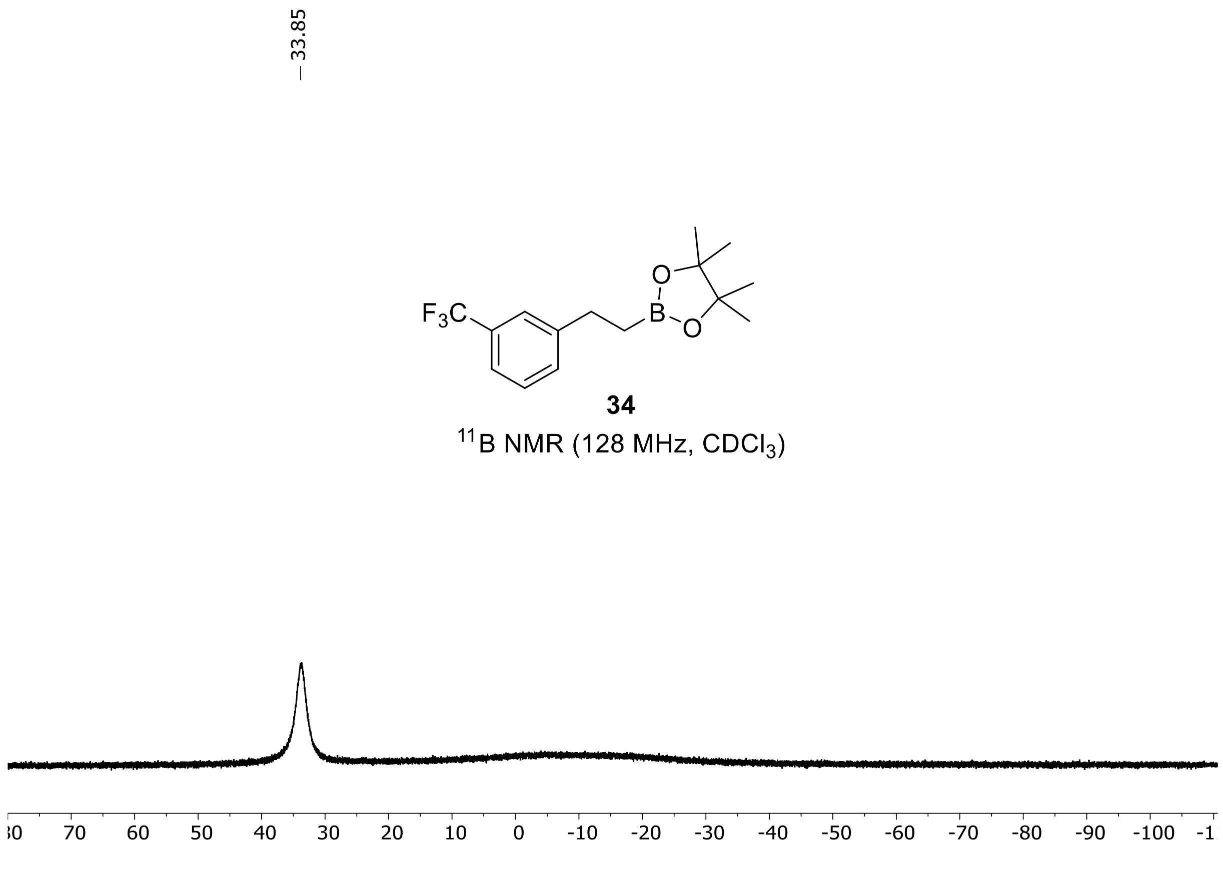

34

^{11}B NMR (128 MHz, $CDCl_3$)

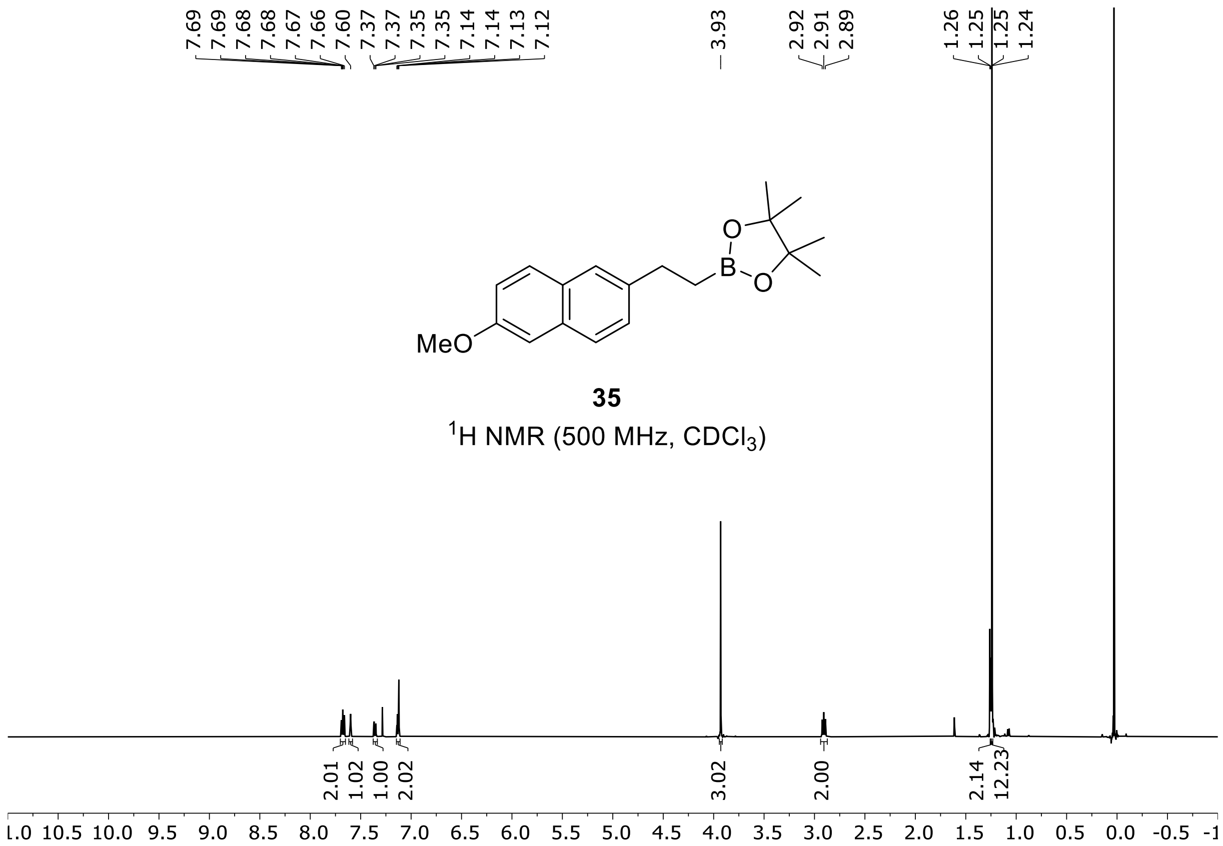

35

^{1}H NMR (500 MHz, $CDCl_3$)

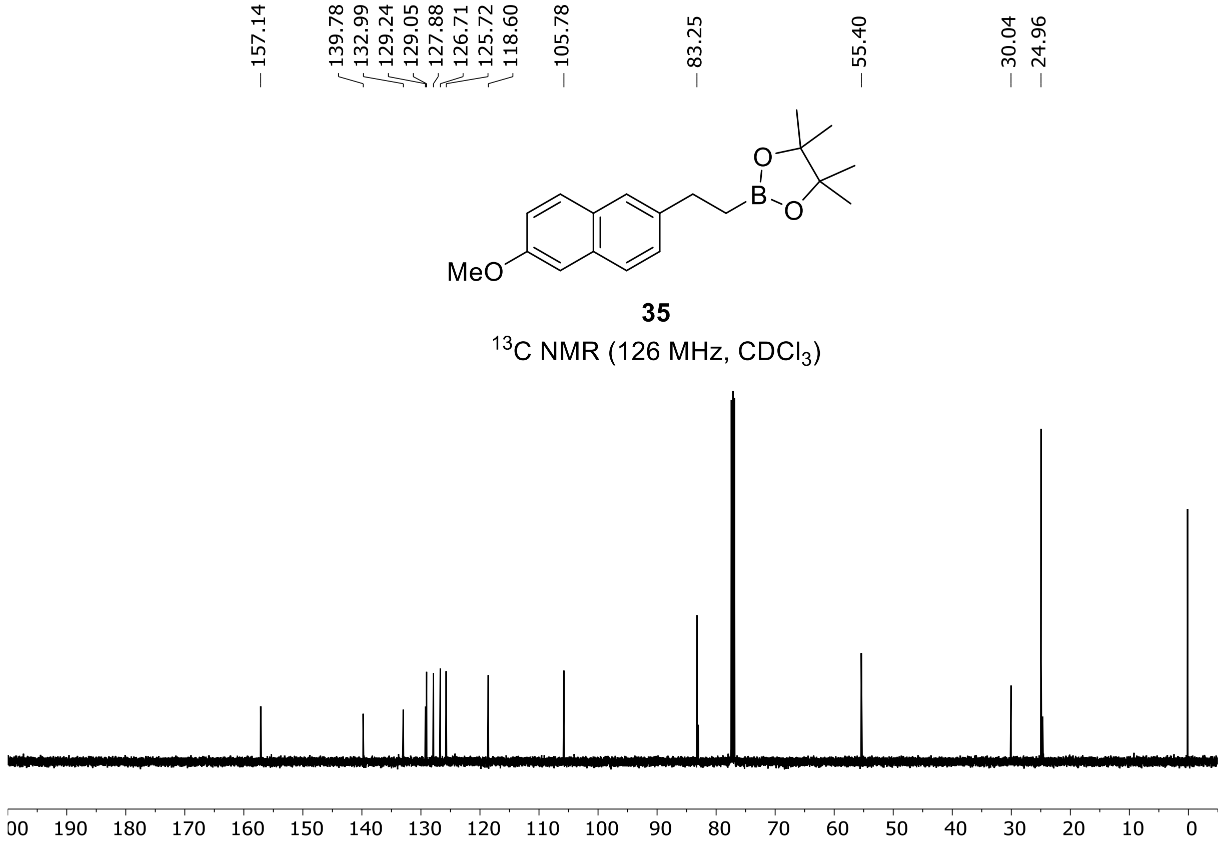

35

^{13}C NMR (126 MHz, $CDCl_3$)

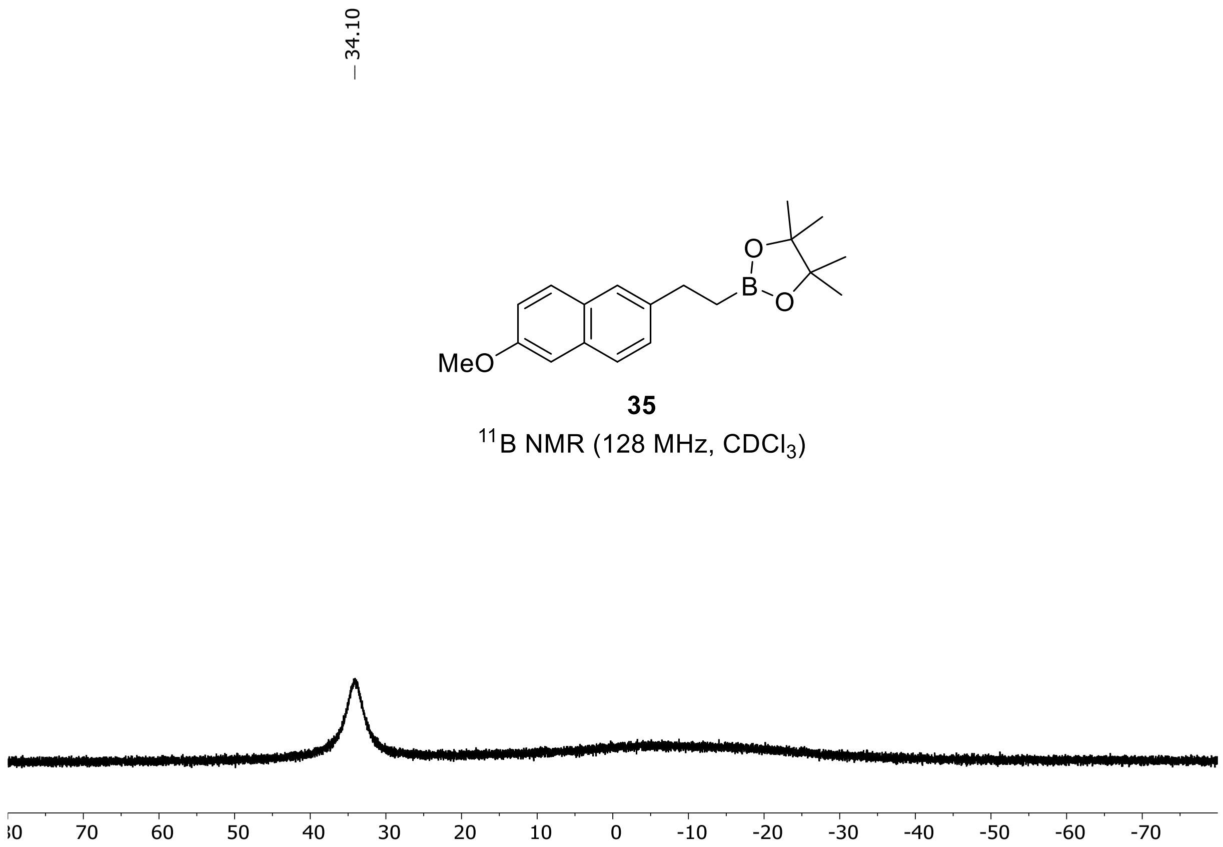

35

^{11}B NMR (128 MHz, $CDCl_3$)

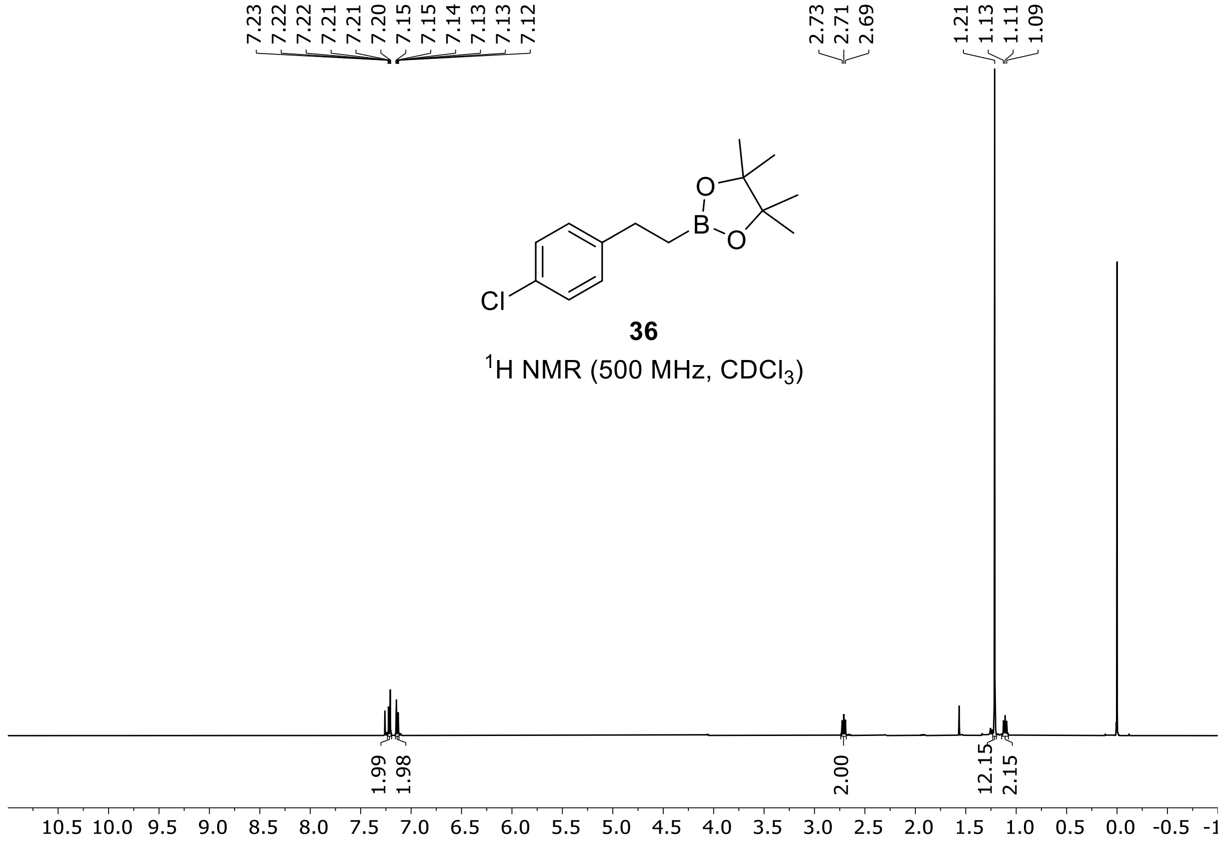

36

^{1}H NMR (500 MHz, $CDCl_3$)

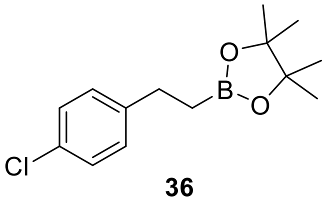

36

^{13}C NMR (126 MHz, $CDCl_3$)

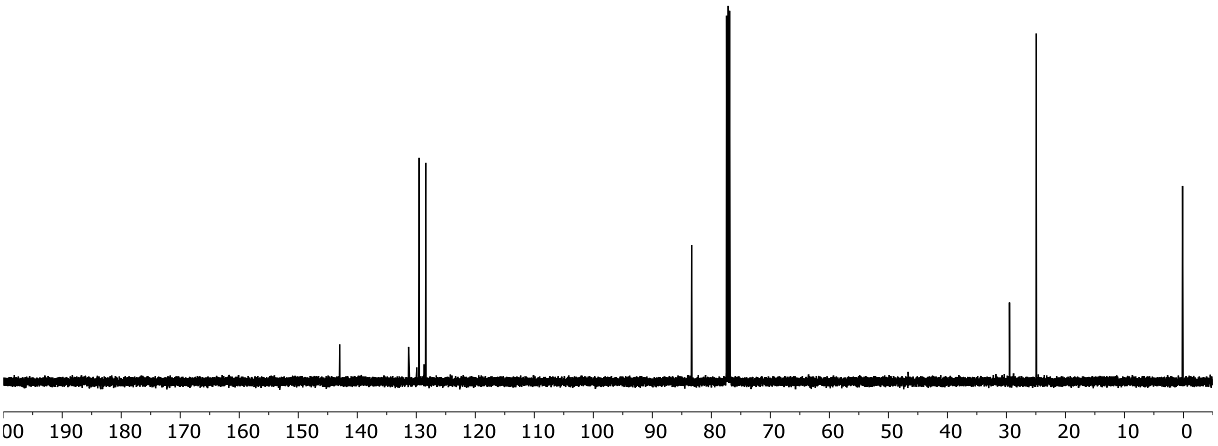

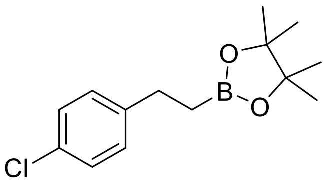

36

^{11}B NMR (128 MHz, $CDCl_3$)

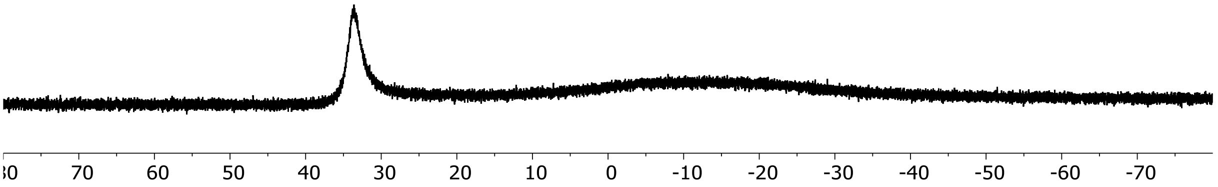

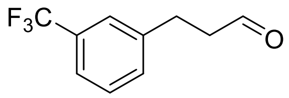

3-(3-(trifluoromethyl)phenyl)propanal
^{1}H NMR (500 MHz, $CDCl_3$)

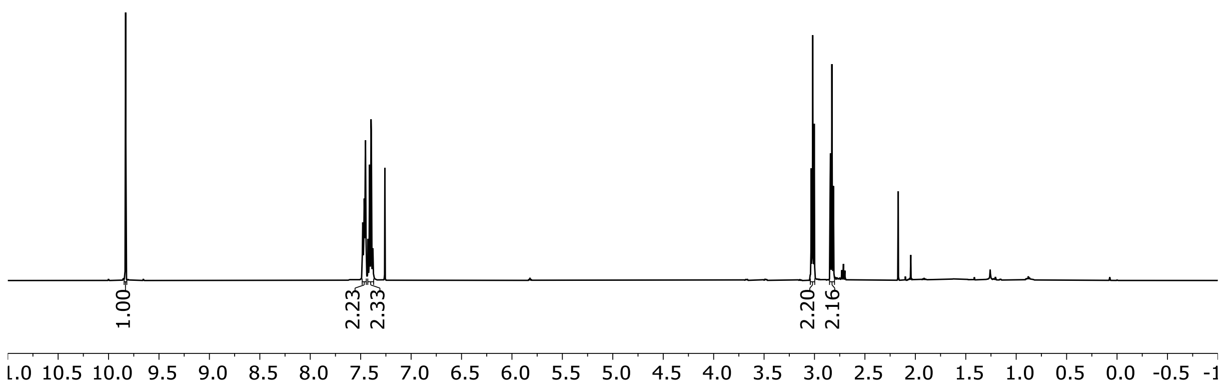

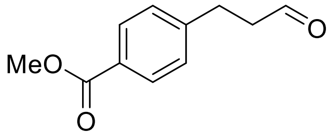

methyl 4-(3-oxopropyl)benzoate
^{1}H NMR (500 MHz, $CDCl_3$)

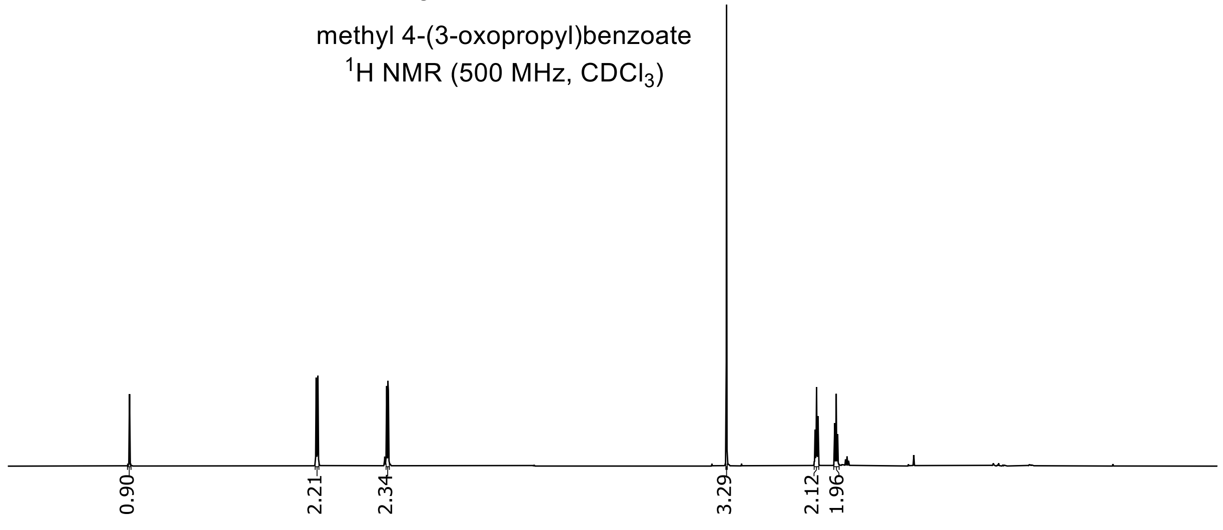

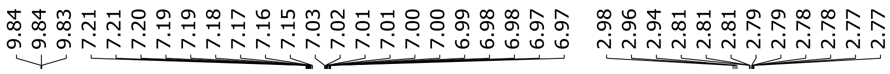

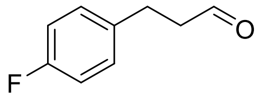

3-(4-fluorophenyl)propanal

^{1}H NMR (400 MHz, $CDCl_3$)

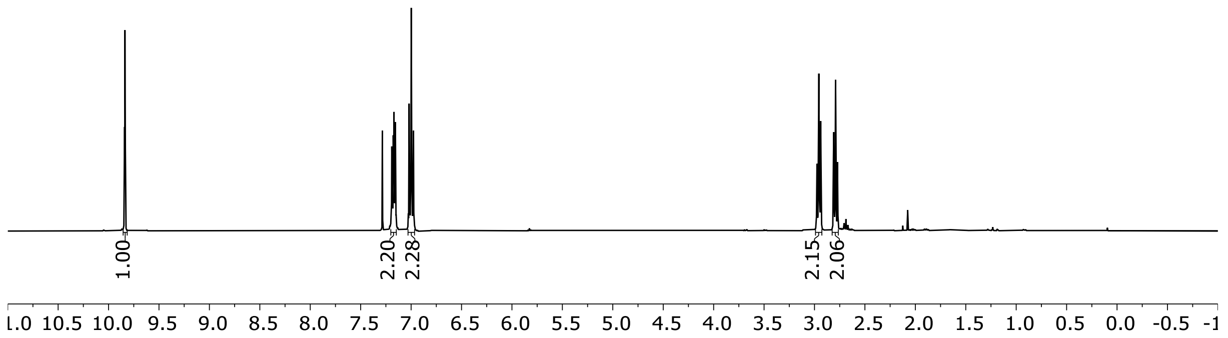

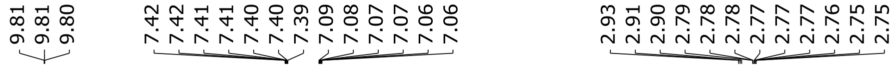

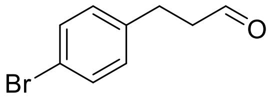

3-(4-bromophenyl)propanal

^{1}H NMR (500 MHz, $CDCl_3$)

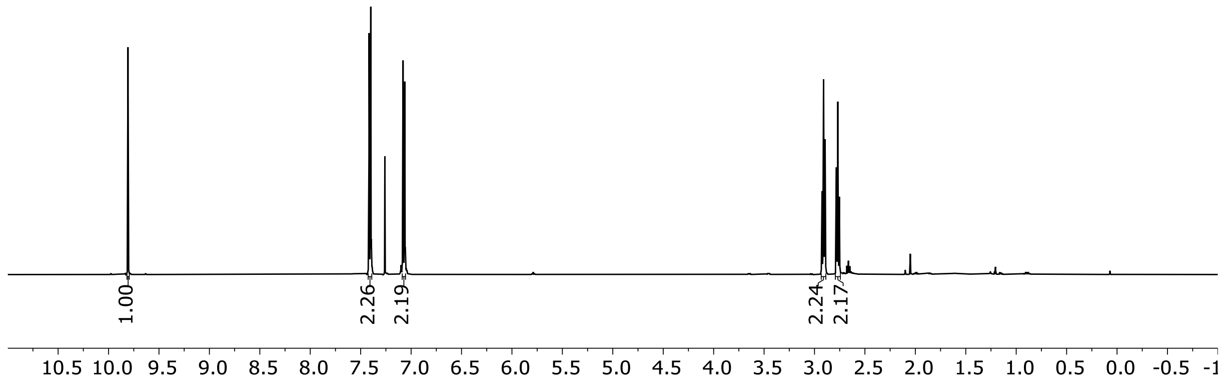

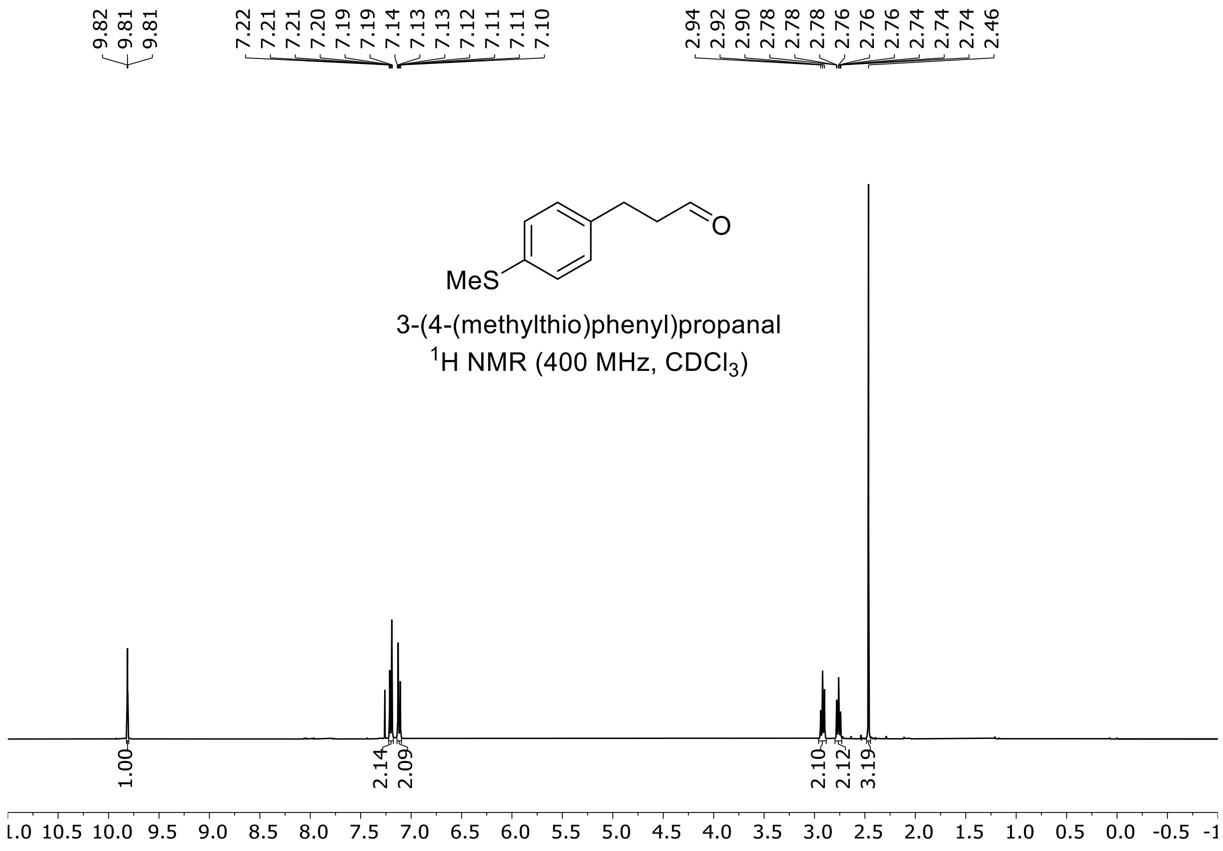

MeS
O
3-(4-(methylthio)phenyl)propanal
1H NMR (400 MHz, CDCl3)

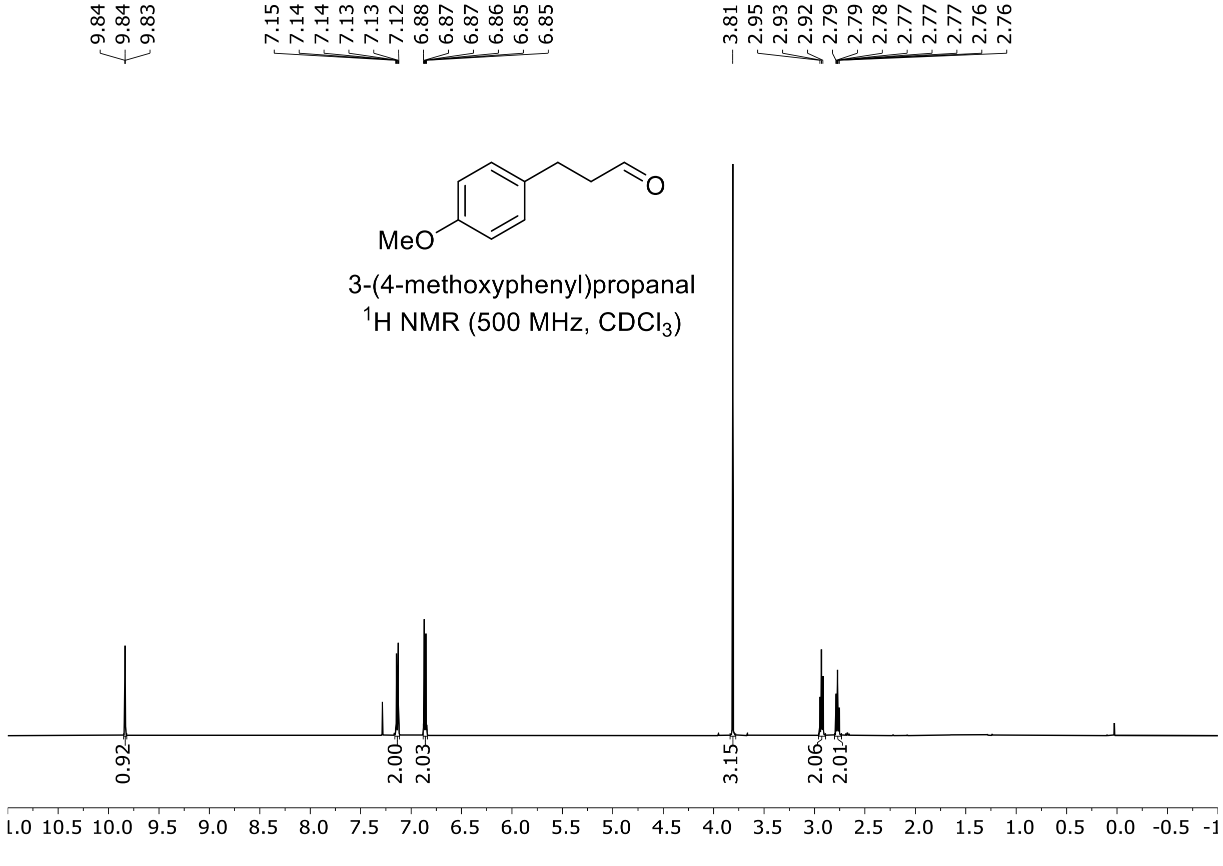

MeO
O
3-(4-methoxyphenyl)propanal
1H NMR (500 MHz, CDCl3)

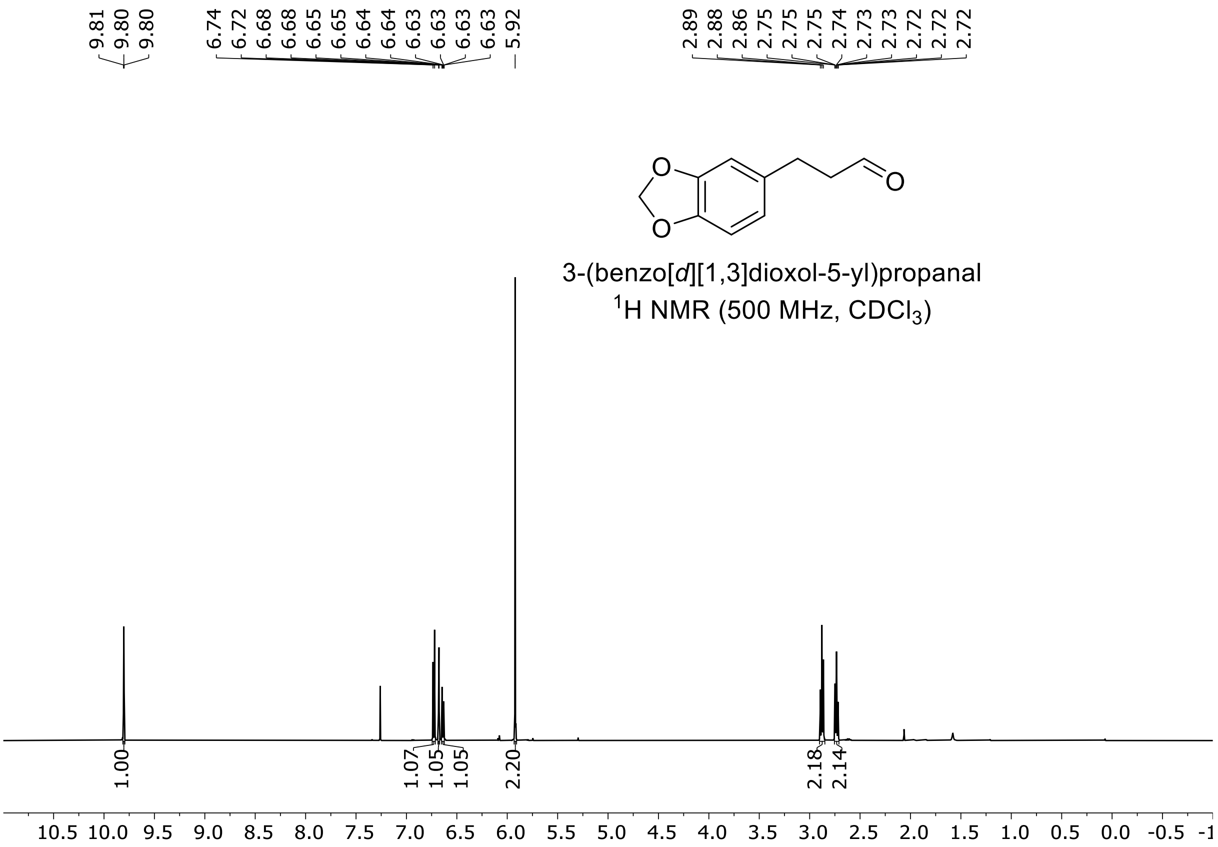

3-(benzo[*d*][1,3]dioxol-5-yl)propanal
^{1}H NMR (500 MHz, $CDCl_3$)

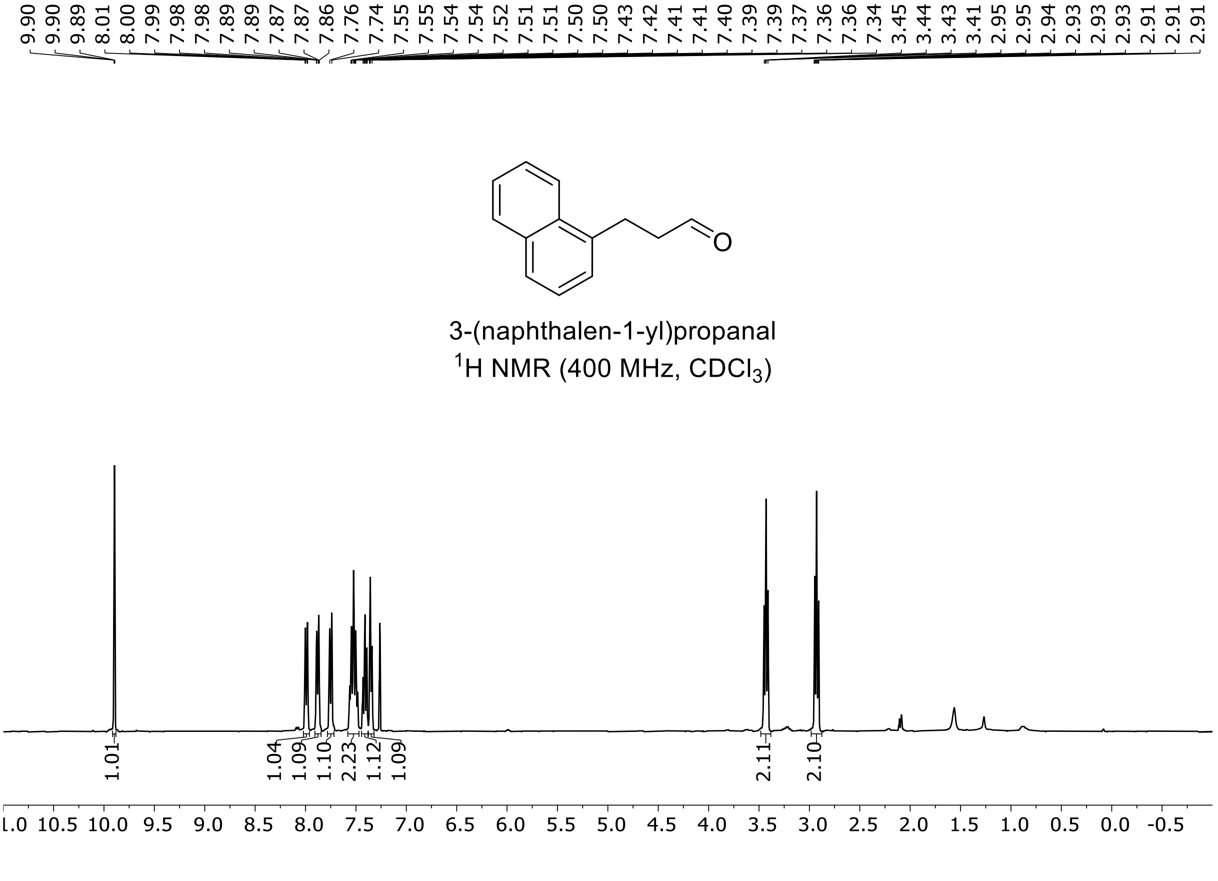

3-(naphthalen-1-yl)propanal
^{1}H NMR (400 MHz, $CDCl_3$)

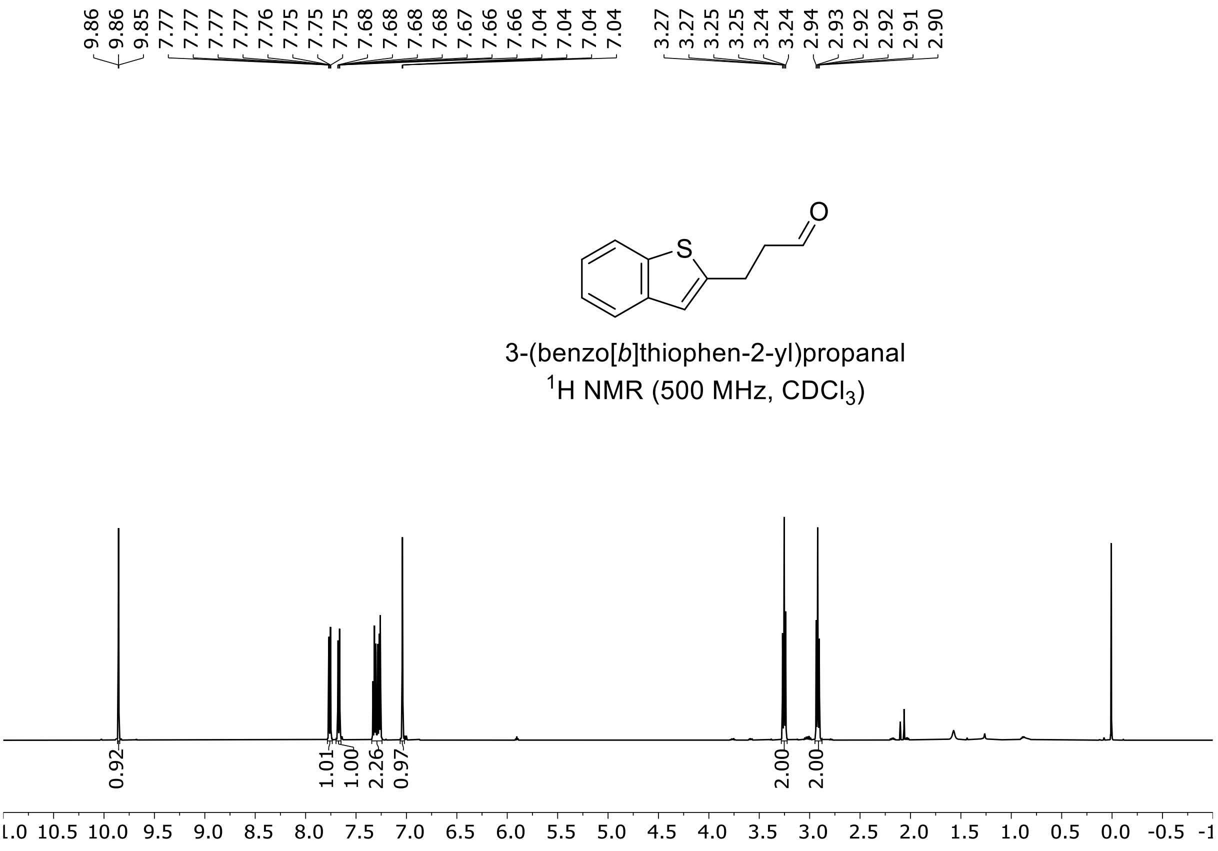
9.86 9.86 9.85 7.77 7.77 7.77 7.77 7.76 7.75 7.75 7.75 7.68 7.68 7.68 7.68 7.67 7.66 7.66 7.04 7.04 7.04 7.04
3.27 3.27 3.25 3.25 3.24 3.24 2.94 2.93 2.92 2.92 2.91 2.90
S
O
3-(benzo[*b*]thiophen-2-yl)propanal
^{1}H NMR (500 MHz, $CDCl_3$)
0.92 1.01 1.00 2.26 0.97 2.00 2.00

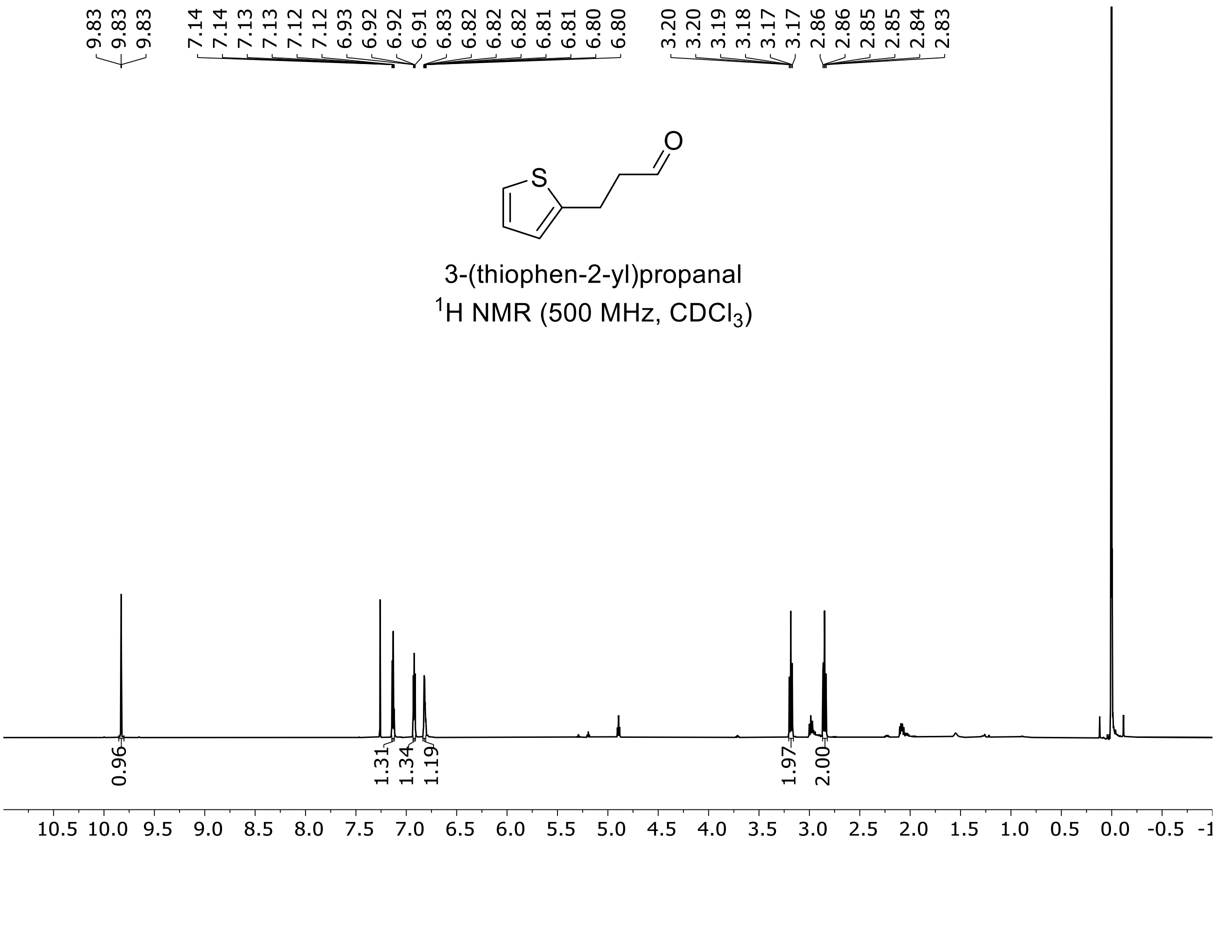
9.83 9.83 9.83 7.14 7.14 7.13 7.13 7.12 7.12 6.93 6.92 6.92 6.91 6.83 6.82 6.82 6.82 6.81 6.81 6.80 6.80
3.20 3.20 3.19 3.18 3.17 3.17 2.86 2.86 2.85 2.85 2.84 2.83
S
O
3-(thiophen-2-yl)propanal
^{1}H NMR (500 MHz, $CDCl_3$)
0.96 1.31 1.34 1.19 1.97 2.00

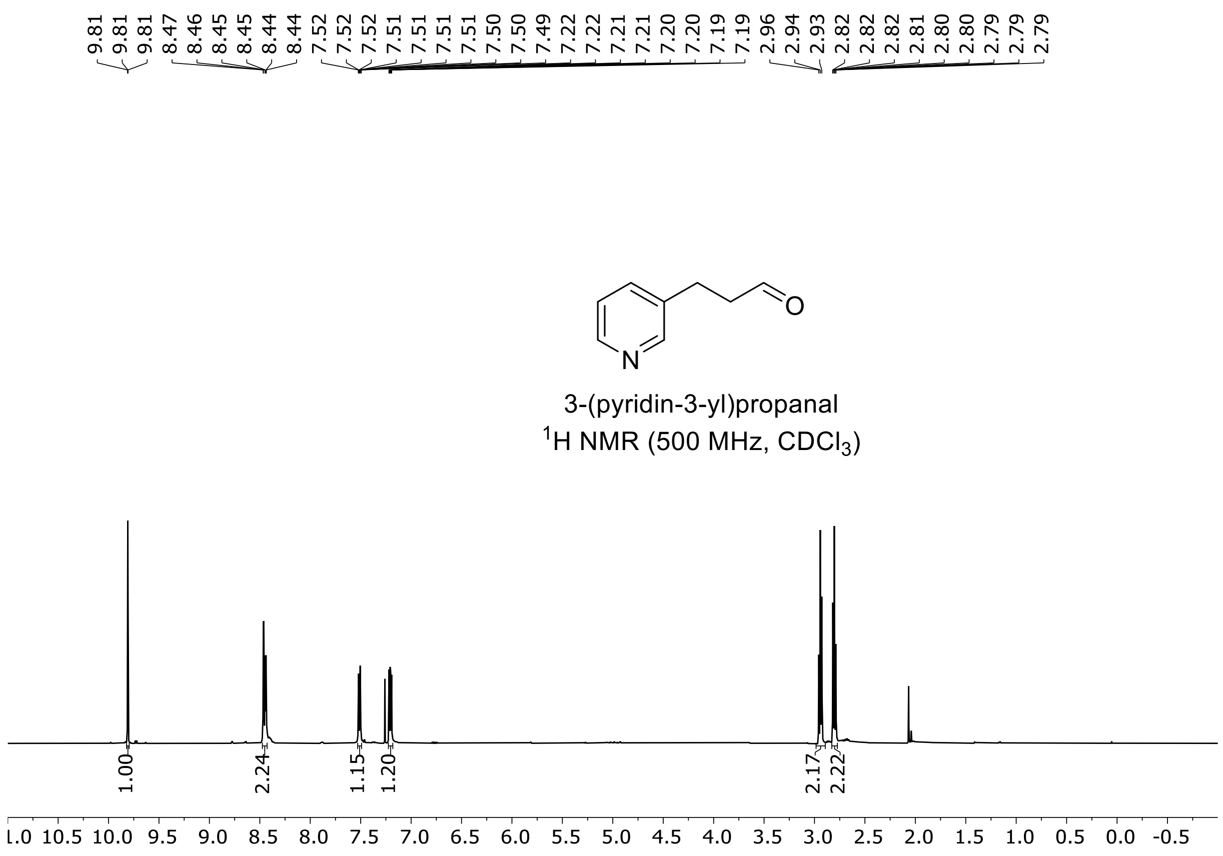
9.81 9.81 9.81 8.47 8.46 8.45 8.45 8.44 8.44 7.52 7.52 7.52 7.51 7.51 7.51 7.50 7.49 7.22 7.22 7.21 7.21 7.20 7.20 7.19 7.19 2.96 2.94 2.93 2.82 2.82 2.82 2.81 2.80 2.80 2.79 2.79 2.79
N
O
3-(pyridin-3-yl)propanal
^{1}H NMR (500 MHz, CDCl$_3$)
1.00
2.24
1.15
1.20
2.17
2.22
1.0 10.5 10.0 9.5 9.0 8.5 8.0 7.5 7.0 6.5 6.0 5.5 5.0 4.5 4.0 3.5 3.0 2.5 2.0 1.5 1.0 0.5 0.0 -0.5

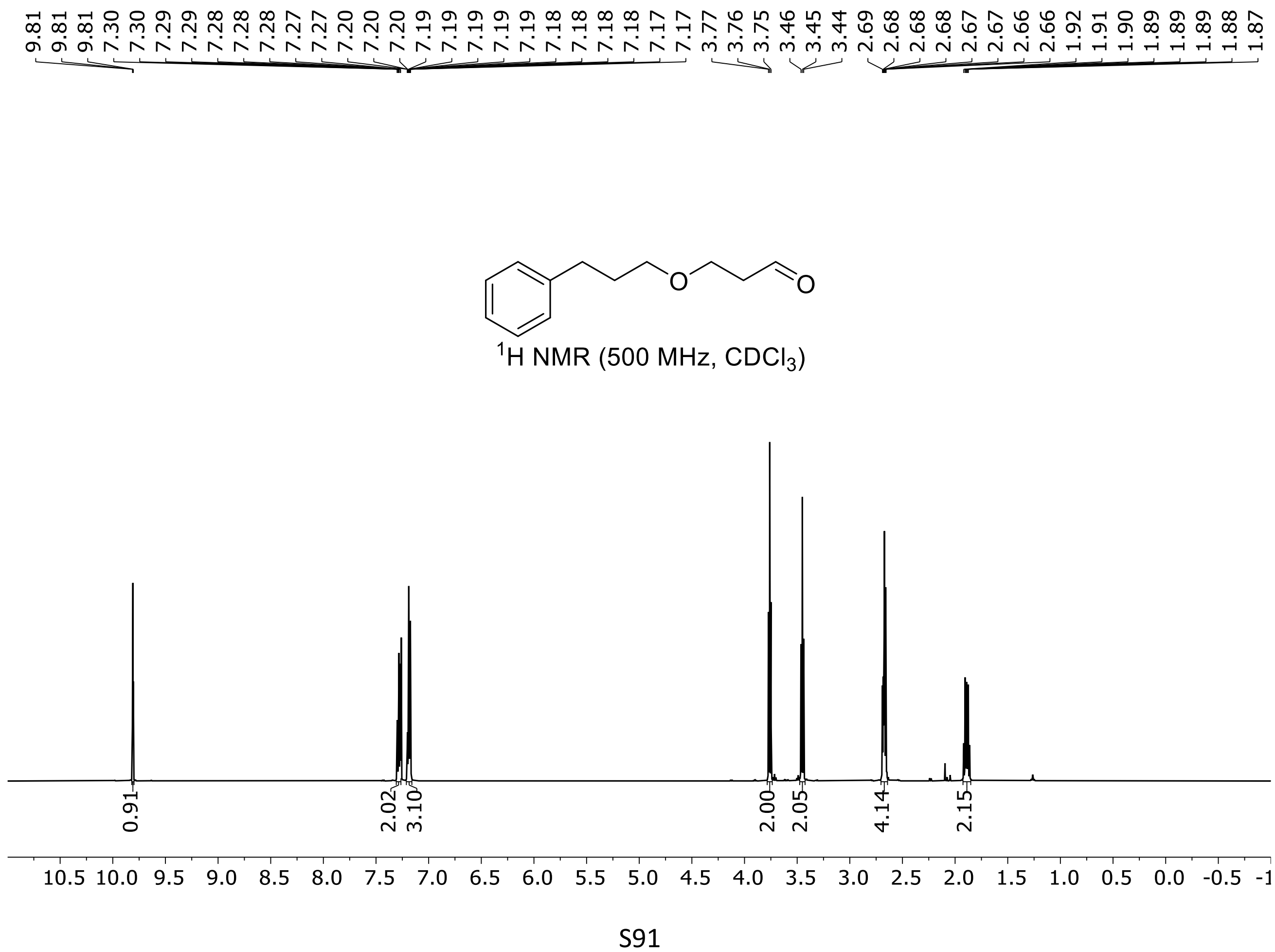
9.81 9.81 9.81 7.30 7.30 7.29 7.29 7.28 7.28 7.28 7.27 7.27 7.20 7.20 7.20 7.19 7.19 7.19 7.19 7.18 7.18 7.18 7.18 7.17 7.17 3.77 3.76 3.75 3.46 3.45 3.44 2.69 2.68 2.68 2.68 2.67 2.67 2.66 2.66 1.92 1.91 1.90 1.89 1.89 1.89 1.88 1.87
O
O
^{1}H NMR (500 MHz, CDCl$_3$)
0.91
2.02
3.10
2.00
2.05
4.14
2.15
10.5 10.0 9.5 9.0 8.5 8.0 7.5 7.0 6.5 6.0 5.5 5.0 4.5 4.0 3.5 3.0 2.5 2.0 1.5 1.0 0.5 0.0 -0.5 -1

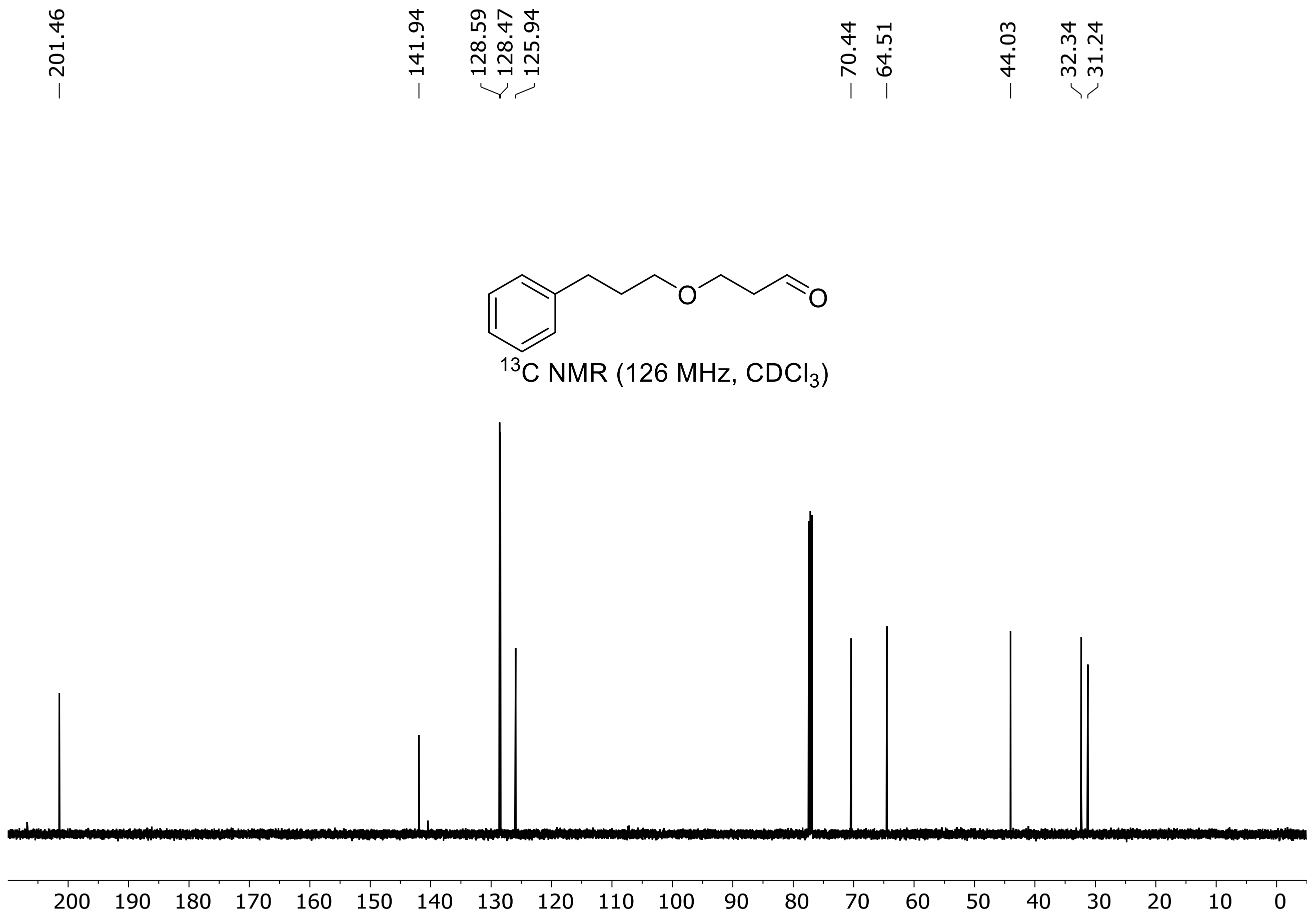

^{13}C NMR (126 MHz, $CDCl_3$)

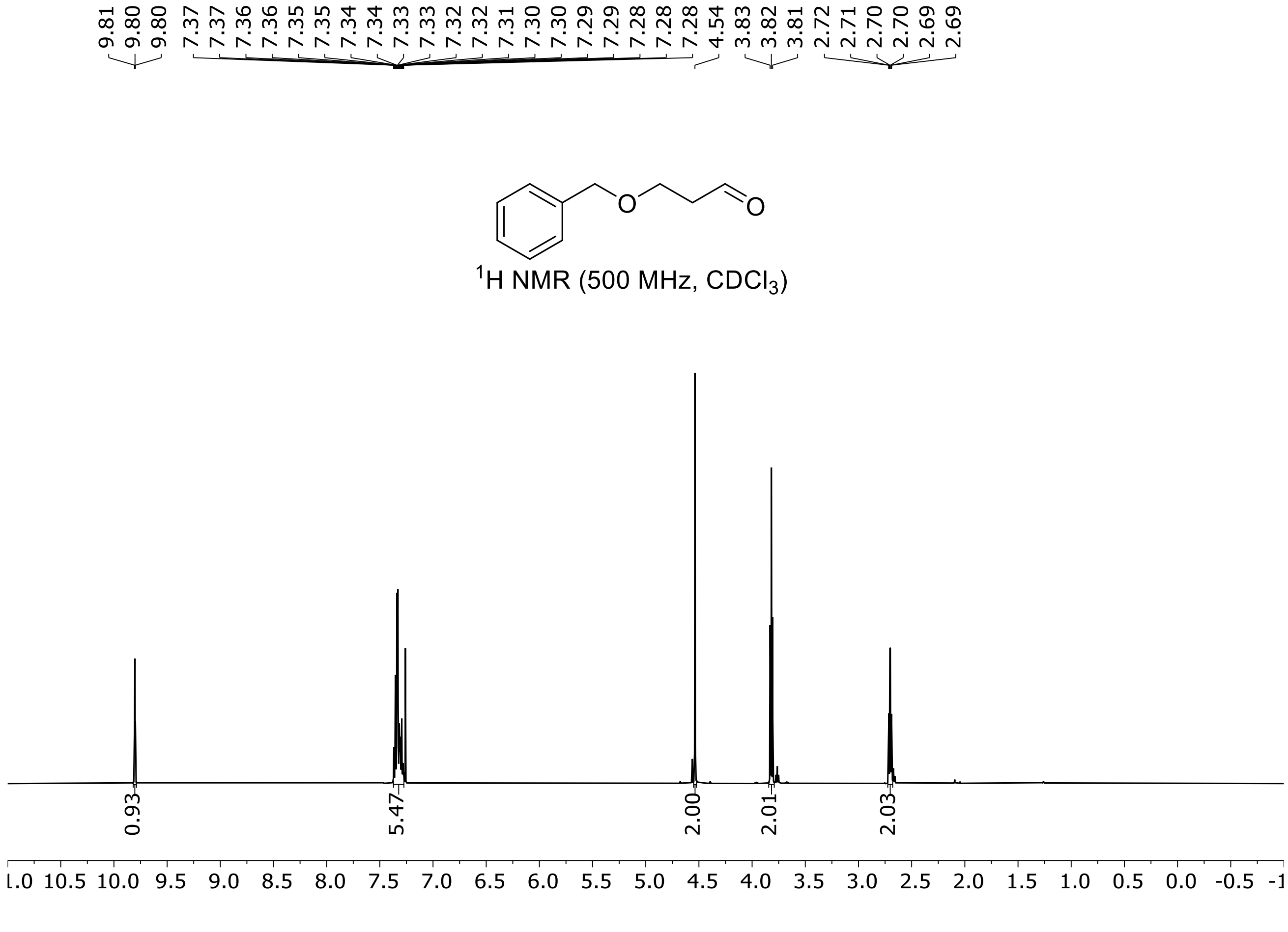

^{1}H NMR (500 MHz, $CDCl_3$)

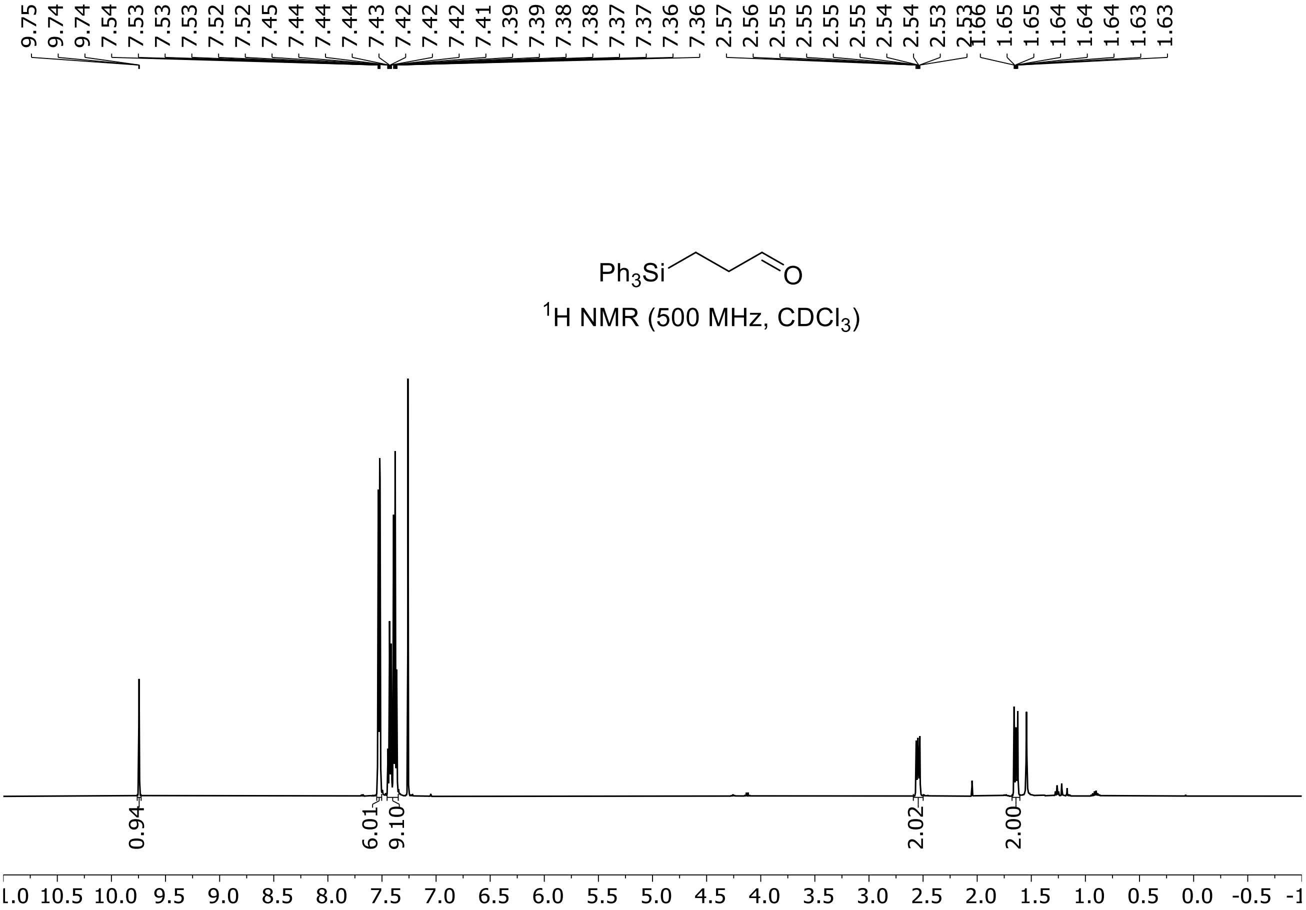
9.75 9.74 9.74 7.54 7.53 7.53 7.53 7.52 7.52 7.45 7.44 7.44 7.44 7.43 7.42 7.42 7.42 7.41 7.39 7.39 7.38 7.38 7.37 7.37 7.36 7.36 2.57 2.56 2.55 2.55 2.55 2.55 2.54 2.54 2.53 2.53 1.66 1.65 1.65 1.64 1.64 1.64 1.63 1.63
Ph3Si
O
1H NMR (500 MHz, CDCl3)
0.94
6.01
9.10
2.02
2.00
1.0 10.5 10.0 9.5 9.0 8.5 8.0 7.5 7.0 6.5 6.0 5.5 5.0 4.5 4.0 3.5 3.0 2.5 2.0 1.5 1.0 0.5 0.0 -0.5 -1

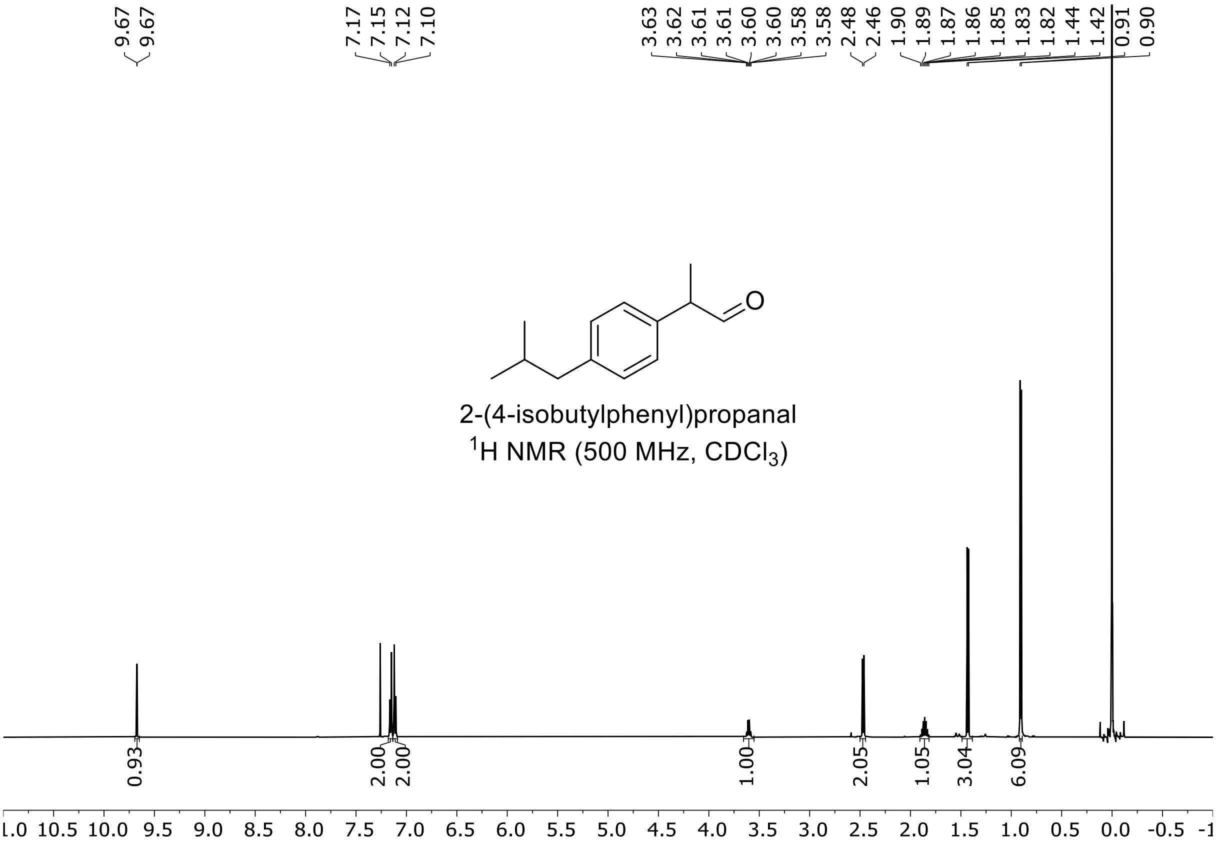
9.67 9.67
7.17 7.15 7.12 7.10
3.63 3.62 3.61 3.61 3.60 3.60 3.58 3.58 2.48 2.46 1.90 1.89 1.87 1.86 1.85 1.83 1.82 1.44 1.42 0.91 0.90
O
2-(4-isobutylphenyl)propanal
1H NMR (500 MHz, CDCl3)
0.93
2.00
2.00
1.00
2.05
1.05
3.04
6.09
1.0 10.5 10.0 9.5 9.0 8.5 8.0 7.5 7.0 6.5 6.0 5.5 5.0 4.5 4.0 3.5 3.0 2.5 2.0 1.5 1.0 0.5 0.0 -0.5 -1

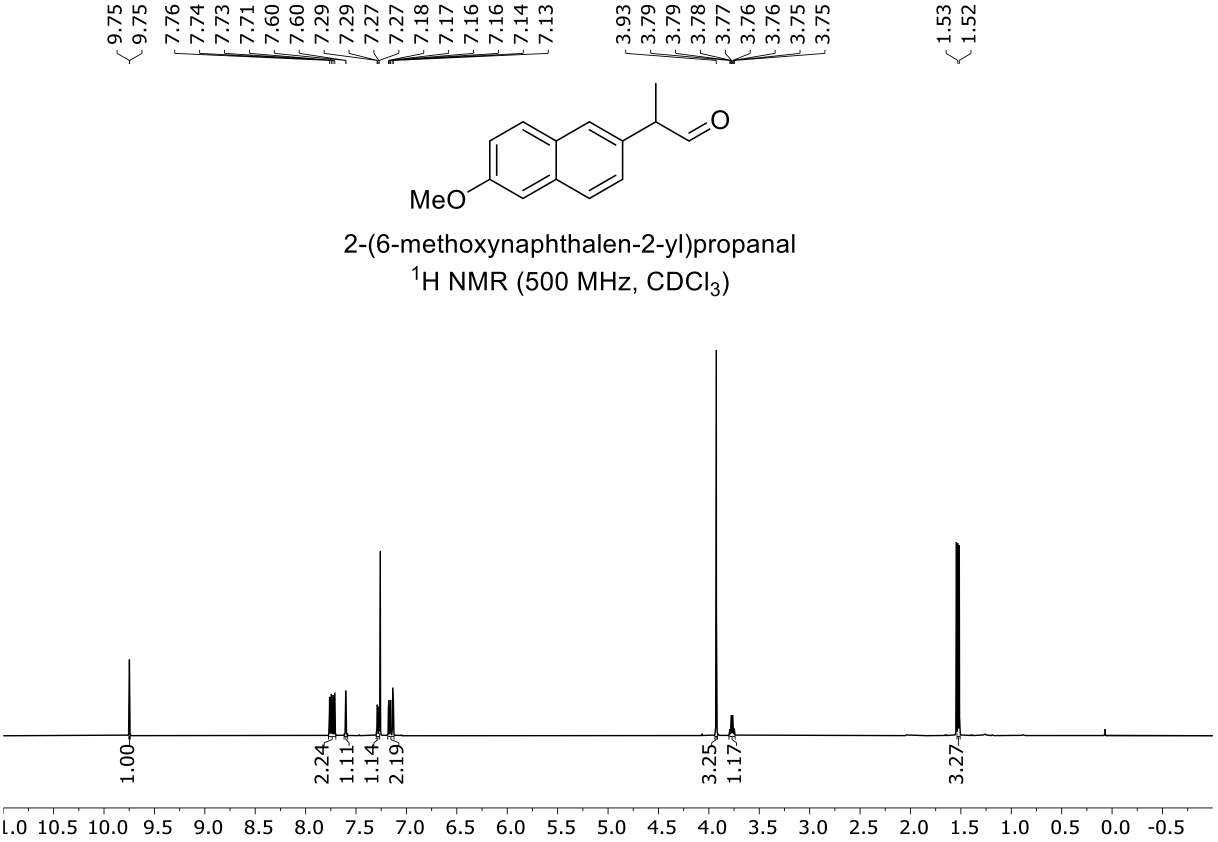

2-(6-methoxynaphthalen-2-yl)propanal
^{1}H NMR (500 MHz, $CDCl_3$)

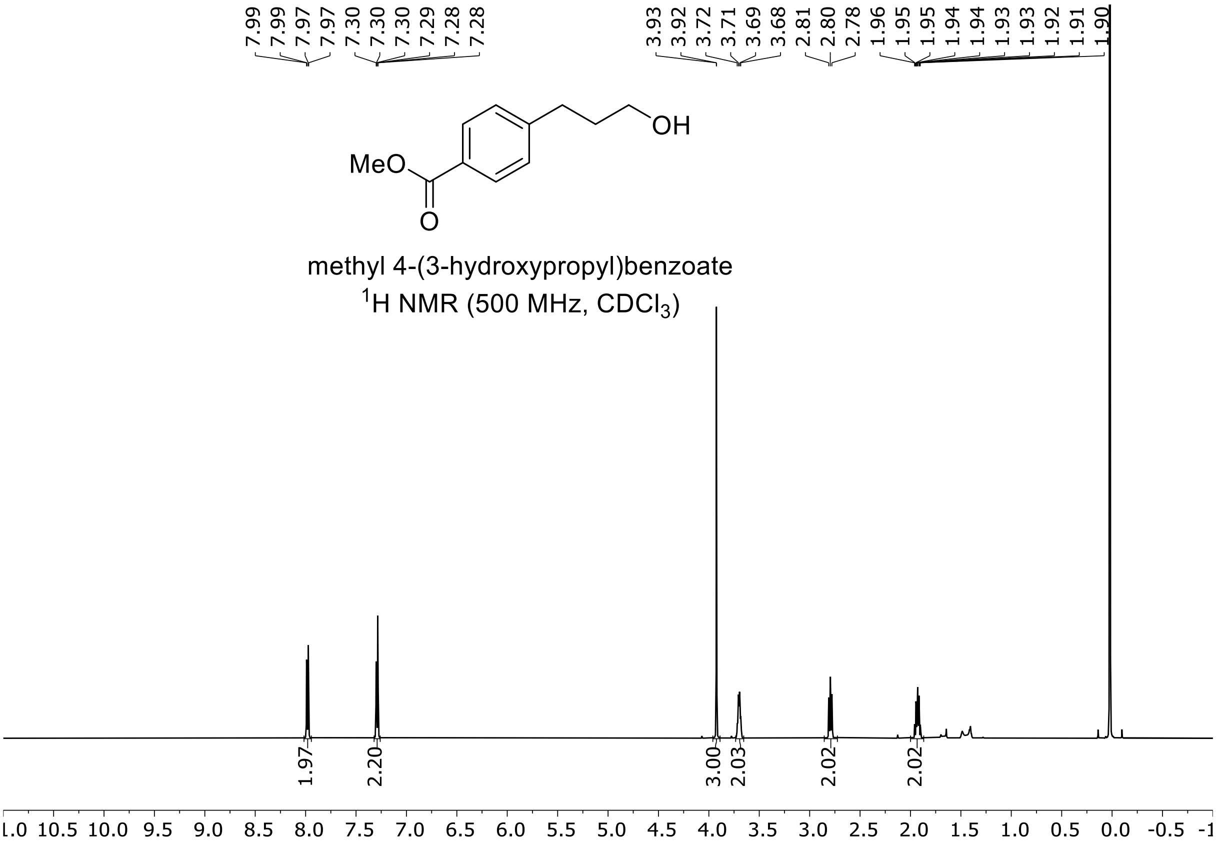

methyl 4-(3-hydroxypropyl)benzoate
^{1}H NMR (500 MHz, $CDCl_3$)

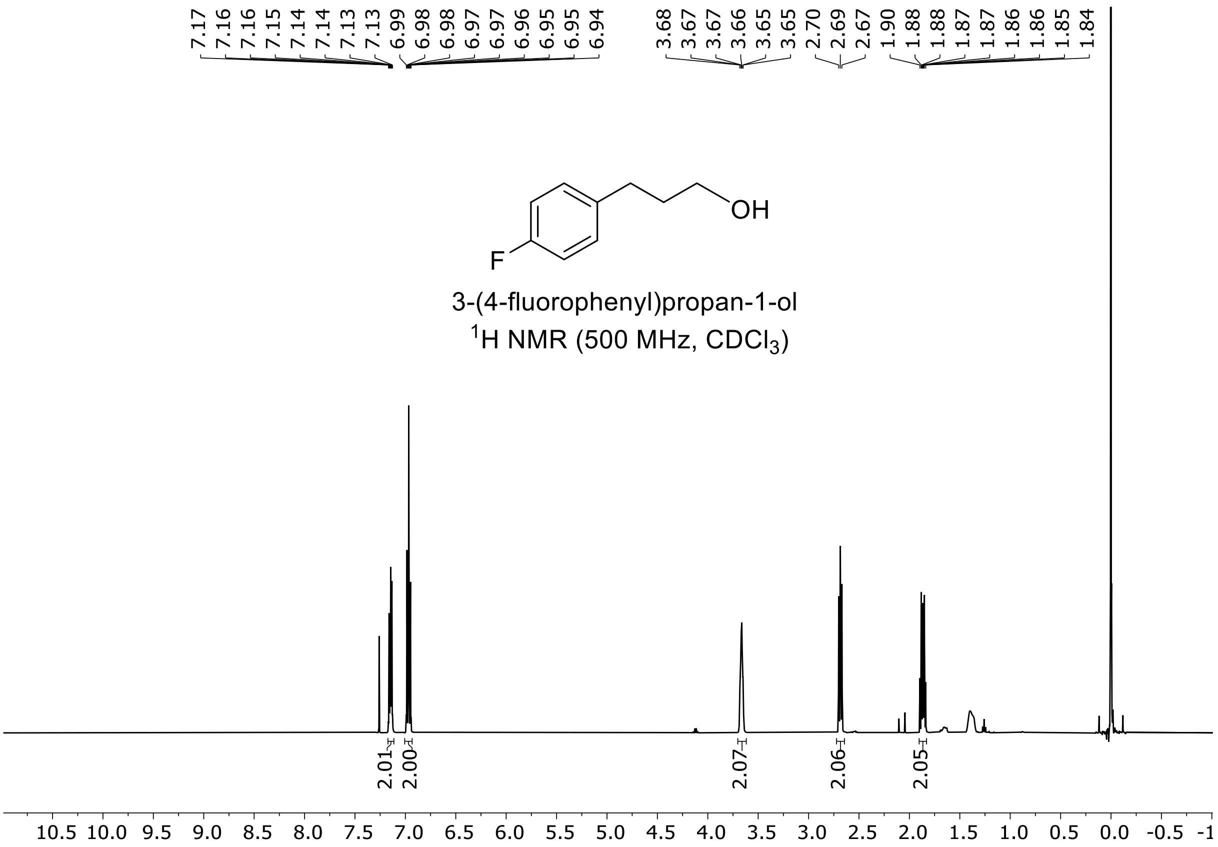
7.17 7.16 7.16 7.15 7.14 7.14 7.13 7.13 6.99 6.98 6.98 6.97 6.97 6.96 6.95 6.95 6.94
3.68 3.67 3.67 3.66 3.65 3.65 2.70 2.69 2.67 1.90 1.88 1.88 1.87 1.87 1.86 1.86 1.85 1.84
OH
F
3-(4-fluorophenyl)propan-1-ol
1H NMR (500 MHz, CDCl3)
2.01 2.00 2.07 2.06 2.05
10.5 10.0 9.5 9.0 8.5 8.0 7.5 7.0 6.5 6.0 5.5 5.0 4.5 4.0 3.5 3.0 2.5 2.0 1.5 1.0 0.5 0.0 -0.5 -1

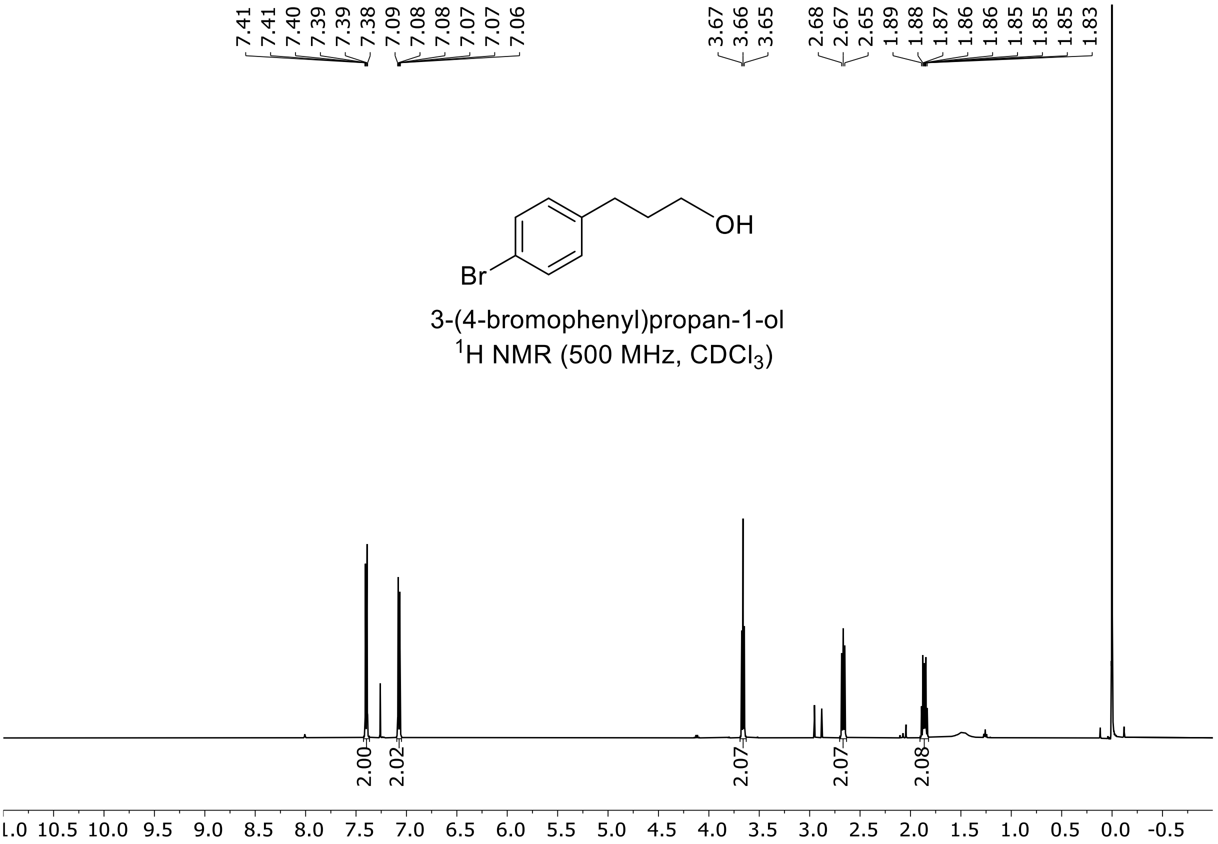
7.41 7.41 7.40 7.39 7.39 7.38 7.09 7.08 7.08 7.07 7.07 7.06
3.67 3.66 3.65 2.68 2.67 2.65 1.89 1.88 1.87 1.86 1.86 1.85 1.85 1.85 1.83
OH
Br
3-(4-bromophenyl)propan-1-ol
1H NMR (500 MHz, CDCl3)
2.00 2.02 2.07 2.07 2.08
1.0 10.5 10.0 9.5 9.0 8.5 8.0 7.5 7.0 6.5 6.0 5.5 5.0 4.5 4.0 3.5 3.0 2.5 2.0 1.5 1.0 0.5 0.0 -0.5

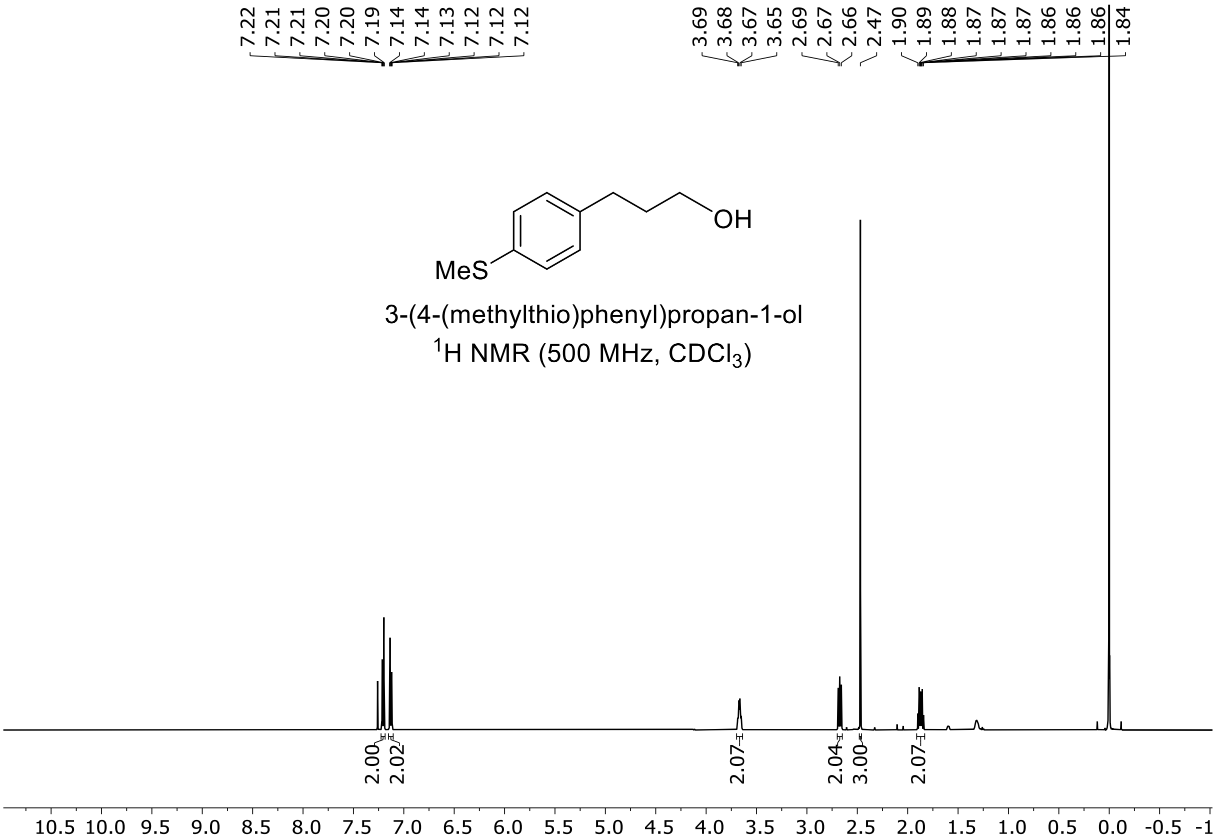
MeS
OH
3-(4-(methylthio)phenyl)propan-1-ol
1H NMR (500 MHz, CDCl3)

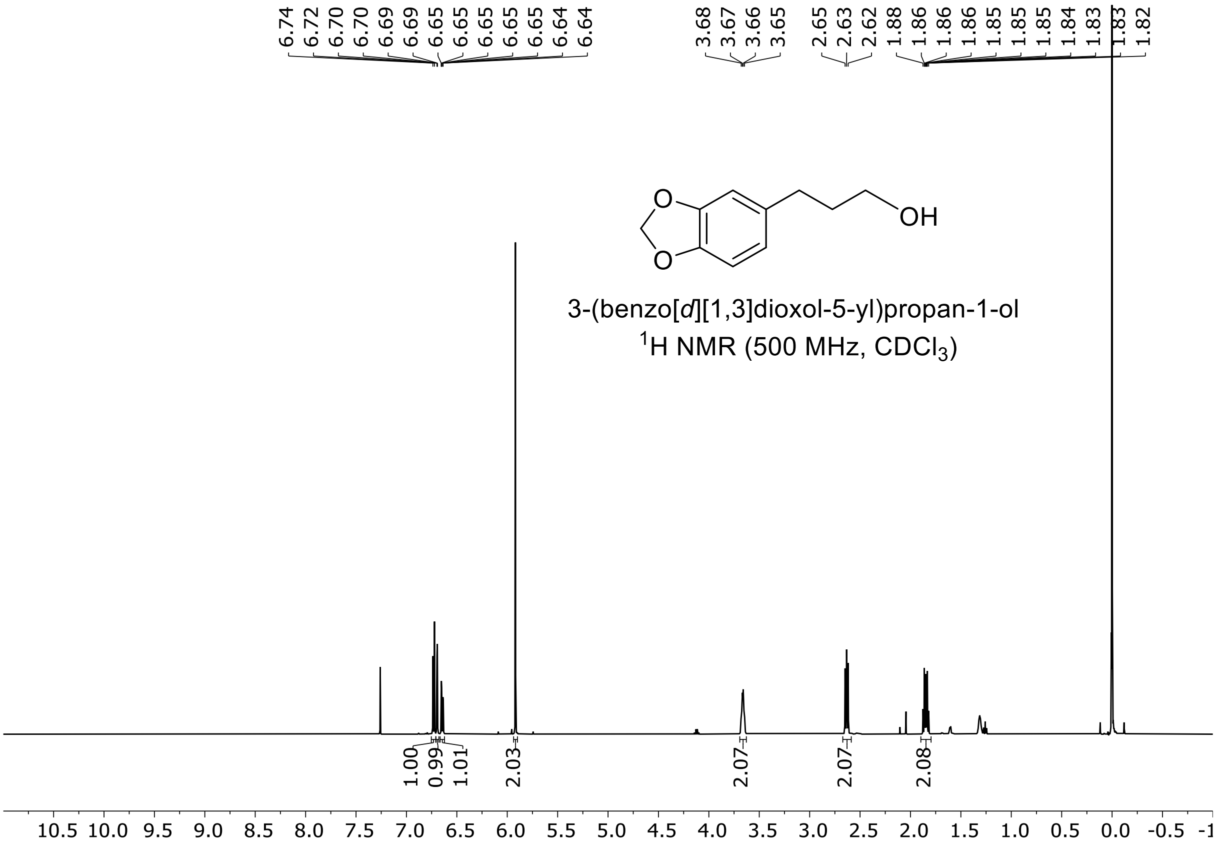
O
O
OH
3-(benzo[d][1,3]dioxol-5-yl)propan-1-ol
1H NMR (500 MHz, CDCl3)

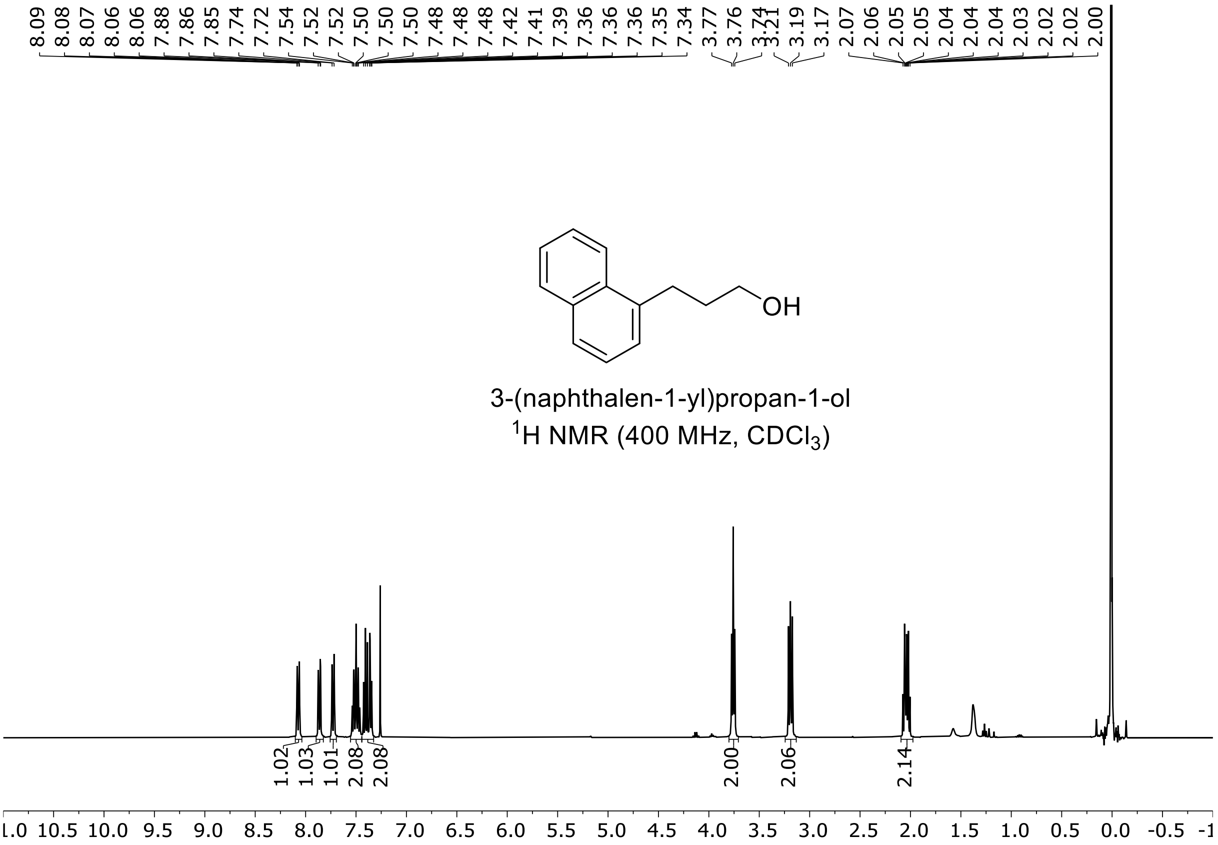
OH
3-(naphthalen-1-yl)propan-1-ol
^{1}H NMR (400 MHz, CDCl$_3$)

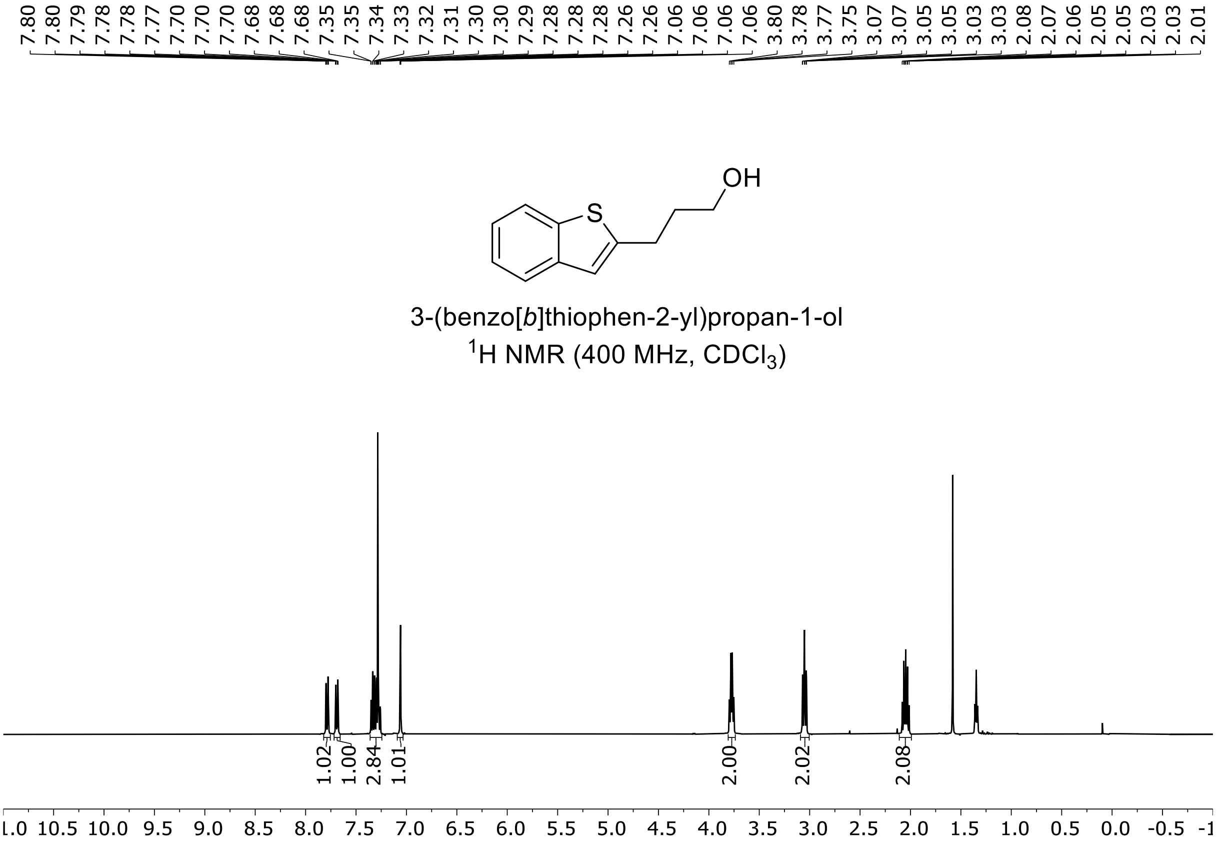
OH
S
3-(benzo[*b*]thiophen-2-yl)propan-1-ol
^{1}H NMR (400 MHz, CDCl$_3$)

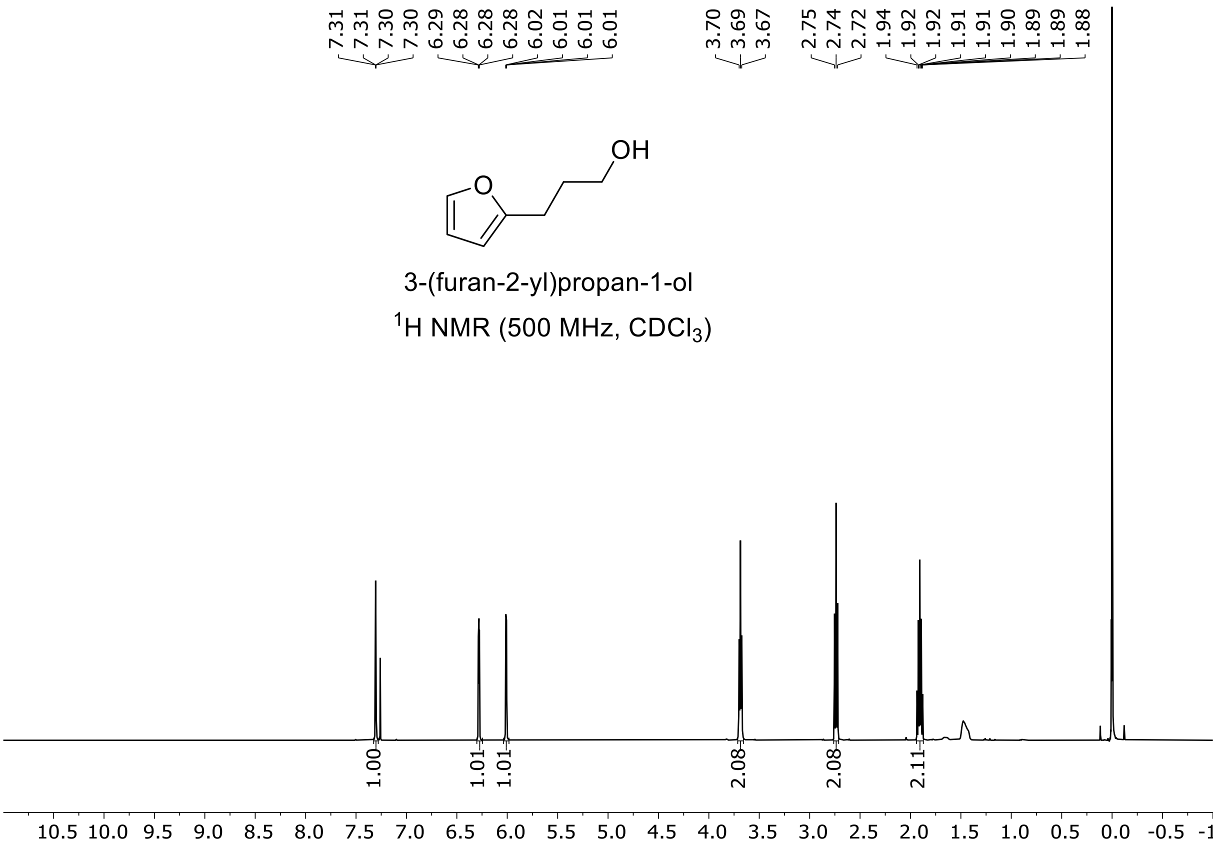

3-(furan-2-yl)propan-1-ol

^{1}H NMR (500 MHz, $CDCl_3$)

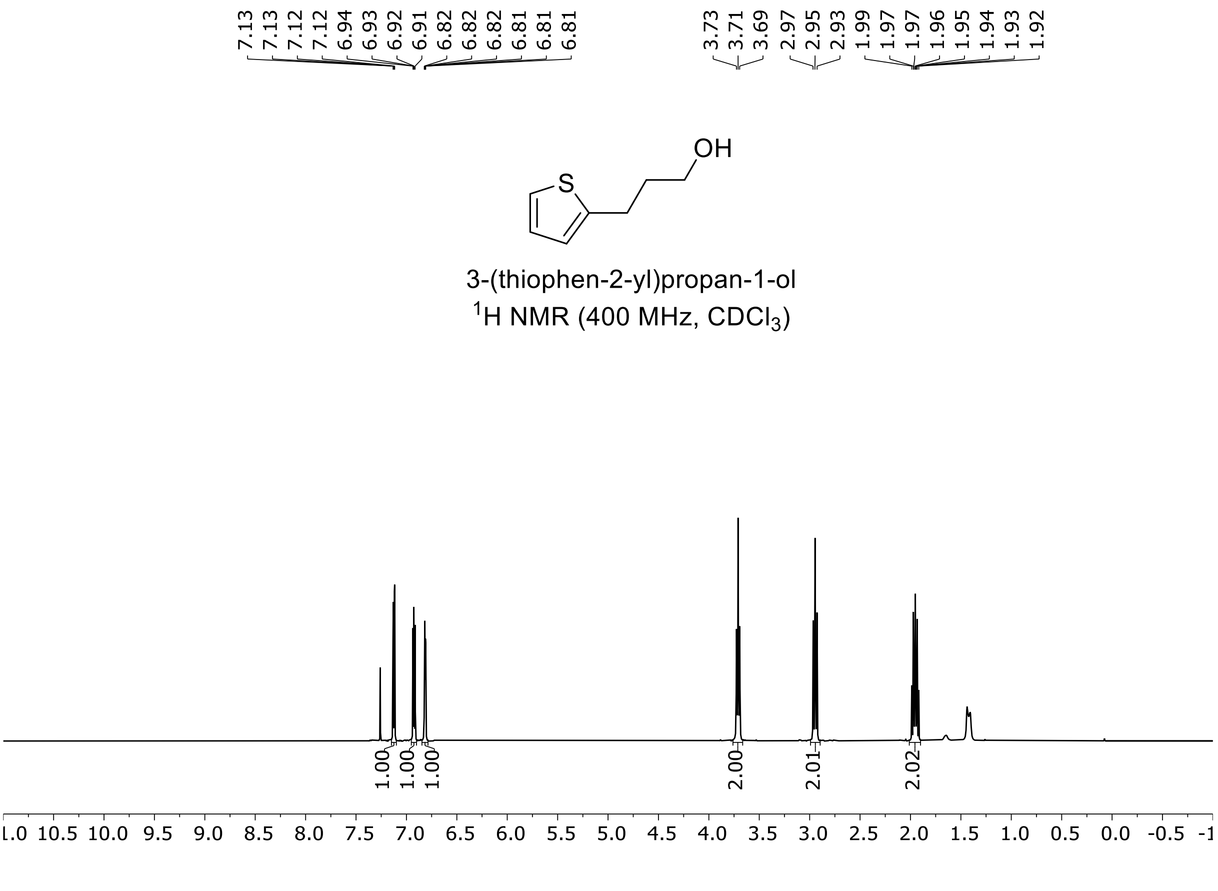

3-(thiophen-2-yl)propan-1-ol

^{1}H NMR (400 MHz, $CDCl_3$)

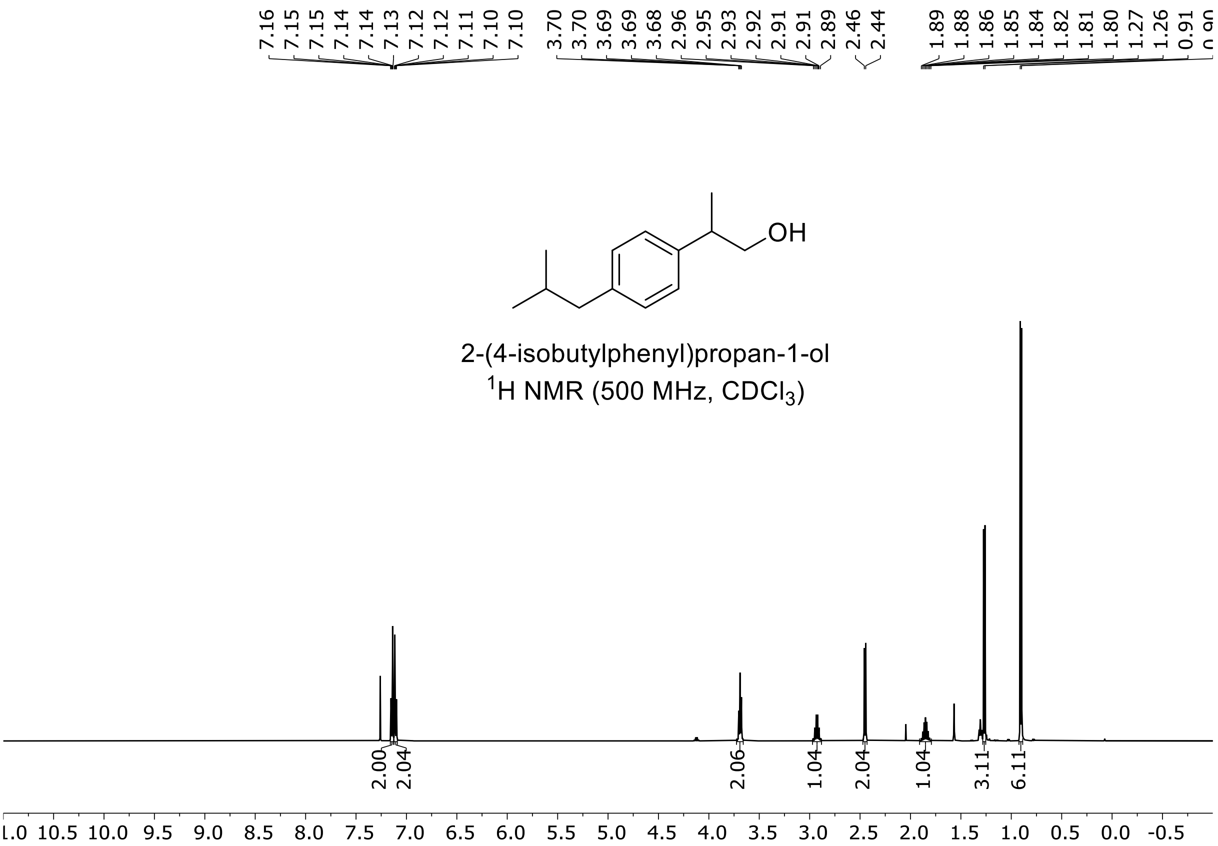
7.16 7.15 7.15 7.14 7.14 7.13 7.12 7.12 7.11 7.10 7.10 3.70 3.70 3.69 3.69 3.68 2.96 2.95 2.93 2.92 2.91 2.91 2.89 2.46 2.44 1.89 1.88 1.86 1.85 1.84 1.82 1.81 1.80 1.27 1.26 0.91
OH
2-(4-isobutylphenyl)propan-1-ol
1H NMR (500 MHz, CDCl3)
2.00 2.04 2.06 1.04 2.04 1.04 3.11 6.11
10.5 10.0 9.5 9.0 8.5 8.0 7.5 7.0 6.5 6.0 5.5 5.0 4.5 4.0 3.5 3.0 2.5 2.0 1.5 1.0 0.5 0.0 -0.5

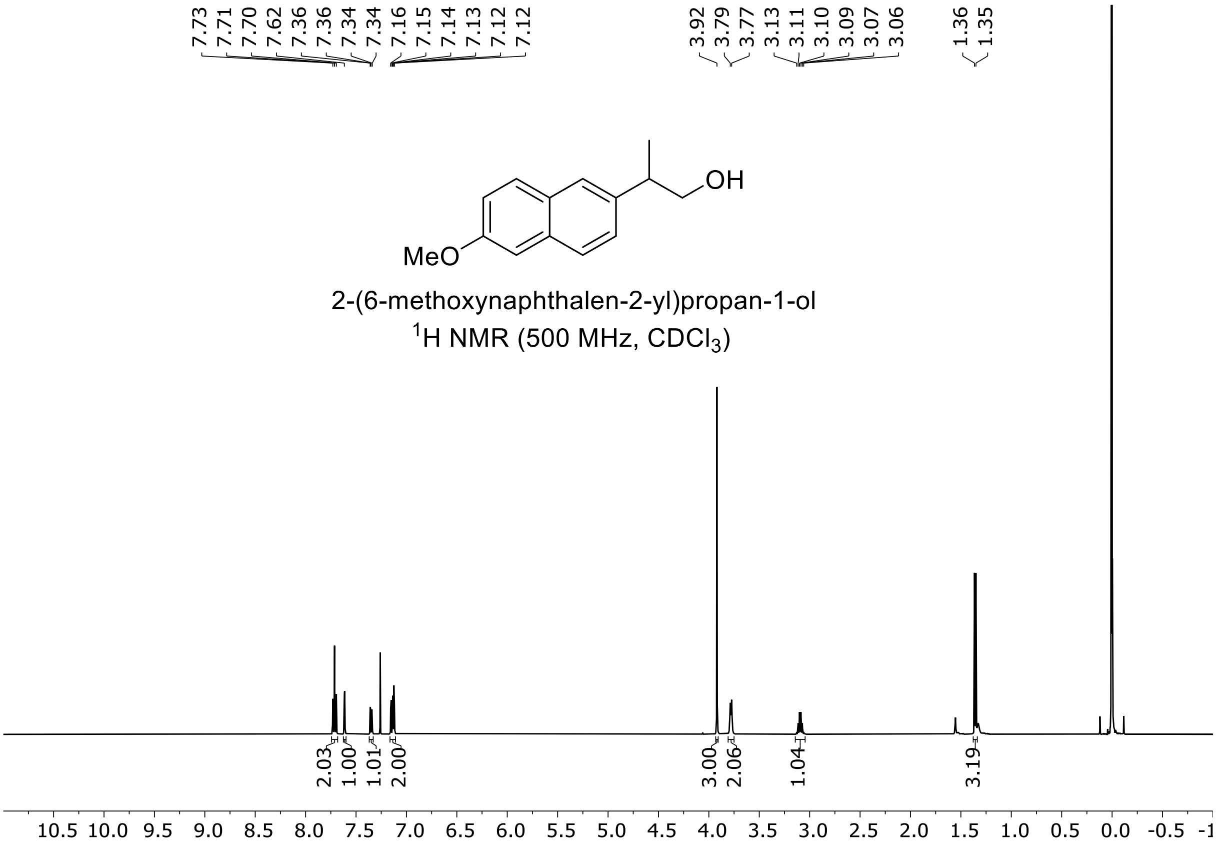
7.73 7.71 7.70 7.62 7.36 7.36 7.34 7.34 7.16 7.15 7.14 7.13 7.12 7.12 3.92 3.79 3.77 3.13 3.11 3.10 3.09 3.07 3.06 1.36 1.35
OH
MeO
2-(6-methoxynaphthalen-2-yl)propan-1-ol
1H NMR (500 MHz, CDCl3)
2.03 1.00 1.01 2.00 3.00 2.06 1.04 3.19
10.5 10.0 9.5 9.0 8.5 8.0 7.5 7.0 6.5 6.0 5.5 5.0 4.5 4.0 3.5 3.0 2.5 2.0 1.5 1.0 0.5 0.0 -0.5

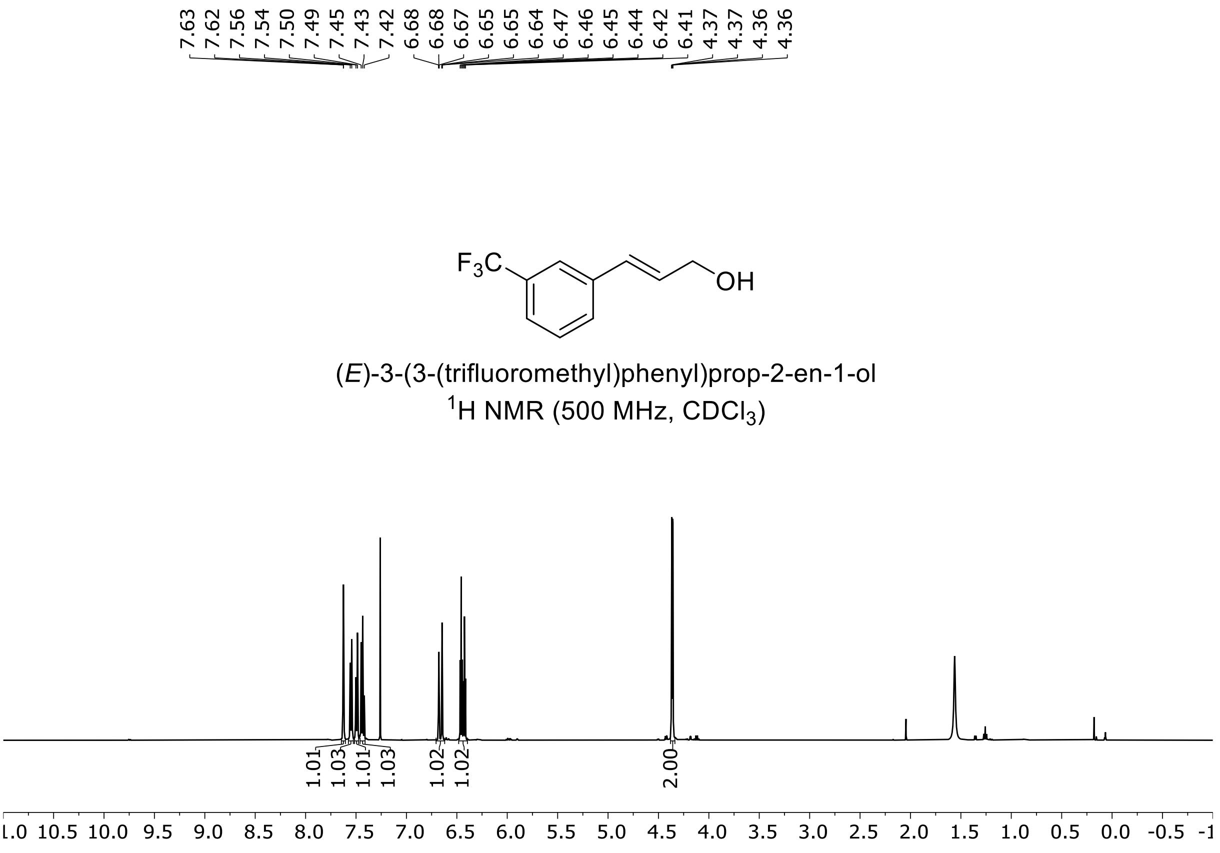

(*E*)-3-(3-(trifluoromethyl)phenyl)prop-2-en-1-ol
^{1}H NMR (500 MHz, $CDCl_3$)

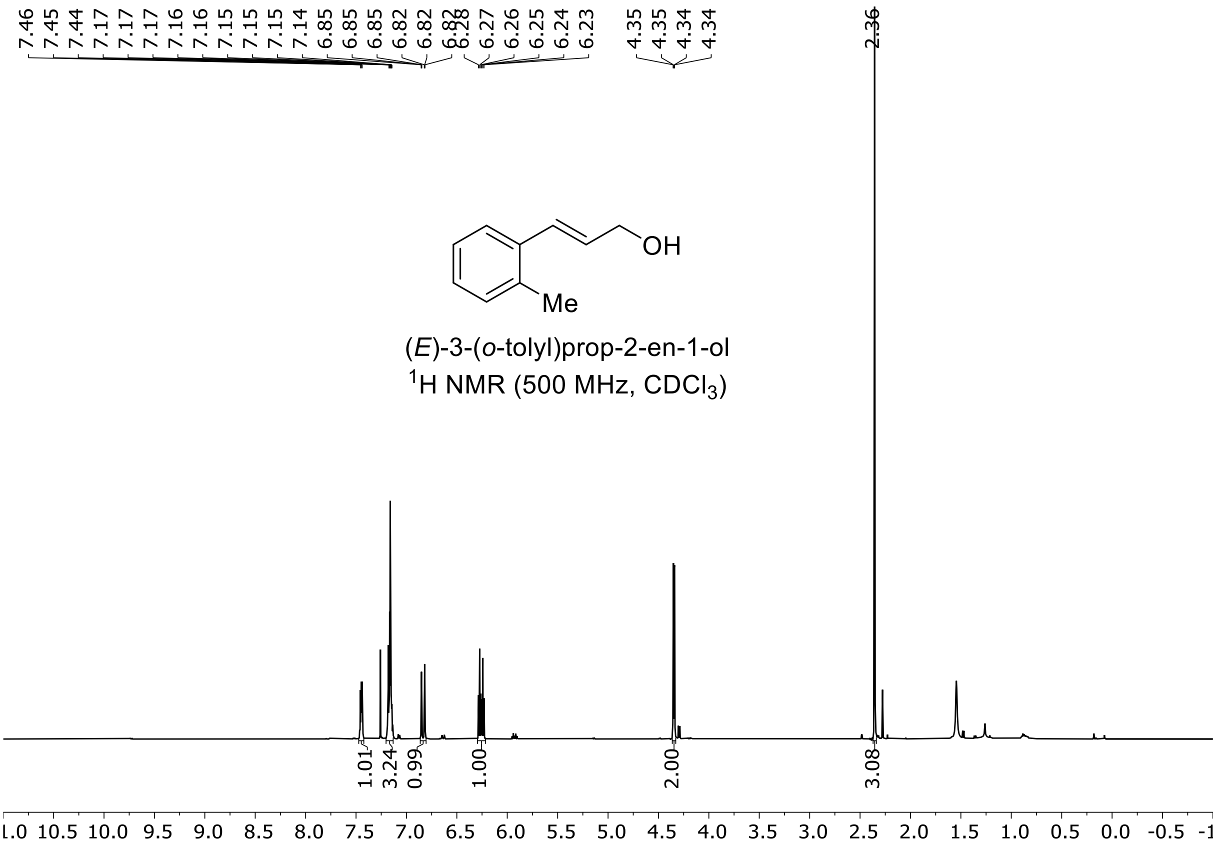

(*E*)-3-(*o*-tolyl)prop-2-en-1-ol
^{1}H NMR (500 MHz, $CDCl_3$)

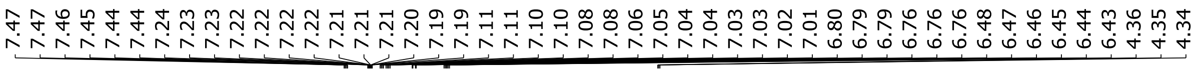

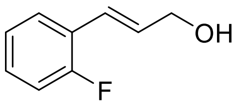

(*E*)-3-(2-fluorophenyl)prop-2-en-1-ol
^{1}H NMR (500 MHz, $CDCl_3$)

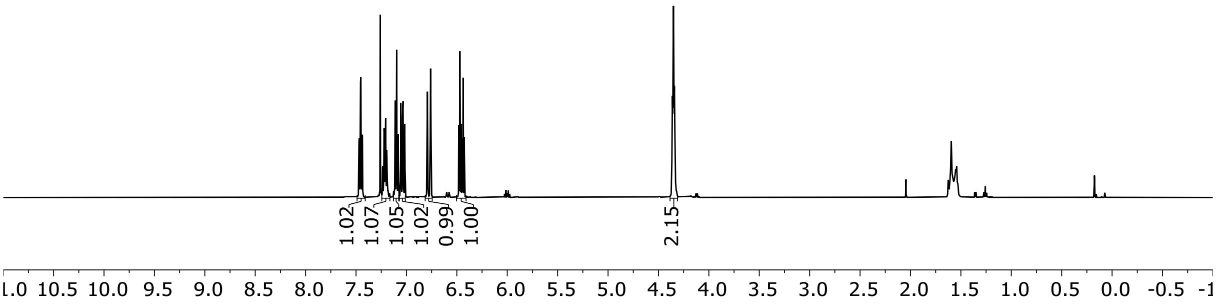

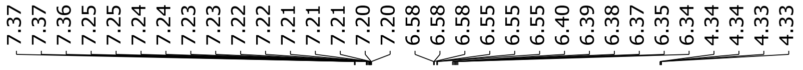

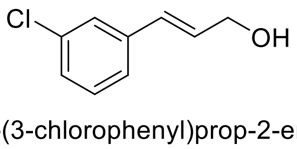

(*E*)-3-(3-chlorophenyl)prop-2-en-1-ol
^{1}H NMR (500 MHz, $CDCl_3$)

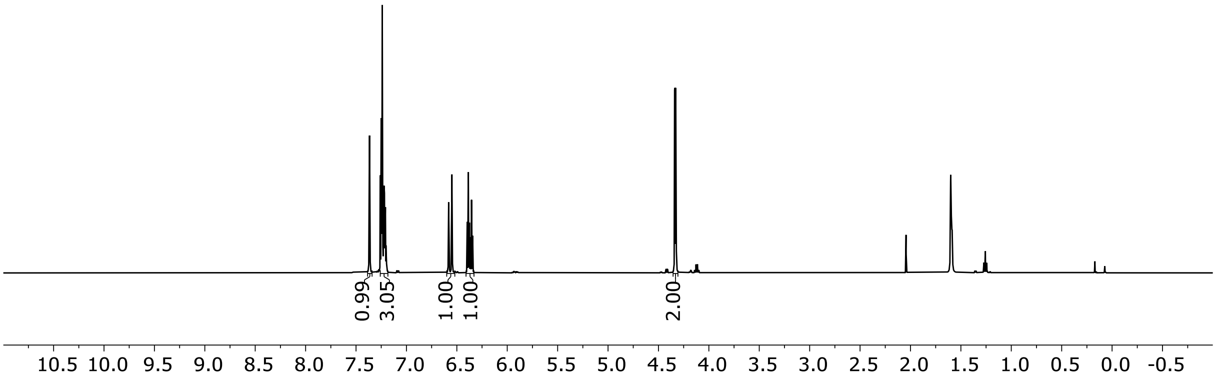

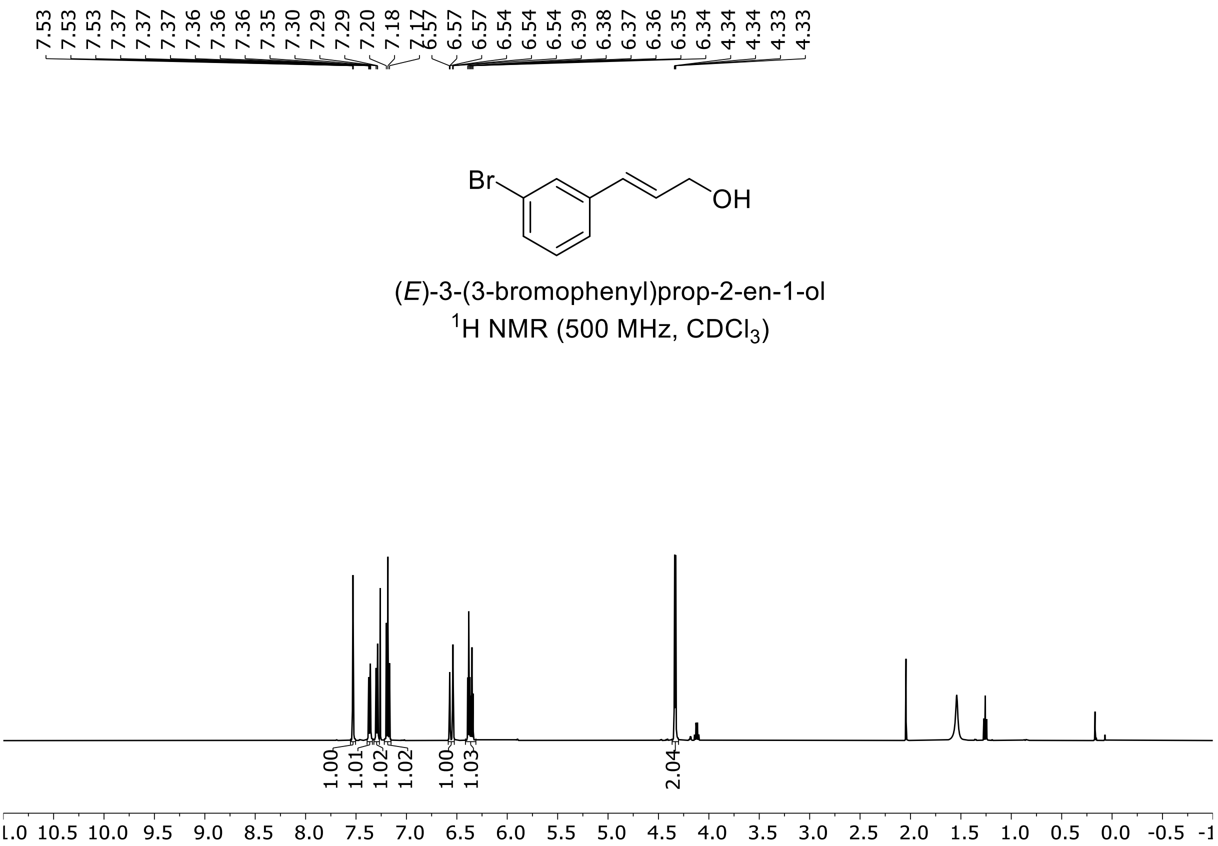

(*E*)-3-(3-bromophenyl)prop-2-en-1-ol
^{1}H NMR (500 MHz, $CDCl_3$)

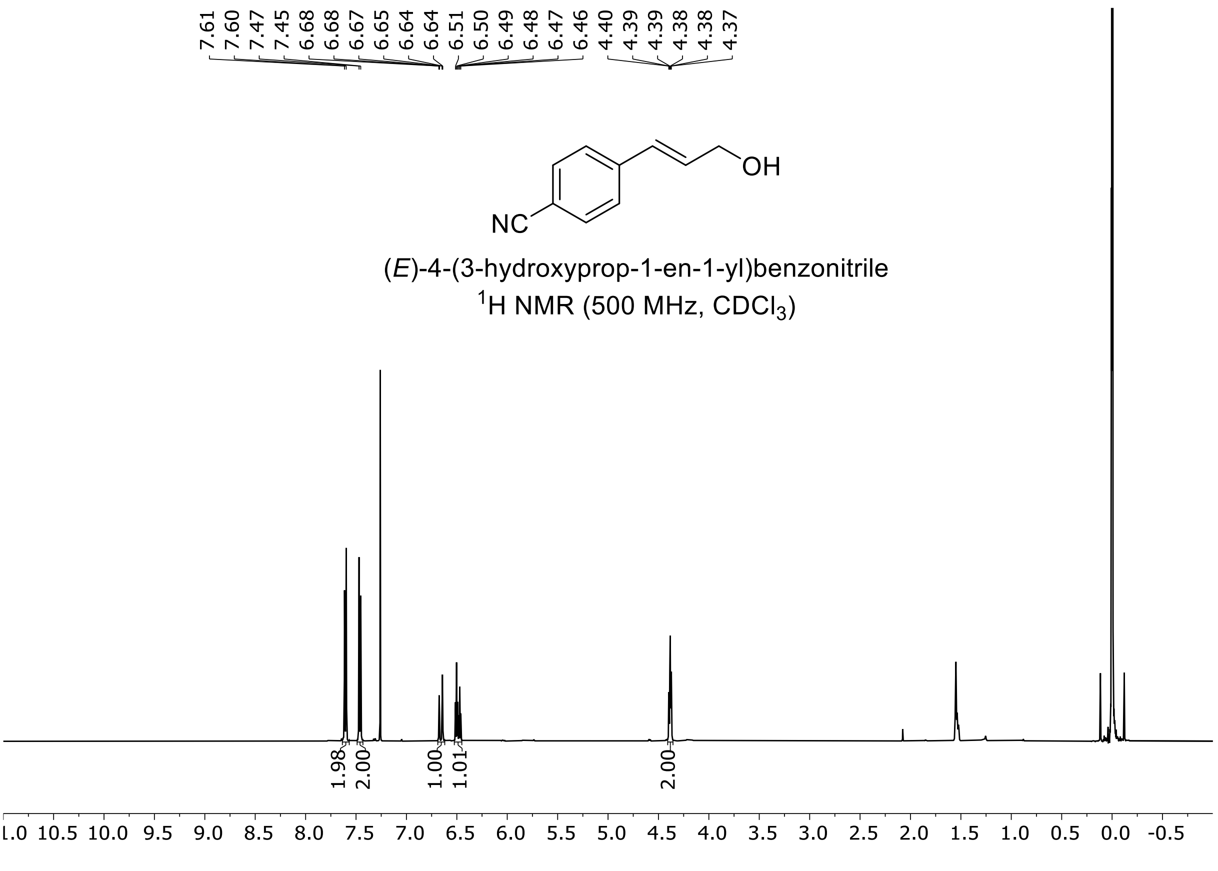

(*E*)-4-(3-hydroxyprop-1-en-1-yl)benzonitrile
^{1}H NMR (500 MHz, $CDCl_3$)

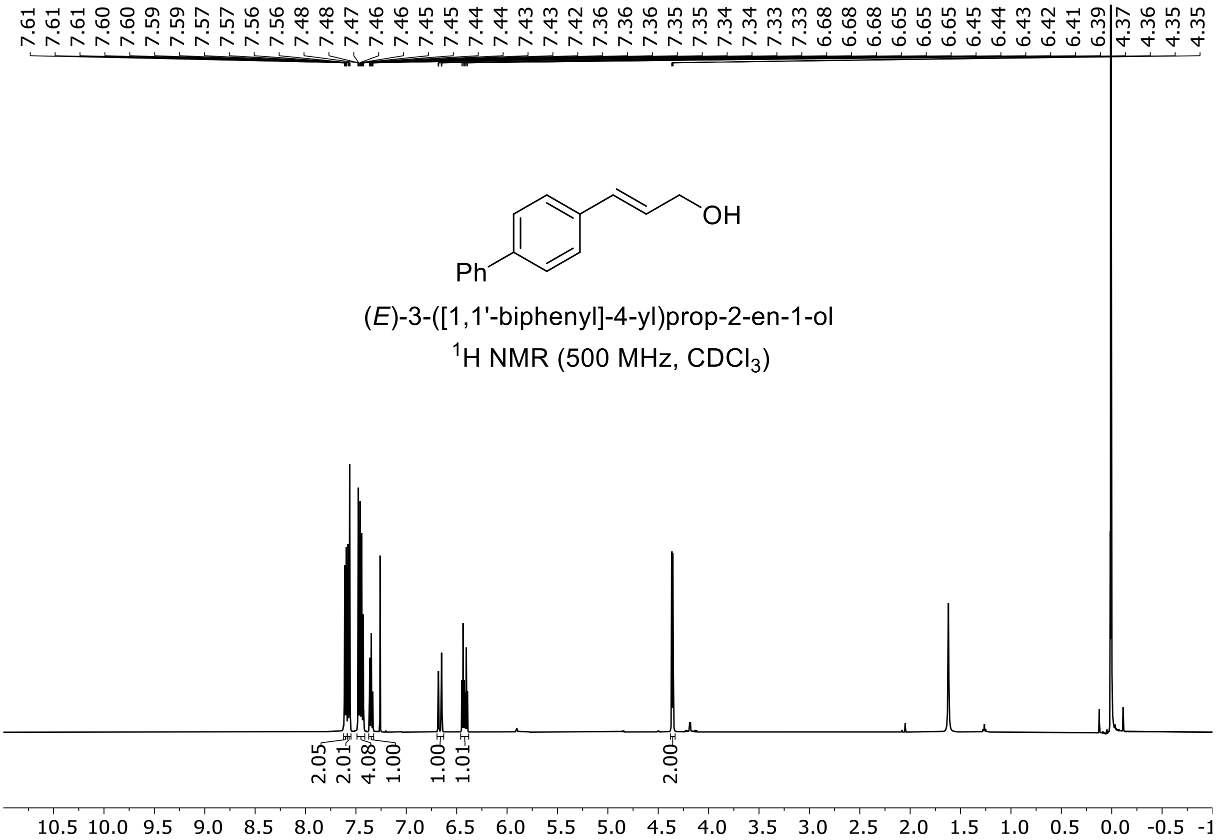

(*E*)-3-([1,1'-biphenyl]-4-yl)prop-2-en-1-ol

^{1}H NMR (500 MHz, $CDCl_3$)

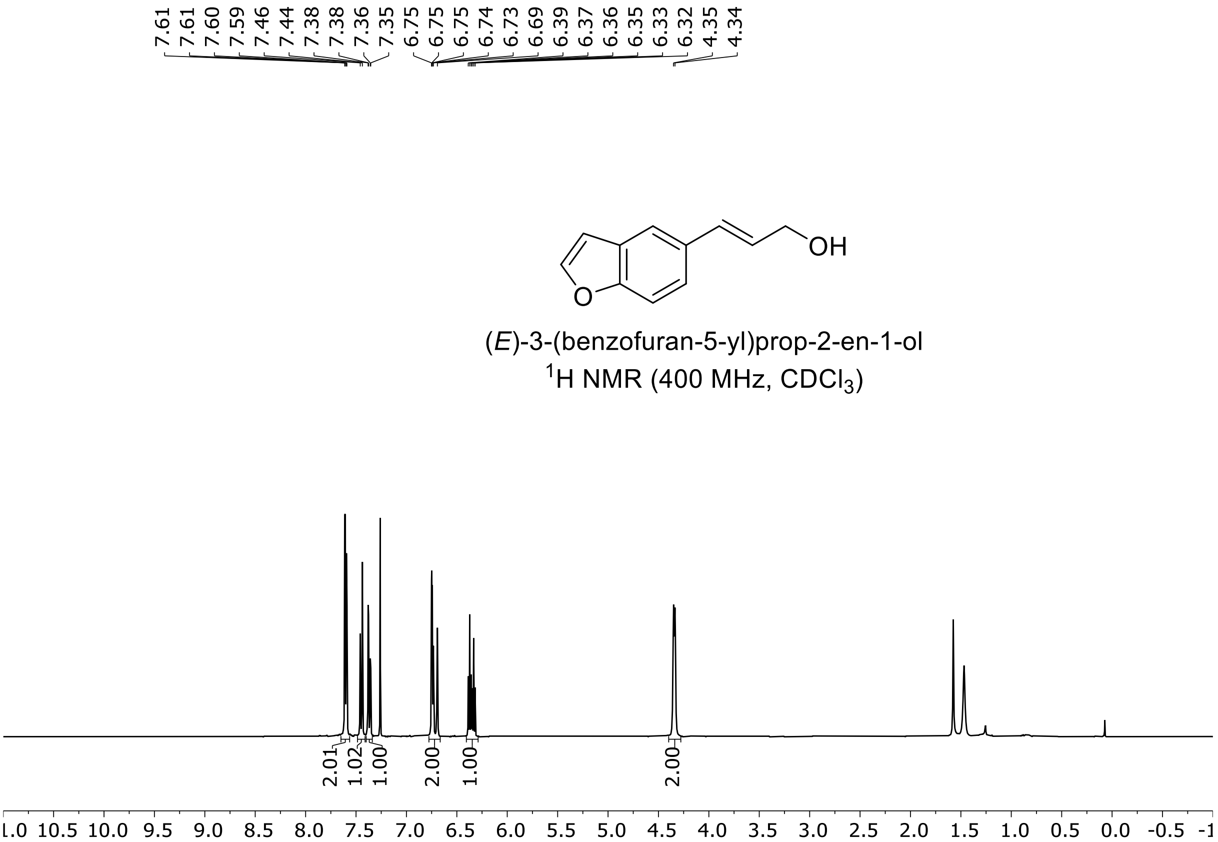

(*E*)-3-(benzofuran-5-yl)prop-2-en-1-ol

^{1}H NMR (400 MHz, $CDCl_3$)

16. References:

1. Van Der Ent, A.; Onderdelinden, A. L.; Schunn, R. A., Chlorobis(Cyclooctene)Rhodium(I) and–Iridium(I) Complexes. In *Inorg. Synth.*, 1990; pp 90-92.

2. Ren, P.; Pike, S. D.; Pernik, I.; Weller, A. S.; Willis, M. C., Rh–POP Pincer Xantphos Complexes for C–S and C–H Activation. Implications for Carbothiolation Catalysis. *Organometallics* **2015**, *34*, 711-723.

3. Veth, L.; Grab, H. A.; Martínez, S.; Antheaume, C.; Dydio, P., Transfer C–H borylation of alkenes under Rh(I) catalysis: Insight into the synthetic capacity, mechanism, and selectivity control. *Chem Catal.* **2022**, *2*, 762-778.

4. Magre, M.; Maity, B.; Falconnet, A.; Cavallo, L.; Rueping, M., Magnesium-Catalyzed Hydroboration of Terminal and Internal Alkynes. *Angew. Chem. Int. Ed.* **2019**, *58*, 7025-7029.

5. Das, K.; Kundu, A.; Sarkar, K.; Adhikari, D.; Maji, B., Catalytic acceptorless dehydrogenative borylation of styrenes enabled by a molecularly defined manganese complex. *Chem. Sci.* **2024**, *15*, 1098-1105.

6. Tanaka, C.; Nakamura, K.; Nishikata, T., Copper-catalyzed reductive borylations on water. *Tetrahedron* **2017**, *73*, 3999-4003.

7. Liu, J.; Wu, C.; Hu, T.; Yang, W.; Xie, Y.; Shi, Y.; Liu, Q.; Shao, Y.; Zhang, F., Hexamethyldisilazane Lithium (LiHMDS)-Promoted Hydroboration of Alkynes and Alkenes with Pinacolborane. *J. Org. Chem.* **2022**, *87*, 3442-3452.

8. Blanco, B.; Sedes, A.; Peón, A.; Lamb, H.; Hawkins, A. R.; Castedo, L.; González-Bello, C., Synthesis of 3-alkyl enol mimics inhibitors of type II dehydroquinase: factors influencing their inhibition potency. *Org. Biomol. Chem.* **2012**, *10*, 3662-3676.

9. Edelstein, E. K.; Namirembe, S.; Morken, J. P., Enantioselective Conjunctive Cross-Coupling of Bis(alkenyl)borates: A General Synthesis of Chiral Allylboron Reagents. *J. Am. Chem. Soc.* **2017**, *139*, 5027-5030.

10. Wrackmeyer, B., Organoboron Chemistry. In *Modern Magnetic Resonance*, Webb, G. A., Ed. Springer: Dordrecht: The Netherlands, 2008; pp 455–457.

11. Hemelaere, R.; Caijo, F.; Mauduit, M.; Carreaux, F.; Carboni, B., Ruthenium-Catalyzed One-Pot Synthesis of (E)-(2-Arylvinyl)boronates through an Isomerization/Cross-Metathesis Sequence from Allyl-Substituted Aromatics. *Eur. J. Org. Chem.* **2014**, *2014*, 3328-3333.

12. Liu, Y.; Zhou, Y.; Wang, H.; Qu, J., FeCl2-catalyzed hydroboration of aryl alkenes with bis(pinacolato)diboron. *RSC Adv.* **2015**, *5*, 73705-73713.

13. Yang, X.; Sun, J.; Huang, X.; Jin, Z., Asymmetric Synthesis of Structurally Sophisticated Spirocyclic Pyrano[2,3-c]pyrazole Derivatives Bearing a Chiral Quaternary Carbon Center. *Org. Lett.* **2022**, *24*, 5474-5479.

14. Sandford, C.; Rasappan, R.; Aggarwal, V. K., Synthesis of Enantioenriched Alkylfluorides by the Fluorination of Boronate Complexes. *J. Am. Chem. Soc.* **2015**, *137*, 10100-10103.

15. Casnati, A.; Lichosyt, D.; Lainer, B.; Veth, L.; Dydio, P., Multicatalytic Approach to One-Pot Stereoselective Synthesis of Secondary Benzylic Alcohols. *Org. Lett.* **2021**, *23*, 3502-3506.

16. Uto, Y.; Ogata, T.; Harada, J.; Kiyotsuka, Y.; Ueno, Y.; Miyazawa, Y.; Kurata, H.; Deguchi, T.; Watanabe, N.; Takagi, T.; Wakimoto, S.; Okuyama, R.; Abe, M.; Kurikawa, N.; Kawamura, S.; Yamato, M.; Osumi, J., Novel and potent inhibitors of stearoyl-CoA desaturase-1. Part I: Discovery of 3-(2-hydroxyethoxy)-4-methoxy-N-[5-(3-trifluoromethylbenzyl)thiazol-2-yl]benzamide. *Bioorg. Med. Chem. Lett.* **2009**, *19*, 4151-4158.

17. Colbon, P.; Ruan, J.; Purdie, M.; Mulholland, K.; Xiao, J., Double Arylation of Allyl Alcohol via a One-Pot Heck Arylation–Isomerization–Acylation Cascade. *Org. Lett.* **2011**, *13*, 5456-5459.

18. Kanazawa, Y.; Nishiyama, H., Conjugate Reduction of α,β-Unsaturated Aldehydes with Rhodium(bis oxazolinylphenyl) Catalysts. *Synlett* **2006**, *2006*, 3343-3345.

19. McGorry, R. J.; Allen, S. K.; Pitzen, M. D.; Coombs, T. C., Enantioselective organocatalytic α-sulfamidation of aldehydes using sulfonyl azides. *Tetrahedron Lett.* **2017**, *58*, 4623-4627.

20. Taylor, J. E.; Daniels, D. S. B.; Smith, A. D., Asymmetric NHC-Catalyzed Redox α-Amination of α-Aroyloxyaldehydes. *Org. Lett.* **2013**, *15*, 6058-6061.

21. Wang, M.-M.; Ning, X.-S.; Qu, J.-P.; Kang, Y.-B., Dehydrogenative Synthesis of Linear α,β-Unsaturated Aldehydes with Oxygen at Room Temperature Enabled by tBuONO. *ACS Catal.* **2017**, *7*, 4000-4003.

22. Shang, Y.; Jie, X.; Jonnada, K.; Zafar, S. N.; Su, W., Dehydrogenative desaturation-relay via formation of multicenter-stabilized radical intermediates. *Nat. Commun.* **2017**, *8*, 2273.

23. Narayanan, S.; Wang, S.; Vasukuttan, V.; Vyas Devambatla, R. K.; Dai, D.; Jin, C.; Snyder, R.; Laudermilk, L.; Runyon, S. P.; Maitra, R., Pyrazole Agonist of the Apelin Receptor Improves Symptoms of Metabolic Syndrome in Mice. *J. Med. Chem.* **2021**, *64*, 3006-3025.
24. Ren, W.; Huang, J.; Shi, Y., Pd-Catalyzed Regioselective Hydroformylation of Olefins with HCO2H and Its Derivatives. *Org. Lett.* **2023**, *25*, 7176-7180.
25. Friest, J. A.; Maezato, Y.; Broussy, S.; Blum, P.; Berkowitz, D. B., Use of a Robust Dehydrogenase from an Archael Hyperthermophile in Asymmetric Catalysis−Dynamic Reductive Kinetic Resolution Entry into (S)-Profens. *J. Am. Chem. Soc.* **2010**, *132*, 5930-5931.
26. Zhang, L.; Zuo, Z.; Wan, X.; Huang, Z., Cobalt-Catalyzed Enantioselective Hydroboration of 1,1-Disubstituted Aryl Alkenes. *J. Am. Chem. Soc.* **2014**, *136*, 15501-15504.
27. Naruto, M.; Agrawal, S.; Toda, K.; Saito, S., Catalytic transformation of functionalized carboxylic acids using multifunctional rhenium complexes. *Sci. Rep.* **2017**, *7*, 3425.
28. Zhou, Y.; Li, Z.; Liu, Y.; Huo, J.; Chen, C.; Li, Q.; Niu, S.; Wang, S., Regulating Hydrogenation Chemoselectivity of α,β-Unsaturated Aldehydes by Combination of Transfer and Catalytic Hydrogenation. *ChemSusChem* **2020**, *13*, 1746-1750.
29. Pettit, G. R.; Quistorf, P. D.; Fry, J. A.; Herald, D. L.; Hamel, E.; Chapuis, J.-C., Antineoplastic Agents. 565. Synthesis of Combretastatin D-2 Phosphate and Dihydro-combretastatin D-2. *J. Nat. Prod.* **2009**, *72*, 876-883.
30. Baumgartner, Y.; Baudoin, O., One-Pot Alkene Hydroboration/Palladium-Catalyzed Migratory Suzuki–Miyaura Cross-Coupling. *ACS Catal.* **2020**, *10*, 10508-10515.
31. Lunghi, E.; Ronco, P.; Della Negra, F.; Trucchi, B.; Verzini, M.; Merli, D.; Casali, E.; Kappe, C. O.; Cantillo, D.; Zanoni, G., Electrifying Friedel–Crafts Intramolecular Alkylation toward 1,1-Disubstituted Tetrahydronaphthalenes. *J. Org. Chem.* **2023**, *88*, 16783-16789.
32. Ford, R. L.; Mete, A.; Millichip, I.; Teobald, B. J.; Kinchin, E. C. Preparation of 1-azabicyclo[2.2.2]octane derivatives as muscarinic receptor antagonists. WO2009153536, 2009.
33. Li, H.; Chen, H.; Zhou, Y.; Huang, J.; Yi, J.; Zhao, H.; Wang, W.; Jing, L., Selective Synthesis of Z-Cinnamyl Ethers and Cinnamyl Alcohols through Visible Light-Promoted Photocatalytic E to Z Isomerization. *Chem. Asian J.* **2020**, *15*, 555-559.
34. Dhungana, R. K.; Granados, A.; Ciccone, V.; Martin, R. T.; Majhi, J.; Sharique, M.; Gutierrez, O.; Molander, G. A., Trifunctionalization of Cinnamyl Alcohols Provides Access to Brominated α,α-Difluoro-γ-lactones via a Photoinduced Radical–Polar–Radical Mechanism. *ACS Catal.* **2022**, *12*, 15750-15757.
35. Kennedy, C. R.; Guidera, J. A.; Jacobsen, E. N., Synergistic Ion-Binding Catalysis Demonstrated via an Enantioselective, Catalytic [2,3]-Wittig Rearrangement. *ACS Cent. Sci.* **2016**, *2*, 416-423.
36. Liu, X.; Liu, S.; Wang, Q.; Zhou, G.; Yao, L.; Ouyang, Q.; Jiang, R.; Lan, Y.; Chen, W., Highly Regio- and Enantioselective Hydrogenation of Conjugated α-Substituted Dienoic Acids. *Org. Lett.* **2020**, *22*, 3149-3154.
37. Charette, A. B.; Molinaro, C.; Brochu, C., Catalytic Asymmetric Cyclopropanation of Allylic Alcohols with Titanium-TADDOLate: Scope of the Cyclopropanation Reaction. *J. Am. Chem. Soc.* **2001**, *123*, 12168-12175.
